# Controlled partial transfer hydrogenation of quinolines by cobalt-amido cooperative catalysis

**DOI:** 10.1038/s41467-020-15118-x

**Published:** 2020-03-06

**Authors:** Maofu Pang, Jia-Yi Chen, Shengjie Zhang, Rong-Zhen Liao, Chen-Ho Tung, Wenguang Wang

**Affiliations:** 1 Key Lab of Colloid and Interface Chemistry, Ministry of Education, School of Chemistry and Chemical Engineering, Shandong University, No. 27 South Shanda Road, Jinan, 250100 P. R. China; 2 School of Chemistry and Chemical Engineering, Huazhong University of Science and Technology, 1037 Luoyu Road, Wuhan, 430074 China

**Keywords:** Catalytic mechanisms, Homogeneous catalysis, Synthetic chemistry methodology

## Abstract

Catalytic hydrogenation or transfer hydrogenation of quinolines was thought to be a direct strategy to access dihydroquinolines. However, the challenge is to control the chemoselectivity and regioselectivity. Here we report an efficient partial transfer hydrogenation system operated by a cobalt-amido cooperative catalyst, which converts quinolines to 1,2-dihydroquinolines by the reaction with H_3_N·BH_3_ at room temperature. This methodology enables the large scale synthesis of many 1,2-dihydroquinolines with a broad range of functional groups. Mechanistic studies demonstrate that the reduction of quinoline is controlled precisely by cobalt-amido cooperation to operate dihydrogen transfer from H_3_N·BH_3_ to the N=C bond of the substrates.

## Abstract (web summary)

Controlling the partial reduction of quinolines is challenging, given the competing overreduction to tetrahydroquinolines. Here, the authors report a cobalt-amido cooperative catalyst for the selective, partial transfer hydrogenation of quinolines to 1,2-dihydroquinolines with H_3_N·BH_3_ as reductant.

## Introduction

**Fig. 1:**
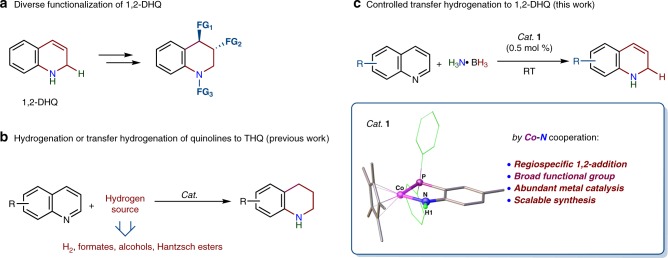
Conversion of quinolines to THQs and 1,2-DHQs. **a** Diverse functionalization of 1,2-DHQ. **b** Hydrogenation and transfer hydrogenation of quinolines to THQs. **c** The present work on transfer hydrogenation of quinolines with H_3_N·BH_3_ to 1,2-DHQs by a cobalt-amido cooperative catalyst.

Dihydroquinolines (DHQs) are a potential surrogate of H_2_ for sustainable transformations, reminiscent of the structure of reduced nicotinamide adenine dinucleotide phosphate (NADPH)^1–4^, More importantly, 1,2-DHQs are versatile synthons leading to bioactive molecules^5^, pharmaceuticals^6,7^, natural products^8–10^, and organic electroluminescent materials^11^. They can be conveniently transformed by C–H functionalization^12^ to complex organic structures, and can achieve asymmetric olefin difunctionalization^13,14^, and *N*-functionalization^15^ (Fig. 1a). Catalytic regioselective hydrogenation and transfer hydrogenation of *N*-heteroarenes have attracted intense interest^16–25^, and could provide the most straightforward protocol with which to harvest the desired DHQs. However, catalytic transformation of quinolines to DHQs is extremely challenging.

Significant progress has been made in hydrogenation of quinolines to tetrahydroquinolines (THQs)^26–29^, and transfer hydrogenation reactions with formates^30^, alcohols^31,32^ or Hantzsch esters^33–35^ (Fig. 1b), but the related catalysis to access DHQs has not been revealed to date. The difficulty associated with such catalysis to access 1,2-DHQs is control of chemoselectivity and regioselectivity since these reactions always suffer from over-reduction of the more reactive DHQs to THQs^16,36^. The breakthrough in this field was catalytic hydrosilylation and hydroboration of quinolines to the *N*-silylated or *N*-borylated 1,2-DHQs^37–44^ respectively, using transition metal catalysts or metal-free organocatalysts^45,46^. Deprotection of the silyl or boryl groups of the *N*-protected DHQs can provide an alternative route to 1,2-DHQs, however, such an *N*-protection/hydrolysis strategy encounter issues related to functional group compatibility and the complicated purification procedures^39^, which limits the expansion of 1,2-DHQ chemistry.

Synergism of metal-ligand reactivity is a practical strategy with which to design new catalysts to perform precise transformations^47–54^. For example, cooperative Ru–S reactivity allows for activation of the Si–H bond of hydrosilanes and catalytic intermolecular electrophilic C−H silylation of *N*-protected indoles to afford the corresponding C3-silylated indoles as single regioisomers^55–57^. In particular, such metal-ligand cooperation strategy greatly promotes the utility of the abundant metals for hydrogenation and transfer hydrogenation of unsaturated hydrocarbons^58–63^. However, the related catalysis has not been reported for the direct synthesis of DHQs.

In this work, we report an efficient cobalt-amido cooperative catalyst for the controlled, partial transfer hydrogenation of quinolines to 1,2-DHQs with H_3_N∙BH_3_ (Fig. 1c). Compared with the previous methods of catalytic hydrosilylation and hydroboration, the functional group tolerance in the present catalysis is exceedingly broad, in particular, with respect to ester, amide, and alkenyl moieties. Notably, tetrahydrogenation of the quinoline substrate can be also realized by using two equiv. of reducing agent under the identical reaction conditions for the catalytic dihydrogenation. Additionally, we have demonstrated that 1,2-DHQs are valuable starting material to synthesize chiral THQs, and they can serve as reducing agent for dihydrogen transfer.

## Results

### Synthesis and reactivity of 1

The phosphinoamido cobalt(II) half-sandwich complex (**1**) was easily prepared by treatment of [Cp*CoCl]_2_ with lithium (2-(diphenylphosphanyl)-4-methyl-phenyl)amide in THF^64^. Single crystal X-ray diffraction confirmed its structure as a neutral cobalt(II) compound with a 17-electron configuration. This compound was initially found to react gently with H_3_N∙BH_3_, gradually releasing H_2_ at room temperature. In particular, **1** is capable of catalyzing this dehydrogenation reaction. With a catalyst loading of 0.5 mol%, a solution in THF of H_3_N∙BH_3_ in 10 h released a nearly equimolar quantity of H_2_ based on GC quantification (see Supplementary Fig. 1).

### Partial transfer hydrogenation of 4-methylquinoline

**Fig. 2:**
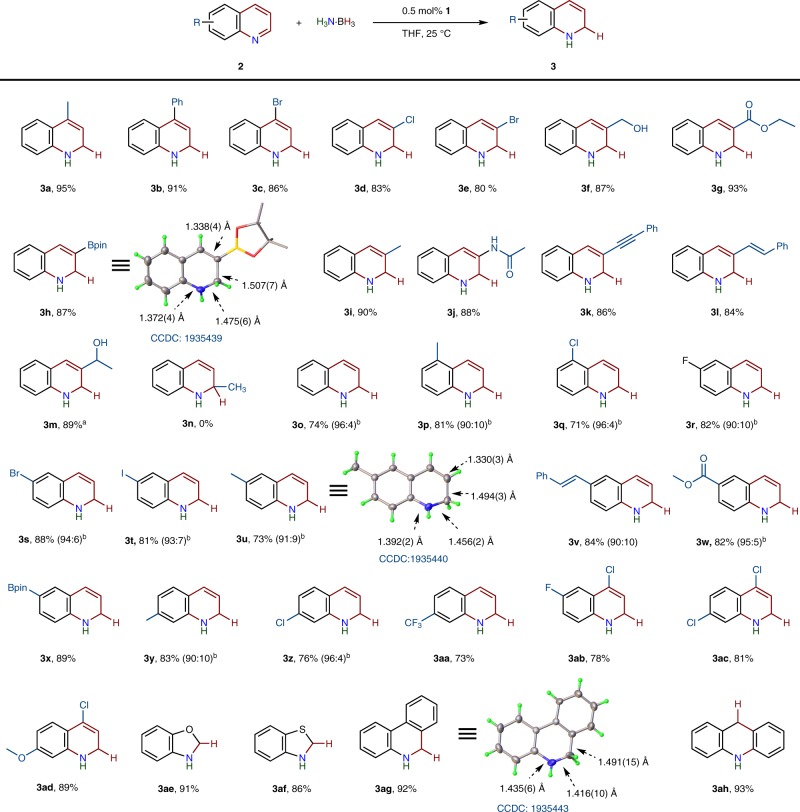
Substrate scope. Reaction conditions: substrate (0.5 mmol), H_3_N∙BH_3_ (0.55 mmol) and **1** (0.5 mol%) were stirred in THF (2 mL) at 25 ^o^C for 20 h. Isolated yields are given. ^a^Two equiv. of H_3_N·BH_3_ (1 mmol) was used for reduction of 3-acetylquinoline. ^b^Ratios in parentheses refer to product ratios of 1,2-DHQ and THQ determined by ^1^H NMR spectroscopy.

Based on the reactivity of **1** toward H_3_N∙BH_3_, the transfer hydrogenation of 4-methylquinoline (**2a**) with H_3_N∙BH_3_ was examined using **1** as a catalyst (see Supplementary Table 1). With 5 mol% of **1** in *d*_8_-toluene, the reaction of the quinoline (**2a**) with 1.1 equiv of H_3_N∙BH_3_ was conducted in a Young NMR tube. According to the ^1^H NMR spectroscopic analysis, the reaction conducted at 25 ^o^C provided 4-methyl-1,2-dihydroquinoline (**3a**) in 36% yield in 4 h. Control experiments indicated that the cobalt complex is responsible for the catalysis, no reaction being observed in the absence of **1**. Remarkably, in THF the quinoline substrate was converted nearly quantitatively to **3a** within 3 h. This system is extremely effective; with even 0.5 mol% of **1**, the yield of **3a** reaches 99% within 20 h. Scaling up of the transfer hydrogenation failed to decrease the yield and pure **3a** was isolated in 95% yield (see Fig. 2).

Using 0.5 mol% of **1** in THF at 25 ^o^C, under the optimized reaction conditions, we investigated the substrate scope for the catalysis (Fig. 2). After changing the methyl group to a phenyl or bromo group, the corresponding 4-substituted-1,2-DHQs (**3b**, **3c**) were obtained with good yields. Notably, quinolines with a variety of functional groups such as Cl (**2d**), Br (**2e**), CH_2_OH (**2f**), COOC_2_H_5_ (**2g**), NHCOCH_3_ (**2j**), Bpin (**2h**), C=CPh (**2l**) and C≡CPh (**2k**) at the 3-position all underwent smooth transfer hydrogenation to furnish the dearomatized products (**3d**-**3l**). The synthesis of 1,2-DHQs with ester substituents (**3g** and **3h**) or an amide group (**3j**) is challenging since they are prone to hydrolysis during the deprotection of the *N*-silylated or *N*-borylated dihydroquinoline precursors^39^. Unlike the ester groups, the acyl group was hydrogenated in the course of partial transfer hydrogenation of the *N*-heterocycle. The reaction of 3-acetylquinoline (**2m**) with two equiv of H_3_N∙BH_3_ produced the corresponding hydroxyl compound (**3m**) in 89% yield. The reduction of 2-methylquinoline failed, probably due to the steric effect on the 1,2-hydrogenation (**3n**)^41^. Transfer hydrogenation of quinoline produced 1,2-DHQ (**3o**), which was obtained as solid.

In general, the regioselective 1,2-reduction of quinolines is not impeded by the electronic or steric nature of functional groups on the aryl ring. A variety of functionalized 1,2-DHQs (**3p**-**3ad**) were obtained in good to excellent yield with this mild catalytic protocol. In particular, halogen substituted quinolines tolerate the catalysis, as exemplified by **3c** (86%), **3d** (83%), **3e** (80%)**, 3q** (71%)**, 3r** (82%)**, 3s** (88%)**, 3t** (81%), **3z** (76%), **3aa** (73%), **3ab** (78%), **3ac** (81%) and **3ad** (89%). Although cobalt-based transfer hydrogenations of alkynes and alkenes with H_3_N∙BH_3_ have been reported^65,66^, the dihydrogenation reaction occurs selectively at the C=N bond of the *N*-heterocyclic ring (**3k**, **3l**, and **3v**). This cobalt-based catalytic reaction is not limited only to quinolines, and it can also efficiently process the transfer hydrogenation of benzo[d]oxazole, benzothiazole, phenanthridine and acridine, providing the desired products (**3ae**–**3ah**) with excellent yields. This protocol is extremely efficient and most of 1,2-DHQs were conveniently synthesized on a large scale and purified by crystallization. Crystal structures of **3h**, **3u** and **3ag** were unequivocally established by single-crystal X-ray diffraction (Fig. 2). In the dihydrogenation of quinolines with substituents on the phenyl ring, THQs were also observed as by-products (<10% yield) which arise from further reduction of the resultant 1,2-DHQs (see below).

### Transfer hydrogenation to THQs

**Fig. 3:**
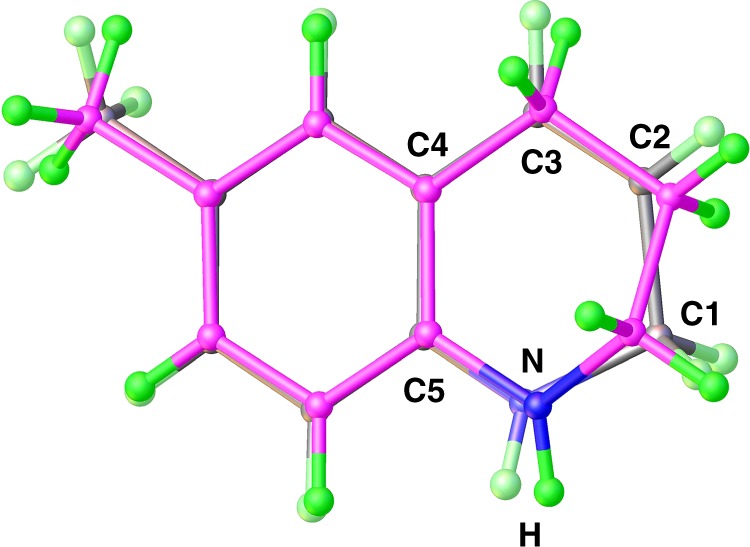
Superposition of 3u (gray) with 4u (magenta). Selected bond distances (Å) for **4u**: N1-C1, 1.452(3); C1-C2, 1.514(3); C2-C3, 1.527(3); C5-N1, 1.398 (3).

DHQs are presumed to be key intermediates in the hydrogenation of quinolines to THQs^16^. Indeed, 1,2-DHQs can be smoothly further reduced to THQs by H_3_N∙BH_3_ in the presence of the cobalt catalyst. For example, the 6-methyldihydroquinoline (**3u**) was completely converted to the tetrahydrogenated product (**4u**) under the catalytic conditions. Use of two equiv of H_3_N∙BH_3_ under the identical conditions of partial reduction allowed the transfer hydrogenation of **2u** to **4u** with an excellent yield. To compare the differences in bond distances and angles between dihydrogenated and tetrahydrogenated quinolines, the structures of **3u** and **4u** were superimposed as shown in Fig. 3. Comparing **3u** with **4u** reveals significant differences in the heterocyclic ring of the compounds. As a result of the second reduction the C2-C3 distance increases from 1.330(3) Å to 1.527(3) Å, and the N-heterocyclic ring becomes puckered.

### Synthetic applications

**Fig. 4:**
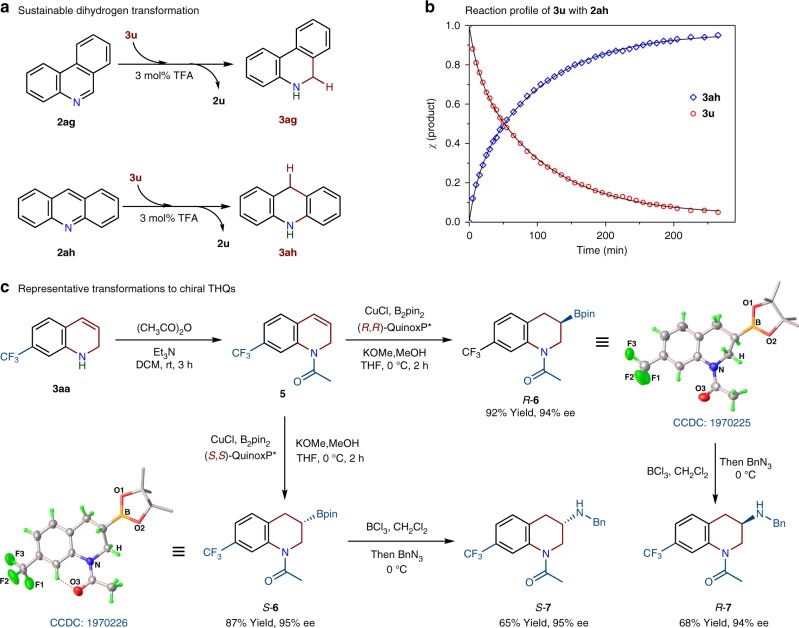
Synthetic Applications. **a 3u** serving as reducing agent for dihydrogen transfer. **b** Reaction profile of transfer hydrogenation of acridine (**2ah**, 0.32 M) to 9,10-dihydroacridine (**3ah**) by **3u** (0.32 M) catalyzed by CF_3_COOH (3 mol%) in THF-*d*_8_ at 25 ^o^C. **c** Asymmetric functionalization of **3aa** through enantioselective borylation and amination.

As a partially saturated heteroaromatic compound, the 1,2-dihydroquinoline system could act as both hydride acceptor and hydride donor, resembling the oxidized and reduced nicotinamide adenine dinucleotide respectively. We found 1,2-DHQs can be employed as dihydrogen sources for sustainable transformations. For example, phenanthridine (**2ag**) is reduced to 5,6-dihydrophenanthridine (**3ag**) quantitatively by **3u** in the presence of 3 mol% of CF_3_COOH (TFA), and acridine (**2ah**) is hydrogenated to 9,10-dihydroacridine (**3ah**) by **3u** under the identical reaction conditions (Fig. 4a). Disproportionation of **3u** was not observed during the acid-catalyzed H-transfer reaction. The reaction profile monitored by ^1^H NMR spectroscopy clearly showed that the increase in the concentration of **3ah** over time is consistent with the consumption of **3u** (Fig. 4b). Interestingly, **3u** is stable toward 2, 6-dimethylpyridine-3, 5-dicarboxylate. These results demonstrate that **3u** is a mild organo-hydride reagent, and its hydride-donating ability (Δ*H*_H-_) is weaker than that of the Hantszch ester (Δ*H*_H_^−^ = 69.3 kcal/mol)^67^.

1,2-DHQs are important synthetic intermediates that can lead to versatile *N*-heterocyclic compounds, such as chiral THQs and *N*-functionalized DHQs, which are common in pharmaceuticals and natural products^68^. For instance, through acylation and enantioselective borylation^69^, 6-trifluoromethyl-dihydroquinoline (**3aa**) was conveniently transformed to enantioenriched 3-boryl-tetrahydroquinolines *R*-**6** (92% yield, 94% ee) and *S*-**6** (87% yield, 95% ee), respectively (Fig. 4c). Such chiral *N*-heterocyclic organoboron compounds are amenable to diverse stereospecific functionalization at the stereogenic C-B bond^70,71^. For example, subsequent amination of the two enantiomers allows for the construction of a new C-N bond^72^, affording *R*-**7** and *S*-**7** compounds without loss of enantiomeric purity (94% ee). In particular, enantiomer *S*-**7** is a structural analog of positive inotropic agent (*S*)-903, and the potential agent Sumanirole for the treatment of Parkinson’s disease^73^.

## Discussion

**Fig. 5:**
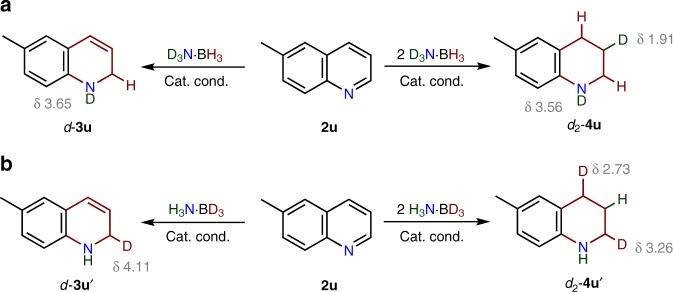
Deuterium labeling experiments. **a** Reactions of **2u** with D_3_N·BH_3_. **b** Reactions of **2u** with H_3_N·BD_3_.

To probe the mechanism of quioline reduction, the regio-specificity of hydrogen transfer with the selectively deuterated ammonia borane derivatives D_3_N∙BH_3_ and H_3_N∙BD_3_ was investigated for **2u** (Fig. 5). Tracing the regioselectivity of the deuterium addition to **2u** with D_3_N∙BH_3_, deuterium incorporation at the 1-position, the N atom in the desired product (*d*-**3u**) was found (see Supplementary Fig. 2). In the case of H_3_N∙BD_3_ as the dihydrogen source, ^2^H NMR spectroscopic analysis established that *d*-**3u′** was D-labeled entirely at the 2-position. Consecutive regioselective transfer hydrogenation of **2u** with two equiv of D_3_N∙BH_3_ afforded the bis-deuterated quinoline (*d*_2_-**4u**) deuterated at the 1- and 3-positions (Supplementary Fig. 4). In comparison with *d*_2_-**4u**, the reaction of **2u** with 2 equiv of H_3_N∙BD_3_ resulted in formation of *d*_2_-**4u′** which has deuterium exclusively at the 2- and 4-positions.

**Fig. 6:**
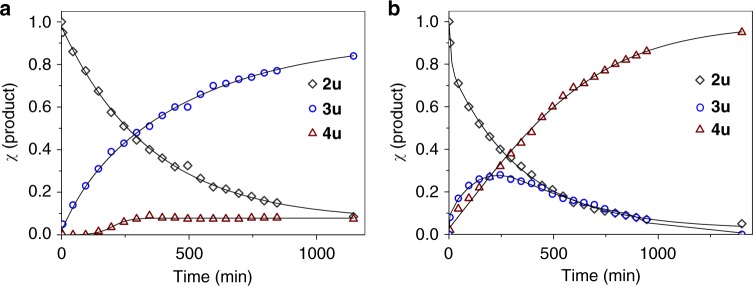
Reaction profiles. **a** Catalytic transfer hydrogenations of 6-methylquinoline (**2u**) with one equiv of H_3_N·BH_3_. **b 2u** with two equiv of H_3_N·BH_3_. Conditions: [**2u**] = 0.32 M, [**1**] = 0.0016 M, [H_3_N·BH_3_] = 0.35 M for (**a**) and 0.70 M for (**b**) in THF-*d*_8_ at 25 ^o^C.

To understand the relationship between the dihydrogenation and tetrahydrogenation catalysis performed by **1**, we followed the kinetics of the reactions of 6-methylquinoline (**2u**) with one or two equiv of H_3_N∙BH_3_. The changes in concentrations of reaction components over time are shown in Fig. 6. Using one equiv of H_3_N∙BH_3_ as the hydrogen source, the reaction profile indicates that the dihydroproduct (**3u**) alone was produced in the initial period of transfer hydrogenation (Fig. 6a). Although 7% of tetrahydrogenated product (**4u**) was observed, the major product of the reaction was the dihydrogenated compound (**3u**). In contrast, when starting with two equiv of H_3_N∙BH_3_, both **3u** and **4u** were produced in the initial stage of the catalysis but the reaction profile unambiguously reveals the intermediacy of **3u** in the tetrahydrogenation reaction (Fig. 6b).

The kinetic order in each reaction component was established for the transformation of **2u** to **3u** at 25 ^o^C. For reactions carried out at constant [**2u**], i.e. [H_3_N∙BH_3_] = 0.32 M, the initial rate (*v*_i_) for the product formation against the concentration of catalyst varied between 0.8 and 4.8 mM and showed first-order kinetics in [**1**], leading to a rate constant, *k*_obs_ = 4 M min^−1^ (Supplementary Fig. 5). At constant [H_3_N∙BH_3_] = 0.32 M and [**1**] = 1.6 mM, performing the dihydrogenation reaction with concentrations of **2u** ranging from 0.16 to 0.64 M led to a linear increase in *v*_i_ (Supplementary Fig. 6), which leads to a first-order reaction rate constant of 3.7 M min^−1^. This value is consistent with *k*_obs_ deduced from the plot of *v*_i_ ~ [**1**]. No inhibition was observed for *v*_i_ at high initial concentrations of the substrate. Since **2u** can be easily reduced to **4u** at high concentrations of H_3_N∙BH_3_, the kinetic experiments were carried out at constant [**2u**] = 0.32 M and [**1**] = 1.6 mM by varying [H_3_N∙BH_3_] in the range of 0.08–0.32 M. A linear plot of *v*_i_ ~ [H_3_N∙BH_3_] established a first-order dependence on the dihydrogen source at this stage (Supplementary Fig. 7). These results indicate that all the three components are involved in the turnover-limiting formation of **3u**. The overall rate law of this dihydrogenated reaction is thus expressed by *rate* = *k*_obs_[**1**][**2u**][H_3_N∙BH_3_].

Deuterium kinetic isotope effects (DKIE) were further probed for the dihydrogenation reaction (Supplementary Fig. 8). According to the rate of formation of **3u** measured for H_3_N∙BH_3_ versus H_3_N∙BD_3_, a KIE of *k*_NH·BH_/*k*_NH·BD_ = 1.55 was calculated. A larger KIE with *k*_NH·BH_/*k*_ND·BH_ = 3.63 was found for **2u** with D_3_N∙BH_3_. For the double DKIE reaction of D_3_N∙BD_3_ with **2u**, the KIE value was determined to be 5.73 (*k*_NH·BH_/*k*_ND·BD_). These results indicate that hydrogen transfers clearly participate in the turnover-determining step.

**Fig. 7:**
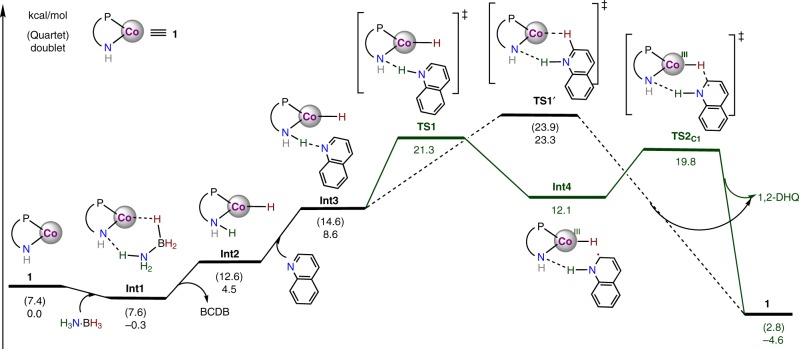
Gibbs energy diagram. For computational details, see Supplementary Information.

Density functional calculations^74^ were performed to explore the detailed mechanism and regioselectivity of the reaction. As a 17-electron neutral compound, the catalyst (**1**) favors the low spin doublet, which is 7.4 kcal/mol lower in energy than the quartet state (Fig. 7). These calculations suggest that the catalysis is initiated by the reaction of **1** with H_3_N∙BH_3_ through an isoenergetic complex intermediate (**Int1**), in which a hydrogen bond N^H^---H-NH_2_ is formed by the interaction of the amide group with the NH_3_ moiety. A concerted proton transfer/hydride transfer^75–77^ from H_3_N∙BH_3_ to the amido-cobalt unit in **Int1** yields a cobalt(II)-hydride species (**Int2)** by release of H_2_N-BH_2_. This step was calculated to be endergonic by 4.8 kcal/mol in the doublet state. Binding of the substrate to **Int2** forming **Int3**, was predicted to proceed via H-bonding between the N atom of the quinoline and the H atom of amino group of the ligand.

From **Int3**, four different mechanistic pathways have been located, and the two with lower barriers are presented here. The first of these involves proton transfer from the amino group to the quinoline nitrogen (**TS1**) coupled with an electron transfer from the Co^II^ center to the quinoline moiety. **TS1** is a doublet whose barrier was calculated to be 12.7 kcal/mol relative to **Int3**. The resultant Co^III^-H species with an N-hydrogenated quinoline radical (**Int4**) is +3.5 kcal/mol higher in energy than **Int3**. Due to the directing effect of the hydrogen bond, C_8_H_7_N---H-N^H^ between the substrate and the amino group of the ligand, the hydrogen atom transfer from the metal center to the *ortho*-position of the substrate via **TS2**_**C1**_ (1,2-hydrogenation pathway, 19.8 kcal/mol) was found to be preferred by 2.3 kcal/mol over the H-transfer to the *para*-position (**TS2**_**C3**_, 1,4-hydrogenation pathway, 22.1 kcal/mol, Supplementary Fig. 99). **TS1** was calculated to be the turnover-determining transition state (TDTS) with a total barrier of 21.6 kcal/mol relative to **Int1** (the turnover determining intermediate or TDI)^77^. The calculated barrier is also in reasonable agreement with the experimental rate constant of 4 min^−1^, which can be converted by classical transition state theory to an energy barrier of 19.0 kcal/mol. Importantly, **TS2** is the regioselectivity determining step and the regioselectivity is kinetically controlled. As a consequence, 1,2-DHQ is the major product, with a calculated product ratio 1,2-/1,4-DHQ of 48.6:1.

Such a stepwise H^+^-e^−^/H∙ pathway can only take place in the doublet state, while in the quartet state the hydrogen transfers proceed via a concerted H^+^/H^–^ pathway (**TS1’**). The concerted transfer of the hydridic Co(III)-H and the protic N^H^-H to the C=N bond in the doublet state is energetically slightly favorable (+0.6 kcal/mol) over the quartet state. Related to the stepwise pathway, the total barrier of the concerted pathway is 2.0 kcal/mol higher. For both pathways, the ammonia proton is transferred to the quinoline nitrogen and the hydride of the BH_3_ unit is delivered to the 2-position of quinoline. This agrees well with the results obtained from deuterium isotope labeling experiments (Fig. 5). Other mechanisms through different H_3_N∙BH_3_ activation modes have also been considered (Supplementary Figs. 100–108), but are associated with much higher barriers.

DFT predictions imply a stepwise H^+^-e^−^/H∙ pathway for the partial transfer hydrogenation. To gain more evidence, we calculated the kinetic isotope effects of deuterated ammonia boranes on the transition states of **TS1**, **TS2**_**C1**_, and **TS1’** (Table 1). The calculated DKIE effects for **TS1** using H_3_N∙BD_3_ or D_3_N∙BH_3_ are 3.57 and 1.67, respectively. Since **TS1** involves a proton-coupled electron transfer process, it is reasonable that a larger KIE should come from deuterated D_3_N∙BH_3_ with respect to H_3_N∙BD_3_. The DKIE effect observed for H_3_N∙BD_3_ is dominated by an equilibrium isotope effect, resulting from the formation of a Co-D bond in **Int2** when compared with the B-D bond in **Int1**. For the double DKIE reaction of D_3_N∙BD_3_, the total KIE was predicted to be 6.01. Compared with the transition state of **TS1**, **TS2**_**C1**_ was found to exhibit a much larger DKIE of 3.21 using H_3_N∙BD_3_ and only an equilibrium isotope effect (1.36) for D_3_N∙BH_3._ These DKIEs arise from transferring a deuterium atom from Co-D to C1, and the formation of N-D versus N-H bonds in **TS2**_**C1**_ and **Int1**. In contrast, the DKIE increases to 6.09 when D_3_N∙BH_3_ is used in the concerted pathway involving **TS1’** while an inverse KIE of 0.50 was calculated for H_3_N∙BD_3_. The calculated DKIE values for **TS2**_**C1**_ and **TS1’** are inconsistent with the experimental results.

**Table 1:** Computational and experimental kinetic isotope effects.

KIE	H_3_N·BD_3_	D_3_N·BH_3_	D_3_N·BD_3_
Cal.(TS1)	1.67	3.57	6.01
Cal.(TS2_C1_)	3.21	1.36	4.35
Cal.(TS1’)	0.50	6.09	3.06
Exp.	1.55	3.63	5.73

**Fig. 8:**
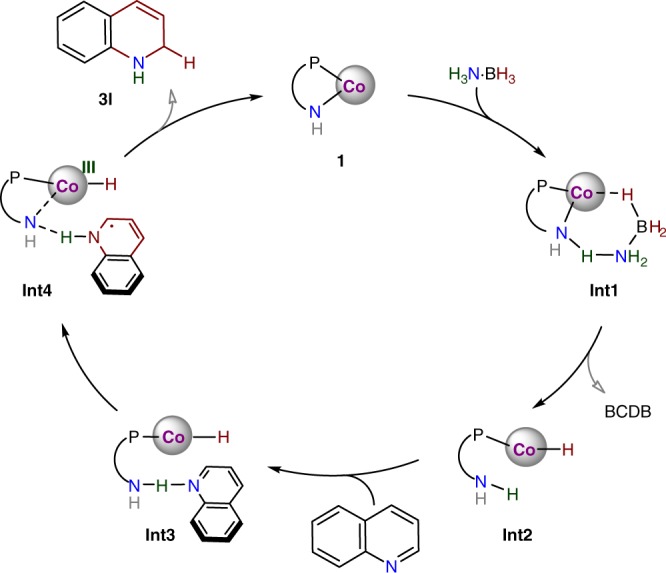
Proposed Mechanism. Catalytic cycle for partial transfer hydrogenation of quinoline.

The calculated DKIE values for **TS1** are consistent with the experimental values (1.55 for H_3_N∙BD_3_, 3.63 for D_3_N∙BH_3_, and 5.73 for D_3_N∙BD_3_), and this validates the suggested reaction pathway via **TS1** as the TDTS. Accordingly, a proposed mechanism of the catalytic partial transfer hydrogenation of quinoline is shown in Fig. 8. Complemented by the basic site, the cobalt-amido complex (Co-N^H^) is able to activate H_3_N∙BH_3_, generating a hydride-proton species (HCo-N^H^(H), **Int2**) for hydrogen transfers^75–77^. Combining the experimental data with theoretical studies, a stepwise H^+^-e^−^/H∙ mechanism is proposed for the subsequent reactions. Transfer of the proton from the amino group of the ligand to the N atom of quinoline, induced by the H-bonding C_8_H_7_N---H-N^H^ (**Int3**) and accompanied by an electron transfer from the metal center, generates **Int4**. Through the H-bonding interaction with the N atom of the substrate, the amido site not only assists the proton transfer from the NH_3_ moiety to the N atom but also directs the hydrogen atom transfer from Co(III)-H to the 2-position to furnish 1,2-regioselective reduction.

**Fig. 9:**
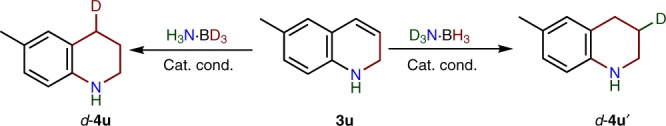
Deuterium labeling. Reductions of **3u** with deuterated ammonia borane derivatives.

Based on the deuterium labeling studies, we propose that the 1,2-DHQ product is the intermediate in the transfer hydrogenation of quinolines to THQs^16,36,38^. The conversion of 1,2-DHQ to 1,4-DHQ was not observed under the catalytic conditions, and we speculate that the partially saturated product is further reduced by the “hydride-proton” species through **Int2**, which is able to transfer the hydride of Co(II)-H to the 4-position, and the proton of the amino ligand to the C-3 position^20,22^. This hypothesis is further supported by the transfer hydrogenation of **3u** with the selectively deuterated ammonia boranes (Fig. 9, Supplementary Fig. 3). For the reaction of **3u** with H_3_N∙BD_3_, the ^2^H NMR spectrum exhibits only a characteristic ^2^H signal at δ 2.73 for *d*-**4u**, indicating deuterium labeling at the 4-position. In the case of D_3_N∙BH_3_, deuterium incorporation was found only at the 3-position in the desired product, *d*-**4u**′. These results also suggest that reversible dehydrogenation of **3u** or **4u** does not occur in the transfer hydrogenations^18,20^.

We have developed a highly efficient transfer hydrogenation system to convert quinolines to 1,2-DHQs. Through cobalt-amido cooperation, the *N*-heteroarene system smoothly undergoes 1,2-reduction by H_3_N∙BH_3_ which serves as a proton/hydride source. The reaction is conveniently controlled by the use of equimolar amounts of reducing agent at room temperature. This catalysis exhibits broad functional group compatibility and enables the large-scale synthesis of 1,2-DHQs. Experimental and theoretical studies reveal that the catalysis invokes a key intermediate, HCo-N^H^(H) for the hydrogen transfers. The reactive nature of such a “hydride-proton” intermediate is reflected by the dehydrogenation of H_3_N∙BH_3_, catalyzed by the cobalt-amino complex to release H_2_ at room temperature. Through H-bonding with the substrate, the amido site is essential in assisting proton transfer and thus directs the 1,2-hydrogenation.

## Methods

### Complex 1

*n*-BuLi (2.5 mol/L) (0.29 mL, 0.72 mmol) was added to a solution of 1,2-Ph_2_P(*p*-CH_3_C_6_H_4_)NH_2_ (210 mg, 0.72 mmol) in THF (20 mL) at 0 °C. After stirring for 3 h, the solution was allowed to warm to room temperature, and then [Cp*CoCl]_2_ (165 mg, 0.36 mmol) was added. The color of the mixture immediately turned from yellow to rose-Bengal. The solvent was removed under vacuum, and the residue was extracted with hexane (3 × 10 mL). After recrystallization from hexane at −30 °C, compound **1** was obtained as red microcrystals. Yield: 282 mg (81%). HRMS (m/z): [M]^+^ calcd for C_29_H_32_CoNP, 484.1604; found, 484.1610; analysis (calcd., found for C_29_H_32_CoNP): C(71.89, 71.68), H (6.66, 6.52), N (2.89, 2.97).

### Partial transfer hydrogenation

In a N_2_-filled glovebox, a scintillation vial (with a magnetic stir bar) was charged with quinoline (0.5 mmol), and H_3_N·BH_3_ (0.55 mmol, 17 mg). Then, catalyst **1** (1.2 mg, 2.5 µmol) and THF (2 ml) were added. The mixture was stirred at 25 ^o^C. After the indicated time, the product was isolated by chromatography on silica gel eluting with EtOAc/petroleum ether.

## Supplementary information

Supplementary Information

Description of Additional Supplementary Files

Supplementary Data 1

## Data Availability

The X-ray crystallographic coordinates for structures reported in this study have been deposited at the Cambridge Crystallographic Data Centre (CCDC), under deposition numbers 1935438-1935441, 1935443, 1970225 and 1970226. These data can be obtained free of charge from The Cambridge Crystallographic Data Centre via www.ccdc.cam.ac.uk/data_request/cif. All other data supporting the findings of this study are available within the article and Supplementary Information.
