# Supplementary material for: Controlled partial transfer hydrogenation of quinolines by cobalt-amido cooperative catalysis
Source: Nat Commun. 2020 Mar 6;11:1249. doi: 10.1038/s41467-020-15118-x (PMC7060234; doi:10.1038/s41467-020-15118-x)
Supplement: Supplementary file 1 — Supplementary Information [file 41467_2020_15118_MOESM1_ESM.pdf]

# Supplementary Information

# Controlled Partial Transfer Hydrogenation of Quinolines by Cobalt-Amido Cooperative Catalysis

**Pang et al**

## Supplementary Methods

All manipulations were done under a $N_2$ atmosphere using standard Schlenk line techniques or in a glovebox, unless otherwise stated. All reagents were purchased from Sigma-Aldrich, TCI or Acros and used without further purification, $D_3N{\cdot}BH_3$, $H_3N{\cdot}BD_3$, $D_3N{\cdot}BD_3$ were synthesized according to literature procedures. $Et_2O$, pentane, hexane, toluene, benzene and THF, were purified using a Glass Contour solvent purification system consisting of a neutral alumina, copper catalyst, and activated molecular sieves, then passed through an in-line, 2 μm filter immediately before being dispensed. $CDCl_3$, THF-$d_8$ were dried over $CaH_2$ and purified by vacuum transfer. 2-(diphenylphosphino)-4-methylaniline[1] and $[Cp^*CoCl]_2$[2] were prepared according to published methods.

NMR spectra were recorded on Bruker Avance 500 spectrometer in J. Young NMR tubes at room temperature. $^{1}H$ and $^{13}C$ NMR chemical shifts are referenced to the proton signal of the deuterated solvent. $^{11}B$ NMR is referenced to the signal of $BF_3{\cdot}OEt_2$. Infrared spectra were recorded on a Perkin Elmer FT-IR Spectrometer Spectrum Two. GC-MS spectra were obtained on a SHIMADZU GCMS-QP2010 SE spectrometer. MS (HRMS) measured with ThermoFisher Q-Exactive Mass Spectrometer. Elemental analyses (C, N, and H) were performed on Elementar Vario EL III analyzer, and samples were handled under $N_2$ atmosphere wherever appropriate.

Single crystals with appropriate dimensions were chosen under an optical microscope and quickly coated with high vacuum grease (Dow Corning Corporation) to prevent decomposition. Intensity data and cell parameters were recorded at 173 K on a Bruker Apex II single crystal diffractometer. Crystal data collection and refinement parameters are summarized in Supplementary Tables 2-8.

## Synthesis of 1

*n*-BuLi (2.5 mol/L) (0.29 mL, 0.72 mmol) was added to a solution of 1,2-$Ph_2P$(*p*-$CH_3$)$C_6H_4NH_2$ (210 mg, 0.72 mmol) in THF (20 mL) at 0 ℃. After stirring for 3 h, the solution was allowed to warm to room temperature, and then [Cp*CoCl]$_2$ (165 mg, 0.36 mmol) was added. The color of the mixture immediately turned from yellow to rose-Bengal. The solvent was removed under vacuum and the residue was extracted with hexane (3×10 mL). After recrystallization from hexane at -30 ℃, compound **1** was obtained as red microcrystals. Yield: 282 mg (81%). ESI-MS: calcd for [**1**]$^+$, 484.1604; found, 484.1610. Anal. Calcd. for $C_{29}H_{32}CoNP$: C, 71.89; H, 6.66; N, 2.89. Found: C, 71.68; H, 6.52; N, 2.97.

## Catalytic Dehydrogenation of $H_3N{\cdot}BH_3$

Under argon, 2 mL of a THF solution of $H_3N{\cdot}BH_3$ (25mg, 0.8 mmol) was placed into a 25 mL flask with a rubber plug. The flask was immersed in a 25 ℃ bath and, after the temperature of the solvent was reached, 1 mL of a THF solution of **1** (2mg, 0.004 mmol) was syringed. Then, the flask was sealed with paraffin and the volume of the evolved hydrogen was monitored by GC with methane as the internal standard (Supplementary Fig. 1). A white solid was formed during the reaction. The $^{11}B$ spectrum confirmed the presence of B-(cyclodiborazanyl)amine-borane (BCDB) (Supplementary Fig. 11). The assignment of the signals was agreed with the data reported by Berke et al.[1]

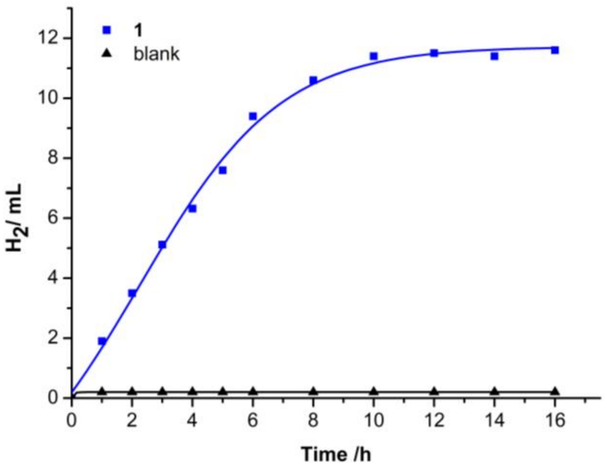


**Supplementary Figure 1. $H_2$ gas evolution in the catalytic dehydrogenation of $H_3N{\cdot}BH_3$ with 1 in THF at 298 K.**

## Optimization Studies

In a glovebox under an $N_2$ atmosphere, a scintillation vial (with a magnetic stir bar) was charged with 72 mg of 4-methylquinoline (**2a,** 0.5 mmol), and 17 mg of $H_3N·BH_3$ (0.55 mmol). The catalyst (0.5-5 mol%) and corresponding solvent (1 ml) were added. The mixture was stirred at the desired temperature (25-50 °C). After the indicated time, the yield was calculated by $^1$H NMR using 1,3,5-trimethoxybenzene as internal standard.

**Supplementary Table 1**. **Optimization of reaction conditions for the hydrogenation of 2a**.

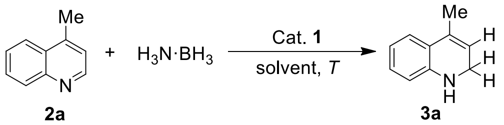


**2a** **3a**

| Entry | catalyst (loading) | solvent | *T* (°C) | time (h) | yield (%)[a] |
|---|---|---|---|---|---|
| 1 | none | toluene | 25 | 4 | 0 |
| 2 | **1** (5 mol%) | toluene | 50 | 4 | 76 |
| 3 | **1** (5 mol%) | toluene | 25 | 4 | 36 |
| 4 | **1** (5 mol%) | THF | 25 | 3 | >99 |
| 5 | **1** (2 mol%) | THF | 25 | 10 | >99 |
| 6 | **1** (0.5 mol%) | THF | 25 | 20 | >99 |
| 7 | **1** (0.5 mol%) | MeOH | 25 | 20 | 91 |

Reaction conditions: **2a** (0.5 mmol), $H_3N·BH_3$ (0.55 mol). [a]Determined by $^1$H NMR spectroscopy using 1,3,5-trimethoxybenzene as an internal standard.

## General Procedure

**General Procedure for the Semi-Hydrogenation of Quinoline.** In a glovebox under an $N_2$ atmosphere, a scintillation vial (with a magnetic stir bar) was charged with quinoline (0.5 mmol), and $H_3N·BH_3$ (0.55, 17 mg). The catalyst (1.2 mg, 2.5 μmol) and THF (2 ml) were added. The mixture was stirred at 25 °C. After the indicated time, the reaction mixture was isolated by chromatography on silica gel eluting with EtOAc/petroleum ether to give the product.

**General Procedure for the Full-Hydrogenation of Quinoline.** In a glovebox under an $N_2$ atmosphere, a scintillation vial (with a magnetic stir bar) was charged with quinoline (0.5 mmol), and $H_3N·BH_3$ (1.1mmol, 34 mg). The catalyst (1.2 mg, 2.5 μmol) and THF (2 mL) were added. The mixture was stirred at 25 °C. After the indicated time, the reaction mixture was isolated by chromatography on silica gel eluting with EtOAc/petroleum ether to give the product.

**Scale Up Reaction.** In a glovebox under an $N_2$ atmosphere, a round-bottom flask (with a magnetic stir bar) was charged with 4-methylquinoline (3.5 mmol, 500 mg), and $H_3N·BH_3$ (3.85 mmol, 120 mg). The catalyst (8.5 mg, 17.5 μmol) and THF (20 mL) were added. The mixture was stirred at 25 °C. After the indicated time, the reaction mixture was isolated through crystallization to give the product 4-methyldihydroquinoline (426 mg, yield: 84%).

**General Procedure for synthesis of 1-(7-(trifluoromethyl)quinolin-1(2H)-yl)ethanone.** A round -bottom flask (with a magnetic stir bar) was charged with 7-(trifluoromethyl)-1,2-dihydroquinolin e (0.5 mmol, 100 mg) and DCM (5mL). $Et_3N$ (0.8 mmol, 81 mg) and acetic anhydride (0.6 m mol, 61 mg) were added. The mixture was stirred at 25 °C for 4 h. Upon completion of the r eaction, the reaction was purified by chromatography on silica gel using to give the correspondi ng product.

**General Procedure of Cu-Catalyzed Enantioselective Hydroboration of 1-(7-(trifluoromethyl) quinolin-1(2H)-yl)ethanone.** In a glovebox under an $N_2$ atmosphere, CuCl (0.025 mmol), (*R*,*R*)-QuinoxP* or (*S*,*S*)-QuinoxP* (0.025 mmol), $B_2pin_2$ (0.6 mmol), and KOMe (0.1 mmol) were pl aced in an oven-dried Schlenk reaction vial, which was sealed with a rubber plug. THF (1.5 m L) was added to the Schlenk vial through the rubber plug. After 1-(7-(trifluoromethyl)quinolin-1 (2H)-yl)ethanone (0.50 mmol) was added to the mixture at 0 ℃, MeOH (1.0 mmol) was added dropwise. Upon completion of the reaction, the reaction mixture was purified by chromatograp hy on silica gel using to give the corresponding borylation product. The ee values were determi ned by HPLC analysis on a chiral stationary phase.

**Procedure for the synthesis of chiral amine 7 through the amination with $BnN_3$.** In an oven-dried vial, DCM solution of $BCl_3$ (1.0 M, 2.5 mL, 2.5 mmol) was added under nitrogen atmosphere. The DCM solution (4.0 mL) of **6** was added to the vial with stirring at room temperature. After 4 h, the volatile materials were removed under reduced pressure, and dry DCM (4.0 mL) was added to the resultant product. The reaction vial was cooled to 0 ℃, and benzylazide (199.7 mg, 1.5 mmol) was added to the mixture. After stirred for 16 h at 0 ℃, the reaction mixture was quenched by adding NaOH aq. (2.0 M), extracted three times with EtOAc, dried over $Na_2SO4$, and filtered. The crude material was then purified by flash column chromatography. The ee values were determined by HPLC analysis on a chiral stationary phase.

## Deuterium Labelling Experiments

In a glovebox under an $N_2$ atmosphere, a scintillation vial (with a magnetic stir bar) was charged with 0.32 mmol of 6-methylquinoline or 6-methyl-1,2-dihydroquinoline in THF (1 mL), a stoichiometric amount of $D_3N{\cdot}BH_3$ (or $H_3N{\cdot}BD_3$) and 0.5 mol% **1**. The vial was capped and the mixture was stirred until the reaction was complete. The solvent was removed under vacuum and $CHCl_3$ was then added and subsequently analyzed through $^2$H NMR spectroscopy.

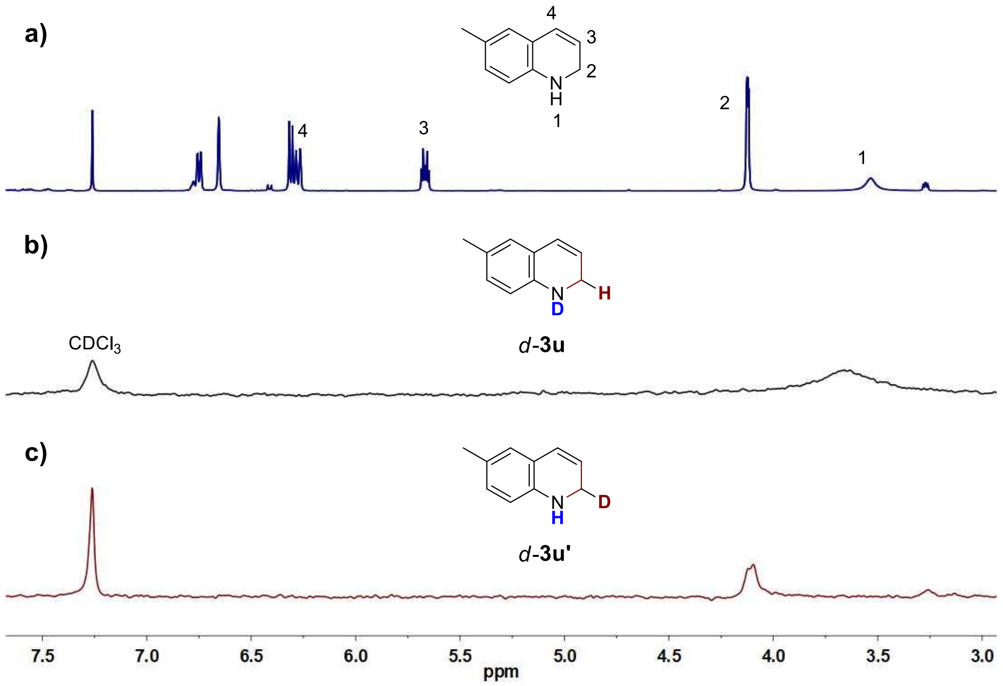


**Supplementary Figure 2. Deuterium Labelling Experiments of 2u with one equiv $D_3N{\cdot}BH_3$ (or $H_3N{\cdot}BD_3$).** a) $^1$H NMR spectrum (500 MHz, $CDCl_3$) of **3u**; b) $^2$H NMR spectrum for the reaction of 6-methylquinoline and $D_3N{\cdot}BH_3$; c) $^2$H NMR spectrum for the reaction of 6-methylquinoline and $H_3N{\cdot}BD_3$.

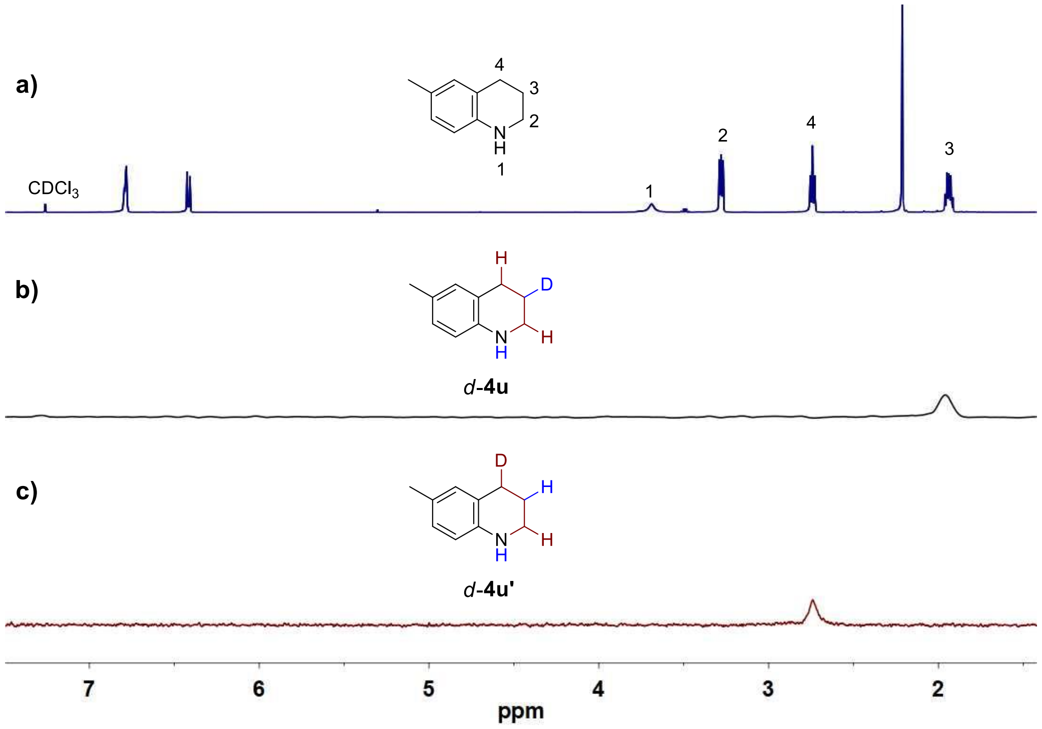


**Supplementary Figure 3. Deuterium Labelling Experiments of 3u with one equiv $D_3N{\cdot}BH_3$ (or $H_3N{\cdot}BD_3$).** a) $^1$H NMR spectrum (500 MHz, $CDCl_3$) of **4u**; b) $^2$H NMR spectrum for the reaction of 6-methyldihydroquinoline and $D_3N{\cdot}BH_3$; c) $^2$H NMR spectrum for the reaction of 6-methyldihydroquinoline and $H_3N{\cdot}BD_3$.

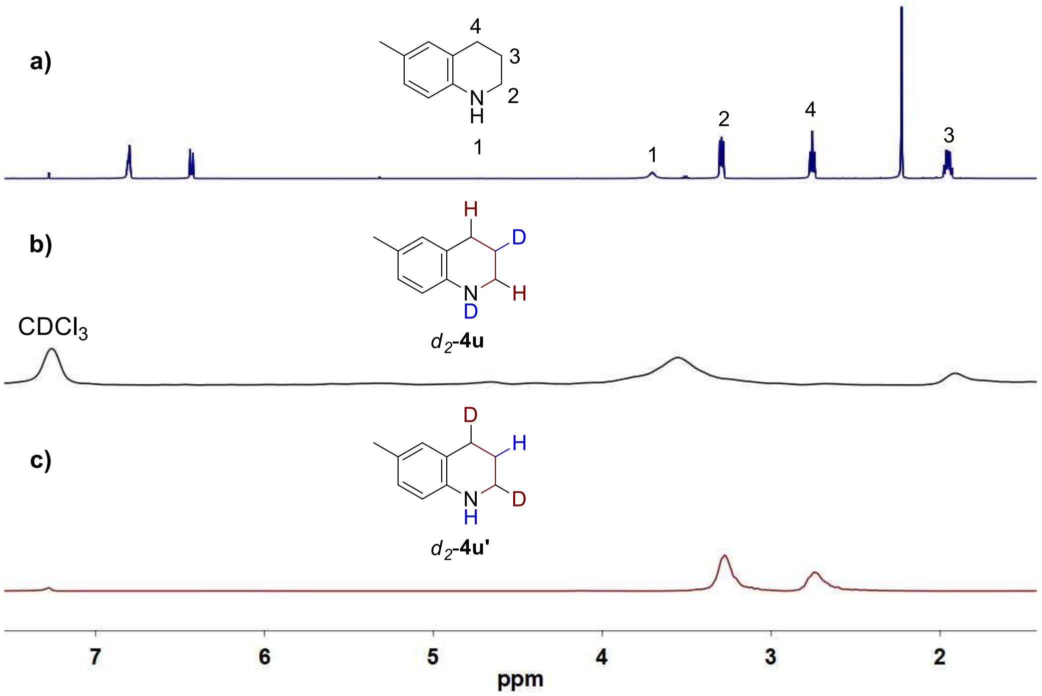


**Supplementary Figure 4. Deuterium Labelling Experiments of 2u with two equiv $D_3N{\cdot}BH_3$ (or $H_3N{\cdot}BD_3$).** a) $^1$H NMR spectrum (500 MHz, $CDCl_3$) of **4u**; b) $^2$H NMR spectrum for the reaction of 6-methylquinoline and two equiv $D_3N{\cdot}BH_3$; c) $^2$H NMR spectrum for the reaction of 6-methylquinoline

and two equiv $H_3N{\cdot}BD_3$.

## Kinetic Experiments

Varying concentrations of **1** while keeping a constant concentrations of both $H_3N{\cdot}BH_3$ and **2u**. The reaction was monitored by $^1$H NMR (500 MHz, THF-$d_8$) analysis at 600 s intervals over 6 h at 298.1 K. The kinetic data were obtained from intensity increase in the C$H_2$ integral of **3u** over time (up to 30% conversion) relative to 1,3,5-trimethoxybenzene (internal standard) to determine the initial reaction rate. Data were fit by least-squares analysis ($R^2 > 0.98$).

| $C_{H3N{\cdot}BH3}$ (M) | $C_{\mathbf{2u}}$ (M) | $C_{\mathbf{1}}$ (M) | $v_i$ (M/min) | $R^2$ |
|---|---|---|---|---|
| 0.32 | 0.32 | 0.0008 | $(3.21 \pm 0.26) \times 10^{-4}$ | 0.98237 |
| 0.32 | 0.32 | 0.0016 | $(6.42 \pm 0.18) \times 10^{-4}$ | 0.9951 |
| 0.32 | 0.32 | 0.0032 | $(1.22 \pm 0.30) \times 10^{-3}$ | 0.98766 |
| 0.32 | 0.32 | 0.0048 | $(1.86 \pm 0.12) \times 10^{-3}$ | 0.98161 |

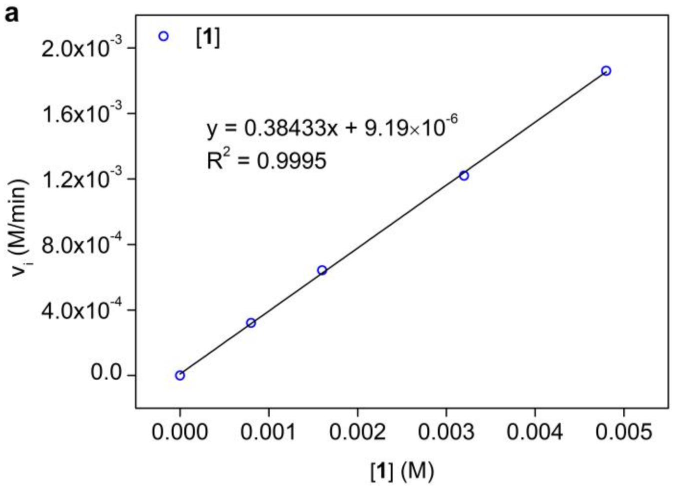


**Supplementary Figure 5. Plot of [1] *vs*. reaction rate.** The reaction follows first order dependence on **1** over the probed concentration range.

Varying concentrations of **2u** while keeping a constant concentrations of both $H_3N \cdot BH_3$ and **1**. The reaction was monitored by $^1$H NMR (500 MHz, THF-$d_8$) analysis at 600 s intervals over 6 h at 298.1 K. The kinetic data were obtained from intensity increase in the C$H_2$ integral of **3u** over time (up to 30% conversion) relative to 1,3,5-trimethoxybenzene (internal standard) to determine the initial reaction rate. Data were fit by least-squares analysis ($R^2 > 0.98$).

| $C_{H3N \cdot BH3}$ (M) | $C_{\mathbf{2u}}$ (M) | $C_{\mathbf{1}}$ (M) | $v_i$ (M/min) | $R^2$ |
|---|---|---|---|---|
| 0.32 | 0.16 | 0.0016 | $(3.24 \pm 0.32) \times 10^{-4}$ | 0.99631 |
| 0.32 | 0.32 | 0.0016 | $(6.42 \pm 0.18) \times 10^{-4}$ | 0.9951 |
| 0.32 | 0.48 | 0.0016 | $(9.94 \pm 0.06) \times 10^{-4}$ | 0.98243 |
| 0.32 | 0.64 | 0.0016 | $(1.31 \pm 0.15) \times 10^{-3}$ | 0.98963 |

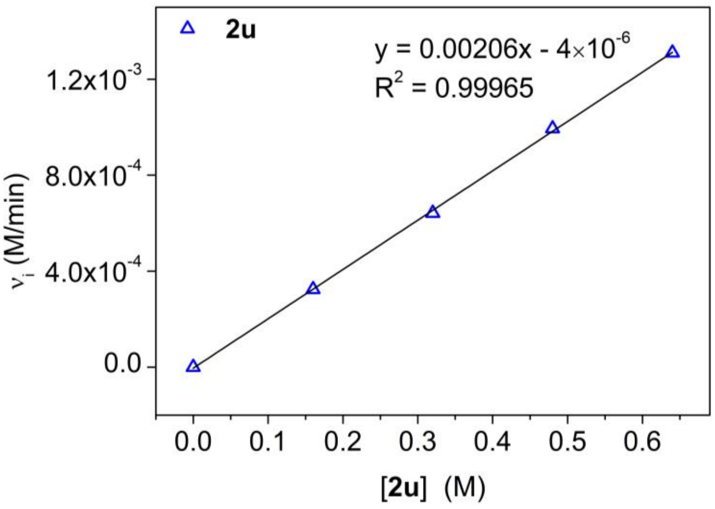


**Supplementary Figure 6. Plot of [2u] *vs*. reaction rate.** The reaction follows first order dependence on **2u** over the probed concentration range.

Varying concentrations of $H_3N\cdot BH_3$ while keeping a constant concentrations of both **2u** and **1**. The reaction was monitored by $^1$H NMR (500 MHz, THF-$d_8$) analysis at 600 s intervals over 6 h at 298.1 K. The kinetic data were obtained from intensity increase in the C$H_2$ integral of **3u** over time (up to 30% conversion) relative to 1,3,5-trimethoxybenzene (internal standard) to determine the initial reaction rate. Data were fit by least-squares analysis ($R^2 > 0.98$).

| $C_{H3N\cdot BH3}$ (M) | $C_{\mathbf{2u}}$ (M) | $C_{\mathbf{1}}$ (M) | $v_i$ (M/min) | $R^2$ |
|---|---|---|---|---|
| 0.08 | 0.32 | 0.0016 | $(1.89 \pm 0.14) \times 10^{-4}$ | 0.98769 |
| 0.16 | 0.32 | 0.0016 | $(2.94 \pm 0.07) \times 10^{-4}$ | 0.99145 |
| 0.24 | 0.32 | 0.0016 | $(4.99 \pm 0.24) \times 10^{-4}$ | 0.98836 |
| 0.32 | 0.32 | 0.0016 | $(6.42 \pm 0.18) \times 10^{-4}$ | 0.9951 |

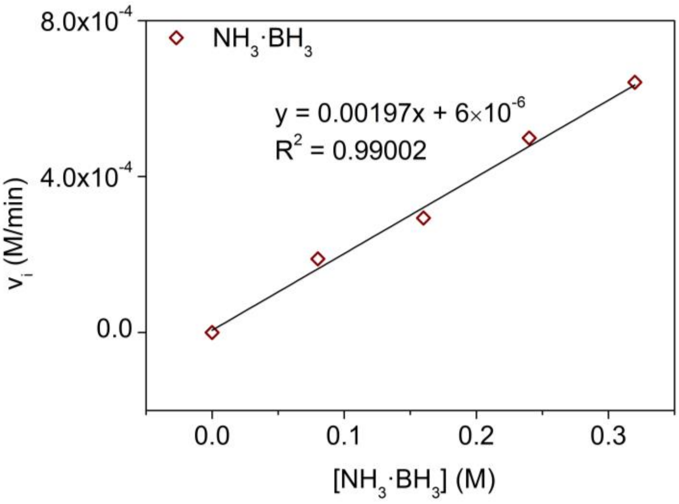


**Supplementary Figure 7. Plot of [$H_3N\cdot BH_3$] *vs.* reaction rate**. The reaction follows first order dependence on $H_3N\cdot BH_3$ over the probed concentration range.

## Determination of the kinetic isotope effect

KIE was determined using standard condition, data points were collected at 600 s intervals over 5 h at 298.1 K, data points before 30% conversion were subjected to the linear regression analysis.

| **1** | **2u** | $H_3N\cdot BH_3$ | 1,3,5-trimethoxybenzene |
|---|---|---|---|
| 0.0016 mmol<br>0.0016 M | 0.32 mmol<br>0.32 M | 0.32 mmol<br>0.32 M | 0.10 mmol<br>0.10 M |

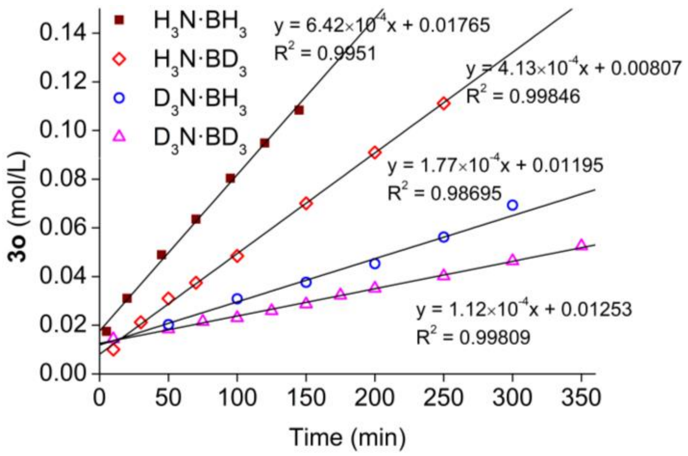


**Supplementary Figure 8. KIE for dihydrogenation of 2u.**

## $CF_3COOH$ catalyzed transfer hydrogenation of acridine

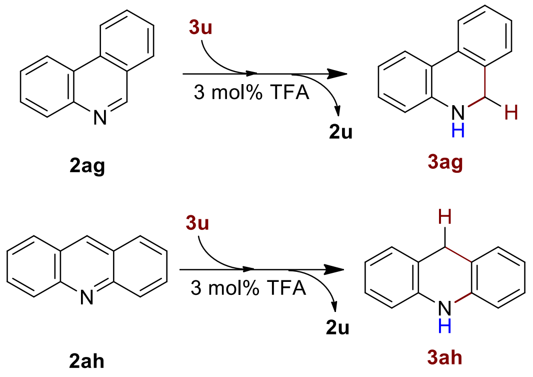


In a glovebox under an $N_2$ atmosphere, a J. YOUNG nmr tube was charged with 20 mg of 6-methyl-1,2-dihydroquinoline (**3u**) in THF-$d_8$ (0.6 mL), a stoichiometric amount of phenanthridine (**2ag**) or acridine (**2ah**) then with 3 mol% trifluoroacetic acid. The mixture was monitored by $^1$H NMR spectroscopy.

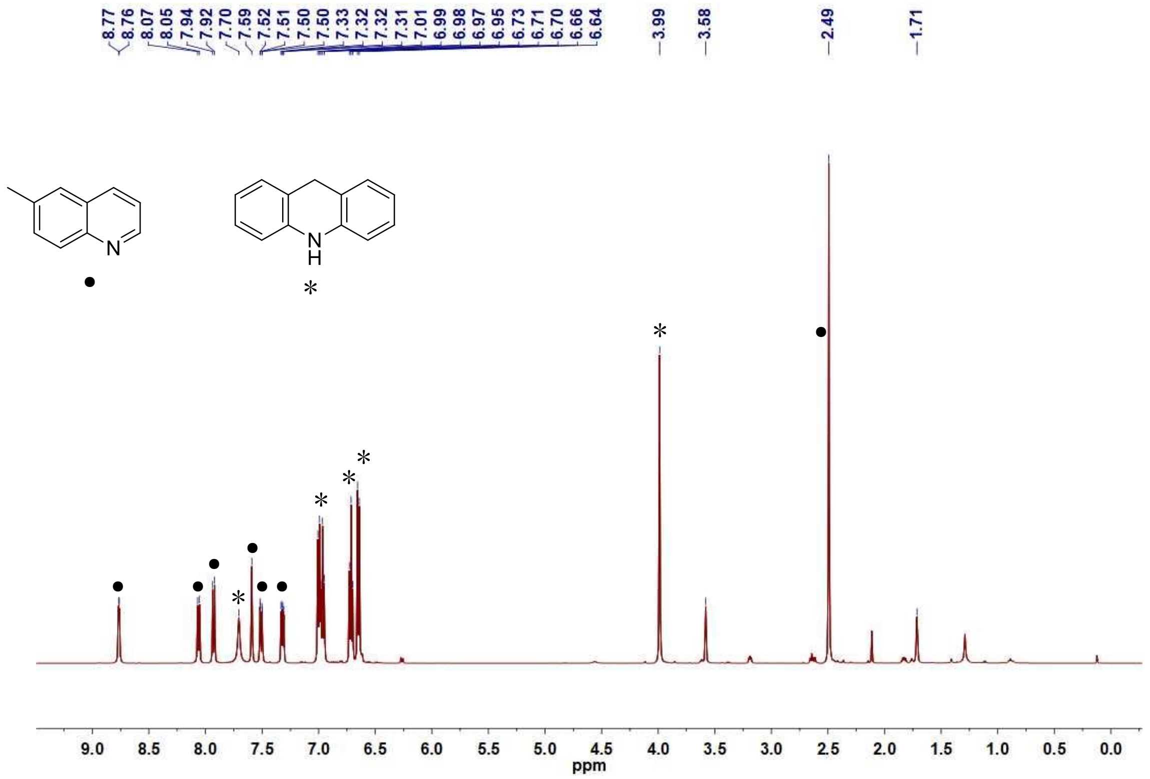


**Supplementary Figure 9. $^1$H NMR (500 MHz, THF-$d_8$) spectrum for the reaction of 3u with 2ah.** (* for **3ah**, • for **2u**)

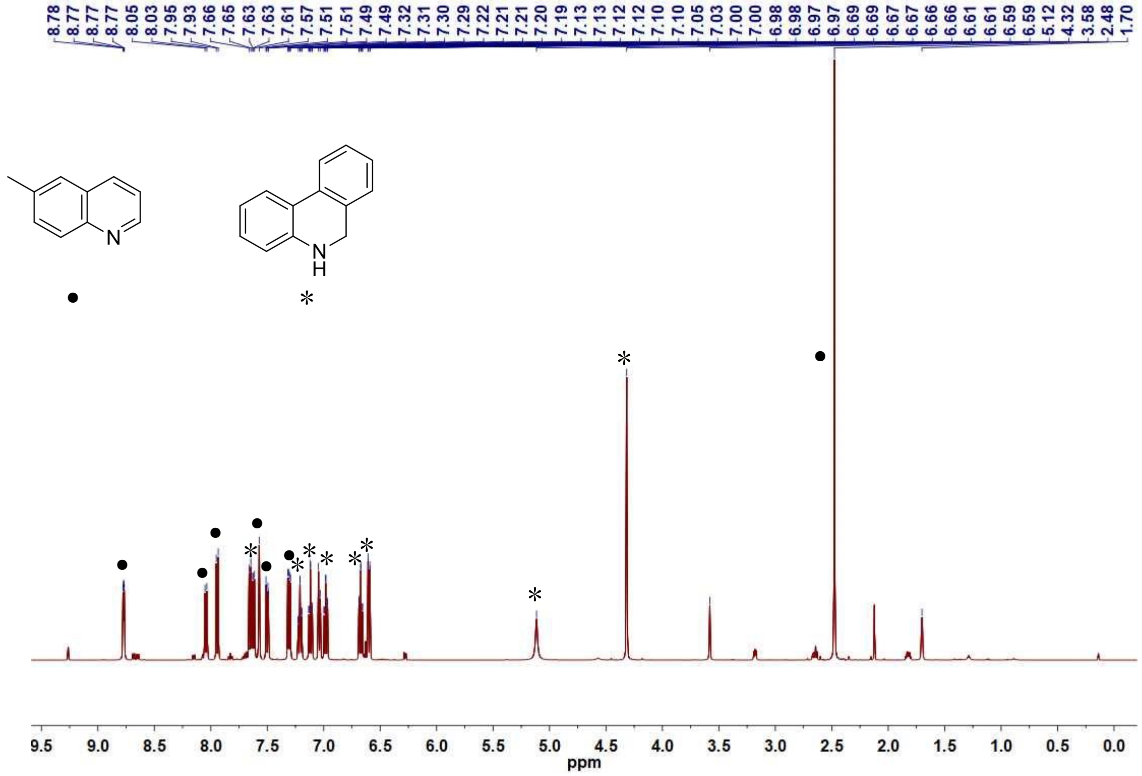


**Supplementary Figure 10. $^{1}$H NMR (500 MHz, THF-$d_8$) spectrum for the reaction of 3u with 2ag.** (* for **3ag**, • for **2u**)

## Characterization data for hydrogenation products

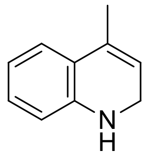

**4-methyl-1,2-dihydroquinoline (3a):**$^{1}$H NMR (500 MHz, $CDCl_3$) δ 7.03 (dd, *J* = 7.6, 1.3 Hz, 1H), 6.97 (td, *J* = 7.7, 1.4 Hz, 1H), 6.64 (td, *J* = 7.5, 1.1 Hz, 1H), 6.41 (dd, *J* = 7.8, 1.0 Hz, 1H), 5.51 (dt, *J* = 5.3, 2.8 Hz, 1H), 4.10 (td, *J* = 3.4, 1.6 Hz, 2H), 3.67 (br s, 1H), 1.98 (dd, *J* = 3.2, 1.6 Hz, 3H). $^{13}$C NMR (126 MHz, $CDCl_3$) δ 145.06 (s), 131.21 (s), 128.43 (s), 123.69 (s), 122.76 (s), 118.96 (s), 117.71 (s), 112.67 (s), 43.00 (s), 18.63 (s). HRMS-ESI (m/z): Calcd for $[M+H]^+$, 146.0970; found, 146.0974.

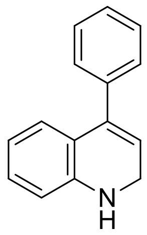

**4-phenyl-1,2-dihydroquinoline (3b):** $^{1}$H NMR (500 MHz, $CDCl_3$) δ 7.42–7.36 (m, 2H), 7.36–7.31 (m, 3H), 6.99 (td, *J* = 7.7, 1.5 Hz, 1H), 6.84 (dd, *J* = 7.7, 1.4 Hz, 1H), 6.57 (td, *J* = 7.5, 1.1 Hz, 1H), 6.51 (dd, *J* = 7.9, 1.0 Hz, 1H), 5.68 (t, *J* = 4.2 Hz, 1H), 4.22 (d, *J* = 4.2 Hz, 2H), 3.72 (br s, 1H). $^{13}$C NMR (126 MHz, $CDCl_3$) δ 145.55 (s), 139.69 (s), 138.47 (s), 128.82 (s), 128.69 (s), 128.22 (s), 127.33 (s), 126.14 (s), 122.36 (s), 120.90 (s), 117.90 (s), 113.34 (s), 43.07 (s). $^{1}$H and $^{13}$C NMR data agree with reported data[3].

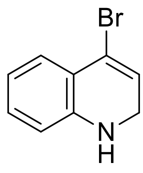

**4-bromo-1,2-dihydroquinoline (3c):** $^{1}$H NMR (500 MHz, $CDCl_3$) δ 7.28 (d, *J* = 7.7 Hz, 1H), 6.99 (td, *J* = 7.8, 1.2 Hz, 1H)., 6.66 (td, *J* = 7.6, 0.9 Hz, 1H), 6.36 (d, *J* = 7.9 Hz, 1H), 6.03 (t, *J* = 4.3 Hz, 1H), 4.19 (d, *J* = 4.3 Hz, 2H), 3.67 (br s, 1H). $^{13}$C NMR (126 MHz, $CDCl_3$) δ 144.22 (s), 129.00 (s), 126.43 (s), 122.59 (s), 118.87 (d, *J* = 9.0 Hz), 117.07 (s), 111.69 (s), 43.85 (s). HRMS-ESI (m/z): Calcd for $[M+H]^+$, 209.9918; found, 209.9922.

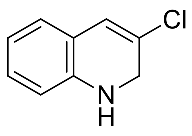

**3-chloro-1,2-dihydroquinoline (3d):** $^{1}$H NMR (500 MHz, CDCl3) δ 6.96 (td, *J* = 7.7, 1.5 Hz, 1H), 6.81 (dd, *J* = 7.4, 1.4 Hz, 1H), 6.61 (td, *J* = 7.4, 1.0 Hz, 1H), 6.44 (s, 1H), 6.41 (d, *J* = 7.9 Hz, 1H), 4.31 (d, *J* = 1.4 Hz, 2H), 3.76 (br s, 1H). $^{13}$C NMR (126 MHz, CDCl3) δ 142.26 (s), 128.86 (s), 126.54 (s), 125.88 (s), 124.04 (s), 119.92 (s), 118.19 (s), 112.51 (s), 48.83 (s). HRMS-ESI (m/z): Calcd for $[M+H]^+$, 165.0345; found, 165.0331.

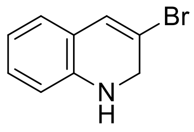

**3-bromo-1,2-dihydroquinoline (3e):** $^{1}$H NMR (500 MHz, $CDCl_3$) δ 6.98 (td, *J* = 7.8, 1.3 Hz, 1H), 6.80 (d, *J* = 6.6 Hz, 1H), 6.66 (s, 1H), 6.64–6.59 (td, 1H), 6.40 (d, *J* = 7.9 Hz, 1H), 4.39 (d, *J* = 1.4 Hz, 2H), 3.71 (s, 1H). $^{13}$C NMR (126 MHz, $CDCl_3$) δ 142.39 (s), 129.05 (s), 128.12 (s), 126.46 (s), 120.43 (s), 118.21 (s), 115.33 (s), 112.70 (s), 50.64 (s). HRMS-ESI (m/z): Calcd for $[M+H]^+$, 208.9840; found, 208.9835.

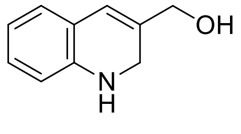

**(1,2-dihydroquinolin-3-yl)methanol (3f):** $^{1}$H NMR (500 MHz, $CDCl_3$) δ 6.95 (td, *J* = 7.7, 1.4 Hz, 1H), 6.85 (dd, *J* = 7.4, 1.2 Hz, 1H), 6.60 (td, *J* = 7.4, 0.9 Hz, 1H), 6.41 (d, *J* = 7.9 Hz, 1H), 6.28 (s, 1H), 4.12 (d, *J* = 4.0 Hz, 4H), 3.67 (br s, 1H). $^{13}$C NMR (126 MHz, $CDCl_3$) δ 143.05 (s), 132.35 (s), 127.65 (s), 125.95 (s), 120.94 (s), 119.83 (s), 116.97 (s), 111.48 (s), 63.82 (s), 42.87 (s). HRMS-ESI (m/z): Calcd for $[M+H]^+$, 162.0919; found, 162.0925.

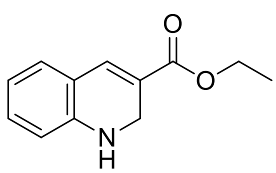

**ethyl 1,2-dihydroquinoline-3-carboxylate (3g):** $^{1}$H NMR (500 MHz, $CDCl_3$) δ 7.34 (s, 1H), 7.03 (td, 1H), 6.97 (dd, *J* = 7.5, 1.4 Hz, 1H), 6.59 (td, *J* = 7.4, 1.0 Hz, 1H), 6.41 (d, *J* = 8.0 Hz, 1H), 4.35 (d, *J* = 1.4 Hz, 2H), 4.25 (q, *J* = 7.1 Hz, 2H), 3.86 (br s, 1H), 1.33 (t, *J* = 7.1 Hz, 3H). $^{13}$C NMR (126 MHz, $CDCl_3$) δ

165.74 (s), 146.25 (s), 135.60 (s), 131.52 (s), 129.53 (s), 121.76 (s), 119.25 (s), 117.93 (s), 112.92 (s), 60.56 (s), 42.02 (s), 14.34 (s). HRMS-ESI (m/z): Calcd for $[M+H]^+$, 204.1025; found, 204.1021.

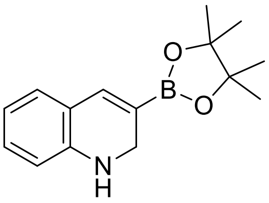

**3-(4,4,5,5-tetramethyl-1,3,2-dioxaborolan-2-yl)-1,2-dihydroquinoline (3h):** $^{1}H$ NMR (500 MHz, $CDCl_3$) δ 7.00 (s, 1H), 6.96 (td, *J* = 7.7, 1.5 Hz, 1H), 6.88 (dd, *J* = 7.4, 1.4 Hz, 1H), 6.56 (td, *J* = 7.4, 1.1 Hz, 1H), 6.37 (d, *J* = 7.9 Hz, 1H), 4.18 (d, *J* = 1.7 Hz, 2H), 3.71 (br s, 1H), 1.29 (s, 12H). $^{13}C$ NMR (126 MHz, $CDCl_3$) δ 145.40 (s), 138.42 (s), 128.95 (s), 127.01 (s), 120.32 (s), 116.74 (s), 111.84 (s), 82.46 (s), 42.69 (s), 23.78 (s). HRMS-ESI (m/z): Calcd for $[M+H]^+$, 258.1665; found, 258.1659.

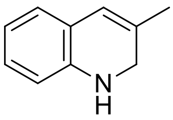

**3-methyl-1,2-dihydroquinoline (3i):** $^{1}H$ NMR (500 MHz, $CDCl_3$) δ 6.90 (t, *J* = 7.6 Hz, 1H), 6.78 (d, *J* = 7.2 Hz, 1H), 6.57 (t, *J* = 7.3 Hz, 1H), 6.37 (d, *J* = 7.8 Hz, 1H), 6.07 (s, 1H), 4.05 (s, 2H), 3.69 (br s, 1H), 1.76 (s, 3H). $^{13}C$ NMR (126 MHz, $CDCl_3$) δ 143.01 (s), 131.05 (s), 127.63 (s), 125.88 (s), 121.69 (s), 121.38 (s), 117.76 (s), 112.08 (s), 47.59 (s), 20.93 (s), 1.02 (s). $^{1}H$ and $^{13}C$ NMR data agree with reported data[4].

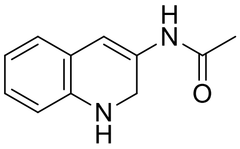

***N*-(1,2-dihydroquinolin-3-yl)acetamide (3j):** $^{1}H$ NMR (500 MHz, DMSO) δ 9.21 (s, 1H), 6.78 (s, 1H), 6.78–6.73 (td, 1H), 6.70 (d, *J* = 7.3 Hz, 1H), 6.41 (td, *J* = 7.4, 1.0 Hz, 1H), 6.37 (d, *J* = 7.8 Hz, 1H), 5.72 (s, 1H), 4.03 (s, 2H), 1.96 (s, 3H). $^{13}C$ NMR (126 MHz, DMSO) δ 169.07 (s), 143.63 (s), 130.84 (s), 126.90 (s), 126.07 (s), 121.77 (s), 117.10 (s), 112.15 (s), 107.92 (s), 44.21 (s), 24.34 (s). HRMS-ESI (m/z): Calcd for $[M+H]^+$, 189.1028; found, 189.1024.

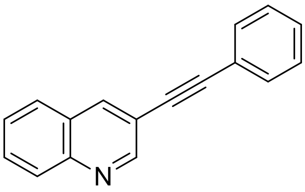

**3-(phenylethynyl)quinolone (2k):** $^{1}H$ NMR (500 MHz, $CDCl_3$) δ 9.01 (d, *J* = 2.1 Hz, 1H), 8.31 (d, *J* = 1.8 Hz, 1H), 8.11 (d, *J* = 8.4 Hz, 1H), 7.81 (d, *J* = 8.0 Hz, 1H), 7.73 (ddd, *J* = 8.4, 6.9, 1.4 Hz, 1H), 7.59 (ddd, *J* = 15.3, 6.1, 1.7 Hz, 3H), 7.42–7.36 (m, 3H). $^{13}C$ NMR (126 MHz, $CDCl_3$) δ 152.16 (s), 146.88 (s), 138.25 (s), 131.77 (s), 130.08 (s), 129.46 (s), 128.84 (s), 128.50 (s), 127.63 (s), 127.31 (s), 122.63 (s), 117.49 (s), 92.64 (s), 86.65 (s). $^{1}H$ and $^{13}C$ NMR data agree with reported data[5].

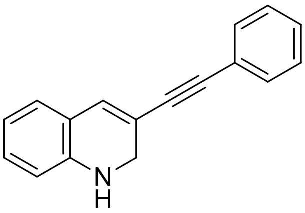

**3-(phenylethynyl)-1,2-dihydroquinoline (3k):** $^{1}H$ NMR (500 MHz, $CDCl_3$) δ 7.48–7.43 (m, 2H), 7.36–7.29 (m, 3H), 6.97 (td, *J* = 7.7, 1.4 Hz, 1H), 6.88 (dd, *J* = 7.4, 1.1 Hz, 1H), 6.70 (s, 1H), 6.61 (td, *J* = 7.4, 0.9 Hz, 1H), 6.42 (d, *J* = 7.9 Hz, 1H), 4.26 (d, *J* = 1.3 Hz, 2H), 3.77 (br s, 1H). $^{13}C$ NMR (126 MHz, $CDCl_3$) δ 144.24 (s), 131.89 (s), 131.52 (s), 129.49 (s), 128.36 (s), 128.23 (s), 127.39 (s), 123.22 (s), 120.98 (s), 118.19 (s), 114.45 (s), 112.83 (s), 91.63 (s), 88.68 (s), 45.98 (s). HRMS-ESI (m/z): Calcd for $[M+H]^+$, 232.1126; found, 232.1132.

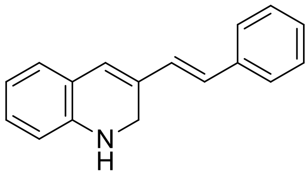

**(E)-3-styryl-1,2-dihydroquinoline (3l):** $^{1}$H NMR (500 MHz, $CDCl_3$) δ 7.44 (d, *J* = 7.6 Hz, 2H), 7.33 (t, *J* = 7.6 Hz, 2H), 7.23 (t, *J* = 7.3 Hz, 1H), 6.96 (t, *J* = 7.6 Hz, 1H), 6.93 (s, 1H), 6.90 (d, *J* = 8.1 Hz, 1H), 6.60 (t, *J* = 7.3 Hz, 1H), 6.47 (s, 1H), 6.44 (d, *J* = 7.8 Hz, 1H), 6.34 (d, *J* = 16.2 Hz, 1H), 4.44 (s, 2H), 3.94 (s, 1H). $^{13}$C NMR (126 MHz, $CDCl_3$) δ 144.29 (s), 137.39 (s), 130.22 (s), 128.89 (s), 128.70 (s), 128.41 (s), 127.46 (s), 127.35 (s), 127.05 (s), 126.70 (s), 126.35 (s), 120.98 (s), 117.80 (s), 112.22 (s), 43.08 (s). HRMS-ESI (m/z): Calcd for $[M+H]^+$, 234.1283; found, 234.1285.

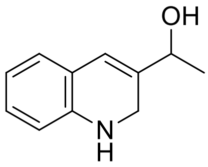

**1-(1,2-dihydroquinolin-3-yl)ethanol (3m):** $^{1}$H NMR (500 MHz, $CDCl_3$) δ 6.95 (td, *J* = 7.7, 1.5 Hz, 1H), 6.87 (dd, *J* = 7.4, 1.3 Hz, 1H), 6.60 (td, *J* = 7.4, 1.0 Hz, 1H), 6.43 (d, *J* = 7.8 Hz, 1H), 6.29 (s, 1H), 4.35 (q, *J* = 6.4 Hz, 1H), 4.12 (s, 2H), 1.34 (d, *J* = 6.5 Hz, 3H). $^{13}$C NMR (126 MHz, $CDCl_3$) δ 143.20 (s), 136.25 (s), 127.54 (s), 125.99 (s), 120.18 (s), 119.54 (s), 117.09 (s), 111.53 (s), 68.74 (s), 41.61 (s), 19.86 (s). HRMS-ESI (m/z): Calcd for $[M+H]^+$, 176.1075; found, 176.1082.

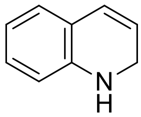

**1,2-dihydroquinoline (3o):** $^{1}$H NMR (500 MHz, $CDCl_3$) δ 6.93 (td, *J* = 7.7, 1.5 Hz, 1H), 6.81 (dd, *J* = 7.4, 1.3 Hz, 1H), 6.57 (td, *J* = 7.4, 1.0 Hz, 1H), 6.36 (d, *J* = 7.9 Hz, 1H), 6.29 (dt, *J* = 9.8, 1.7 Hz, 1H), 5.65 (dt, *J* = 9.8, 3.9 Hz, 1H), 4.19 (dd, *J* = 3.9, 1.9 Hz, 2H), 3.60 (br s, 1H). $^{13}$C NMR (126 MHz, $CDCl_3$) δ 144.86 (s), 128.65 (s), 126.80 (s), 126.34 (s), 122.00 (s), 121.09 (s), 117.83 (s), 112.61 (s), 43.18 (s).

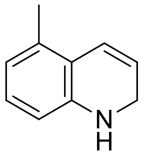

**5-methyl-1,2-dihydroquinoline (3p):** $^{1}$H NMR (500 MHz, $CDCl_3$) δ 6.84 (t, *J* = 7.7 Hz, 1H), 6.53 (dtd, *J* = 10.1, 1.9, 0.7 Hz, 1H), 6.45 (dt, *J* = 7.3 Hz, 1H), 6.27 (d, *J* = 7.9 Hz, 1H), 5.72 (dt, *J* = 10.0, 4.0 Hz, 1H), 4.11 (dd, *J* = 4.0, 1.9 Hz, 2H), 3.64 (br s, 1H), 2.23 (s, 3H). $^{13}$C NMR (126 MHz, $CDCl_3$) δ 145.03 (s), 134.08 (s), 128.12 (s), 123.25 (s), 121.82 (s), 119.91 (s), 119.50 (s), 111.05 (s), 42.60 (s), 18.53 (s). HRMS-ESI (m/z): Calcd for $[M+H]^+$, 146.0970; found, 146.0967.

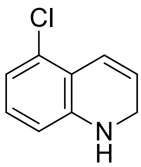

**5-chloro-1,2-dihydroquinoline (3q):** $^{1}$H NMR (500 MHz, $CDCl_3$) δ 6.81 (t, *J* = 8.0 Hz, 1H), 6.69 (dt, *J* = 10.1, 1.6 Hz, 1H), 6.59 (dd, *J* = 8.0, 0.8 Hz, 1H), 6.24 (dt, *J* = 8.0 Hz, 1H), 5.74 (dt, *J* = 10.0, 4.0 Hz, 1H), 4.17 (dd, *J* = 3.9, 1.9 Hz, 2H), 3.70 (br s, 1H). $^{13}$C NMR (126 MHz, $CDCl_3$) δ 146.20 (s), 131.67 (s), 128.88 (s), 123.12 (s), 122.47 (s), 118.33 (s), 111.16 (s), 42.78 (s). HRMS-ESI (m/z): Calcd for $[M+H]^+$, 166.0424; found, 166.0431.

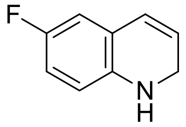

**6-fluoro-1,2-dihydroquinoline (3r):** $^{1}$H NMR (500 MHz, $CDCl_3$) δ 6.64 (td, *J* = 8.6, 2.9 Hz, 1H), 6.56 (dd, *J* = 8.9, 2.9 Hz, 1H), 6.29 (dd, *J* = 8.6, 4.6 Hz, 1H), 6.25 (dt, *J* = 9.8, 1.8 Hz, 1H), 5.68 (dt, 1H), 4.12 (dd, *J* = 4.0, 1.9 Hz, 2H), 3.54 (br s, 1H). $^{13}$C NMR (126 MHz, $CDCl_3$) δ 157.03 (s), 155.17 (s), 140.87 (s), 125.71 (d, *J* = 2.2 Hz), 123.78 (s), 122.26 (d, *J* = 7.7 Hz), 114.48 (d, *J* = 22.6 Hz), 113.09 (dd, *J* = 15.2, 7.6 Hz), 43.17 (s). $^{19}$F NMR (471 MHz, $CDCl_3$) δ -127.79 (s). HRMS-ESI (m/z): Calcd for $[M+H]^+$, 150.0719; found, 150.0713.

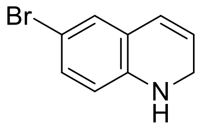

**6-bromo-1,2-dihydroquinoline (3s):** $^{1}$H NMR (500 MHz, $CDCl_3$) δ 6.98 (dd, *J* = 8.4, 2.3 Hz, 1H), 6.89 (d, *J* = 2.2 Hz, 1H), 6.22 (d, *J* = 8.3 Hz, 1H), 6.19 (dt, *J* = 1.7 Hz,

1H), 5.66 (dt, *J* = 9.8, 3.8 Hz, 1H), 4.20 (dd, *J* = 3.7, 1.9 Hz, 2H), 3.60 (br s, 1H). $^{13}$C NMR (126 MHz, $CDCl_3$) δ 143.73 (s), 130.98 (s), 129.13 (s), 125.29 (s), 123.13 (s), 122.74 (s), 113.93 (s), 109.02 (s), 43.17 (s). HRMS-ESI (m/z): Calcd for $[M+H]^+$, 209.9918; found, 209.9927.

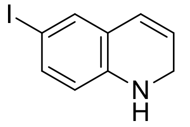

**6-iodo-1,2-dihydroquinoline (3t):** $^1$H NMR (500 MHz, $C_6D_6$) δ 6.95 (dd, *J* = 8.3, 2.0 Hz, 1H), 6.79 (d, *J* = 1.9 Hz, 1H), 5.81 (dt, *J* = 9.6, 1.5 Hz, 1H), 5.65 (d, *J* = 8.4 Hz, 1H), 5.17–5.05 (m, 1H), 3.55–3.43 (m, 2H), 2.43 (s, 1H). $^{13}$C NMR (126 MHz, $C_6D_6$) δ 143.00 (s), 130.10 (s), 128.35 (s), 124.36 (s), 121.60 (d, *J* = 6.8 Hz), 112.79 (s), 107.78 (s), 42.03 (s). HRMS-ESI (m/z): Calcd for $[M+H]^+$, 257.9780; found, 257.9782.

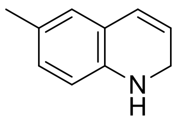

**6-methyl-1,2-dihydroquinoline (3u):** $^1$H NMR (500 MHz, $CDCl_3$) δ 6.75 (dd, *J* = 7.9, 1.5 Hz, 1H), 6.65 (d, *J* = 1.5 Hz, 1H), 6.31 (d, *J* = 7.9 Hz, 1H), 6.28 (dt, *J* = 9.8, 1.7 Hz, 1H), 5.67 (dt, *J* = 9.8, 4.0 Hz, 1H), 4.12 (dd, *J* = 4.0, 1.9 Hz, 2H), 3.53 (br s, 1H), 2.18 (s, 3H). $^{13}$C NMR (126 MHz, $CDCl_3$) δ 142.46 (s), 128.94 (s), 127.35 (s), 127.08 (s), 126.40 (s), 122.29 (s), 121.35 (s), 112.70 (s), 43.16 (s), 20.39 (s). HRMS-ESI (m/z): Calcd for $[M+H]^+$, 146.0970; found, 146.0972. $^1$H and $^{13}$C NMR data agree with reported data[6].

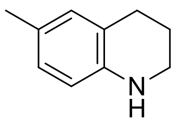

**6-methyl-1,2,3,4-tetrahydroquinoline** (**4u**): $^1$H NMR (500 MHz, $CDCl_3$) δ 6.79 (d, *J* = 5.7 Hz, 1H), 6.42 (d, *J* = 8.6 Hz, 1H), 3.69 (s, 1H), 3.33–3.22 (m, 2H), 2.74 (t, *J* = 6.4 Hz, 2H), 2.21 (s, 3H), 1.99–1.86 (m, 2H). $^{13}$C NMR (126 MHz, $CDCl_3$) δ 142.43 (s), 130.06 (s), 127.25 (s), 126.24 (s), 121.60 (s), 114.45 (s), 42.20 (s), 26.93 (s), 22.46 (s), 20.38 (s).

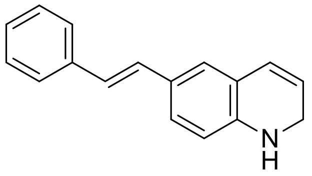

**(E)-6-styryl-1,2-dihydroquinoline (3v):** $^1$H NMR (500 MHz, $CDCl_3$) δ 7.46 (d, *J* = 7.3 Hz, 2H), 7.32 (t, *J* = 7.7 Hz, 2H), 7.20 (dd, *J* = 10.4, 4.2 Hz, 1H), 7.10 (dd, *J* = 8.1, 2.0 Hz, 1H), 7.01 (d, *J* = 1.9 Hz, 1H), 6.96 (d, *J* = 16.4 Hz, 1H), 6.86 (d, *J* = 16.3 Hz, 1H), 6.33 (t, *J* = 8.8 Hz, 2H), 5.67 (dt, *J* = 9.9, 3.9 Hz, 1H), 4.25 (dd, *J* = 3.8, 1.9 Hz, 2H), 3.75 (s, 1H). $^{13}$C NMR (126 MHz, $CDCl_3$) δ 144.67 (s), 138.09 (s), 128.75 (s), 128.60 (s), 127.48 (s), 126.73 (s), 126.18 (s), 126.02 (s), 124.87 (s), 124.29 (s), 122.24 (s), 112.57 (s), 43.37 (s). HRMS-ESI (m/z): Calcd for $[M+H]^+$, 234.1283; found, 234.1280.

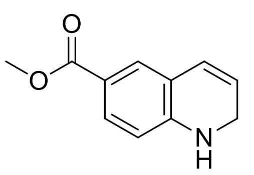

**methyl 1,2-dihydroquinoline-6-carboxylate (3w):** $^1$H NMR (500 MHz, $CDCl_3$) δ 6.75 (d, *J* = 7.8 Hz, 1H), 6.65 (s, 1H), 6.33–6.29 (m, 1H), 6.27 (d, *J* = 9.8 Hz, 1H), 5.66 (dt, *J* = 9.1, 3.6 Hz, 1H), 4.12 (dd, *J* = 2.3, 1.5 Hz, 2H), 3.52 (br s, 1H), 2.18 (s, 3H). $^{13}$C NMR (126 MHz, $CDCl_3$) δ 142.47 (s), 128.94 (s), 127.36 (s), 127.07 (s), 126.41 (s), 122.27 (s), 121.34 (s), 112.68 (s), 43.18 (s), 20.38 (s). HRMS-ESI (m/z): Calcd for $[M+H]^+$, 190.0868; found, 190.0861.

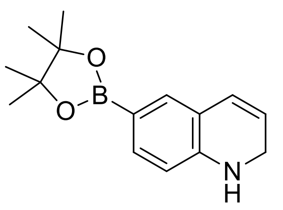

**6-(4,4,5,5-tetramethyl-1,3,2-dioxaborolan-2-yl)-1,2-dihydroquinoline (3x):** $^1$H NMR (500 MHz, $CDCl_3$) δ 7.38 (dd, *J* = 7.9, 1.3 Hz, 1H), 7.23 (d, *J* = 0.8 Hz, 1H), 6.29 (t, *J* = 9.8 Hz, 2H), 5.57 (dt, *J* = 9.8, 3.7 Hz, 1H), 4.26 (dd, *J* = 3.7, 2.0 Hz, 2H), 3.77 (br s, 1H), 1.31 (s, 12H). $^{13}$C NMR (126 MHz, $CDCl_3$) δ 147.64

(s), 136.04 (s), 133.56 (s), 126.24 (s), 121.12 (s), 119.71 (s), 111.69 (s), 83.22 (s), 43.34 (s), 24.89 (d, *J* = 13.3 Hz). HRMS-ESI (m/z): Calcd for $[M+H]^+$, 258.1665; found, 258.1659.

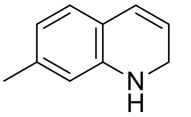

**7-methyl-1,2-dihydroquinoline (3y):** $^1$H NMR (500 MHz, $CDCl_3$) δ 6.71 (d, *J* = 7.5 Hz, 1H), 6.40 (dd, *J* = 7.5, 0.6 Hz, 1H), 6.28 (dt, 1H), 6.20 (s, 1H), 5.59 (dt, *J* = 9.8, 3.9 Hz, 1H), 4.16 (dd, *J* = 3.9, 1.9 Hz, 2H), 3.57 (br s, 1H), 2.20 (s, 3H). $^{13}$C NMR (126 MHz, $CDCl_3$) δ 144.79 (s), 138.62 (s), 126.67 (s), 126.17 (s), 120.87 (s), 118.63 (s), 113.36 (s), 43.14 (s), 21.44 (s). HRMS-ESI (m/z): Calcd for $[M+H]^+$, 146.0970; found, 146.0966.

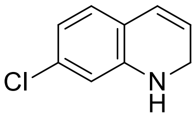

**7-chloro-1,2-dihydroquinoline (3z):** $^1$H NMR (500 MHz, $CDCl_3$) δ 6.69 (d, *J* = 7.9 Hz, 1H), 6.50 (dd, *J* = 7.9, 2.0 Hz, 1H), 6.32 (dd, 1H), 6.23 (dt, *J* = 9.9, 1.7 Hz, 1H), 5.62 (dt, *J* = 9.9, 3.9 Hz, 1H), 4.20 (dd, *J* = 3.8, 2.0 Hz, 2H), 3.65 (br s, 1H). $^{13}$C NMR (126 MHz, $CDCl_3$) δ 145.74 (s), 133.72 (s), 127.62 (s), 125.44 (s), 121.88 (s), 119.29 (s), 117.45 (s), 112.18 (s), 43.07 (s). HRMS-ESI (m/z): Calcd for $[M+H]^+$, 166.0424; found, 166.0427.

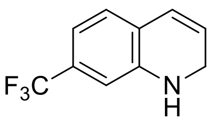

**7-(trifluoromethyl)-1,2-dihydroquinoline (3aa):** $^1$H NMR (500 MHz, $CDCl_3$) δ 6.84 (d, *J* = 7.7 Hz, 1H), 6.77 (dd, *J* = 7.7, 0.8 Hz, 1H), 6.52 (s, 1H), 6.28 (dt, *J* = 9.9, 1.8 Hz, 1H), 5.77–5.65 (dt, 1H), 4.26 (s, 2H), 3.73 (br s, 1H). $^{13}$C NMR (126 MHz, $CDCl_3$) δ 144.85 (s), 130.91–130.11 (t), 126.75 (s), 125.38 (s), 124.20 (s), 123.63 (s), 123.09 (s), 114.25 (q, *J* = 4.1 Hz), 108.71 (q, *J* = 3.9 Hz), 43.23 (s). $^{19}$F NMR (471 MHz, $CDCl_3$) δ -63.09 (s). HRMS-ESI (m/z): Calcd for $[M+H]^+$, 200.0687; found, 200.0693.

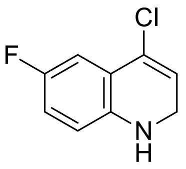

**4-chloro-6-fluoro-1,2-dihydroquinoline (3ab):** $^1$H NMR (500 MHz, $CDCl_3$) δ 7.07 (dd, *J* = 9.5, 2.9 Hz, 1H), 6.72 (td, *J* = 8.5, 2.9 Hz, 2H), 6.33 (dd, *J* = 8.6, 4.5 Hz, 2H), 5.86 (t, *J* = 4.3 Hz, 1H), 4.19 (d, *J* = 4.4 Hz, 4H), 3.64 (s, 2H). $^{13}$C NMR (126 MHz, $CDCl_3$) δ 156.96 (s), 155.09 (s), 141.41 (s), 128.54 (s), 120.66 (s), 116.14 (d, *J* = 22.9 Hz), 113.35 (d, *J* = 7.5 Hz), 111.74 (d, *J* = 25.4 Hz), 44.03 (s). HRMS-ESI (m/z): Calcd for $[M+H]^+$, 184.0329; found, 184.0326.

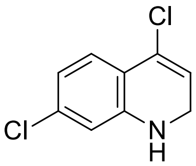

**4,7-dichloro-1,2-dihydroquinoline (3ac):** $^1$H NMR (500 MHz, $CDCl_3$) δ 7.20 (d, *J* = 8.2 Hz, 1H), 6.59 (dd, *J* = 8.2, 2.0 Hz, 1H), 6.35 (d, *J* = 2.0 Hz, 1H), 5.75 (t, *J* = 4.2 Hz, 1H), 4.25 (d, *J* = 4.2 Hz, 2H), 3.70 (br s, 1H). $^{13}$C NMR (126 MHz, $CDCl_3$) δ 145.09 (s), 134.34 (s), 132.11 (d, *J* = 9.6 Hz), 127.82 (d, *J* = 10.3 Hz), 127.58 (s), 125.03 (s), 118.00 (s), 116.61 (s), 116.41 (s), 111.14 (s), 43.01 (s). HRMS-ESI (m/z): Calcd for $[M+H]^+$, 200.0034; found, 200.0032.

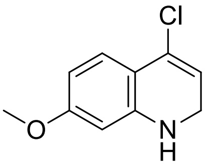

**4-chloro-7-methoxy-1,2-dihydroquinoline (3ad):** $^1$H NMR (500 MHz, $CDCl_3$) δ 7.22 (d, *J* = 8.5 Hz, 1H), 6.20 (dd, *J* = 8.5, 2.4 Hz, 1H), 5.95 (d, *J* = 2.4 Hz, 1H), 5.63 (t, *J* = 4.2 Hz, 1H), 4.19 (d, *J* = 4.2 Hz, 2H), 3.74 (s, 3H). $^{13}$C NMR (126 MHz, $CDCl_3$) δ 160.31 (s), 145.61 (s), 128.05 (s), 125.20 (s), 115.18 (s), 111.75 (s), 101.88 (s), 97.50 (s), 54.15 (s), 42.96 (s). HRMS-ESI (m/z): Calcd for $[M+H]^+$, 196.0529; found, 196.0523.

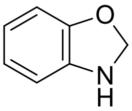

**2,3-dihydrobenzo[d]oxazole (3ae):** $^1$H NMR (500 MHz, $C_6D_6$) δ 6.90 (td, *J* = 7.7, 1.2 Hz, 1H), 6.64 (td, *J* = 7.6, 1.2 Hz, 1H), 6.52 (dd, *J* = 7.8, 1.2 Hz, 1H), 6.35 (d, *J* = 7.7 Hz, 1H), 4.08 (br s, 1H), 2.35 (s, 2H). $^{13}$C NMR (126 MHz, $C_6D_6$) δ 143.58 (s), 138.44 (s), 121.58 (s), 117.02 (s), 113.89 (s), 111.09 (s), 30.06 (s). $^1$H and $^{13}$C NMR data agree with reported data[7].

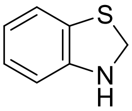

**2,3-dihydrobenzo[d]thiazole (3af):** $^1$H NMR (500 MHz, $CDCl_3$) δ 7.28 – 7.23 (m, 1H), 7.19 (dd, *J* = 7.6, 1.5 Hz, 1H), 6.58 (d, *J* = 8.2 Hz, 1H), 6.53 (td, *J* = 7.5, 1.1 Hz, 1H), 4.89 (br s, 1H), 2.78 (s, 2H). $^{13}$C NMR (126 MHz, $CDCl_3$) δ 147.24 (s), 128.89 (s), 125.27 (s), 122.14 (s), 121.50 (s), 111.78 (s), 53.10 (s). $^1$H NMR data agree with reported data[8].

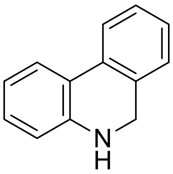

**5,6-dihydrophenanthridine (3ag):** $^1$H NMR (500 MHz, $CDCl_3$) δ 7.75–7.61 (m, 2H), 7.31 (t, *J* = 7.5 Hz, 1H), 7.22 (td, *J* = 7.4, 0.9 Hz, 1H), 7.15 – 7.04 (m, 1H), 6.85 (td, *J* = 7.7, 1.0 Hz, 1H), 6.67 (dd, *J* = 7.9, 0.7 Hz, 1H), 4.40 (s, 2H), 3.98 (br s, 1H). $^{13}$C NMR (126 MHz, $CDCl_3$) δ 144.68 (s), 131.71 (s), 131.04 (s), 127.78 (s), 126.63 (s), 126.10 (s), 124.98 (s), 122.56 (s), 121.39 (s), 121.04 (s), 118.25 (s), 114.09 (s), 45.35 (s). $^1$H and $^{13}$C NMR data agree with reported data[9].

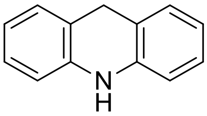

**9,10-dihydroacridine (3ah):** $^1$H NMR (500 MHz, $CDCl_3$) δ 7.09 (dd, *J* = 14.7, 7.4 Hz, 4H), 6.85 (td, *J* = 7.4, 1.1 Hz, 2H), 6.67 (d, *J* = 7.9 Hz, 2H), 5.94 (br s, 1H), 4.06 (s, 2H). $^{13}$C NMR (126 MHz, $CDCl_3$) δ 139.12 (s), 127.58 (s), 125.98 (s), 119.62 (s), 119.03 (s), 112.41 (s), 30.37 (s).

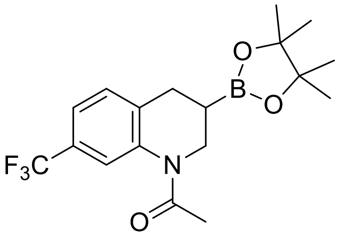


**1-(3-(4,4,5,5-tetramethyl-1,3,2-dioxaborolan-2-yl)-7-(trifluoromethyl)-3,4-dihydroquinolin-1(2H)-yl)ethanone (6):** $^1$H NMR (500 MHz, $CDCl_3$) δ 7.73 (s, 1H), 7.34–7.28 (m, 1H), 7.24 (s, 1H), 3.95 (s, 1H), 3.72 (dd, *J* = 12.6, 9.4 Hz, 1H), 2.91 (dd, *J* = 16.0, 5.3 Hz, 1H), 2.80 (d, *J* = 8.1 Hz, 1H), 2.30 (s, 3H), 1.61 (s, 1H), 1.22 (s, 12H). $^{13}$C NMR (126 MHz, $CDCl_3$) δ 170.05 (s), 139.00 (s), 128.84 (s), 128.34 (d, *J* = 32.0 Hz), 125.07 (s), 122.91 (s), 121.54 (s), 120.90 (s), 83.87 (s), 46.17 (s), 28.89 (s), 24.71 (d, *J* = 1.2 Hz), 23.54 (s). $^{19}$F NMR (471 MHz, $CDCl_3$) δ -62.36 (s). HRMS-ESI (m/z): Calcd for $[M+H]^+$, 370.1801; found, 370.1807. Daicel CHIRALPAK®, AS-H, *i*PrOH/Hexane = 10/90, 1 mL/min, 25 °C. (*R*)-isomer: $t_R$ = 10.745 min; (*S*)-isomer: $t_R$ = 8.325 min

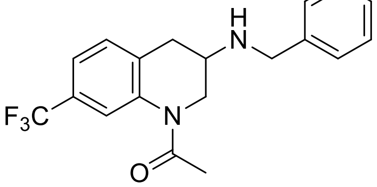


**1-(3-(benzylamino)-7-(trifluoromethyl)-3,4-dihydroquinolin-1(2H)-yl)ethanone (7):** $^1$H NMR (500 MHz, $CDCl_3$) δ 7.43 – 7.32 (m, 5H), 7.28 (s, 2H), 4.03 (s, 1H), 3.91 (q, *J* = 13.2 Hz, 2H), 3.65 (s, 1H), 3.28 – 3.18 (m, 1H), 3.12 (dd, *J* = 16.4, 5.5 Hz, 1H), 2.73 (dd, *J* = 16.4, 6.9 Hz, 1H), 2.29 (s, 3H). $^{13}$C NMR (126 MHz, $CDCl_3$) δ 169.04 (s), 138.84 (s), 128.75 (s), 127.55 (s), 127.07 (s), 126.24 (s), 120.58 (s), 120.16 (s), 51.28 (s), 50.29 (s), 33.55 (s), 28.68 (s), 21.98 (s). $^{19}$F NMR (471 MHz, $CDCl_3$) δ -62.45 (s).HRMS-ESI (m/z): Calcd for $[M+H]^+$, 349.1528; found, 349.1523. Daicel CHIRALPAK®, OD-H, *i*PrOH/Hexane =

10/90, 0.5 mL/min, 25 °C. (*R*)-isomer: $t_R$ = 42.448 min; (*S*)-isomer: $t_R$ = 46.115 min. $^1$H , $^{13}$C NMR and HPLC data agree with reported data[10].

## NMR and HPLC spectra

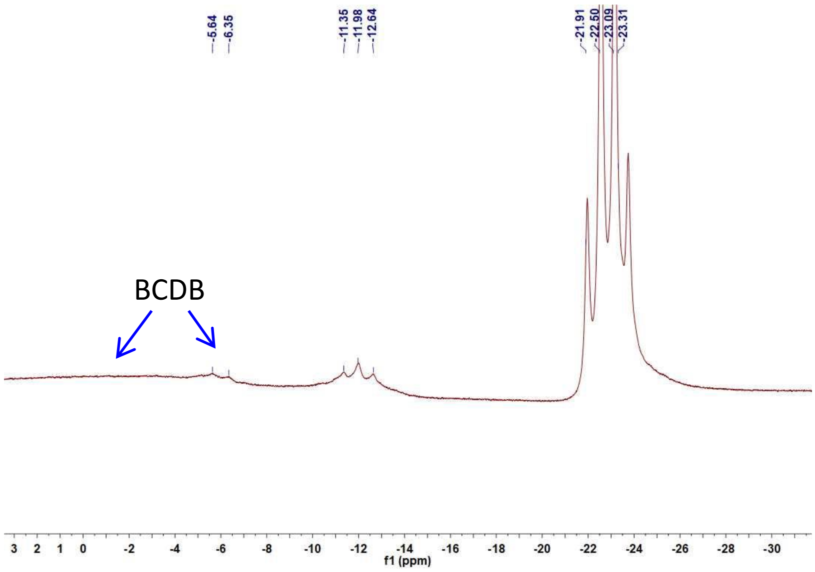


**Supplementary Figure 11. $^{11}B$ spectrum of the BCDB obtained from the catalytic dehydrogenation reaction.**

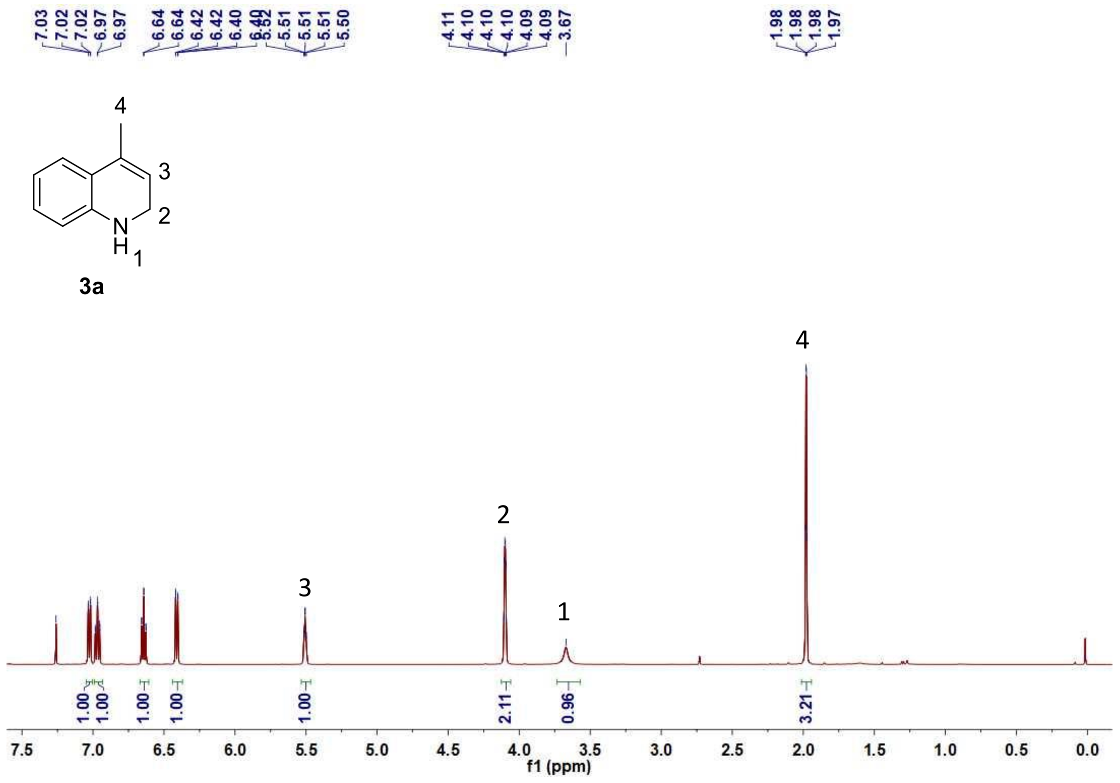


**Supplementary Figure 12.** **$^{1}$H NMR (500 MHz, $CDCl_3$) spectrum of 3a.**

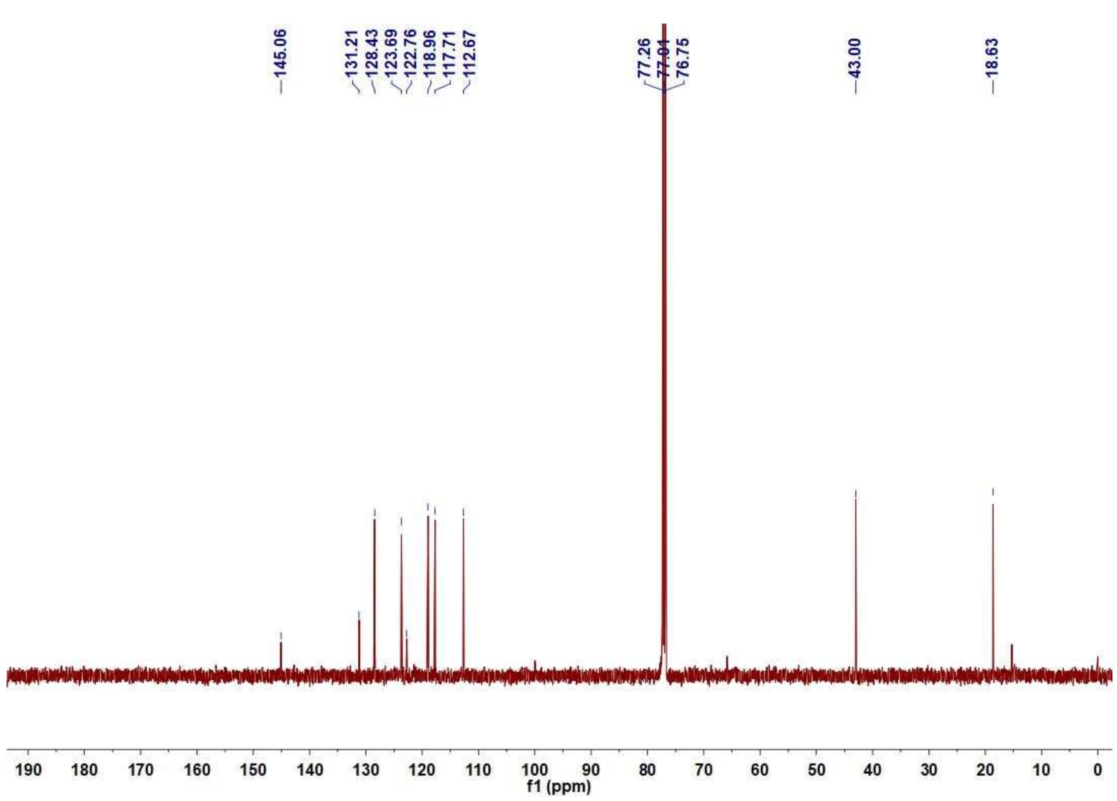


**Supplementary Figure 13.** $^{13}C$ **NMR (126 MHz,** $CDCl_3$**) spectrum of 3a.**

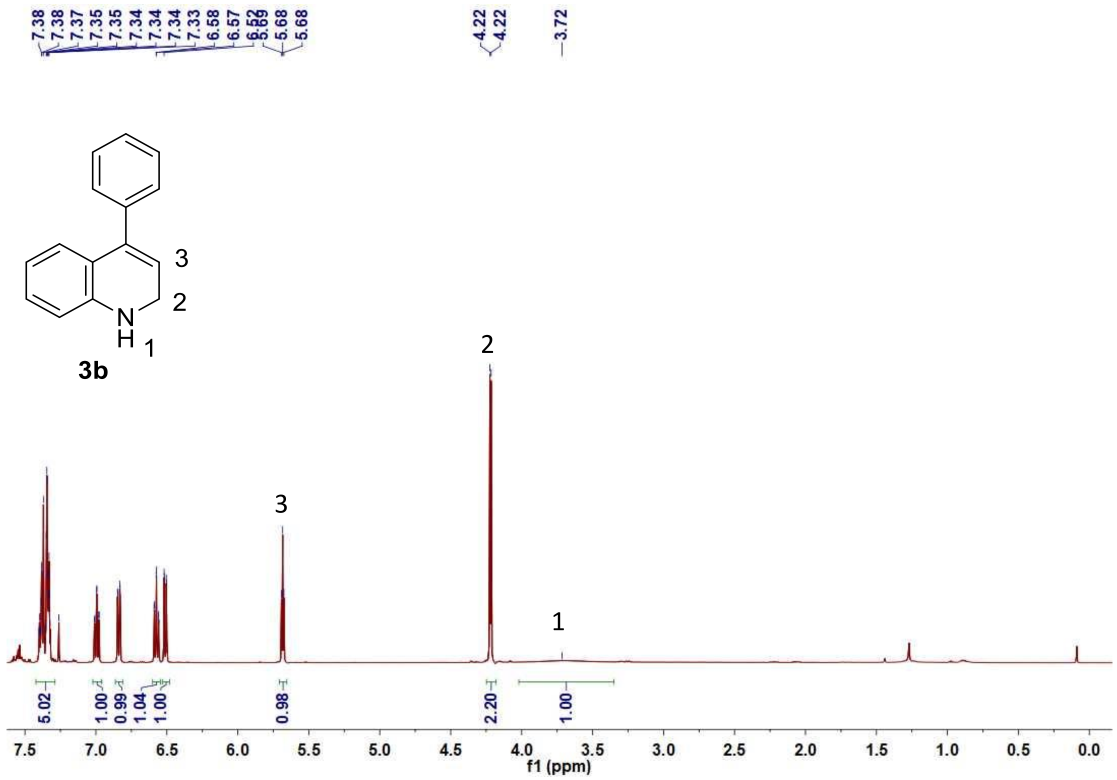


**Supplementary Figure 14. $^{1}$H NMR (500 MHz, $CDCl_3$) spectrum of 3b.**

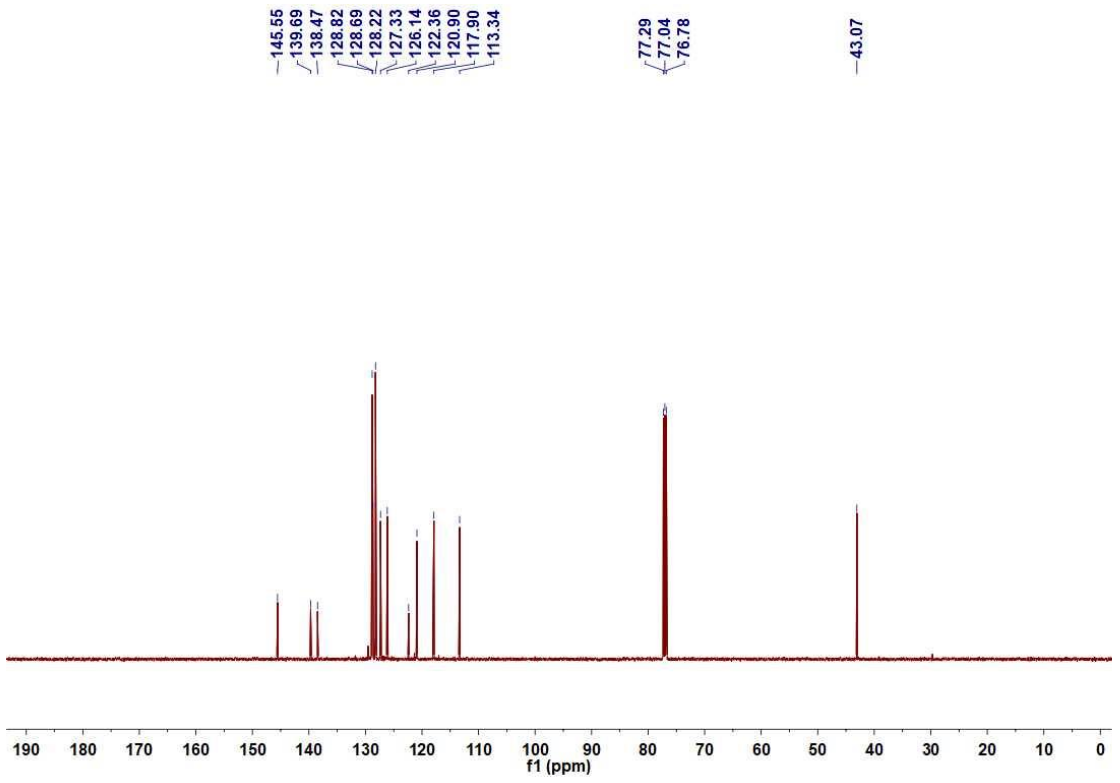


**Supplementary Figure 15. $^{13}$C NMR (126 MHz, $CDCl_3$) spectrum of 3b.**

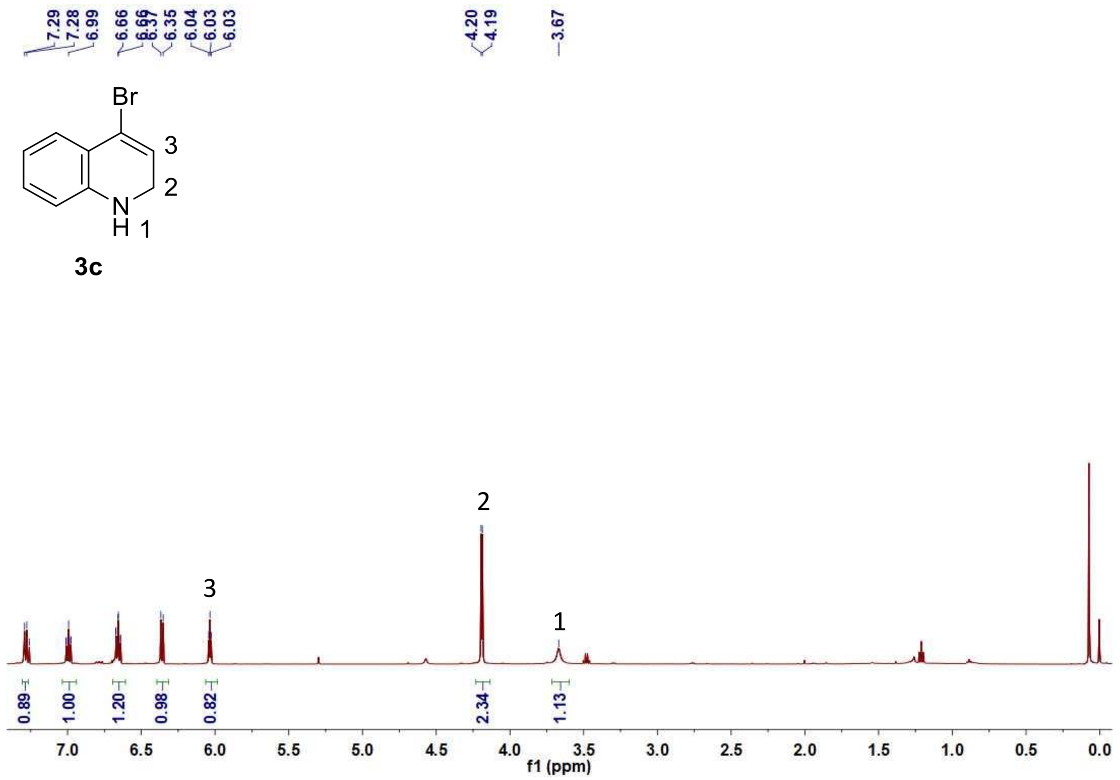


**Supplementary Figure 16. $^1$H NMR (500 MHz, $CDCl_3$) spectrum of 3c.**

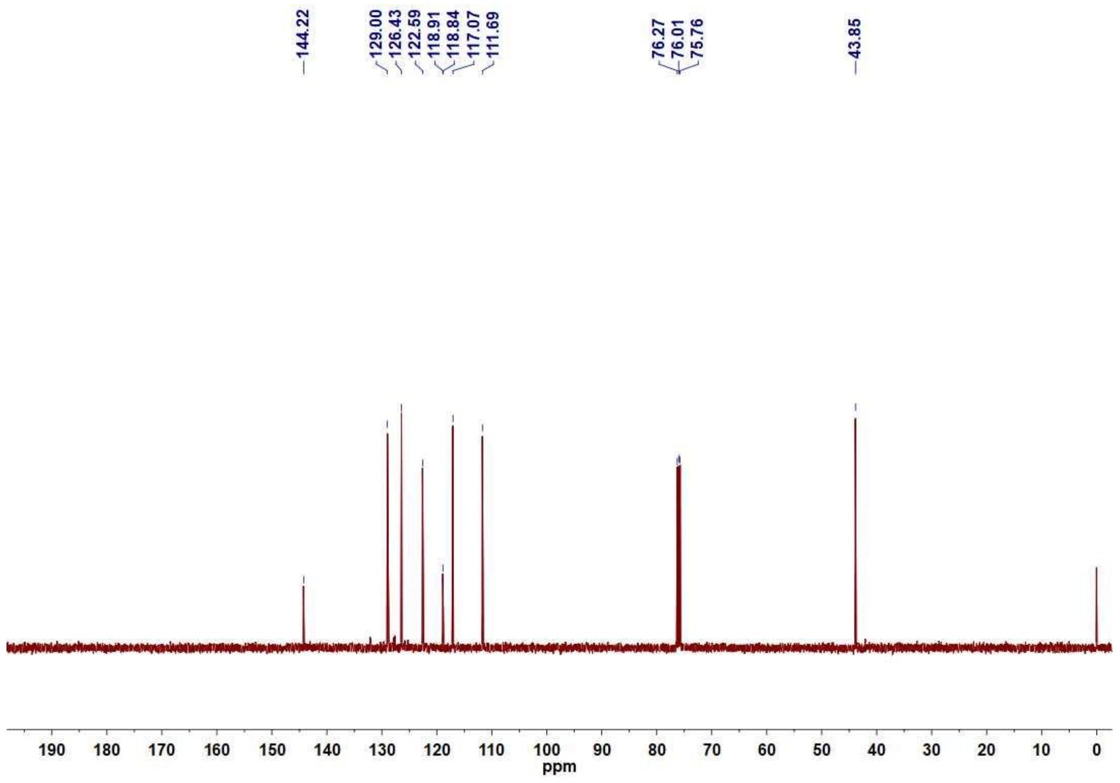


**Supplementary Figure 17.** $^{13}$**C NMR (126 MHz,** $CDCl_3$**) spectrum of 3c.**

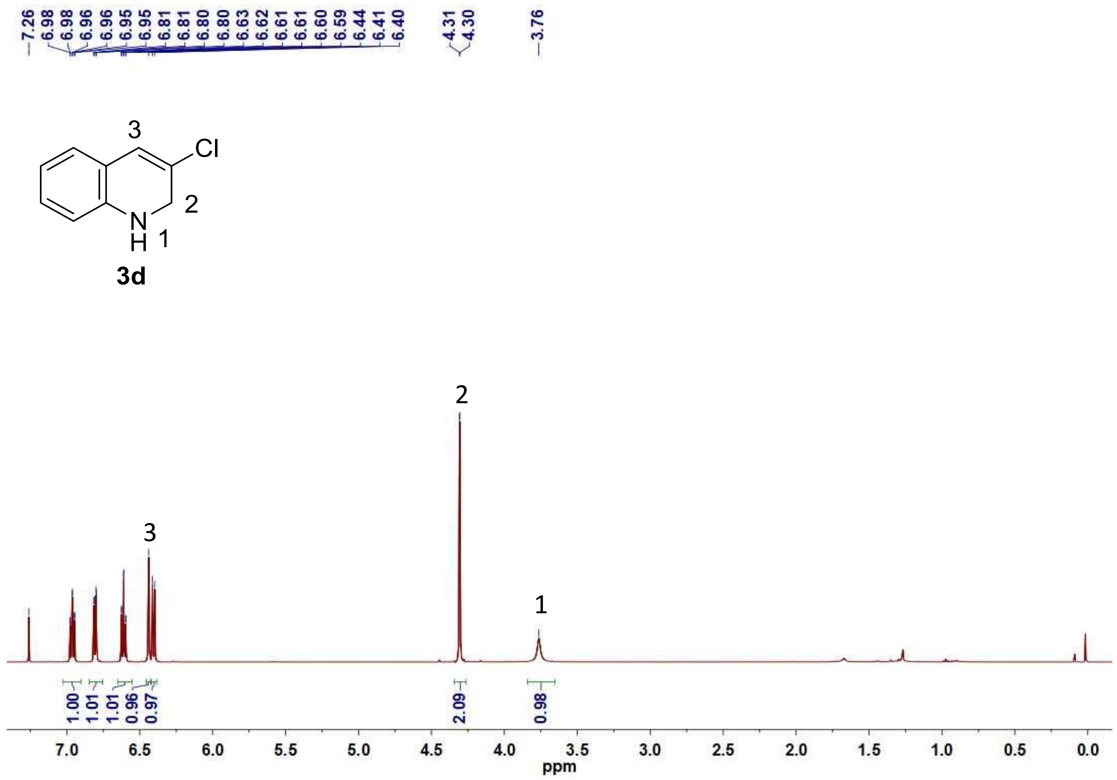


**Supplementary Figure 18. $^{1}$H NMR (500 MHz, $CDCl_3$) spectrum of 3d.**

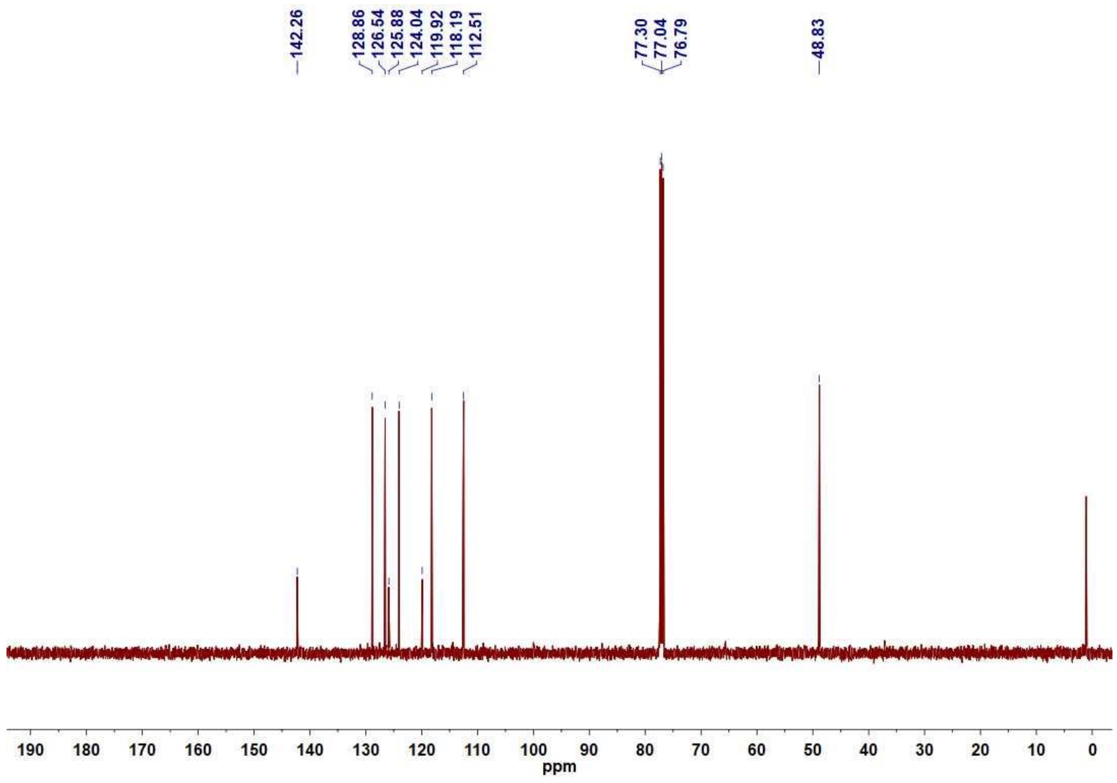


**Supplementary Figure 19.** **$^{13}$C NMR (126 MHz, $CDCl_3$) spectrum of 3d.**

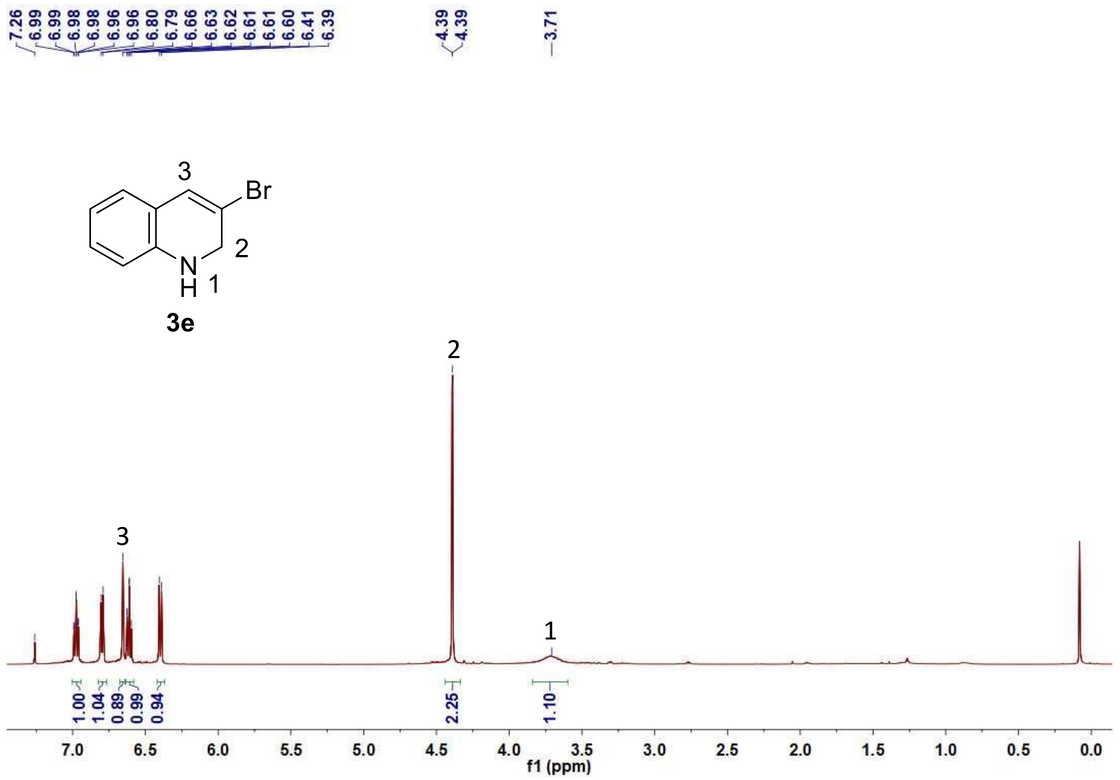


**Supplementary Figure 20. $^{1}$H NMR (500 MHz, $CDCl_3$) spectrum of 3e.**

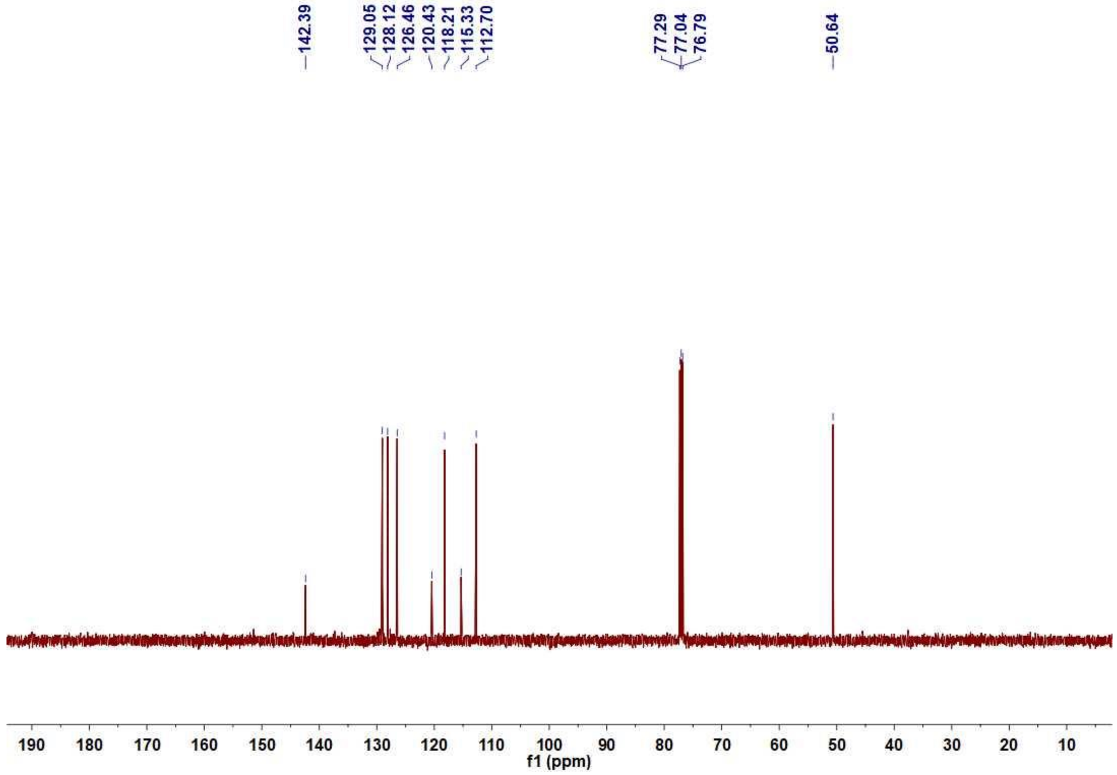


**Supplementary Figure 21. $^{13}C$ NMR (126 MHz, $CDCl_3$) spectrum of 3e.**

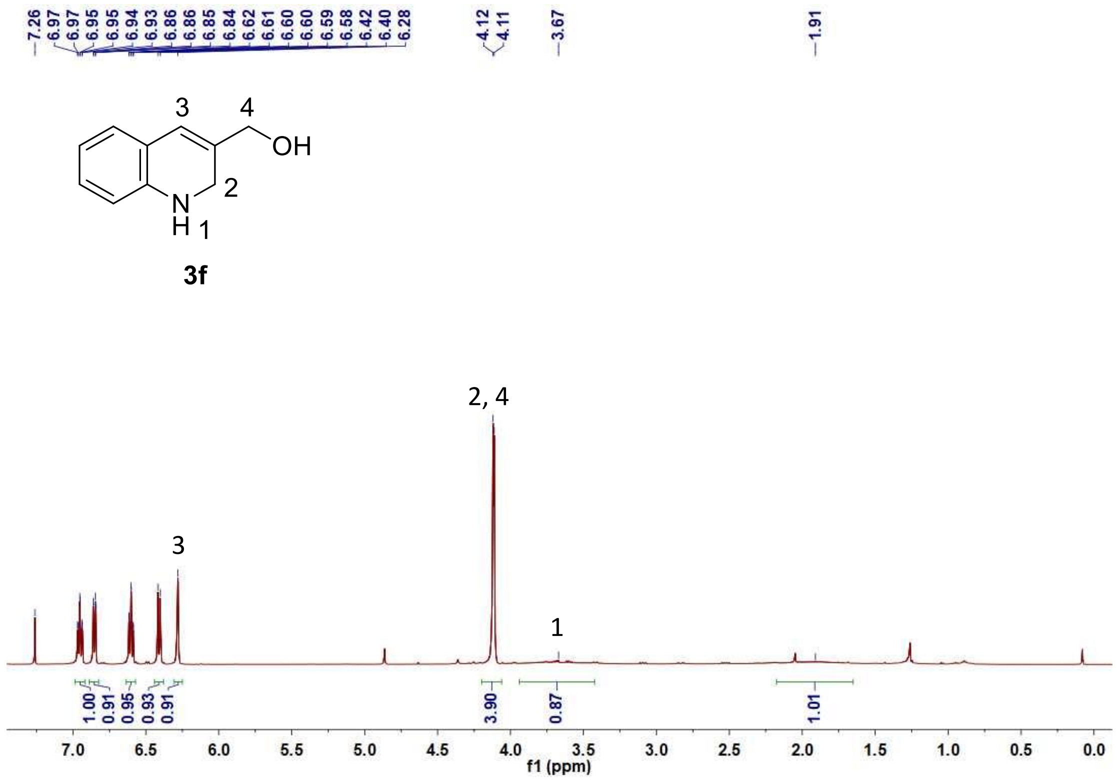


**Supplementary Figure 22. $^{1}$H NMR (500 MHz, $CDCl_3$) spectrum of 3f.**

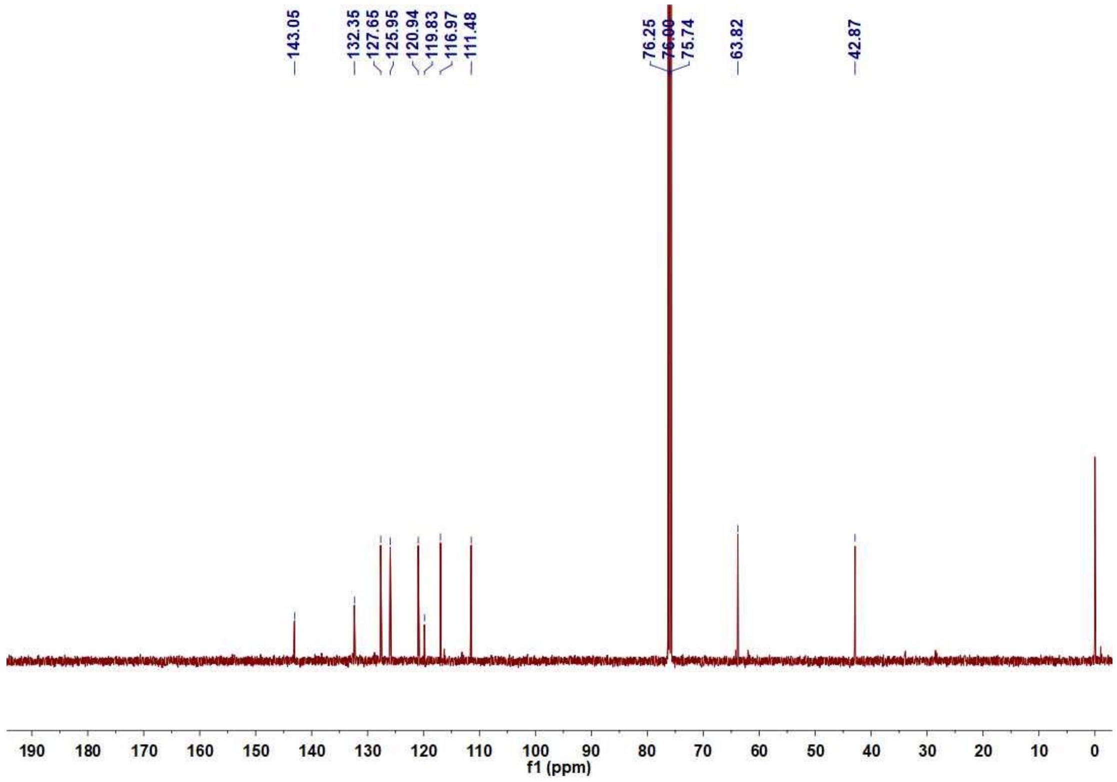


**Supplementary Figure 23.** **$^{13}$C NMR (126 MHz, $CDCl_3$) spectrum of 3f.**

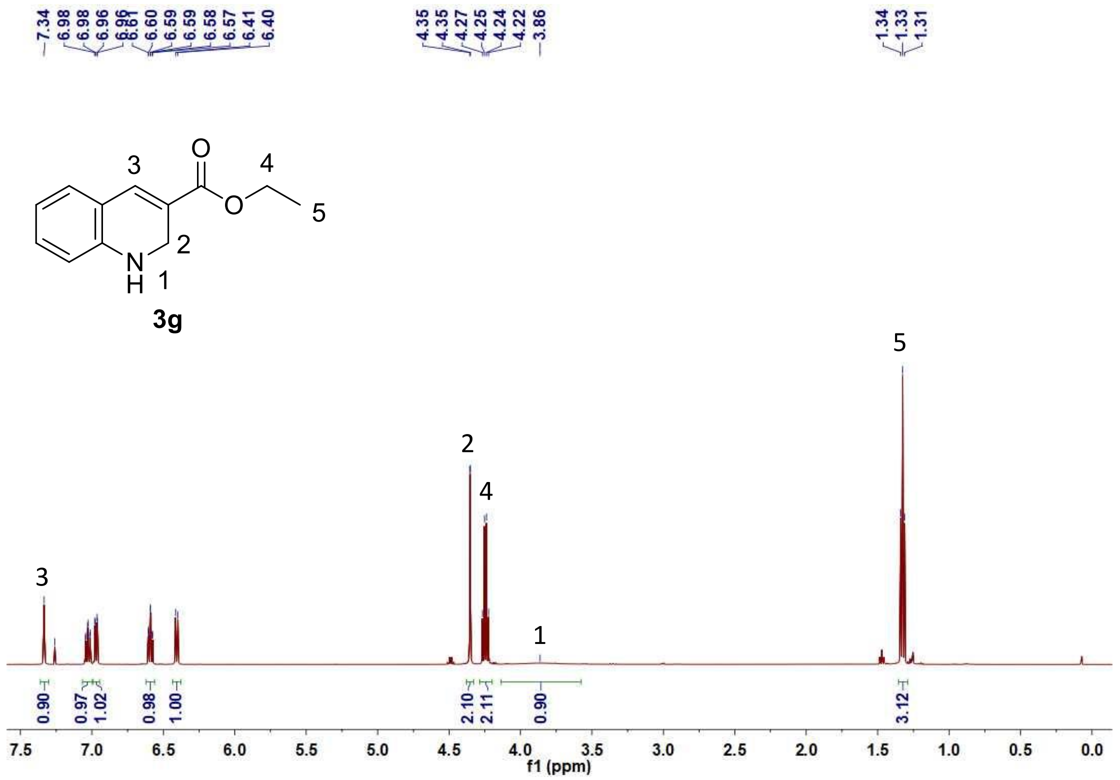


**Supplementary Figure 24. $^1$H NMR (500 MHz, $CDCl_3$) spectrum of 3g.**

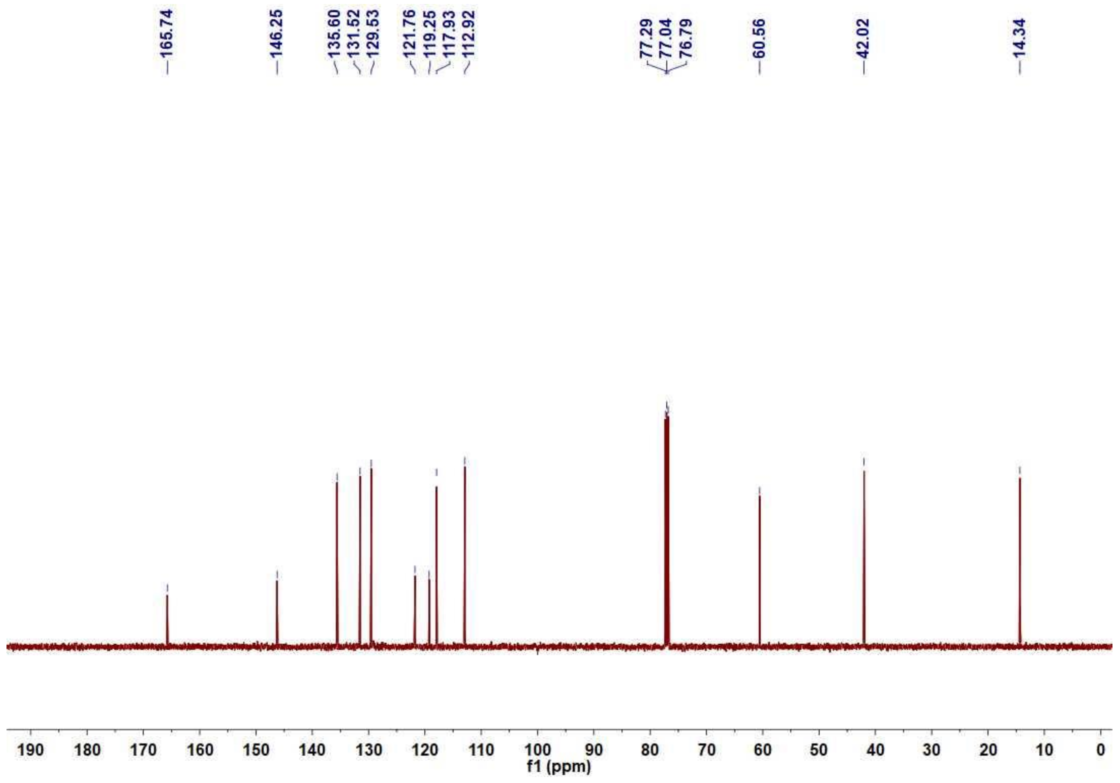


**Supplementary Figure 25.** **$^{13}C$ NMR (126 MHz, $CDCl_3$) spectrum of 3g.**

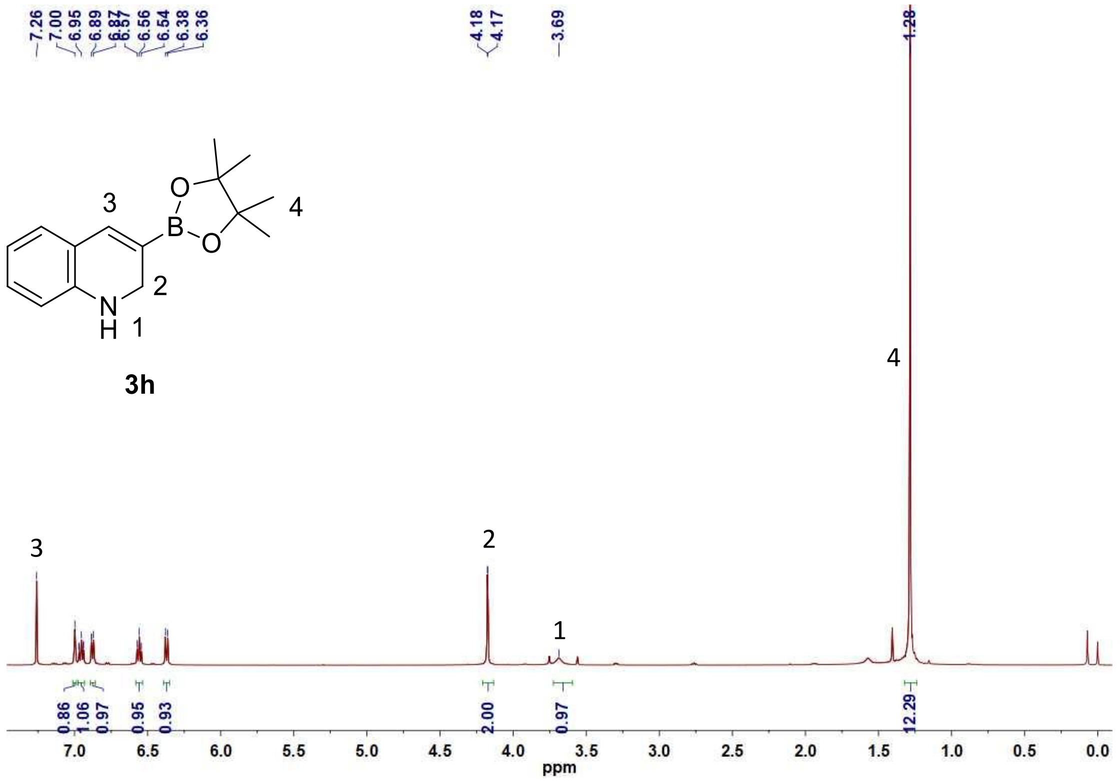


**Supplementary Figure 26. $^{1}$H NMR (500 MHz, $CDCl_3$) pectrum of 3h.**

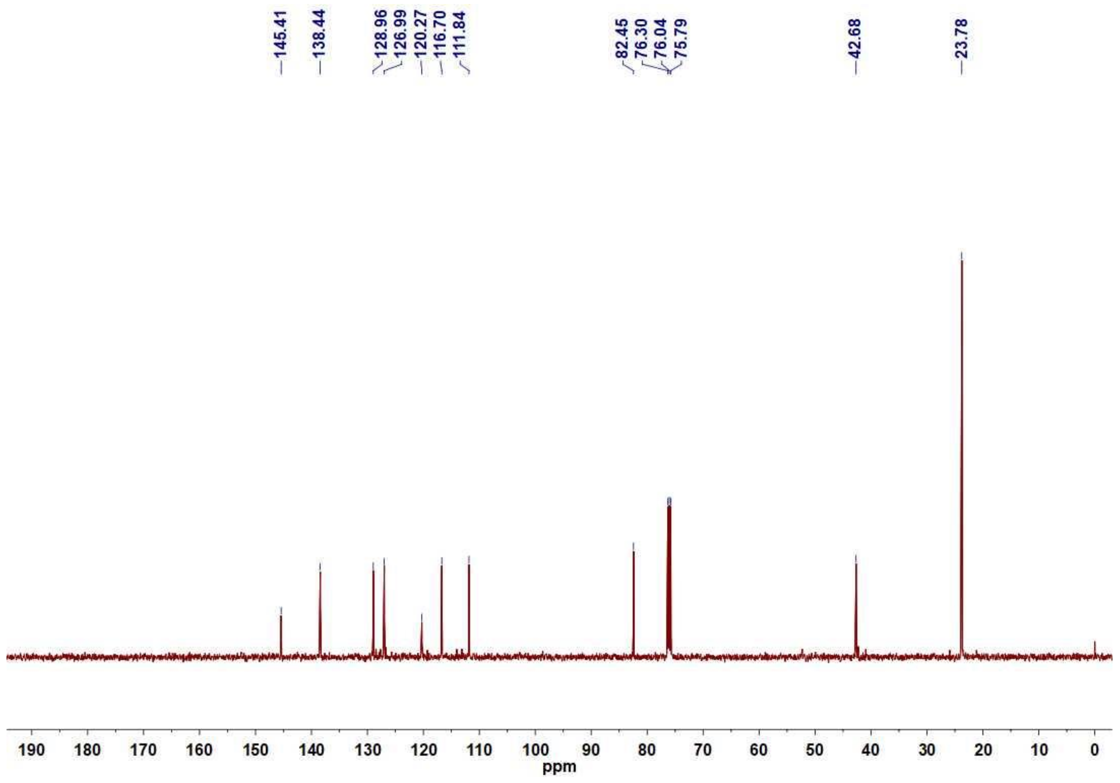


**Supplementary Figure 27. $^{13}$C NMR (126 MHz, $CDCl_3$) spectrum of 3h.**

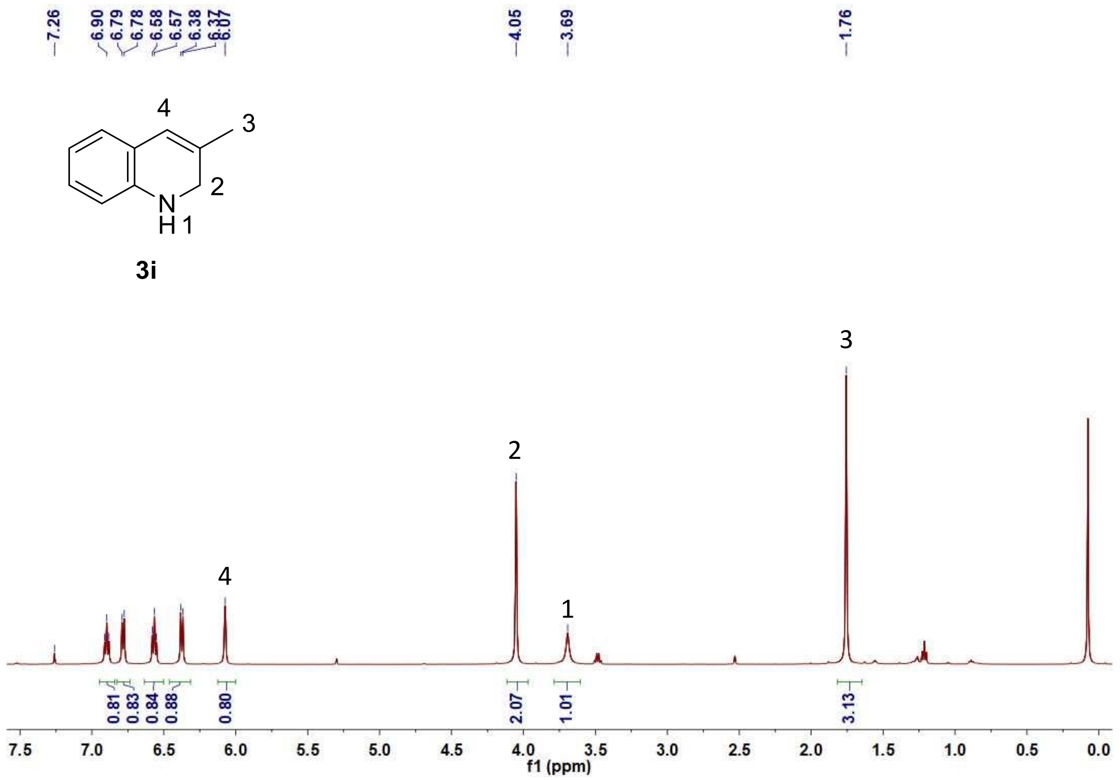


**Supplementary Figure 28. $^{1}$H NMR (500 MHz, $CDCl_3$) spectrum of 3i.**

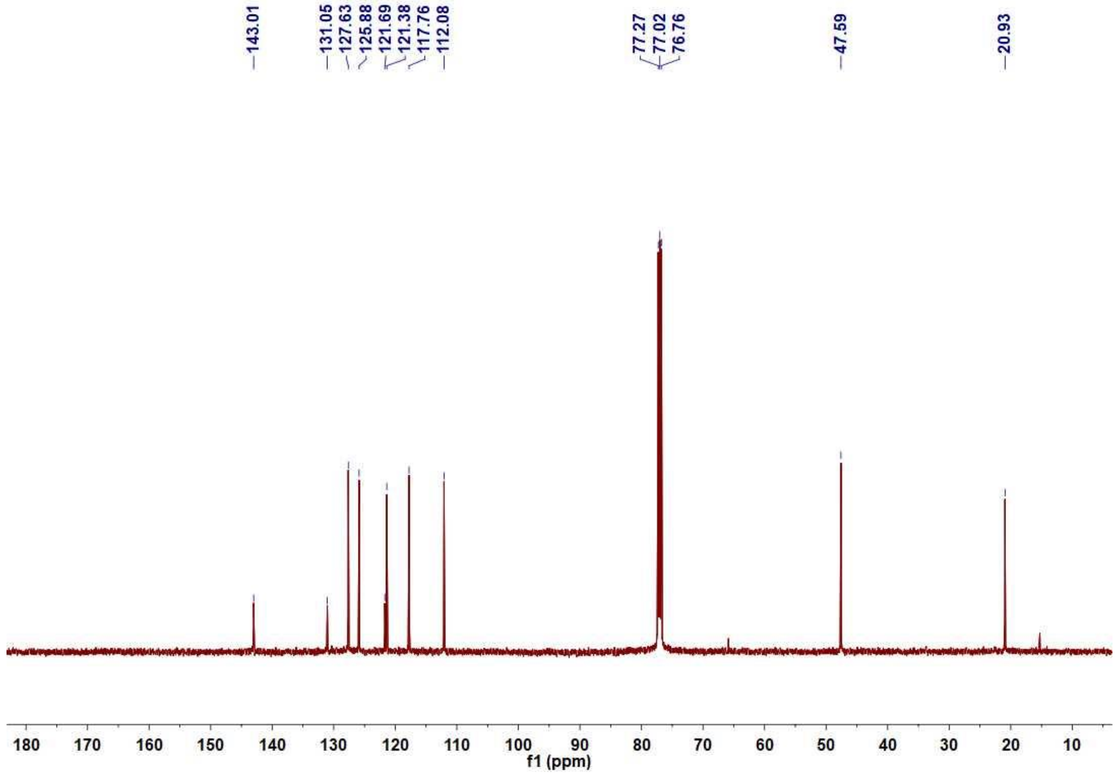


**Supplementary Figure 29.** **$^{13}$C NMR (126 MHz, $CDCl_3$) spectrum of 3i.**

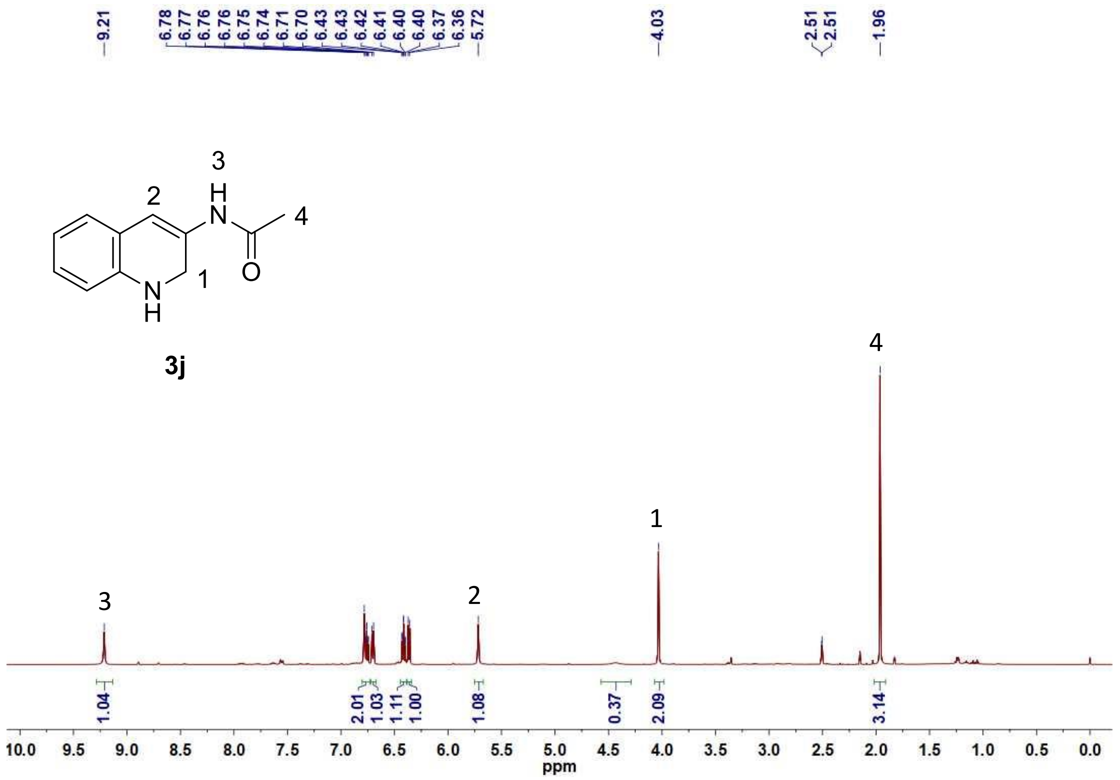


**Supplementary Figure 30. $^{1}$H NMR (500 MHz, DMSO-*d*$_6$) spectrum of 3j.**

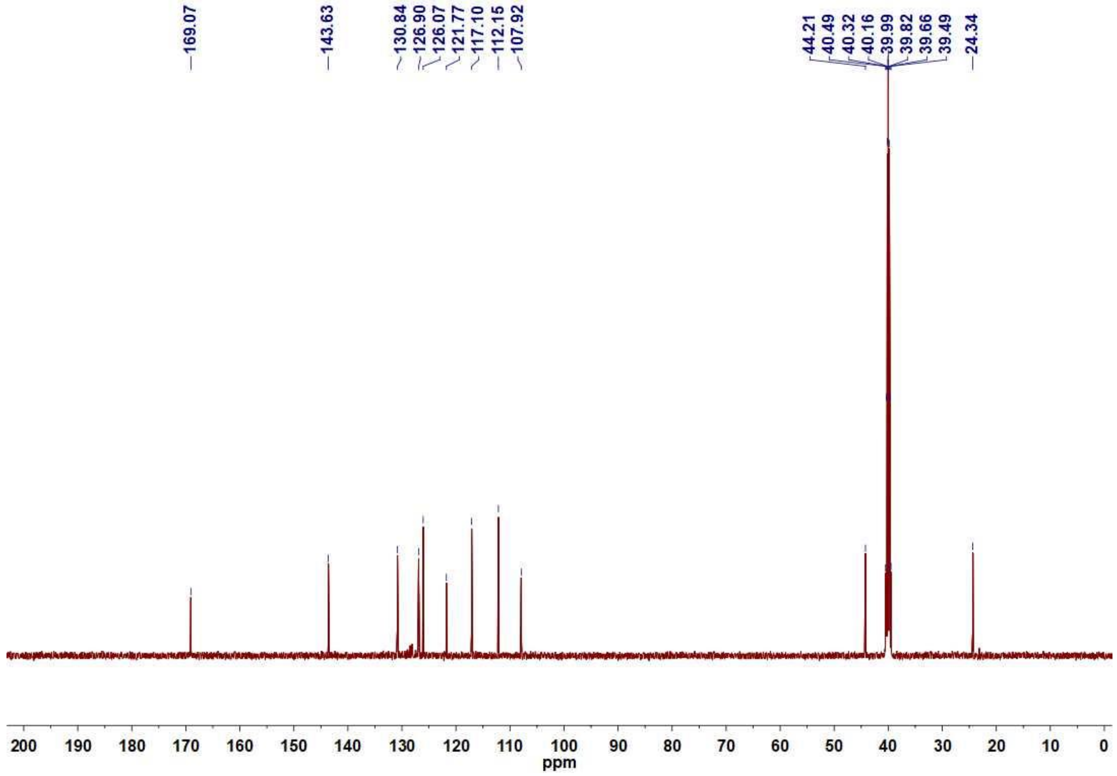


**Supplementary Figure 31. $^{13}$C NMR (126 MHz, DMSO-$d_6$) spectrum of 3j.**

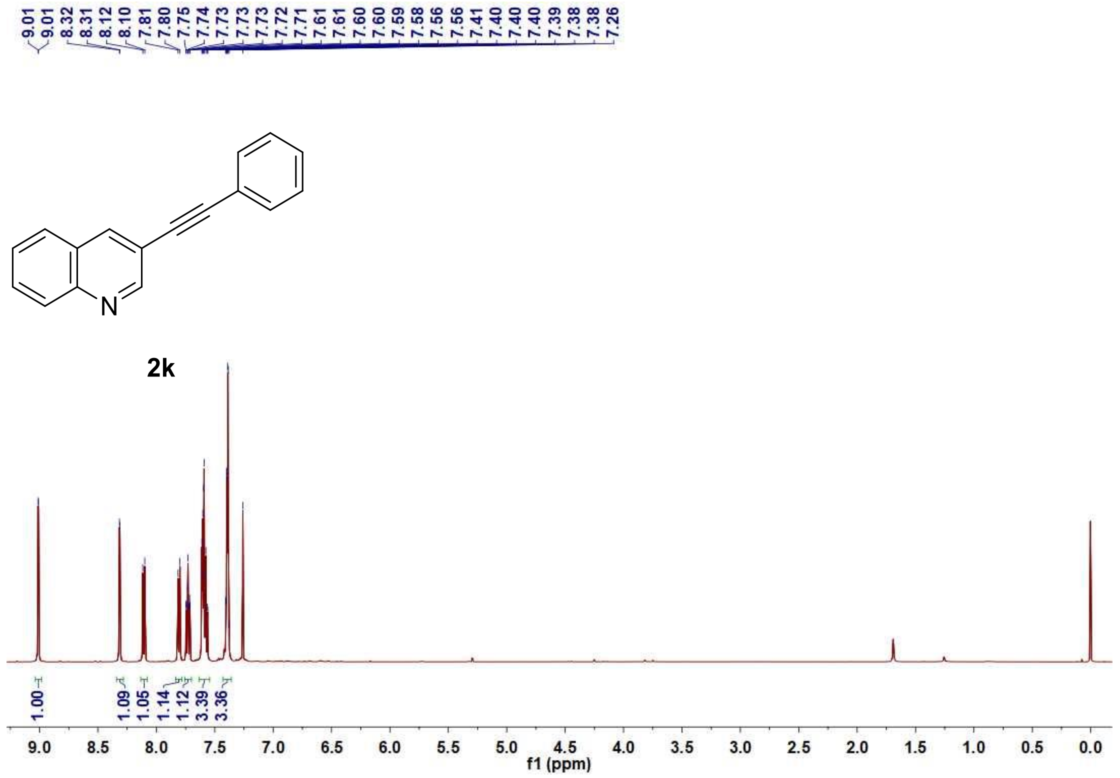


**Supplementary Figure 32.** **$^{1}$H NMR (500 MHz, CDCl$_3$) spectrum of 2k.**

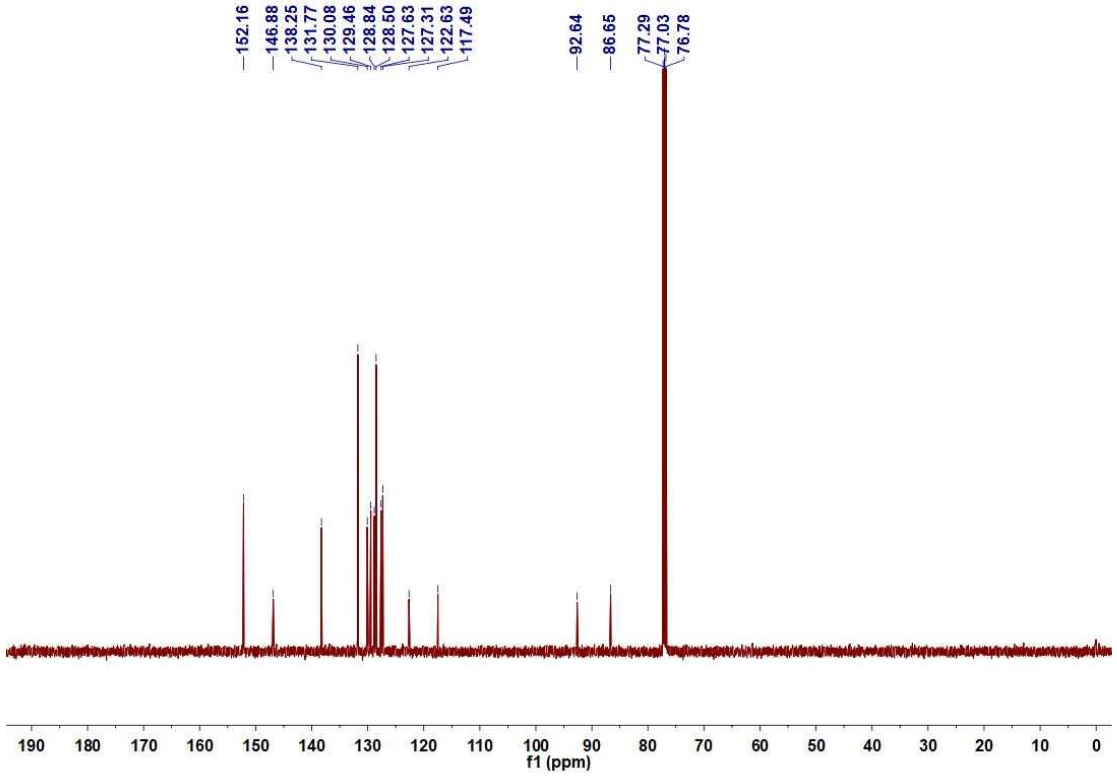


**Supplementary Figure 33.** $^{13}$**C NMR (126 MHz, CDCl$_3$) spectrum of 2k.**

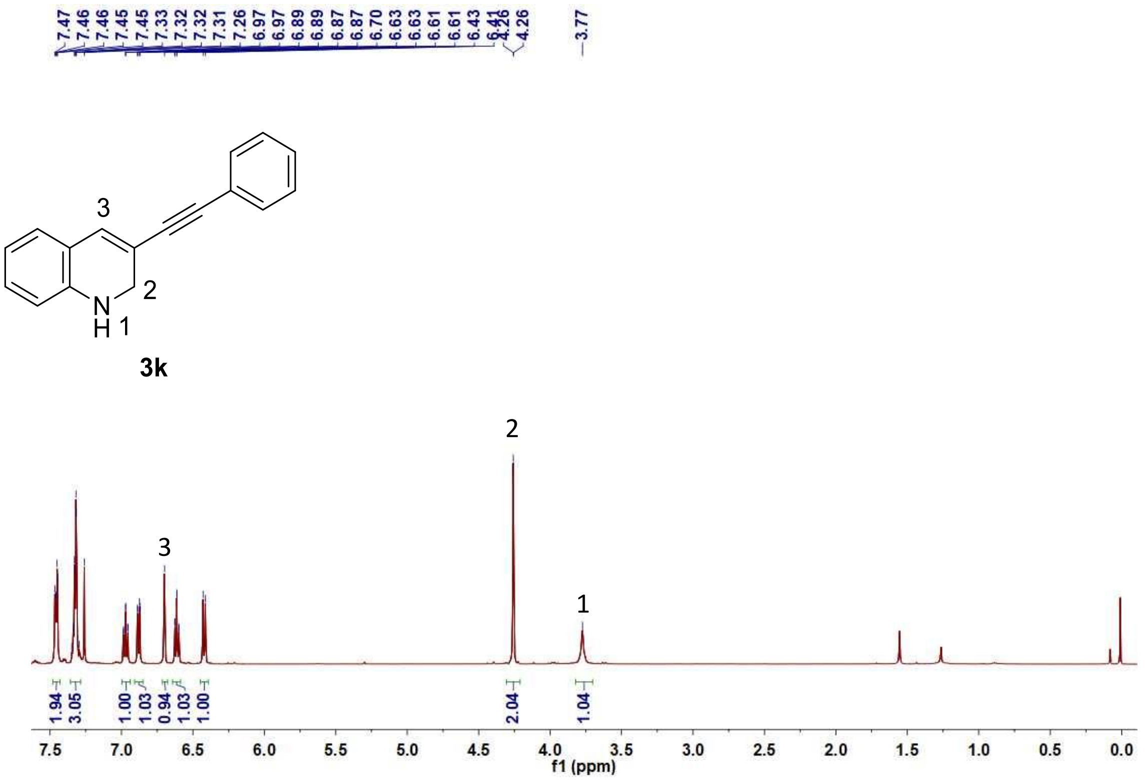

**Supplementary Figure 34.** **$^{1}$H NMR (500 MHz, $CDCl_3$) spectrum of 3k.**

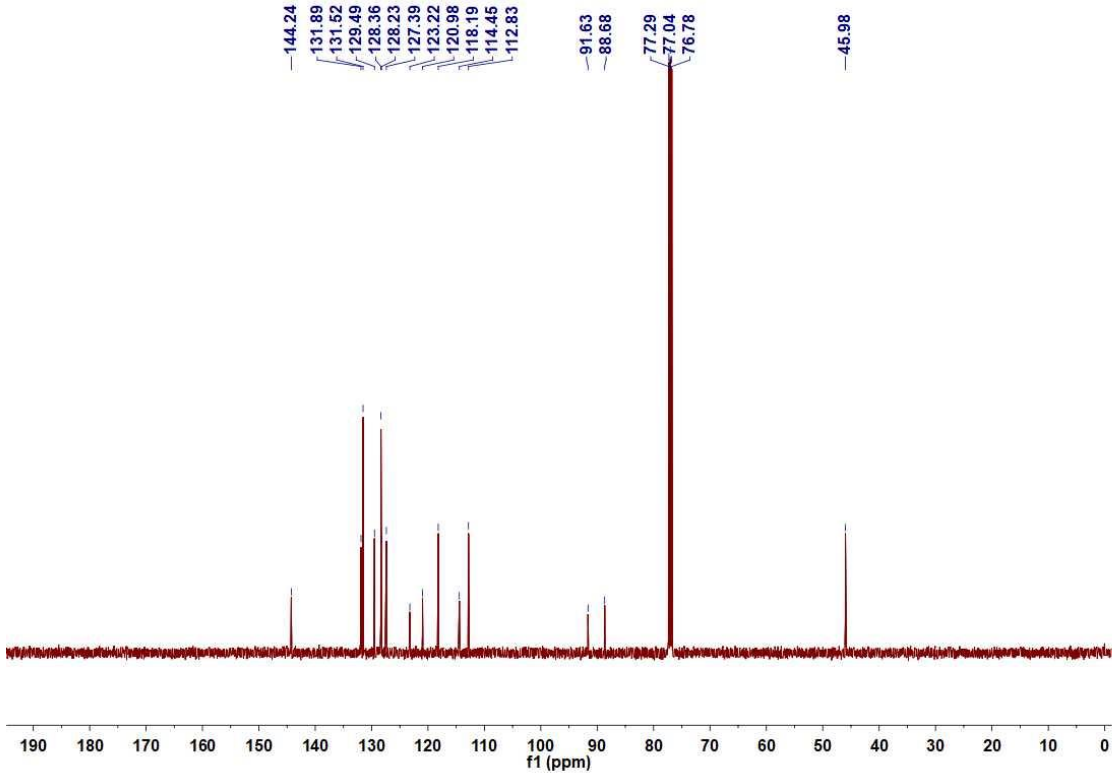


**Supplementary Figure 35. $^{13}$C NMR (126 MHz, CDCl$_3$) spectrum of 3k.**

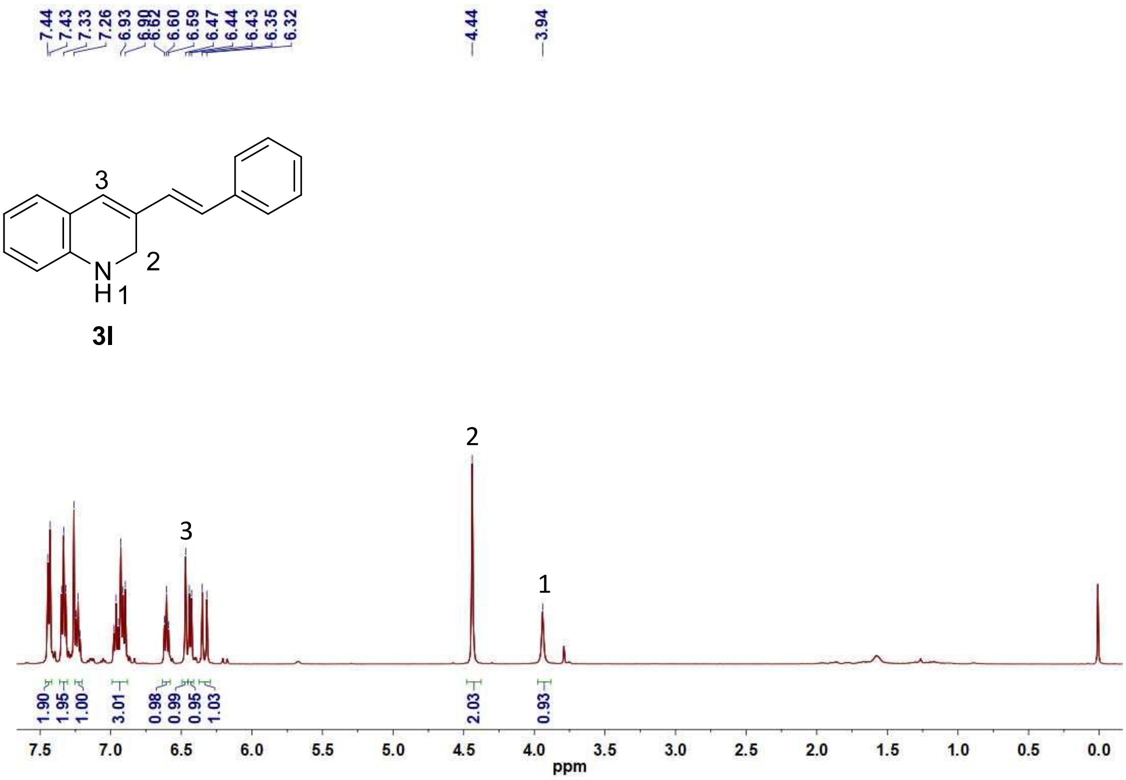


**Supplementary Figure 36. $^{1}H$ NMR (500 MHz, $CDCl_3$) spectrum of 3l.**

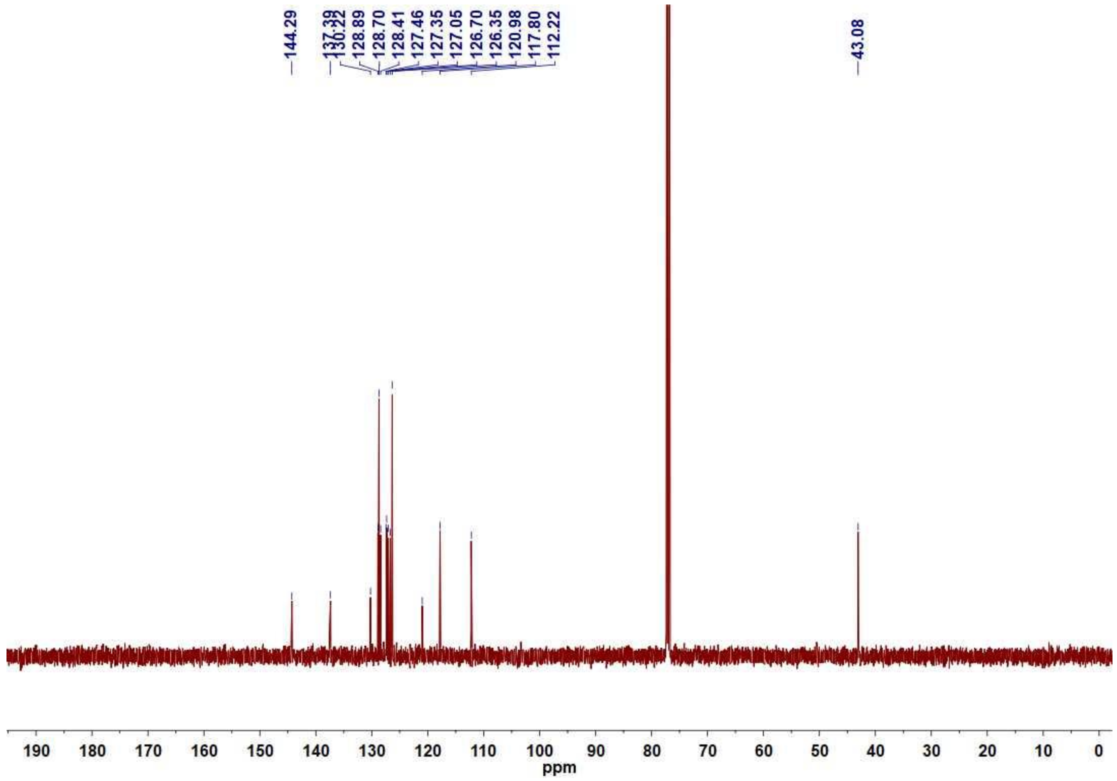


**Supplementary Figure 37.** $^{13}$**C NMR (126 MHz,** $CDCl_3$**) spectrum of 3l.**

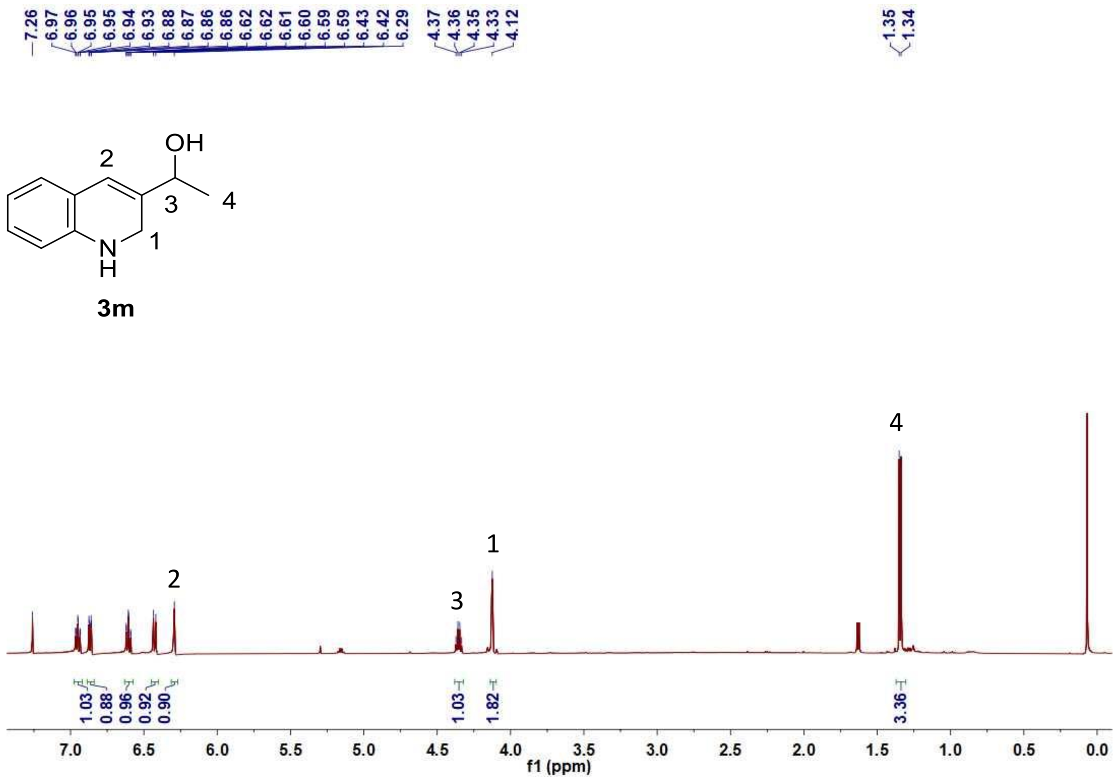


**Supplementary Figure 38.** **$^{1}$H NMR (500 MHz, $CDCl_3$) pectrum of 3m.**

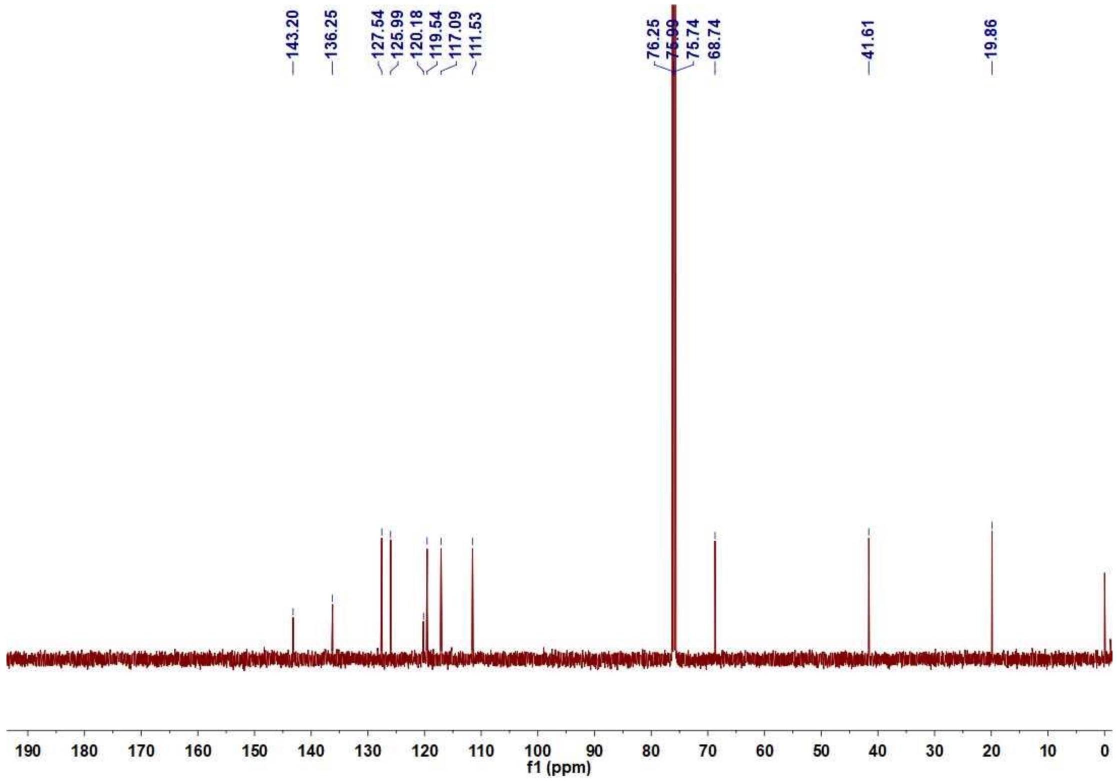


**Supplementary Figure 39.** $^{13}$**C NMR (126 MHz,** $CDCl_3$**) spectrum of 3m.**

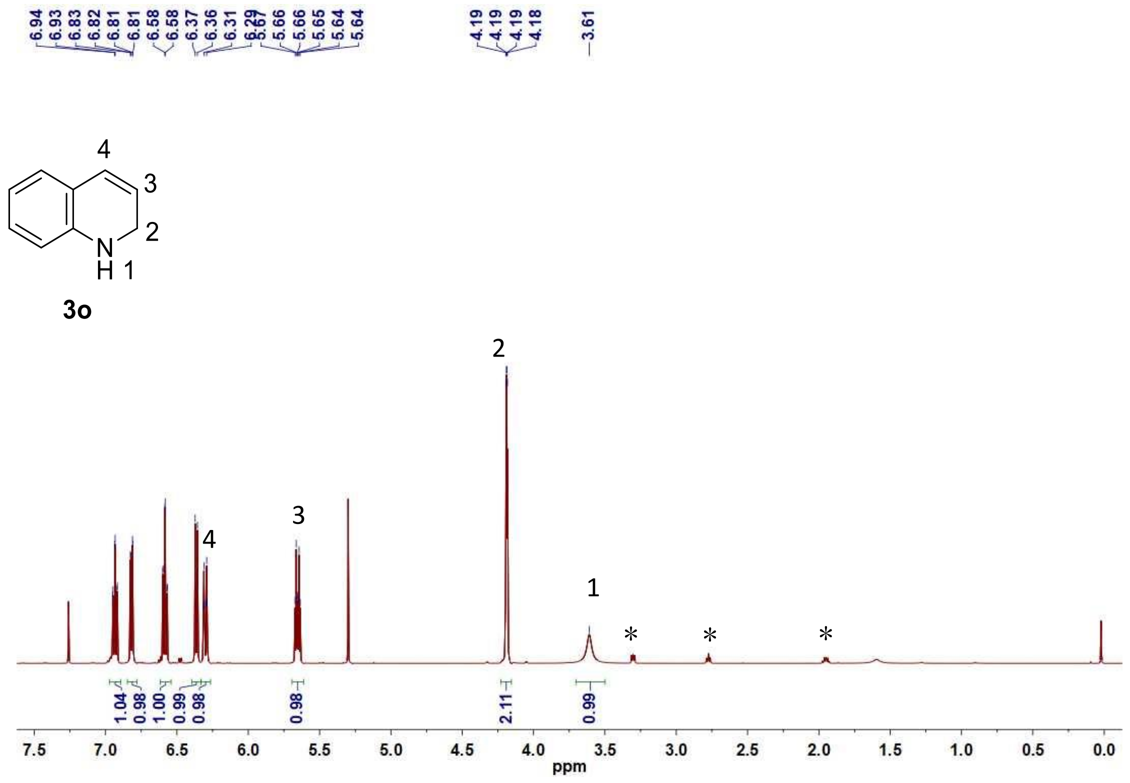


**Supplementary Figure 40. $^{1}$H NMR (500 MHz, $CDCl_3$) spectrum of 3o.** (* for tetrahydroquinoline product)

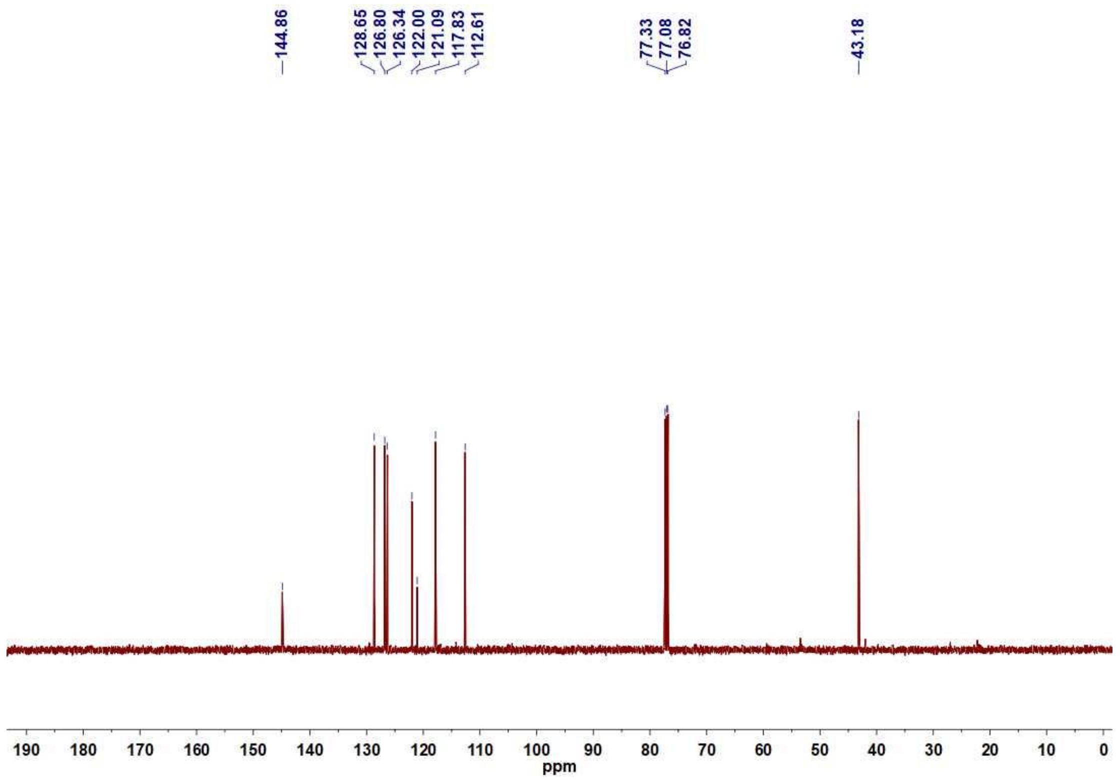


**Supplementary Figure 41.** $^{13}$**C NMR (126 MHz, CDCl$_3$) spectrum of 3o.**

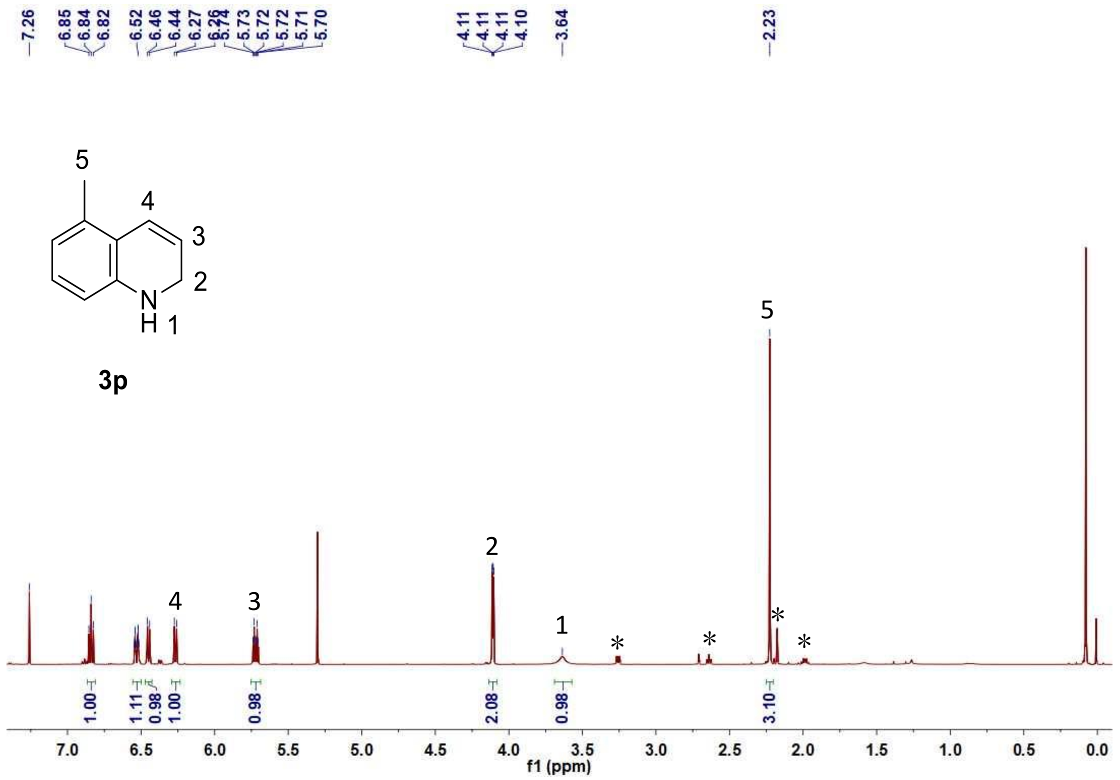


**Supplementary Figure 42. ¹H NMR (500 MHz, CDCl3) spectrum of 3p.** (∗ for tetrahydroquinoline product)

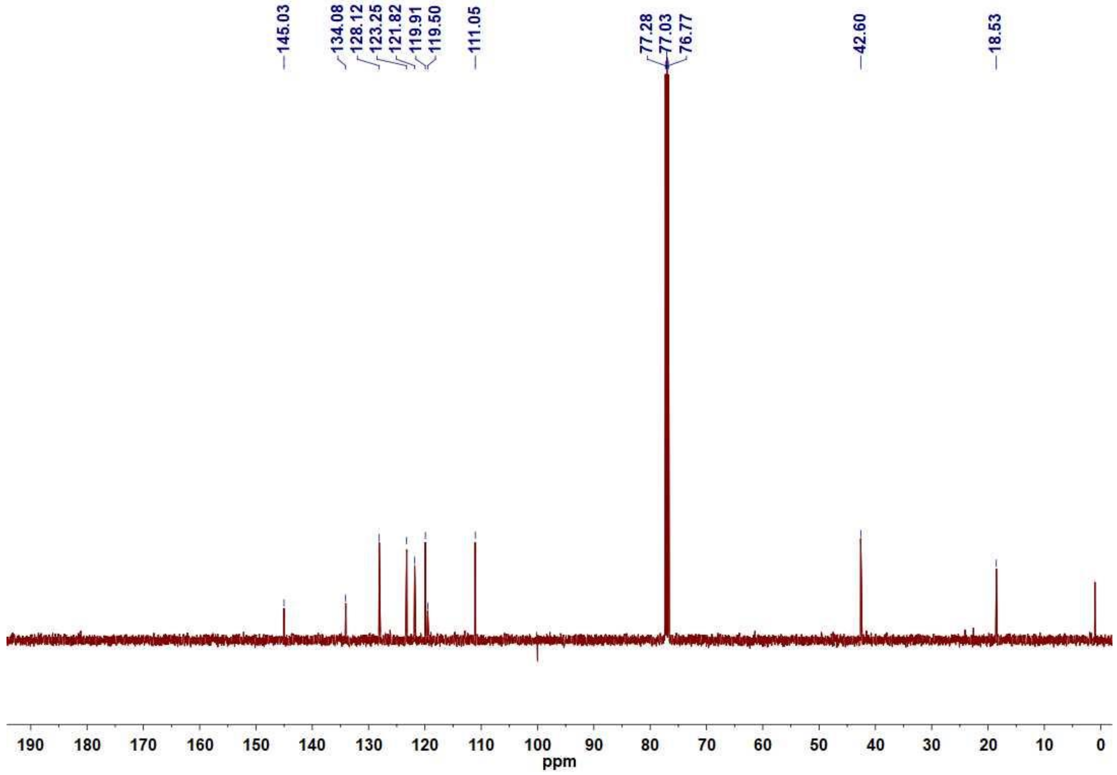


**Supplementary Figure 43. $^{13}C$ NMR (126 MHz, $CDCl_3$) spectrum of 3p.**

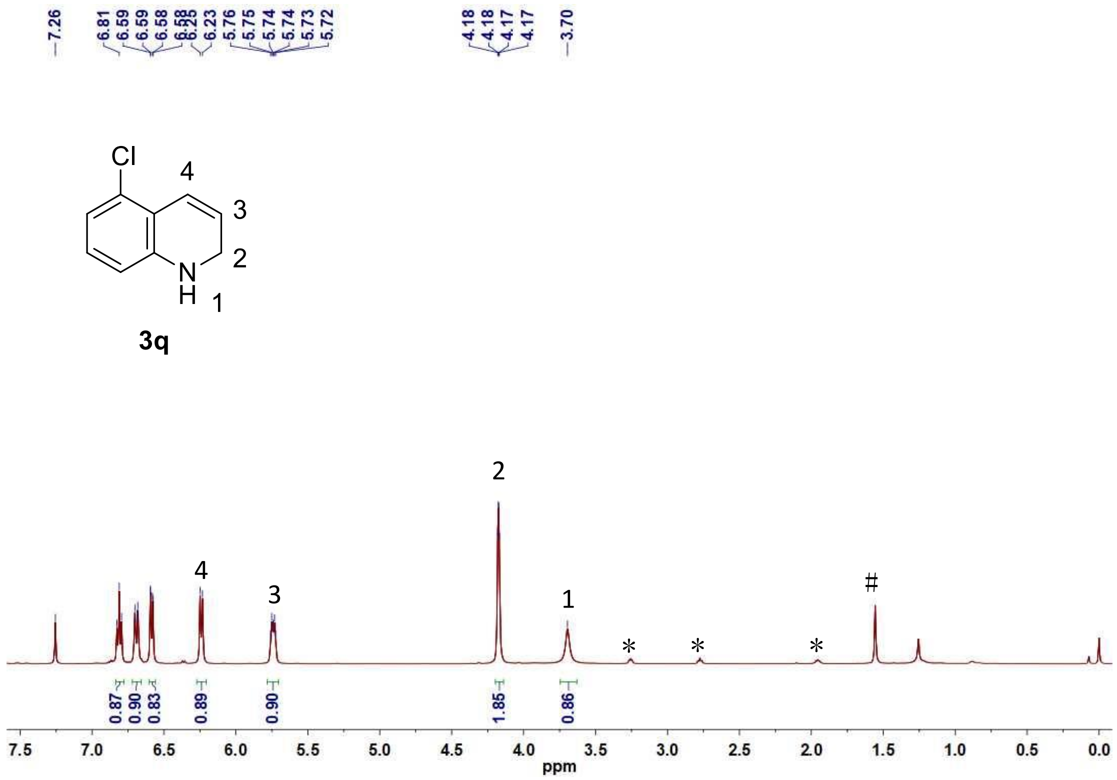

**Supplementary Figure 44. $^{1}$H NMR (500 MHz, $CDCl_3$)spectrum of 3q.** (* for tetrahydroquinoline product; # for water)

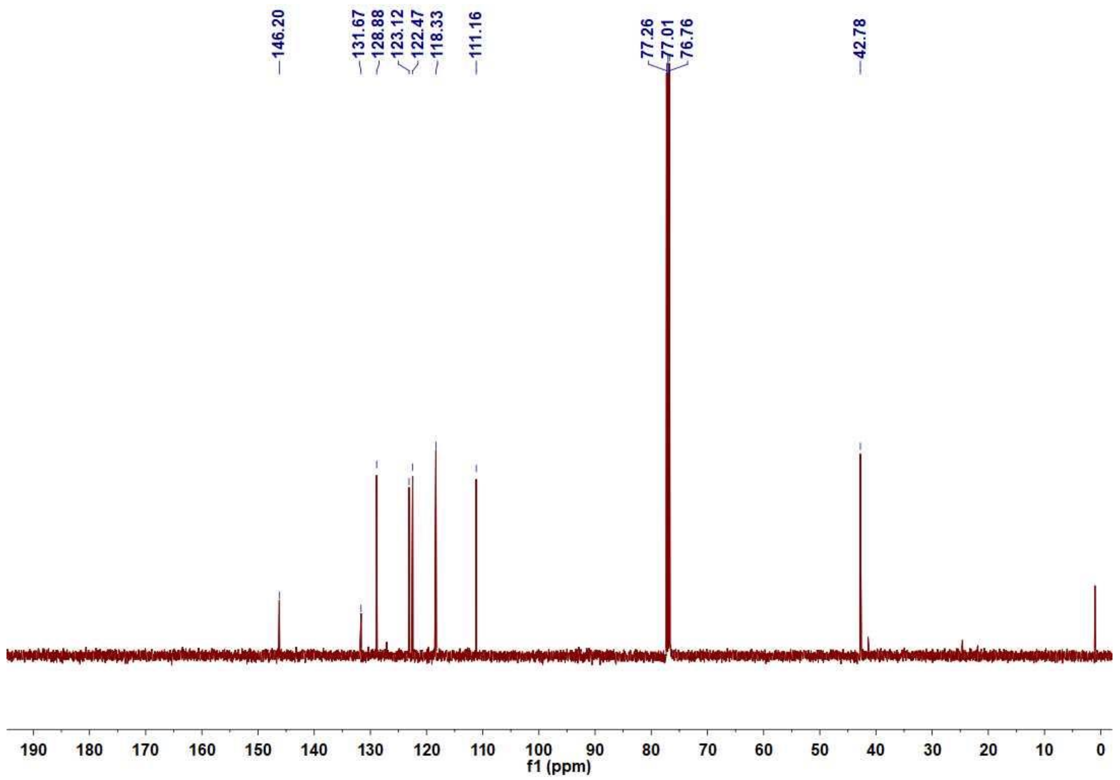


**Supplementary Figure 45. $^{13}$C NMR (126 MHz, $CDCl_3$) spectrum of 3q.**

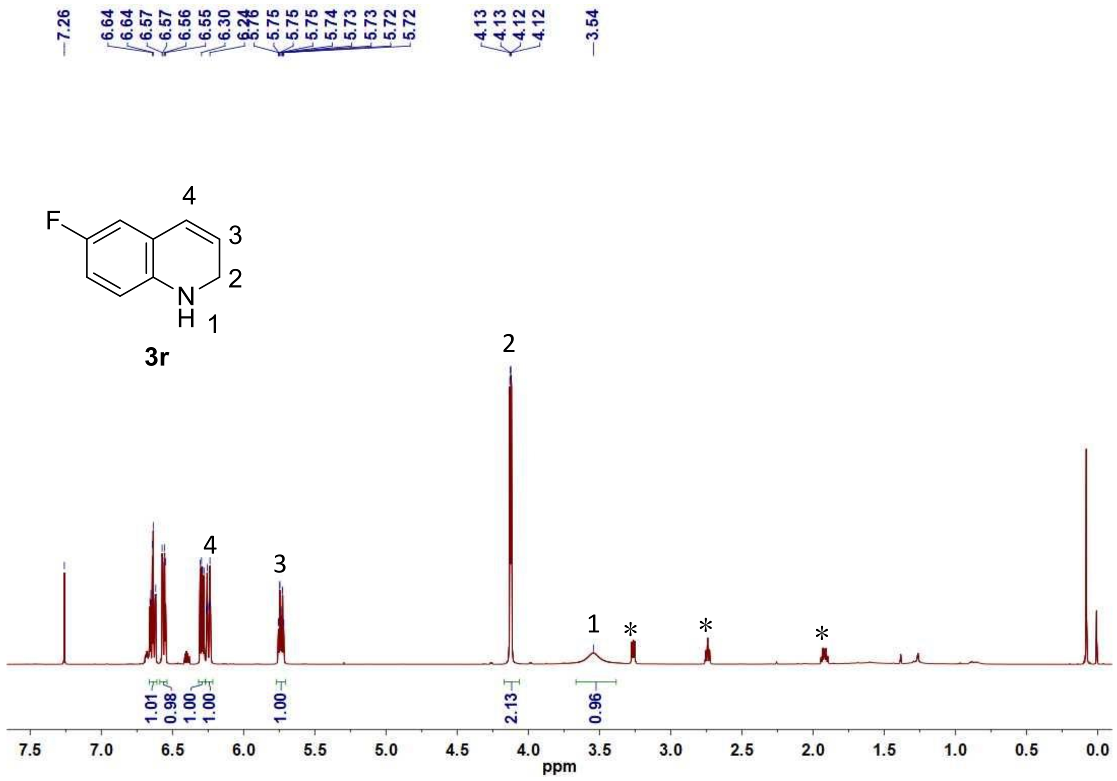


**Supplementary Figure 46. ¹H NMR (500 MHz, $CDCl_3$) spectrum of 3r.** (∗ for tetrahydroquinoline product)

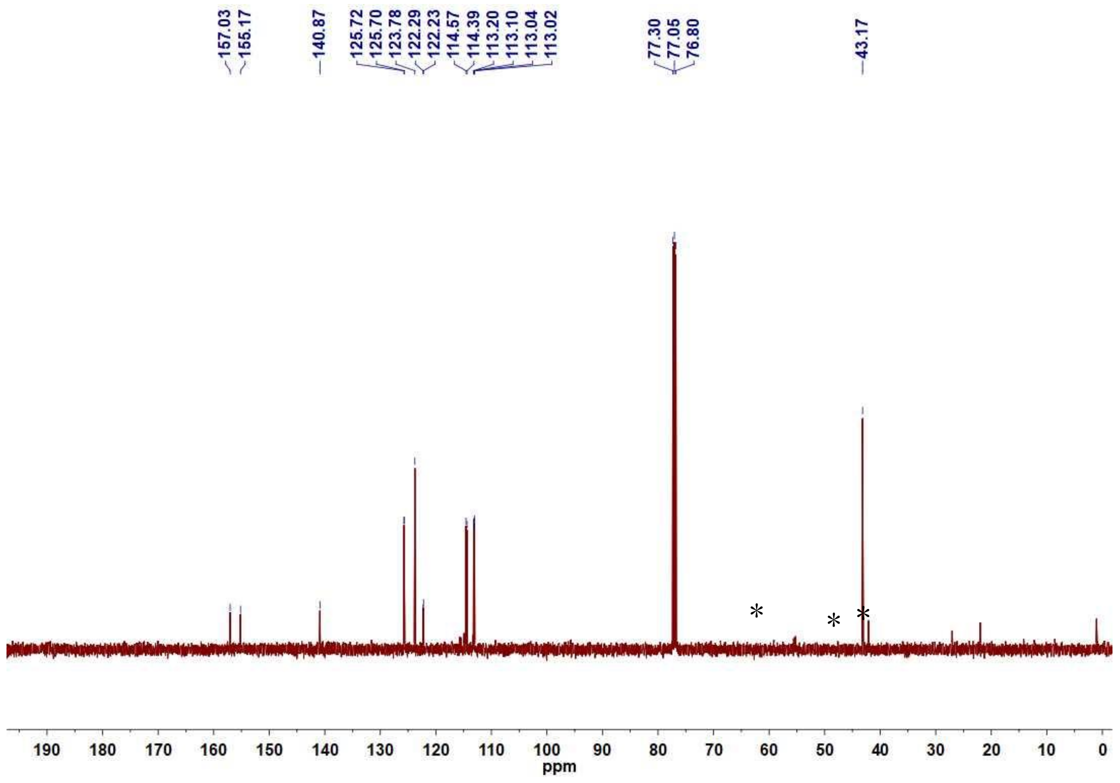


**Supplementary Figure 47. $^{13}$C NMR (126 MHz, $CDCl_3$) spectrum of 3r.** (* for tetrahydroquinoline product)

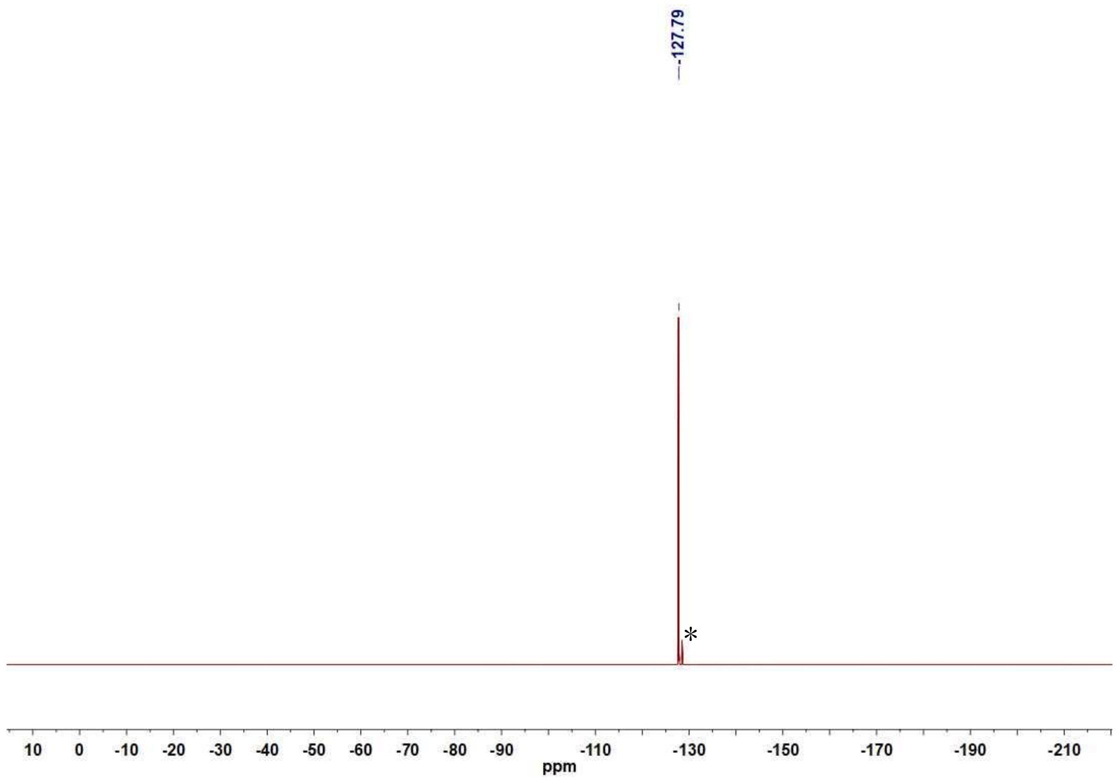


**Supplementary Figure 48. $^{19}$F NMR (471 MHz, $CDCl_3$) spectrum of 3r.** (∗ for tetrahydroquinoline product)

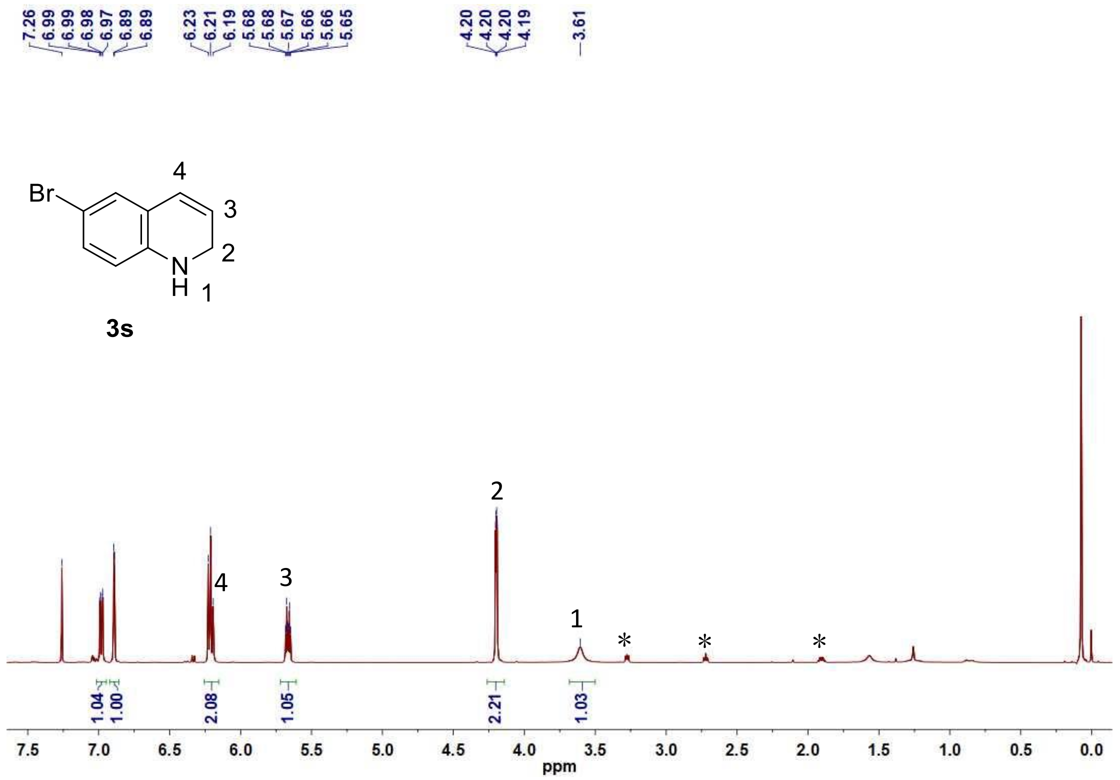


**Supplementary Figure 49. $^1$H NMR (500 MHz, $CDCl_3$) spectrum of 3s.** (* for tetrahydroquinoline product)

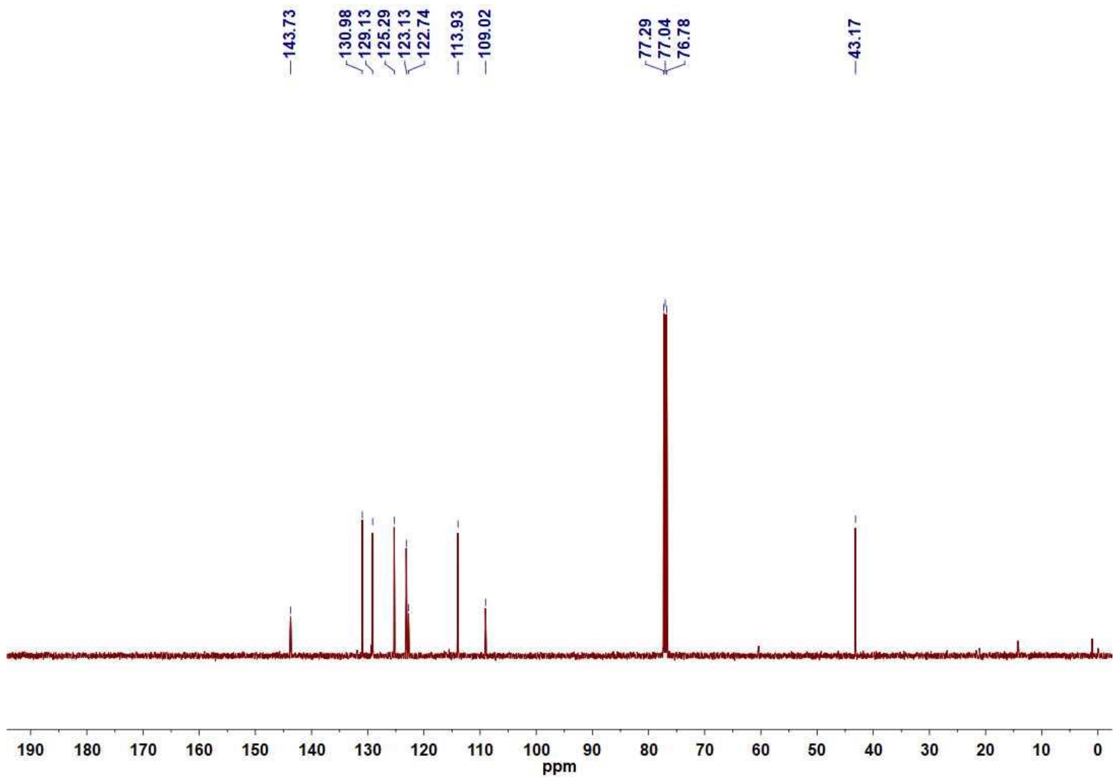


**Supplementary Figure 50.** $^{13}$**C NMR (126 MHz, CDCl$_3$) spectrum of 3s.**

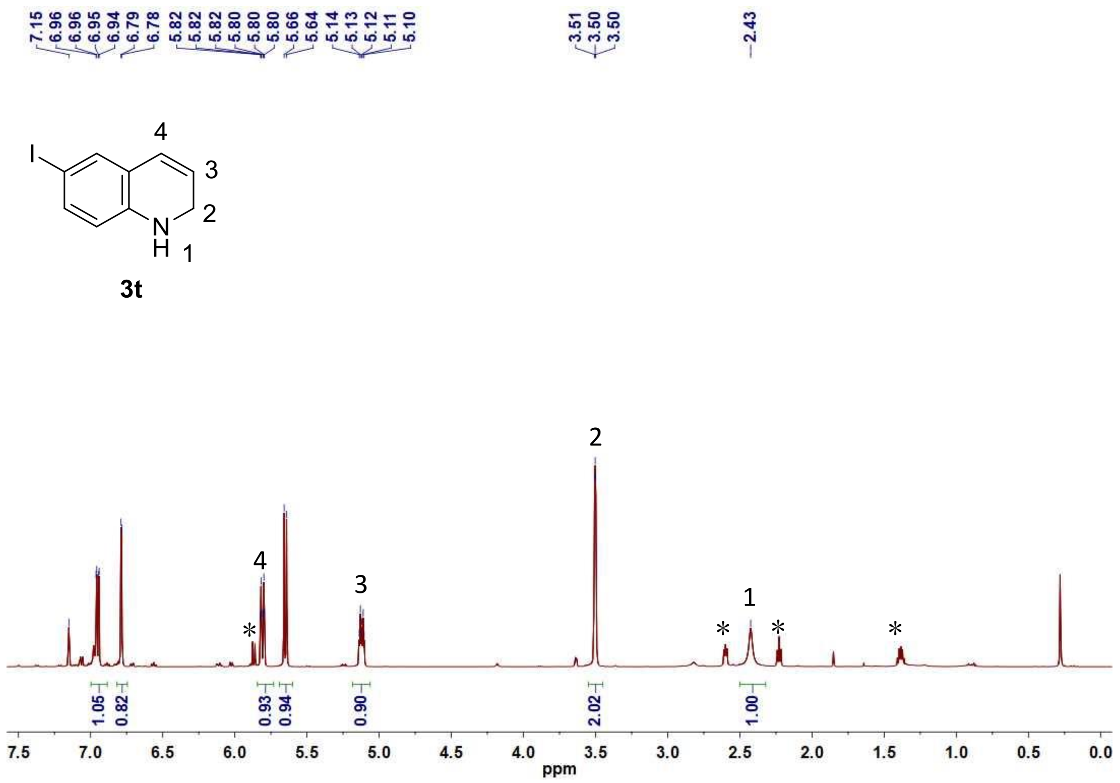


**Supplementary Figure 51. $^{1}$H NMR (500 MHz, $C_6D_6$) spectrum of 3t.** (∗ for tetrahydroquinoline product)

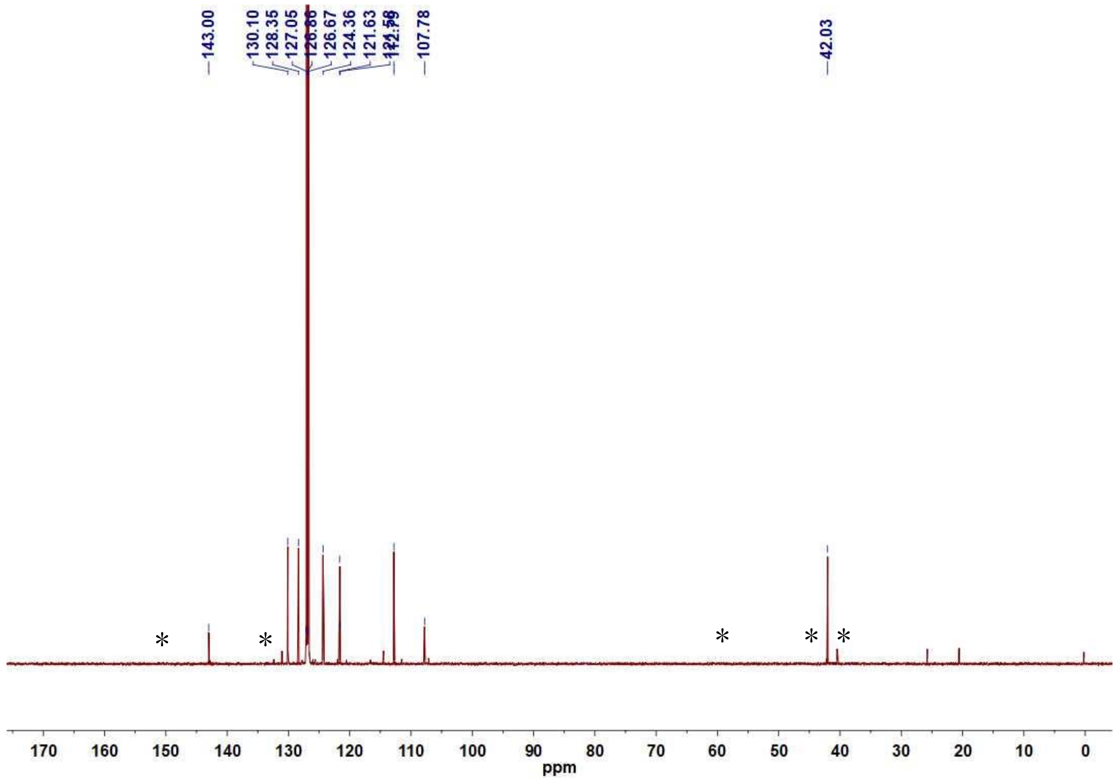


**Supplementary Figure 52. $^{13}C$ NMR (126 MHz, $C_6D_6$) spectrum of 3t.** (* for tetrahydroquinoline product)

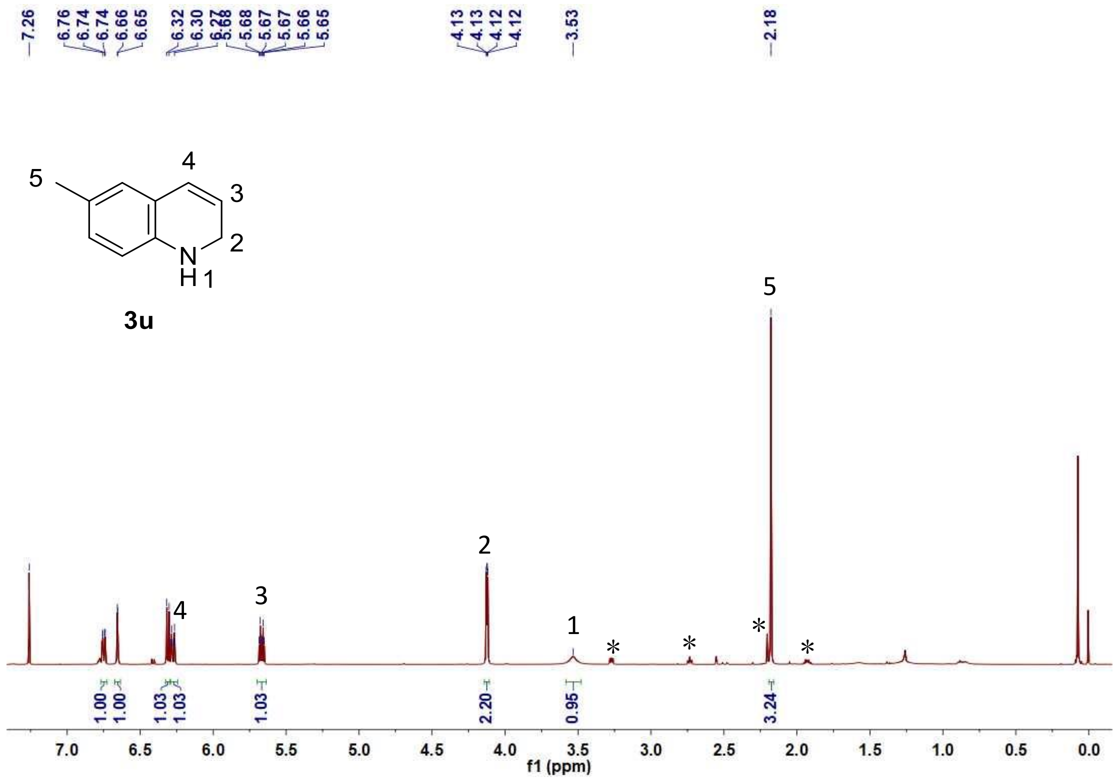


**Supplementary Figure 53. $^1$H NMR (500 MHz, $CDCl_3$) spectrum of 3u.** (* for tetrahydroquinoline product)

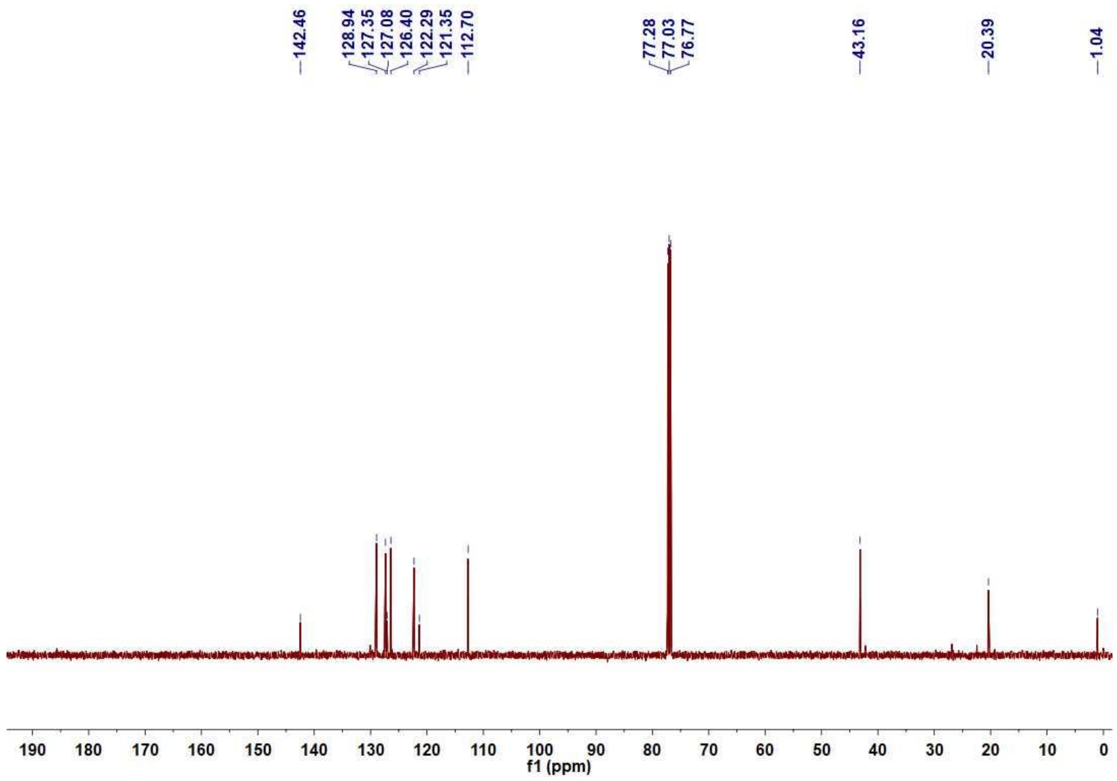


**Supplementary Figure 54.** $^{13}$**C NMR (126 MHz, CDCl$_3$) spectrum of 3u.**

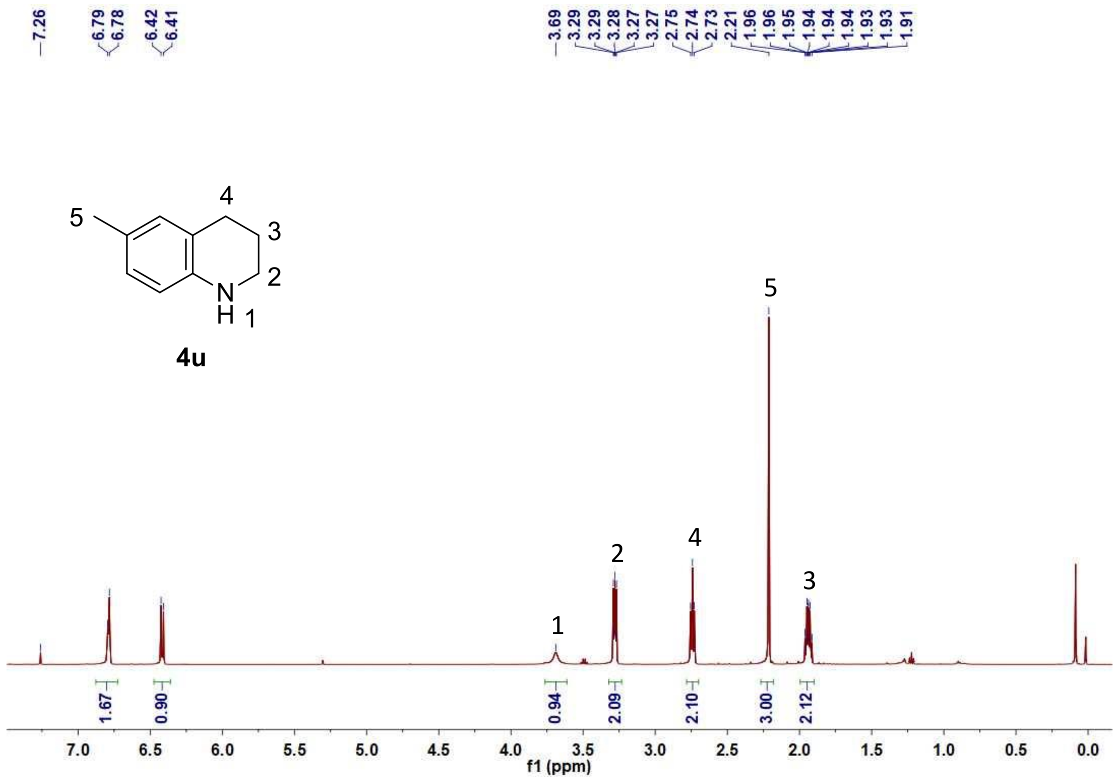


**Supplementary Figure 55. $^{1}$H NMR (500 MHz, $CDCl_3$) spectrum of 4u.**

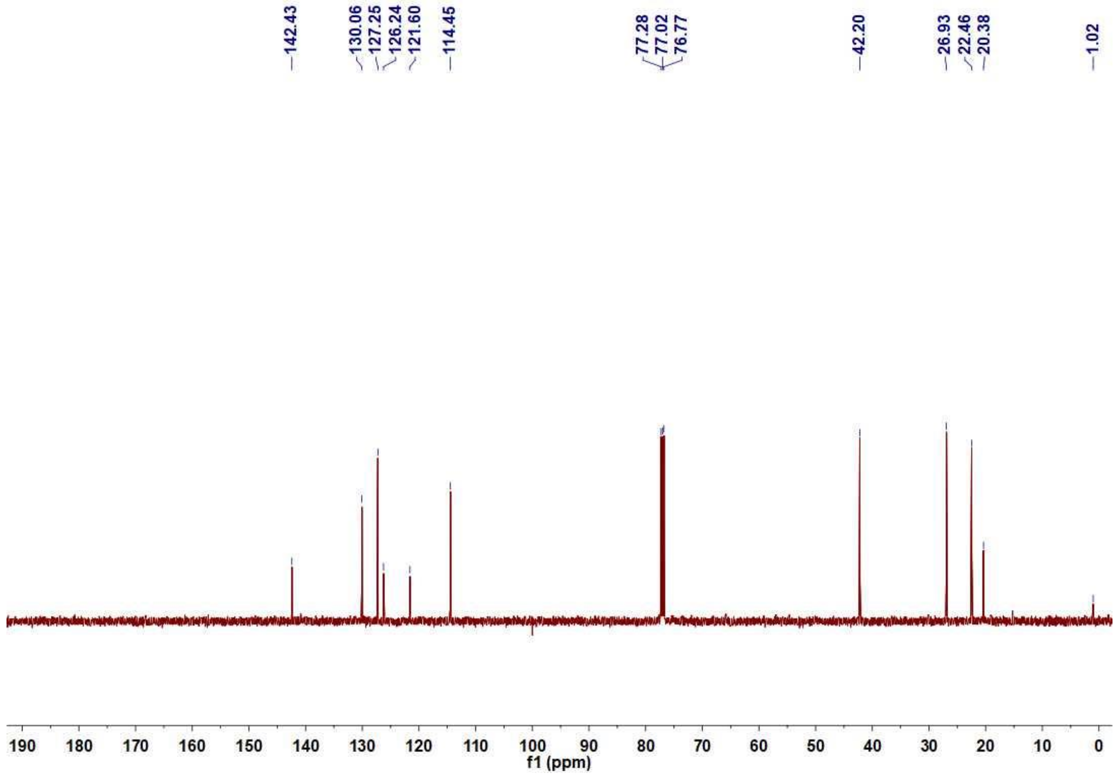


**Supplementary Figure 56. $^{13}$C NMR (126 MHz, $CDCl_3$) spectrum of 4u.**

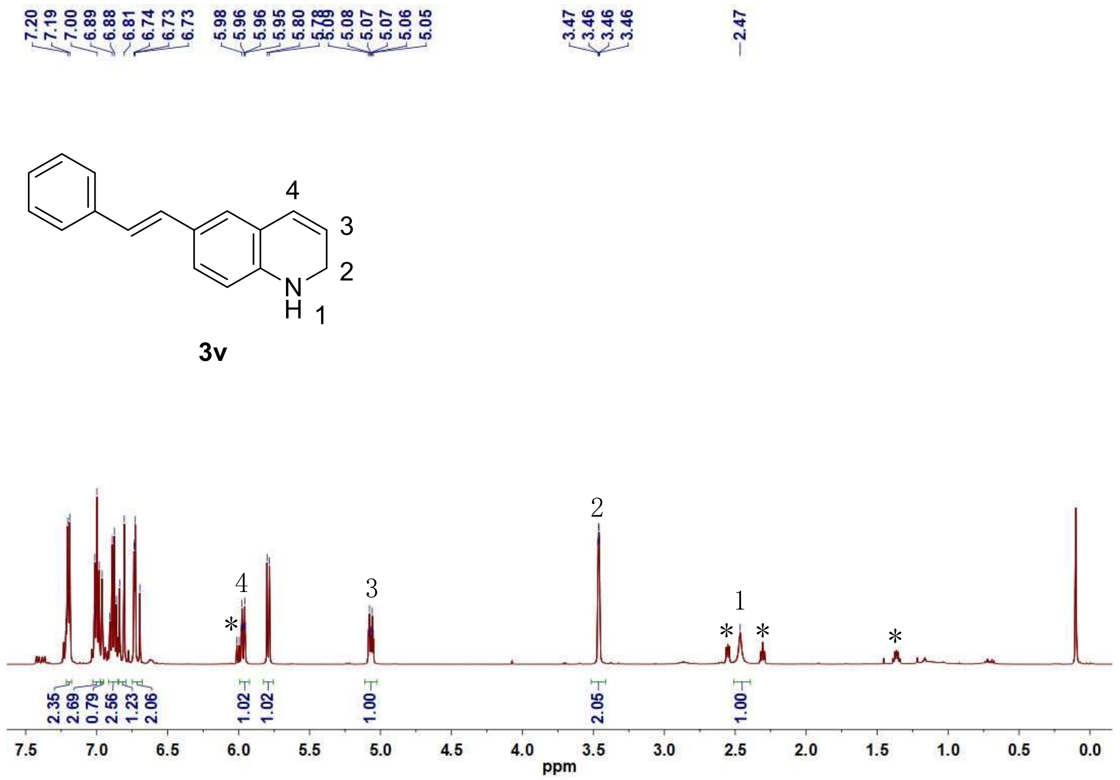

**Supplementary Figure 57. $^{1}$H NMR (500 MHz, $CDCl_3$) spectrum of 3v.** (* for tetrahydroquinoline product)

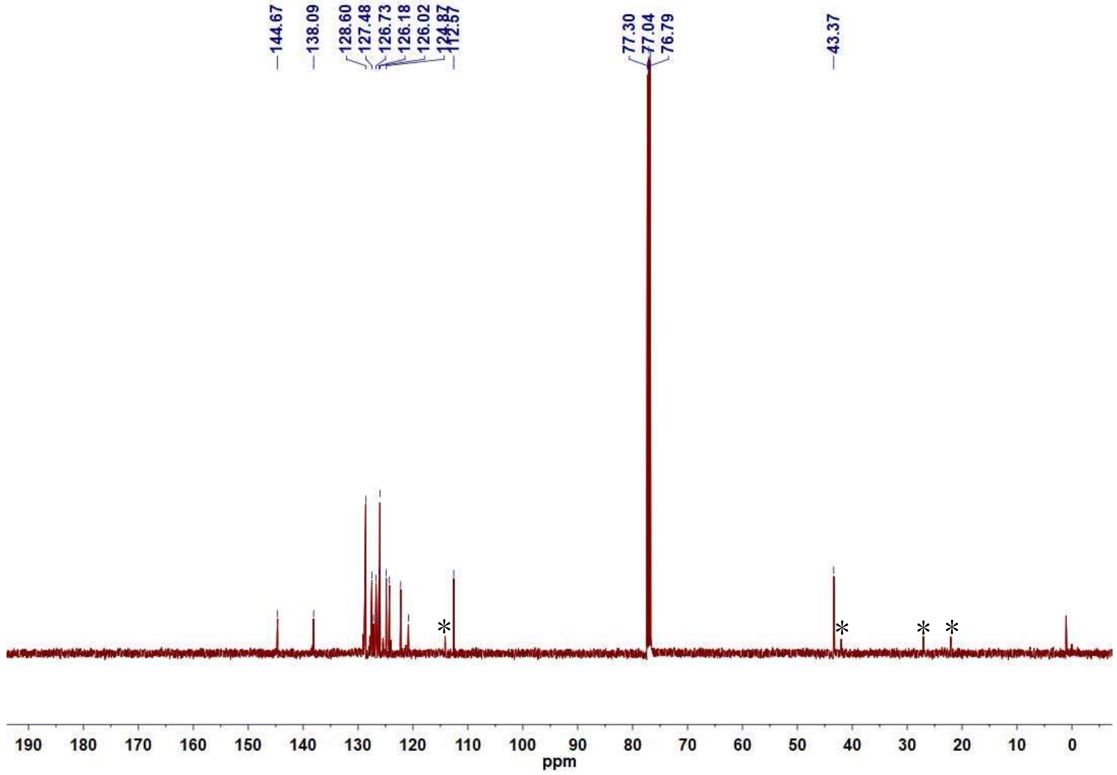


**Supplementary Figure 58. $^{13}C$ NMR (126 MHz, $CDCl_3$) spectrum of 3v.** (∗ for tetrahydroquinoline product)

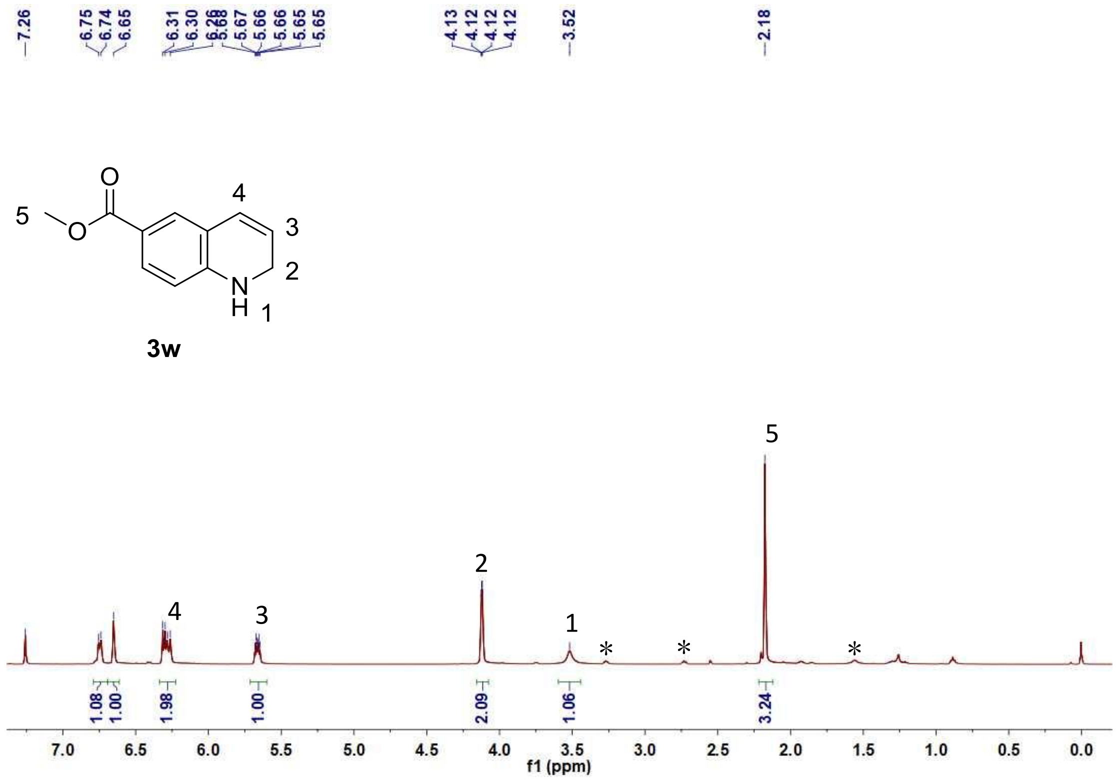


**Supplementary Figure 59. $^{1}$H NMR (500 MHz, $CDCl_3$) spectrum of 3w.** (* for tetrahydroquinoline product)

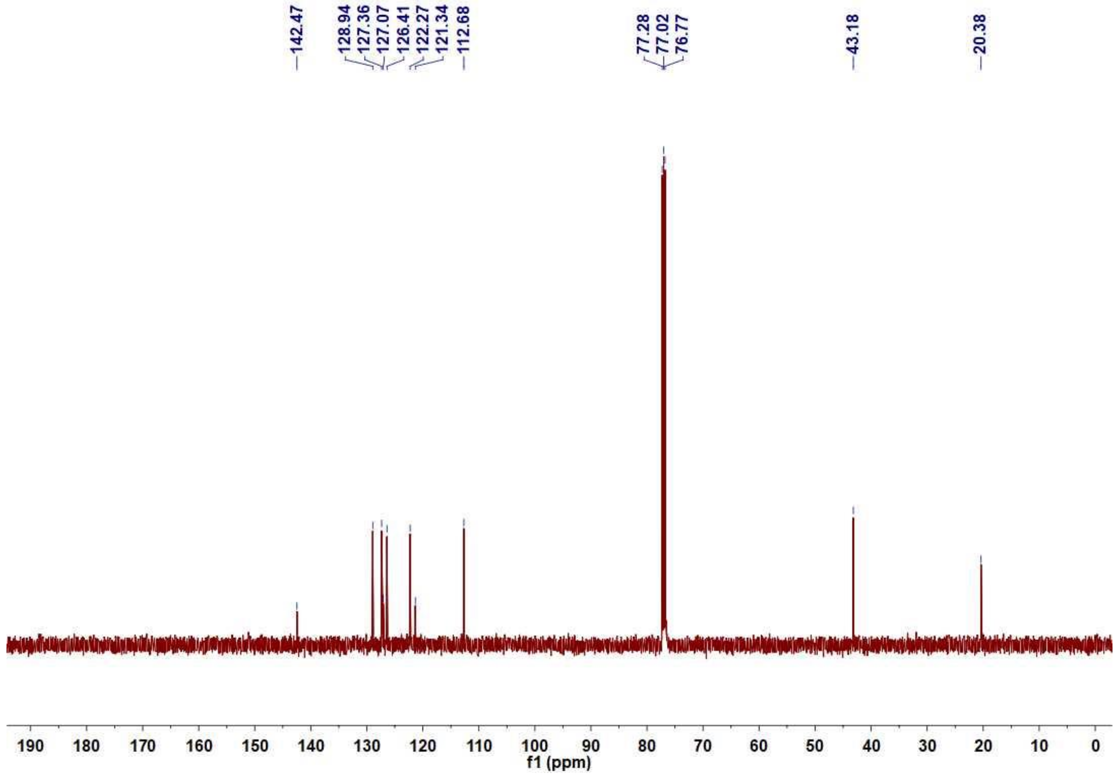


**Supplementary Figure 60. $^{13}$C NMR (126 MHz, $CDCl_3$) spectrum of 3w.**

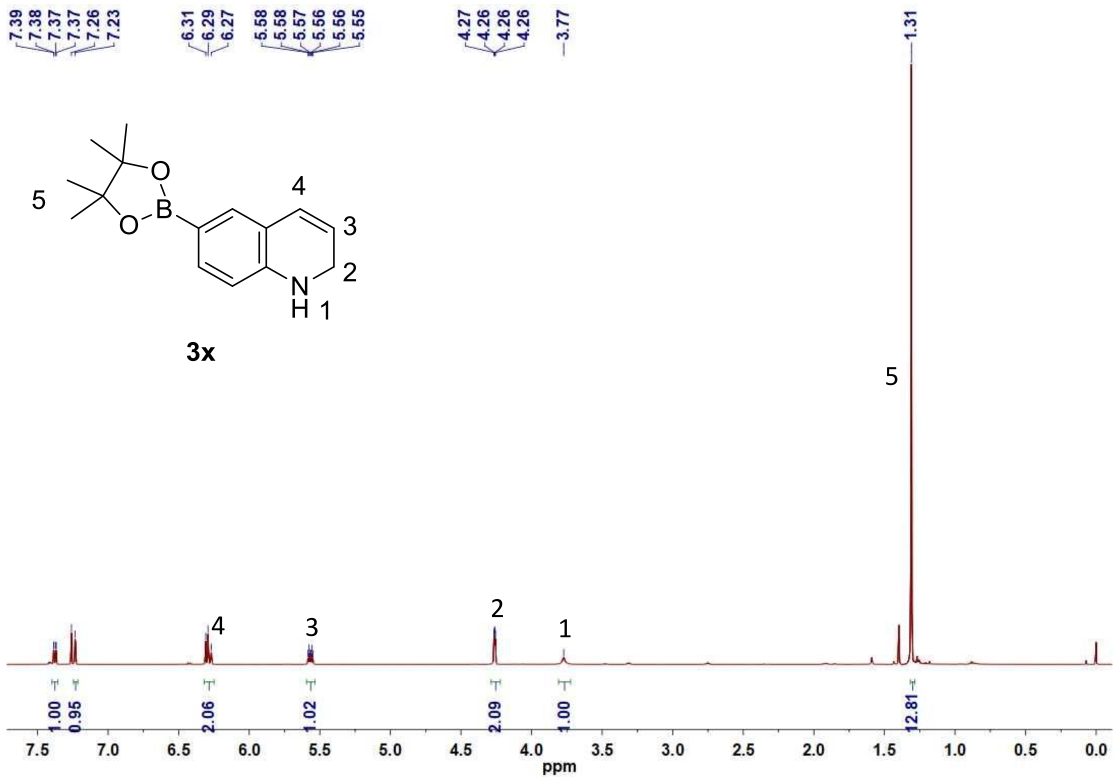


**Supplementary Figure 61.** **$^{1}$H NMR (500 MHz, $CDCl_3$) spectrum of 3x.**

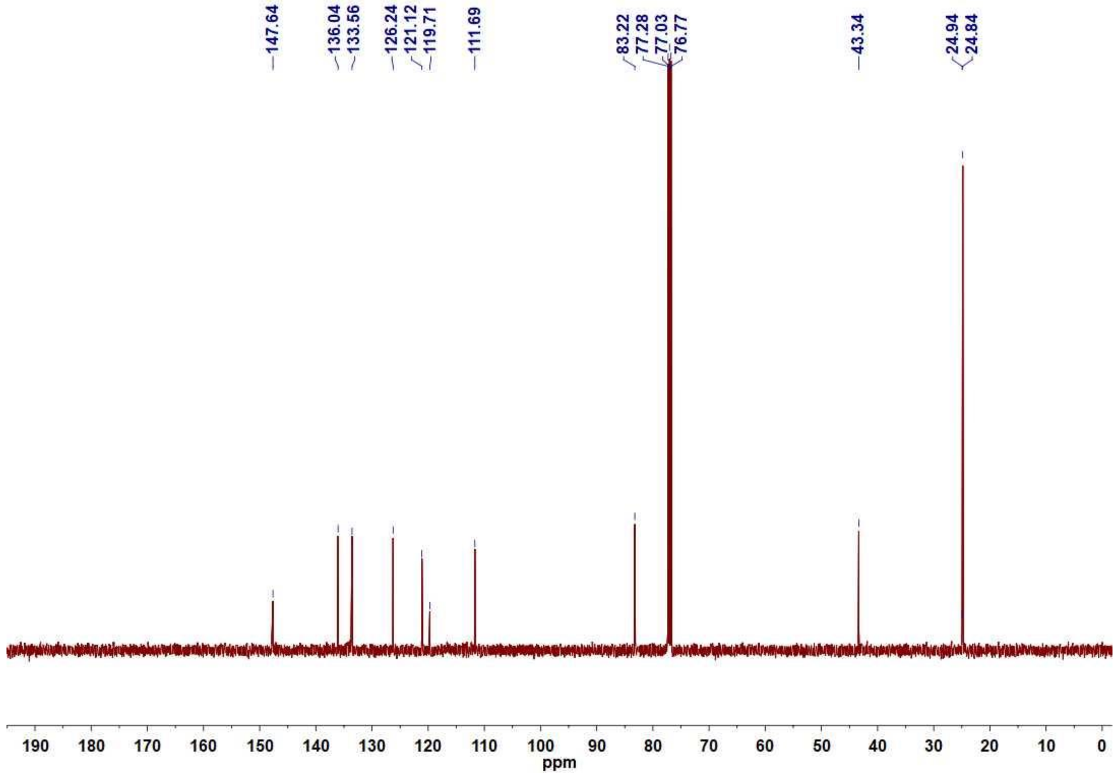


**Supplementary Figure 62.** $^{13}$**C NMR (126 MHz,** $CDCl_3$**) spectrum of 3x.**

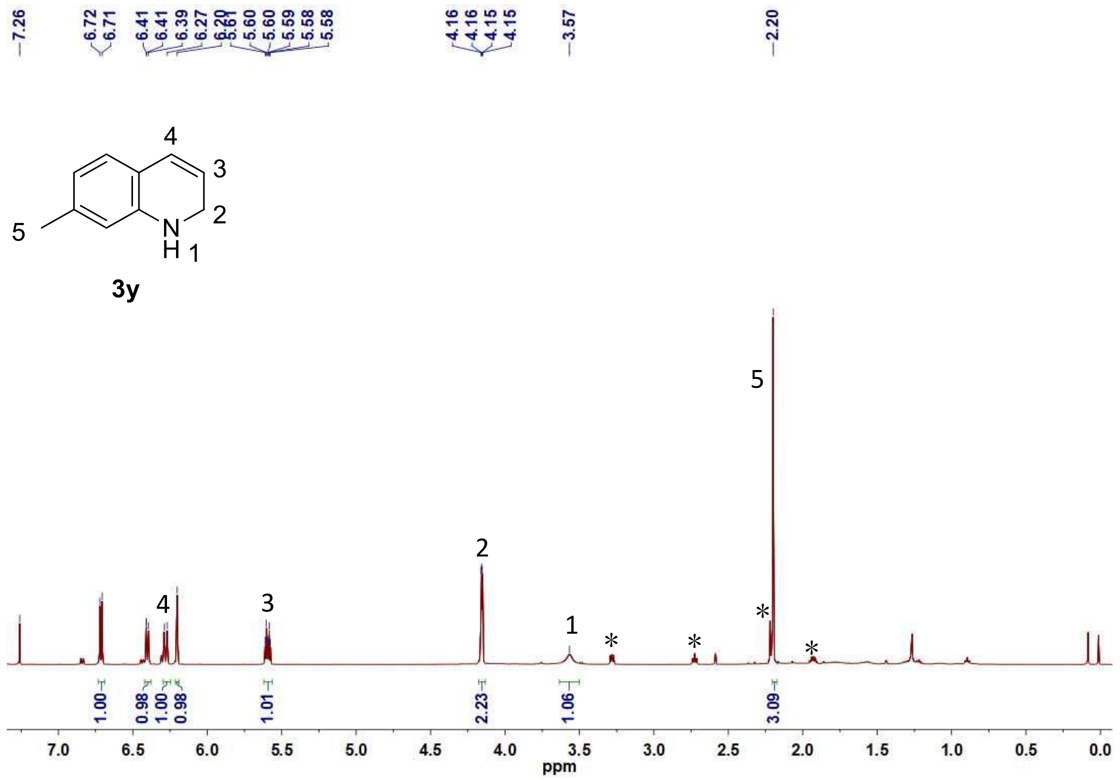


**Supplementary Figure 63. $^{1}$H NMR (500 MHz, $CDCl_3$) spectrum of 3y.** (* for tetrahydroquinoline product)

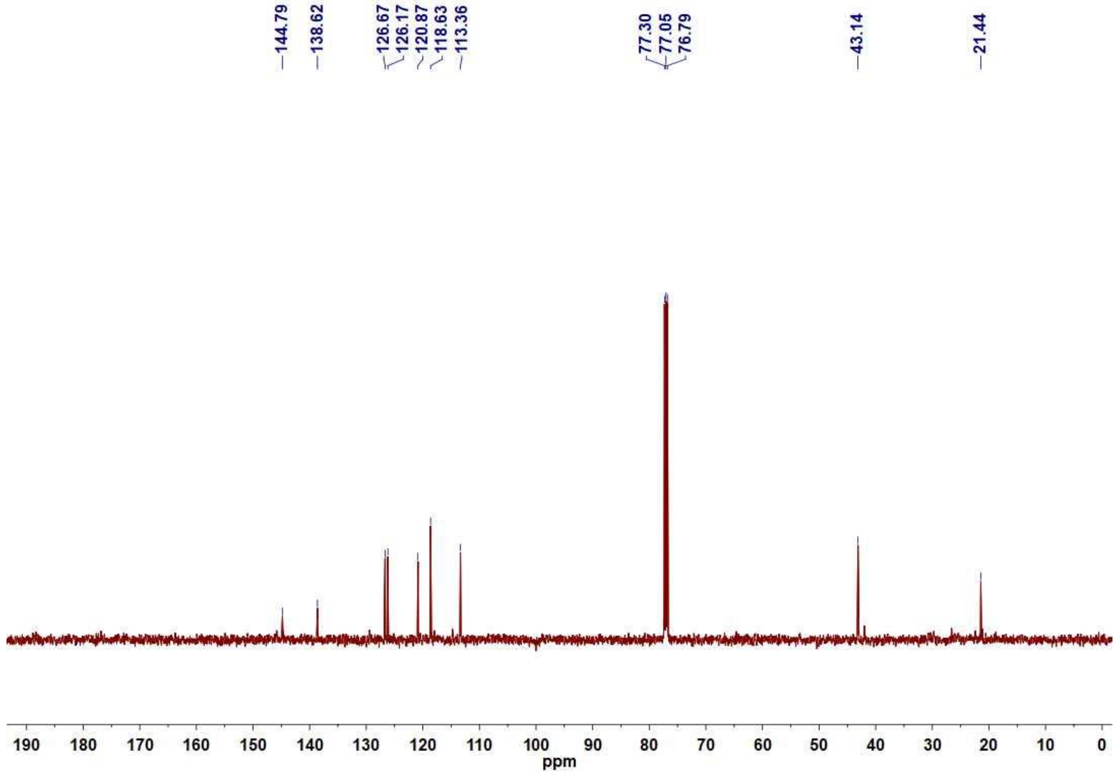


**Supplementary Figure 64.** $^{13}$**C NMR (126 MHz, $C_6D_6$) spectrum of 3y.**

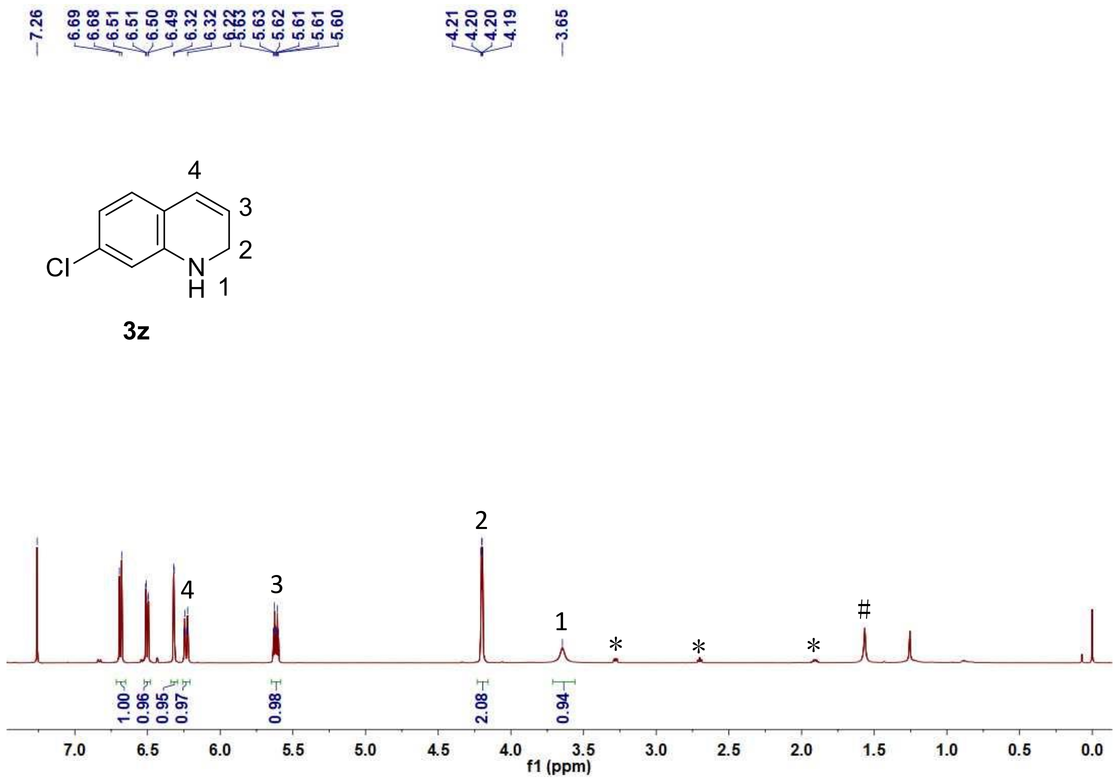


**Supplementary Figure 65. $^{1}$H NMR (500 MHz, $CDCl_3$) spectrum of 3z.** (* for tetrahydroquinoline product; # for water)

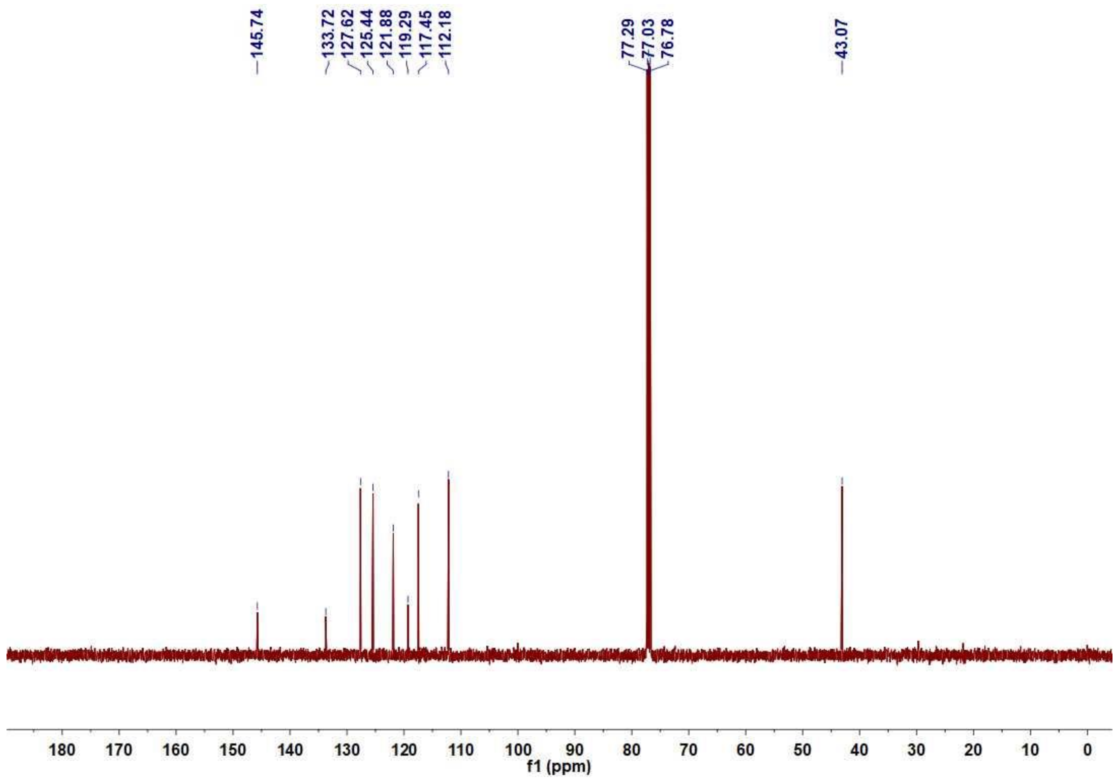


**Supplementary Figure 66.** $^{13}$**C NMR (126 MHz, CDCl$_3$) spectrum of 3z.**

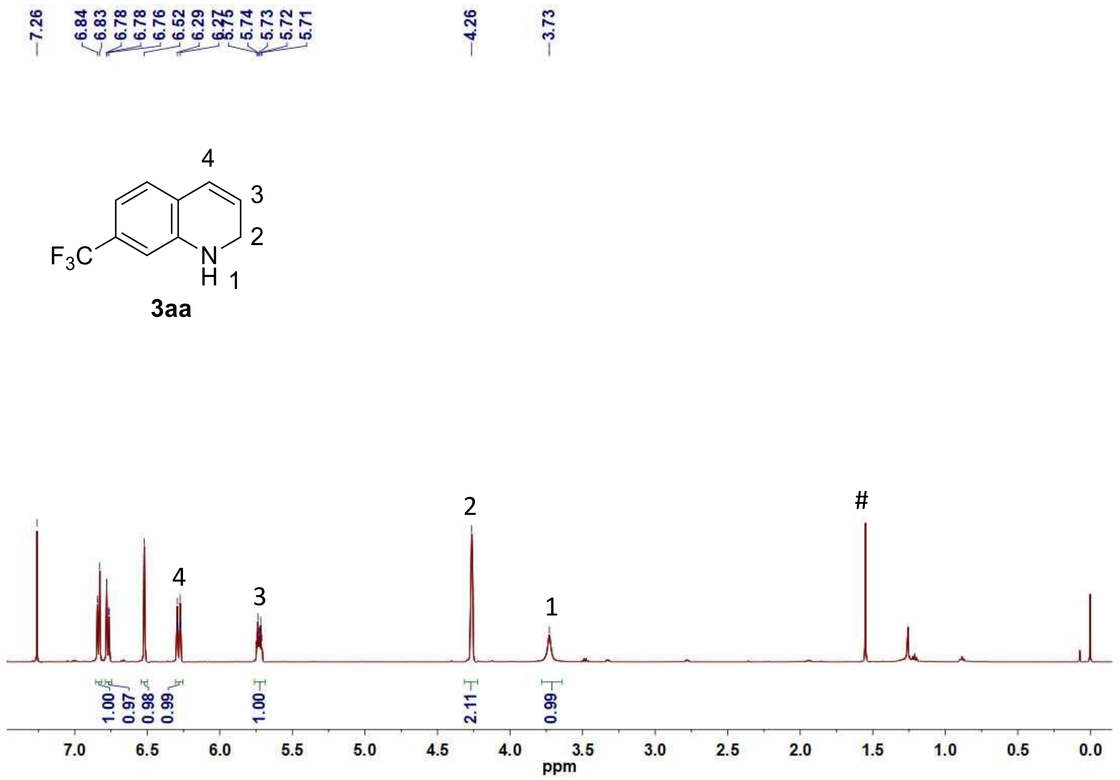


**Supplementary Figure 67. $^{1}$H NMR (500 MHz, CDCl$_3$) spectrum of 3aa.** (# for water)

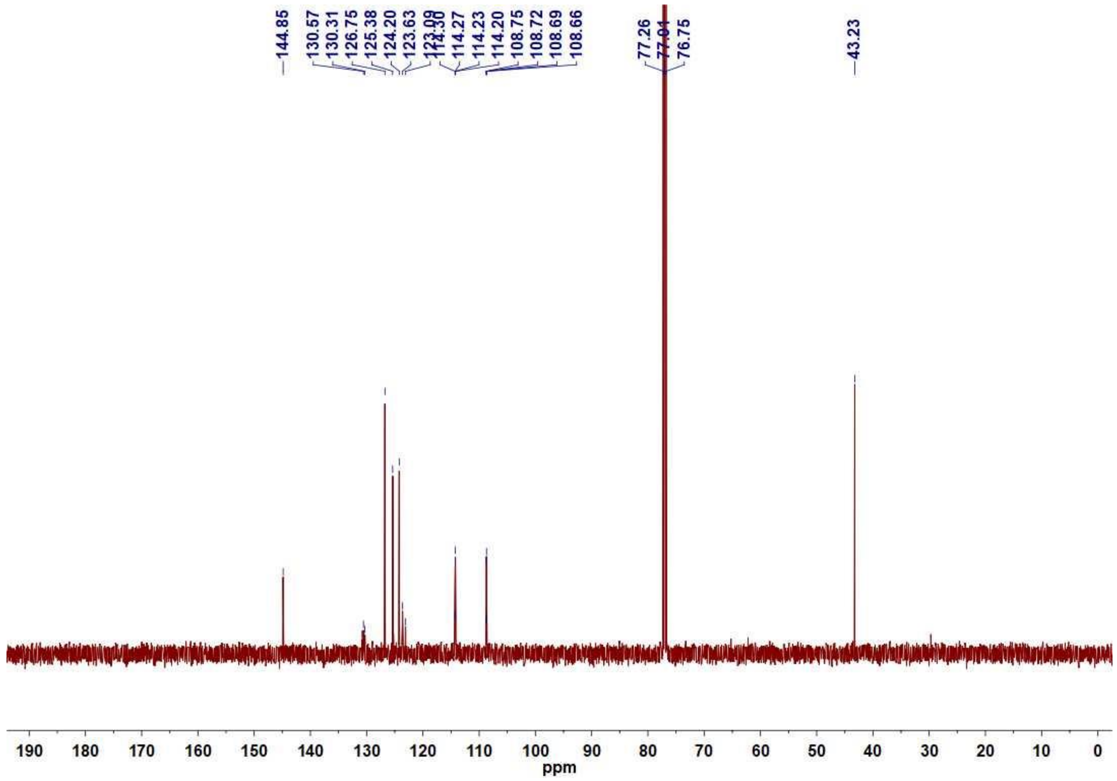


**Supplementary Figure 68.** $^{13}$**C NMR (126 MHz, CDCl$_3$) spectrum of 3aa.**

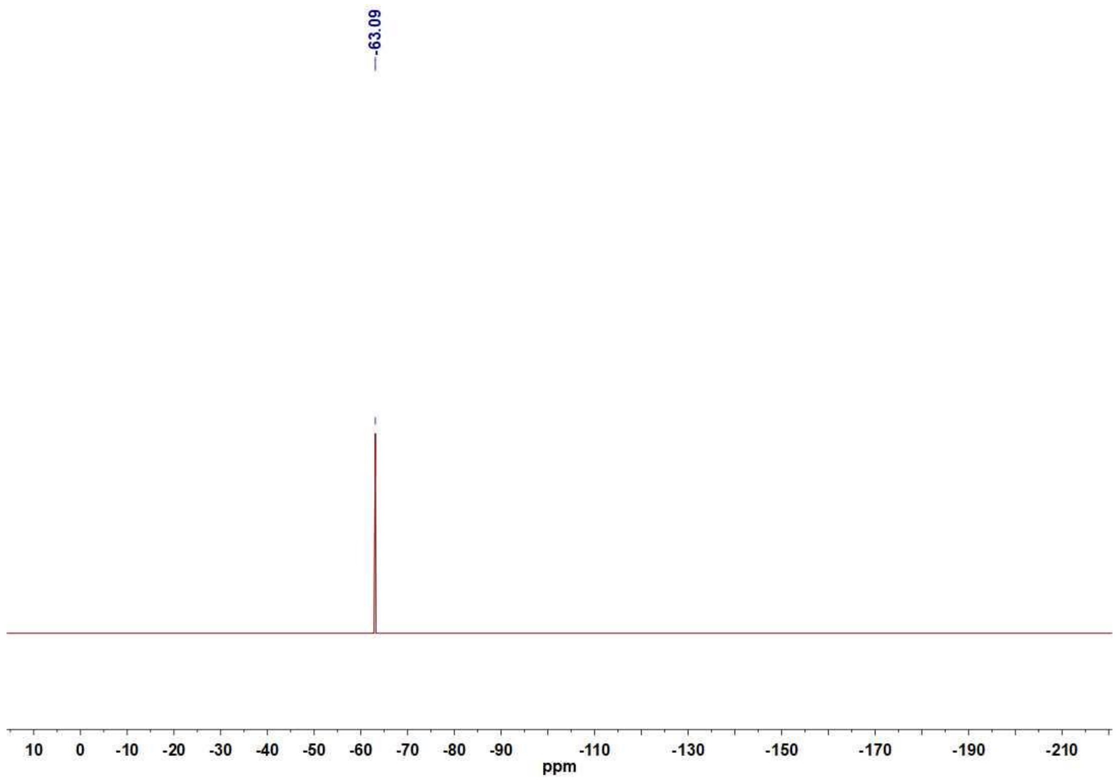


**Supplementary Figure 69. $^{19}$F NMR (471 MHz, $CDCl_3$) spectrum of 3aa.**

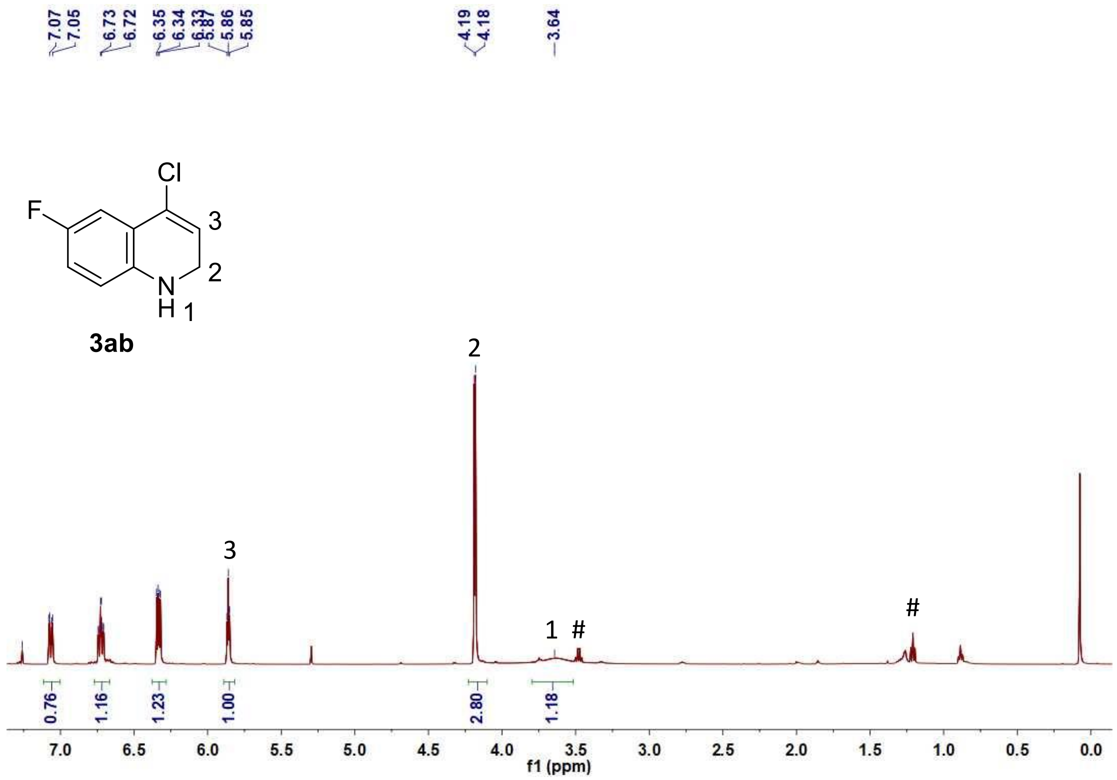


**Supplementary Figure 70. $^{1}$H NMR (500 MHz, $CDCl_3$) spectrum of 3ab.** (# for $Et_2O$)

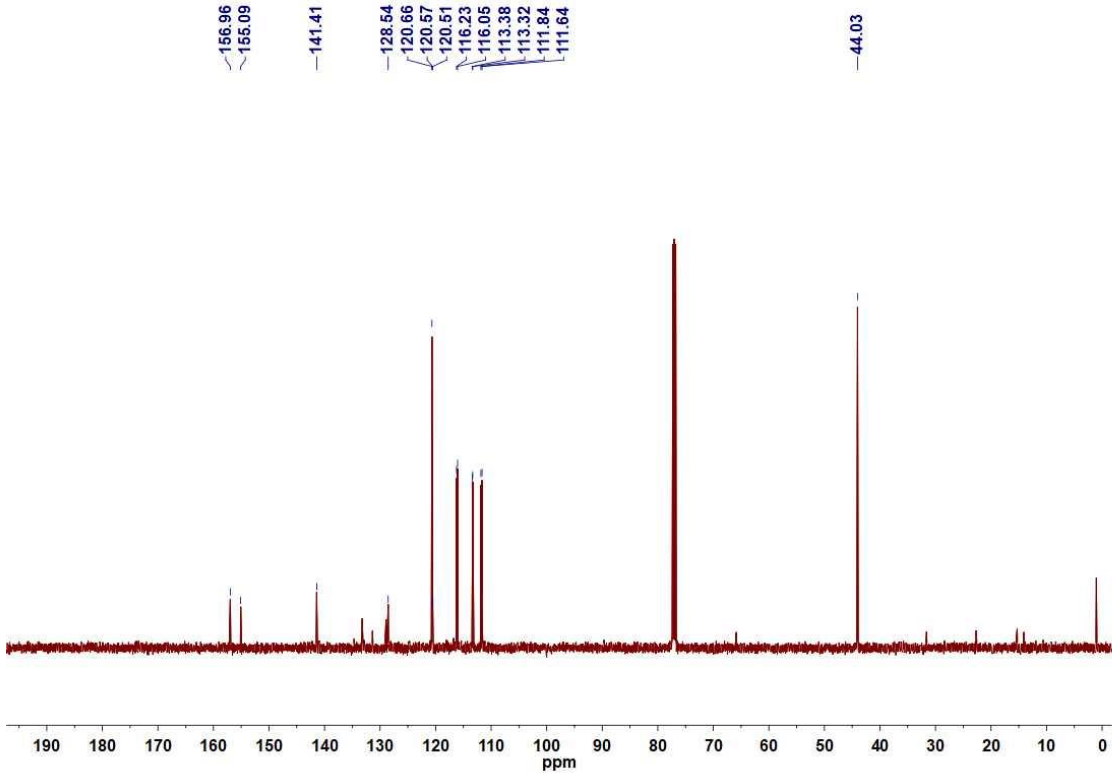


**Supplementary Figure 71. $^{13}$C NMR (126 MHz, $CDCl_3$) spectrum of 3ab.**

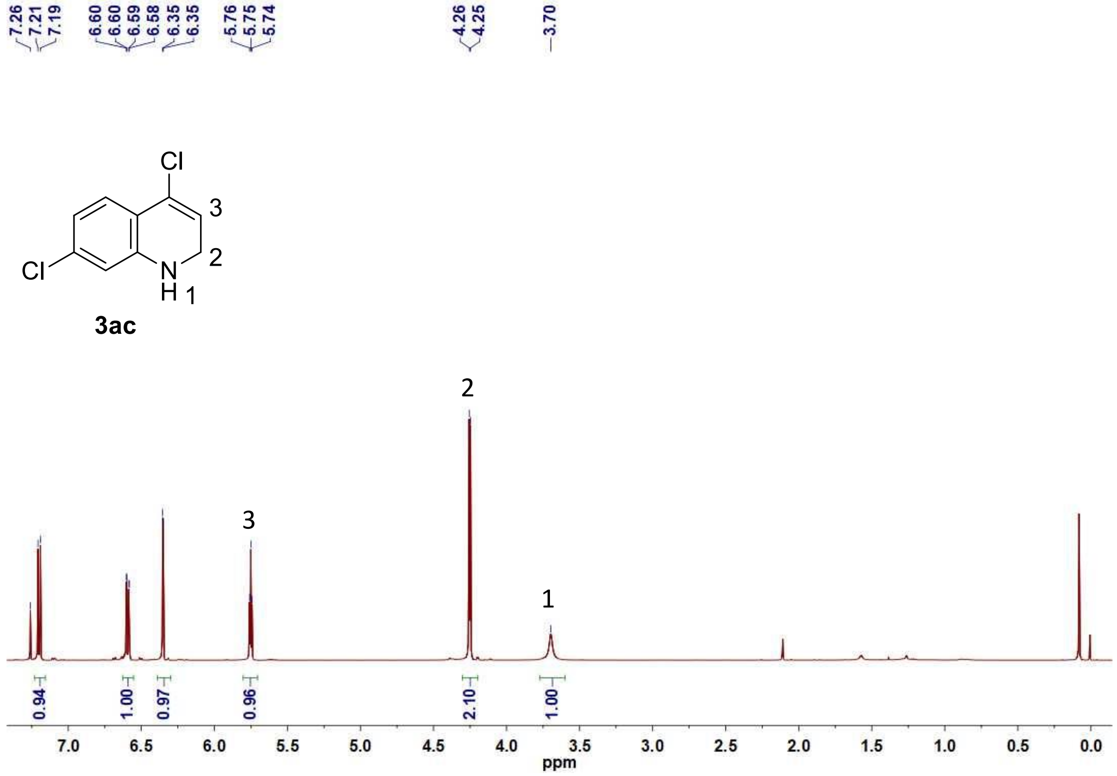


**Supplementary Figure 72. $^1$H NMR (500 MHz, $CDCl_3$) spectrum of 3ac.**

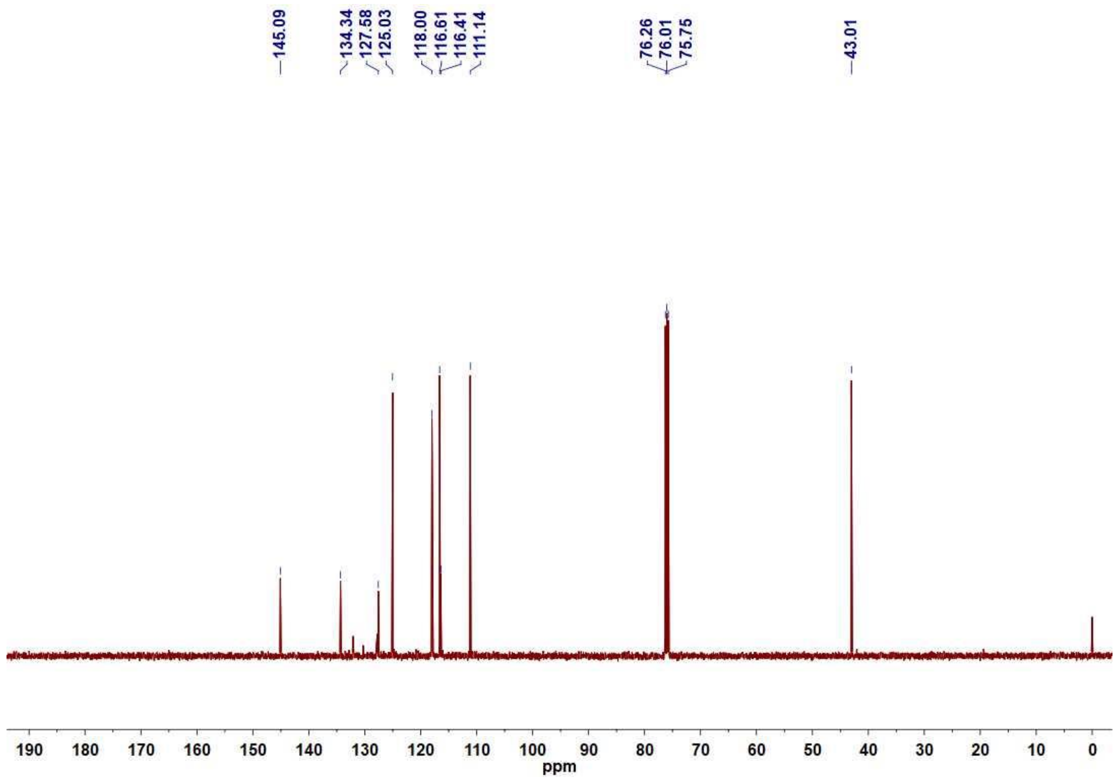


**Supplementary Figure 73. $^{13}$C NMR (126 MHz, $CDCl_3$) spectrum of 3ac.**

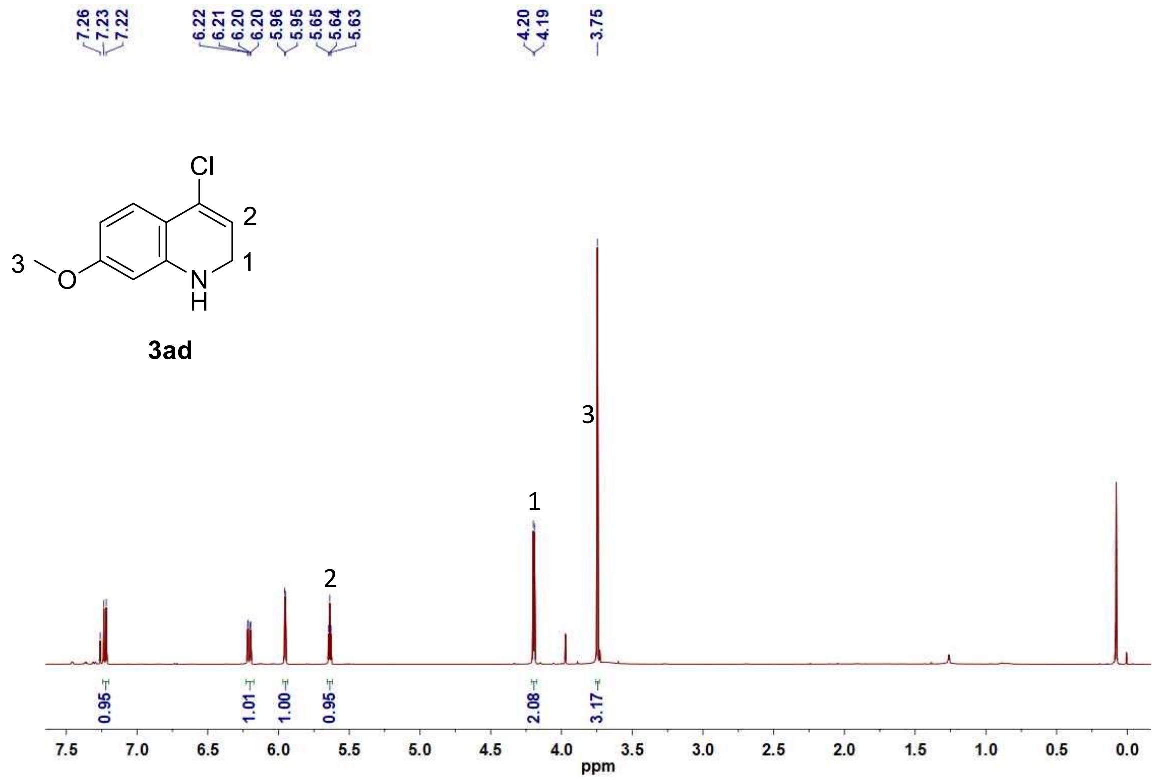


**Supplementary Figure 74. $^{1}$H NMR (500 MHz, $CDCl_3$) spectrum of 3ad.**

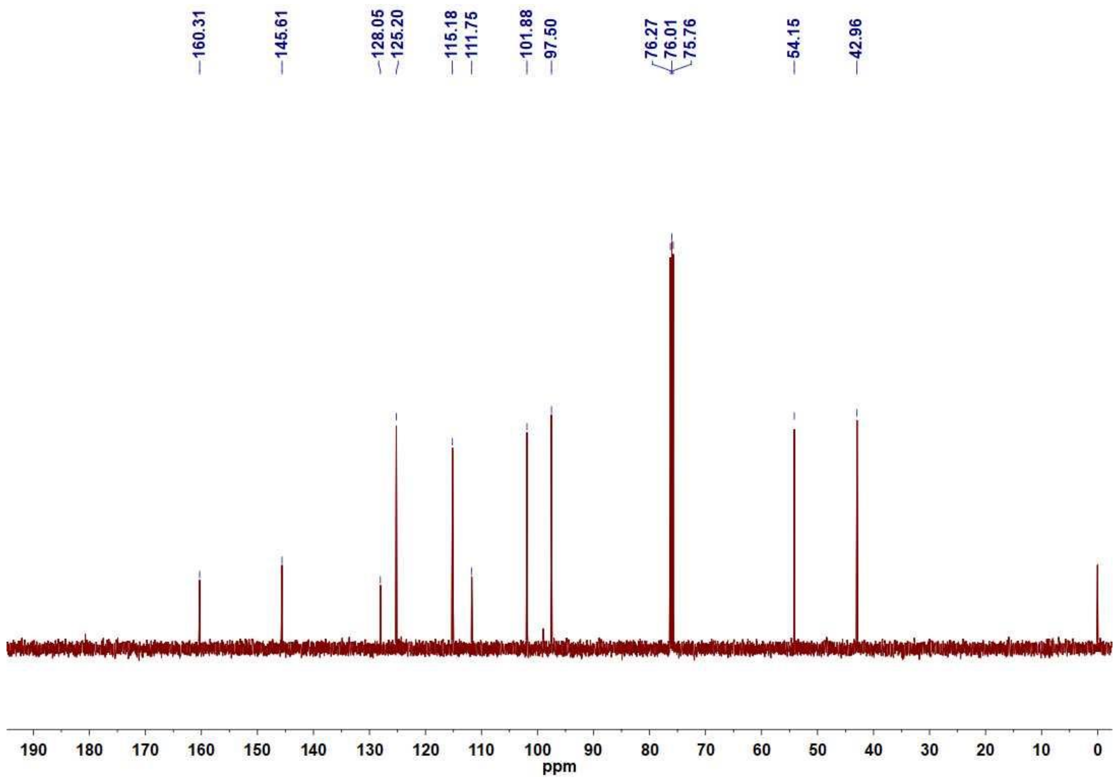


**Supplementary Figure 75. $^{13}$C NMR (126 MHz, $CDCl_3$) spectrum of 3ad.**

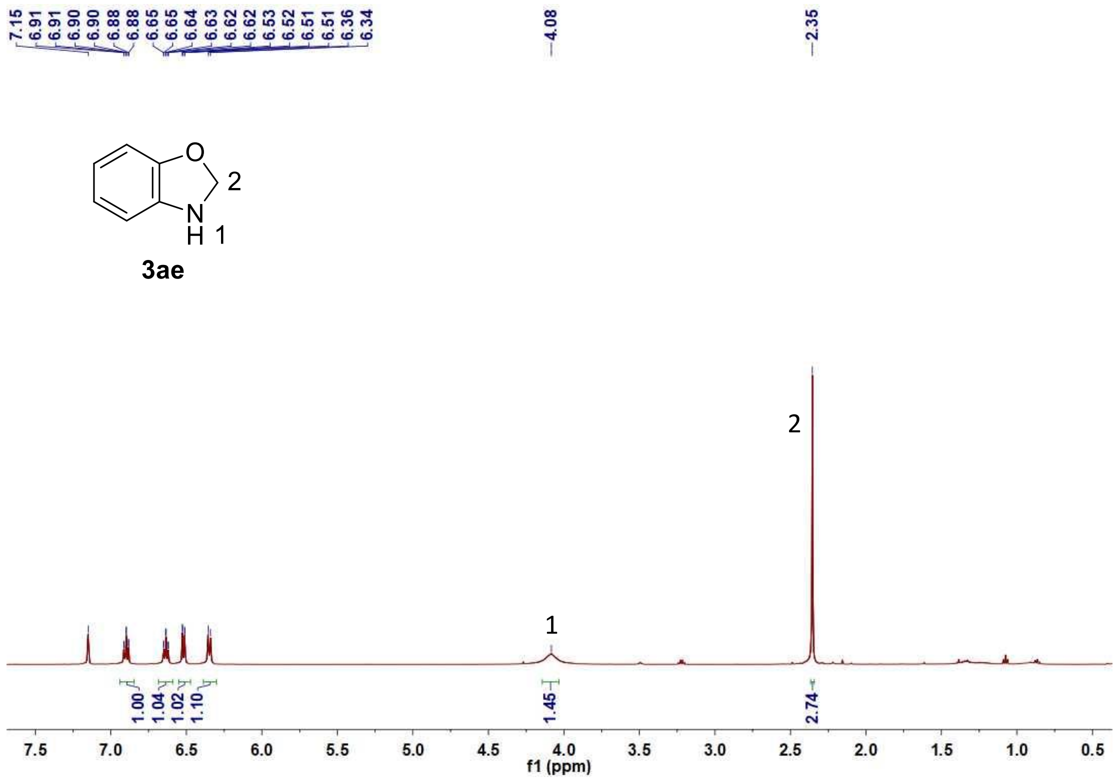


**Supplementary Figure 76. $^{1}H$ NMR (500 MHz, $C_6D_6$) spectrum of 3ae.**

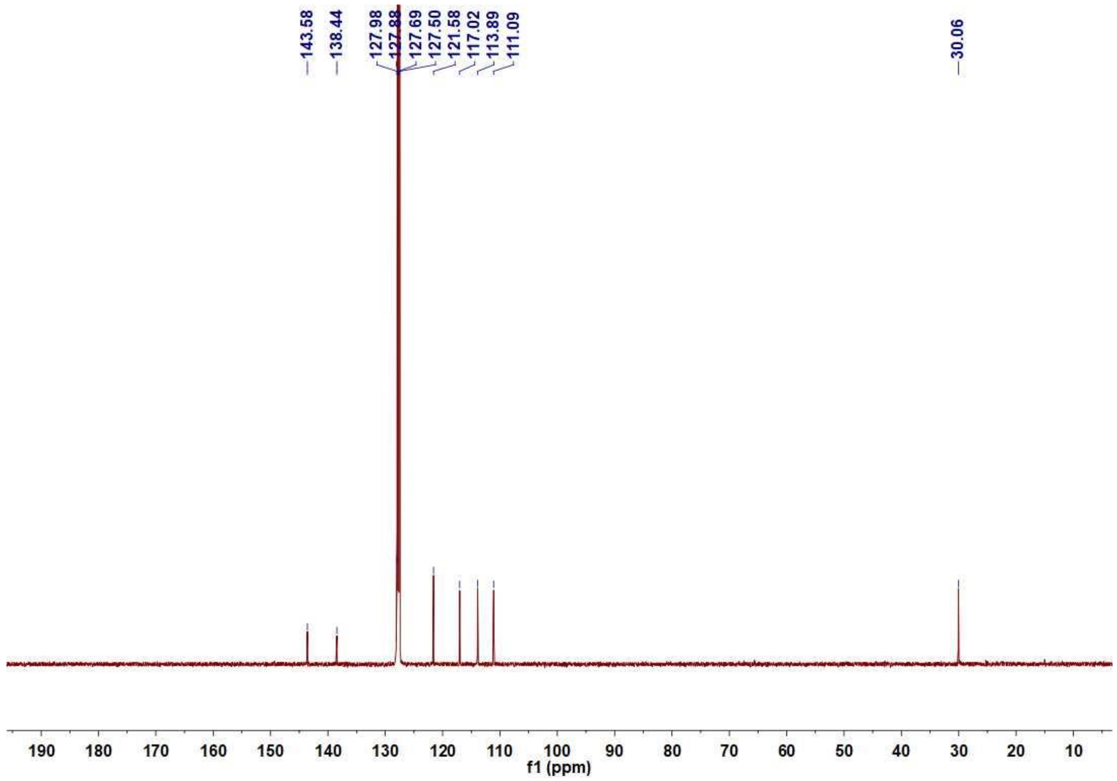


**Supplementary Figure 77. $^{13}C$ NMR (126 MHz, $C_6D_6$) spectrum of 3ae.**

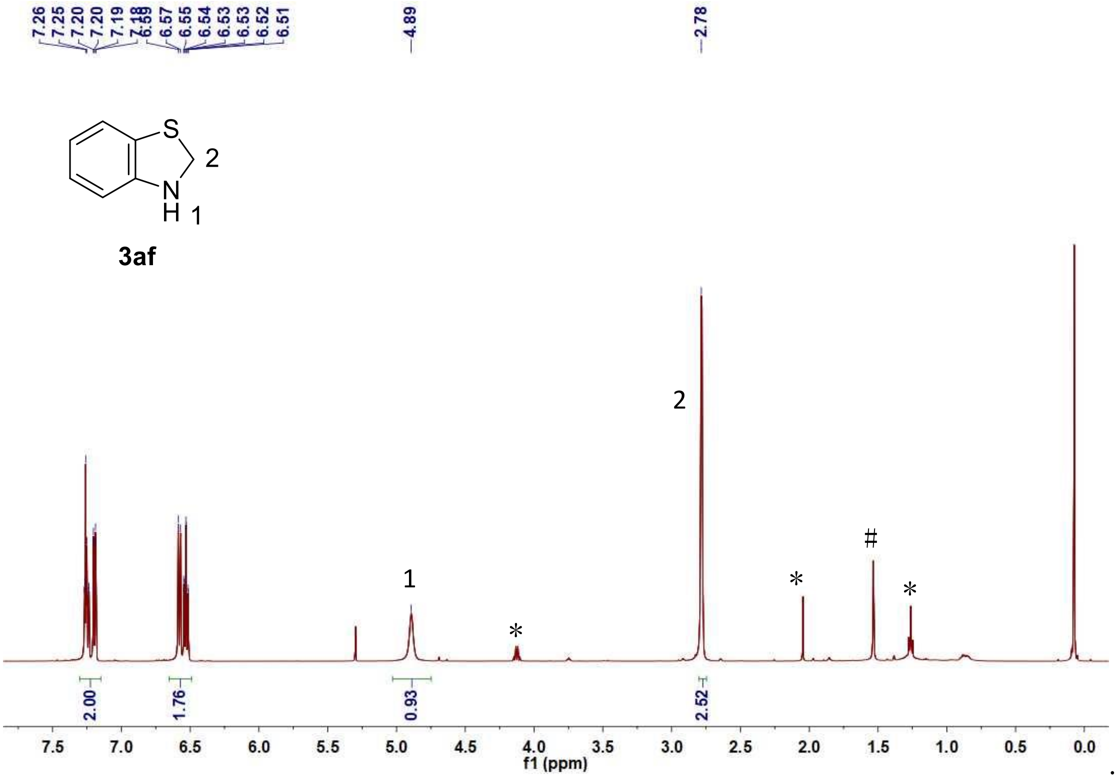


**Supplementary Figure 78. $^1$H NMR (500 MHz, $CDCl_3$) spectrum of 3af.** (* for ethyl acetate; # for water)

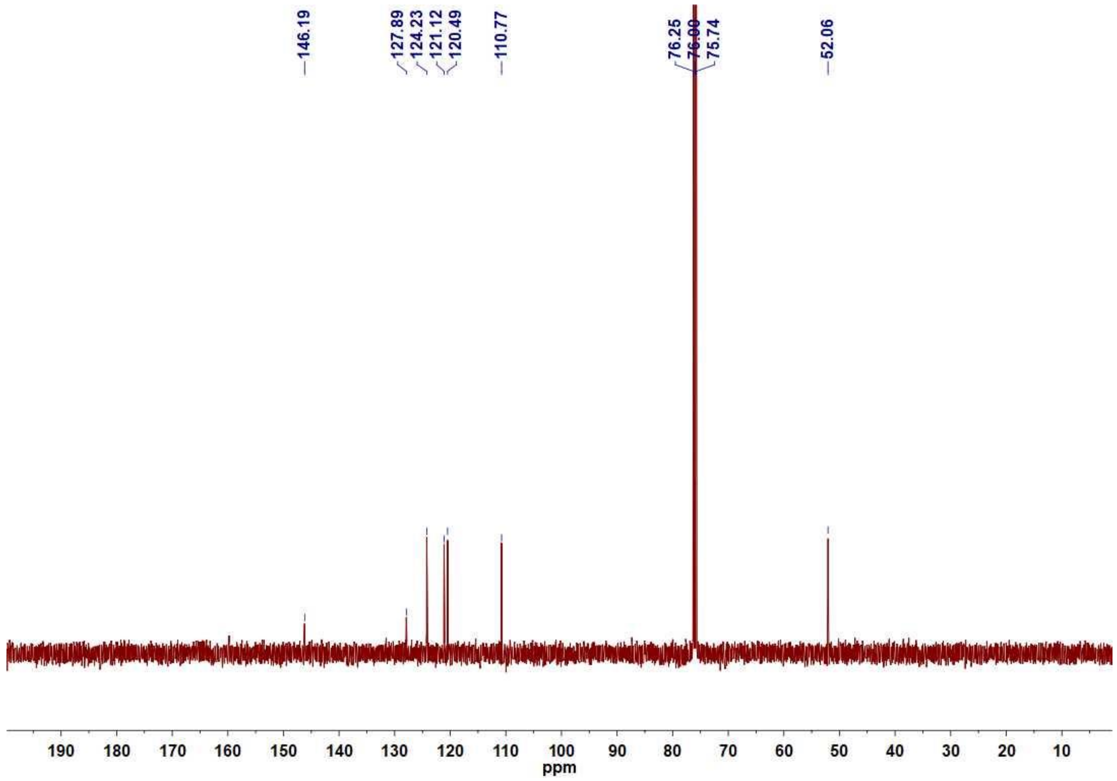

**Supplementary Figure 79. $^{13}C$ NMR (126 MHz, $CDCl_3$) spectrum of 3af.**

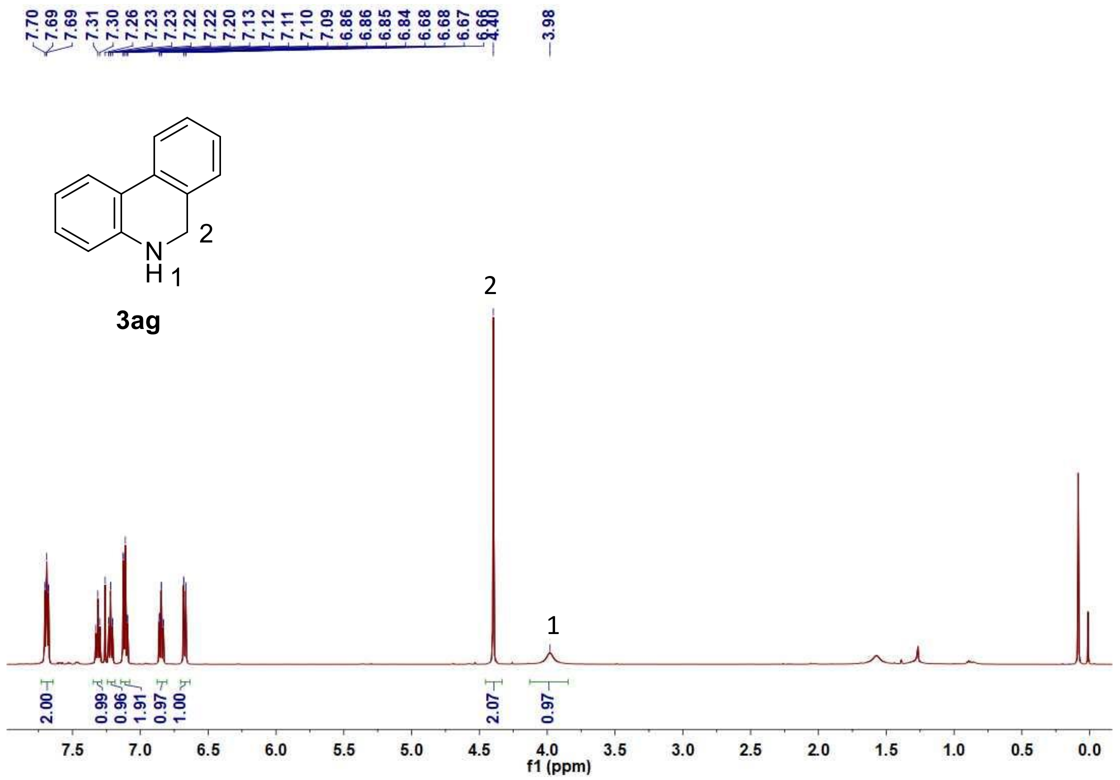


**Supplementary Figure 80. $^{1}$H NMR (500 MHz, $CDCl_3$) spectrum of 3ag.**

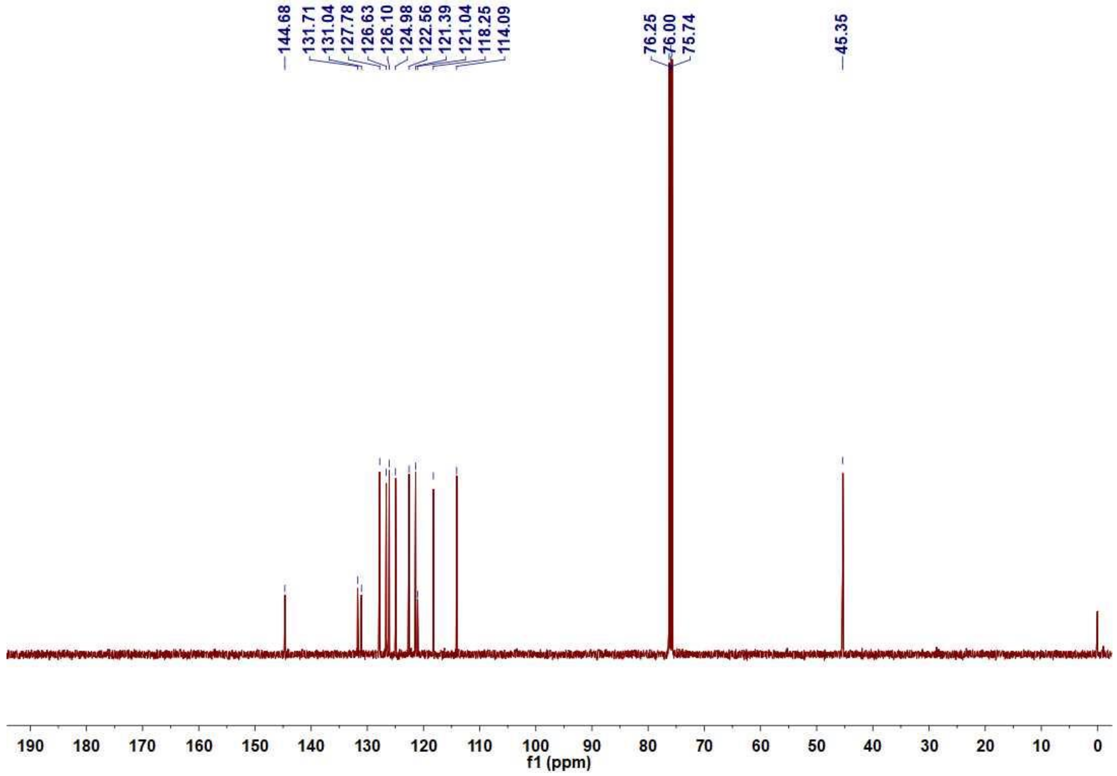


**Supplementary Figure 81. $^{13}C$ NMR (126 MHz, $CDCl_3$) spectrum of 3ag.**

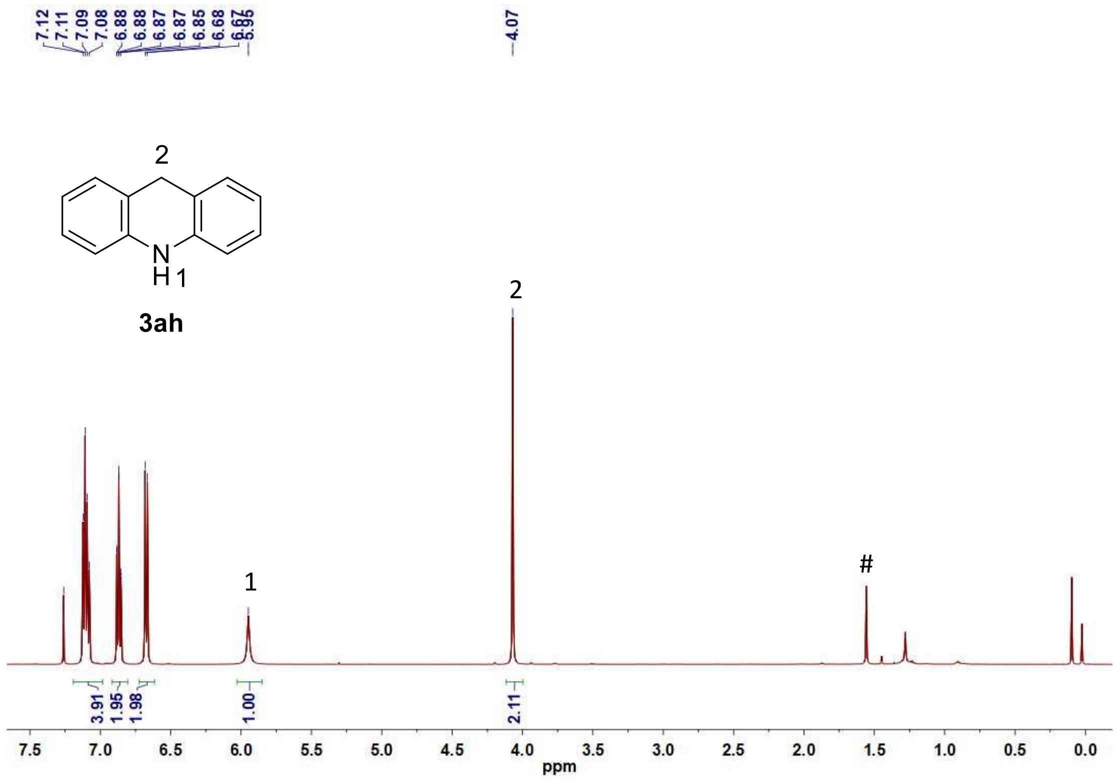


**Supplementary Figure 82. $^{1}$H NMR (500 MHz, CDCl$_3$) spectrum of 3ah.** (# for water)

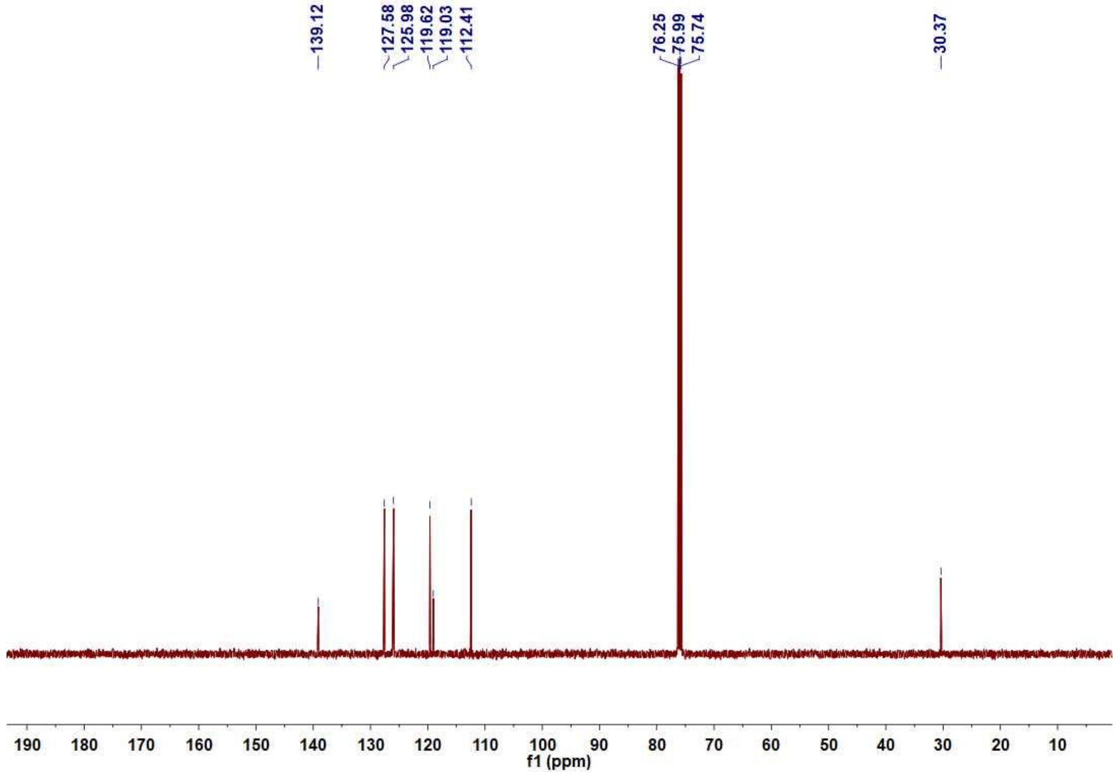


**Supplementary Figure 83. $^{13}$C NMR (126 MHz, $CDCl_3$) spectrum of 3ah.**

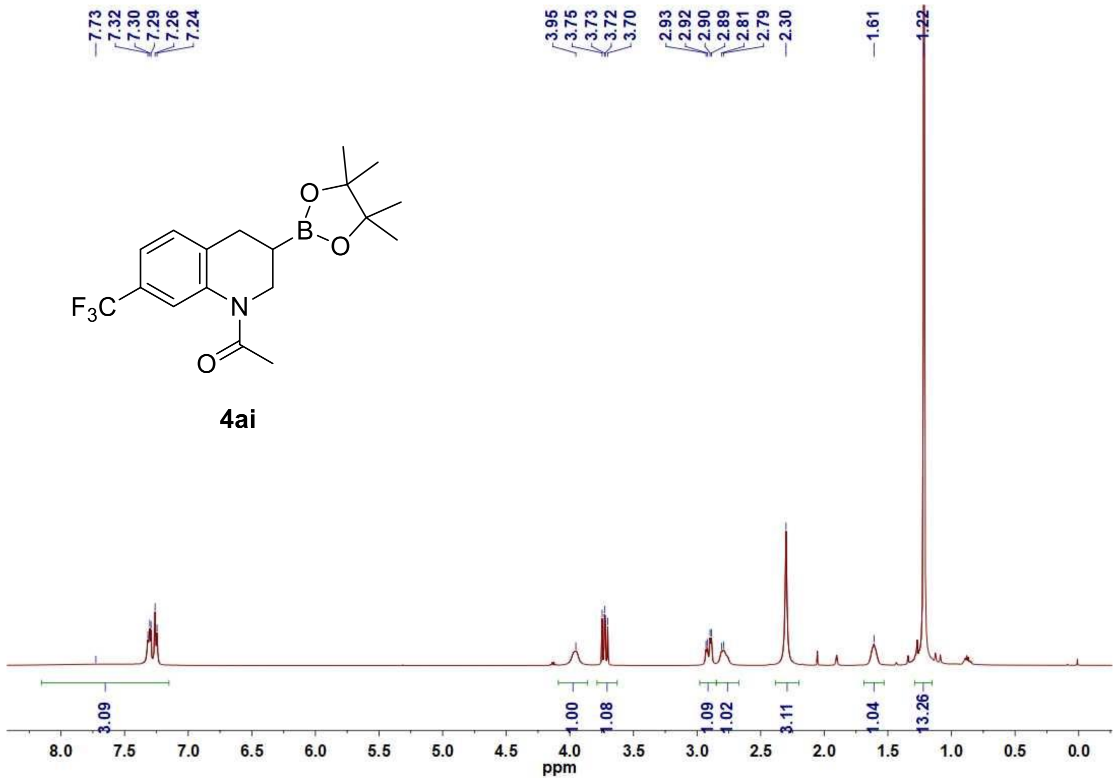


**Supplementary Figure 84. $^{1}$H NMR (500 MHz, $CDCl_3$) spectrum of 6.**

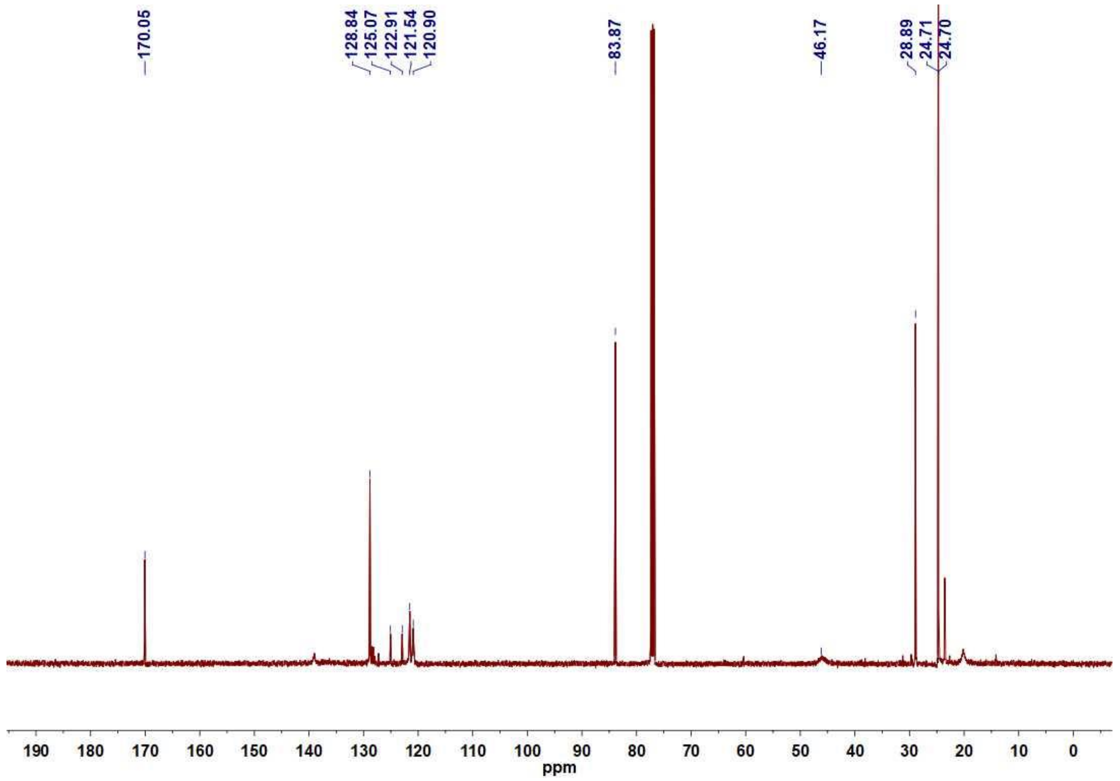


**Supplementary Figure 85. $^{13}$C NMR (126 MHz, $CDCl_3$) spectrum of 6.**

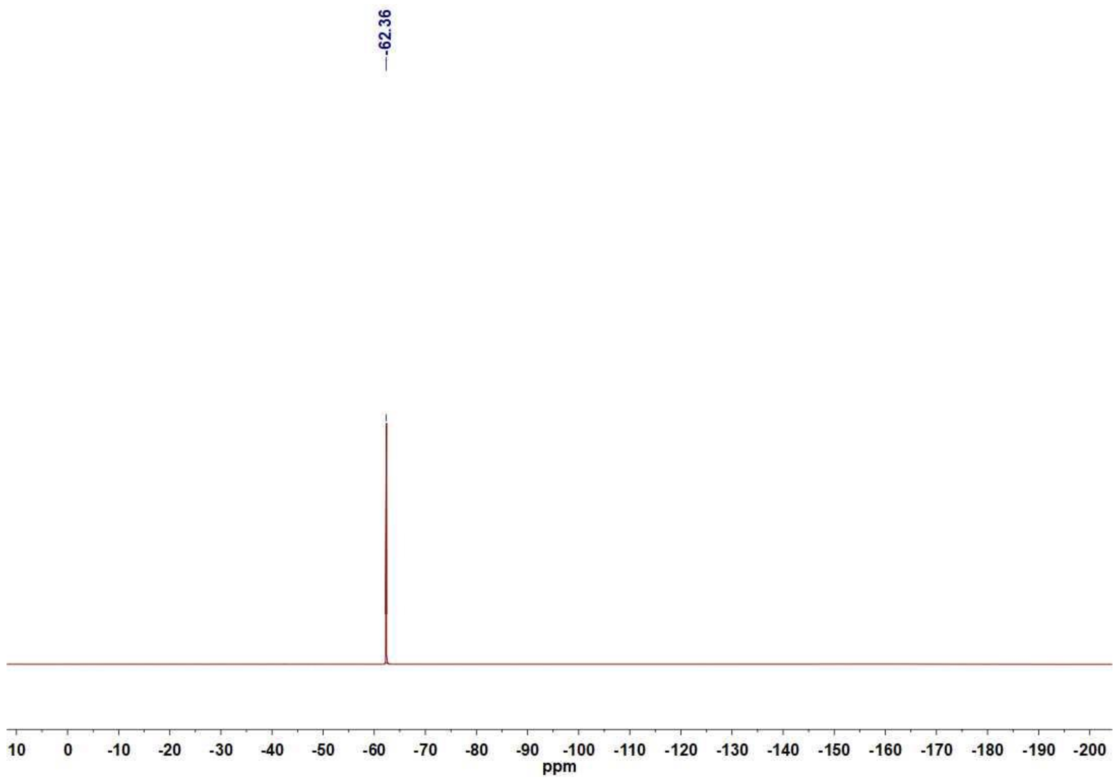


**Supplementary Figure 86. $^{19}F$ NMR (471 MHz, $CDCl_3$) spectrum of 6.**

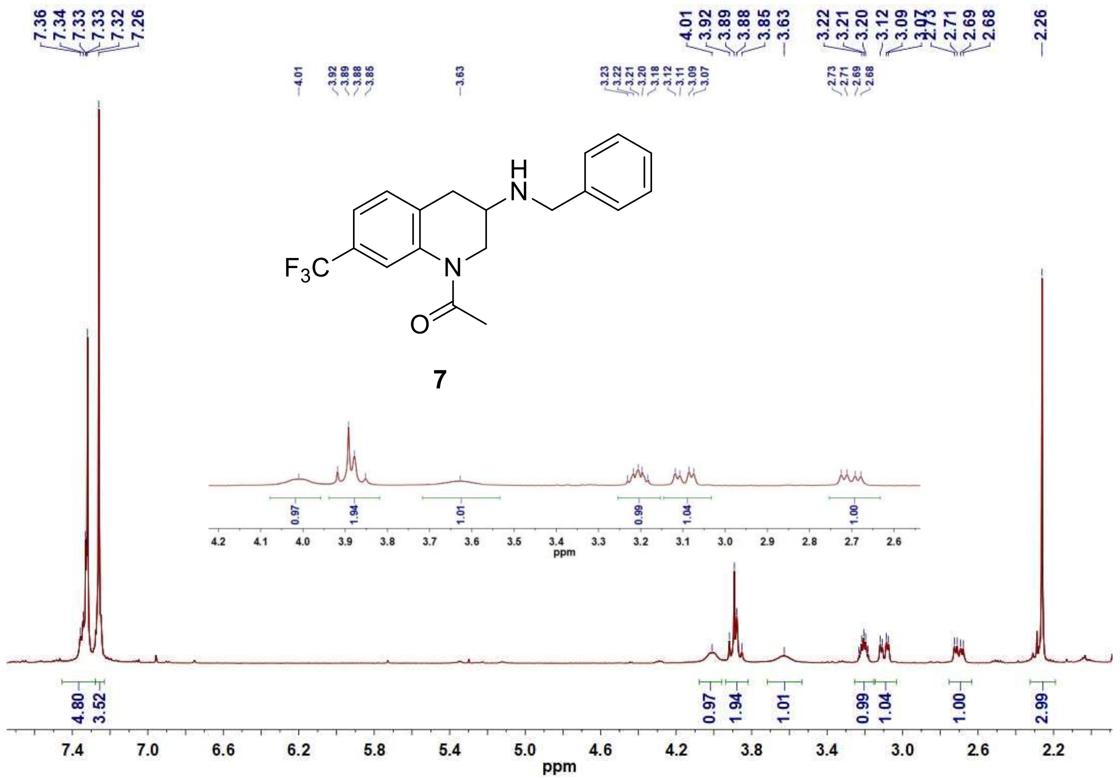

**Supplementary Figure 87. $^{1}$H NMR (500 MHz, CDCl3) spectrum of 7.**

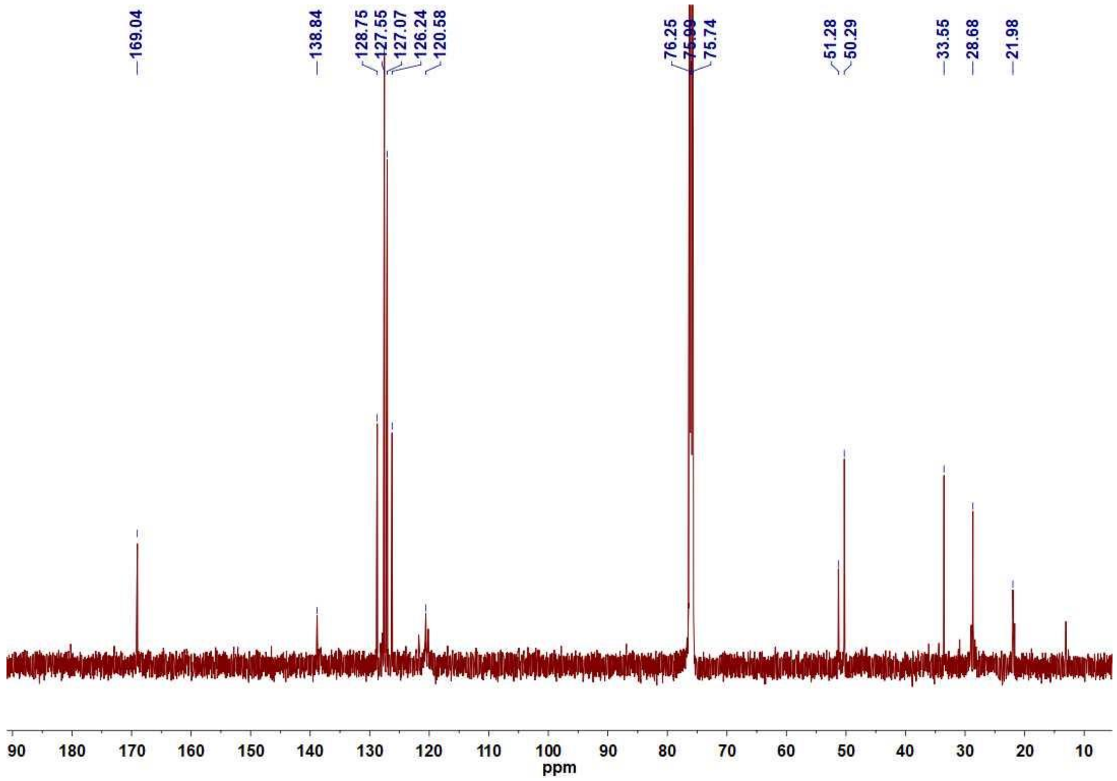


**Supplementary Figure 88.** **$^{13}$C NMR (126 MHz, $CDCl_3$) spectrum of 7.**

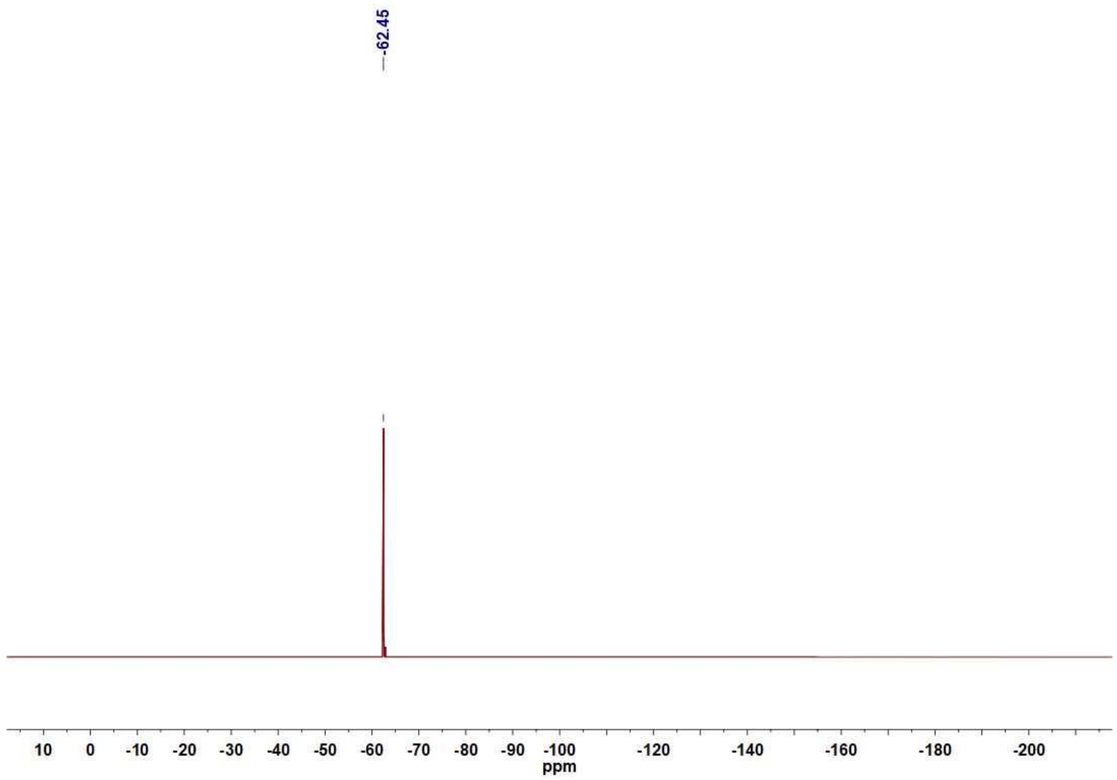


**Supplementary Figure 89. $^{19}$F NMR (471 MHz, $CDCl_3$) spectrum of 7.**

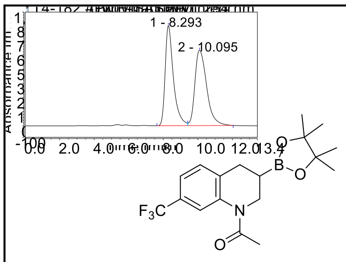


*rac*-**6**

| Integration Results | | | | | | | |
|---|---|---|---|---|---|---|---|
| No. | Peak Name | Retention Time<br>min | Area<br>mAU*min | Height<br>mAU | Relative Area<br>% | Relative Height<br>% | Amount<br>n.a. |
| 1 | | 8.293 | 487.012 | 881.154 | 48.26 | 57.07 | n.a. |
| 2 | | 10.095 | 522.098 | 662.882 | 51.74 | 42.93 | n.a. |
| **Total:** | | | **1009.110** | **1544.036** | **100.00** | **100.00** | |

**Supplementary Figure 90. HPLC spectrum of *rac*-6.**

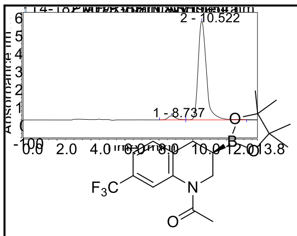


(*R*-**6**) 92% yield, 94% ee

| Integration Results | | | | | | | |
|---|---|---|---|---|---|---|---|
| No. | Peak Name | Retention Time<br>min | Area<br>mAU*min | Height<br>mAU | Relative Area<br>% | Relative Height<br>% | Amount<br>n.a. |
| 1 | | 8.737 | 11.577 | 22.253 | 2.95 | 3.88 | n.a. |
| 2 | | 10.522 | 308.760 | 552.006 | 97.05 | 96.12 | n.a. |
| **Total:** | | | **320.337** | **574.259** | **100.00** | **100.00** | |

**Supplementary Figure 91. HPLC spectrum of *R*-6.**

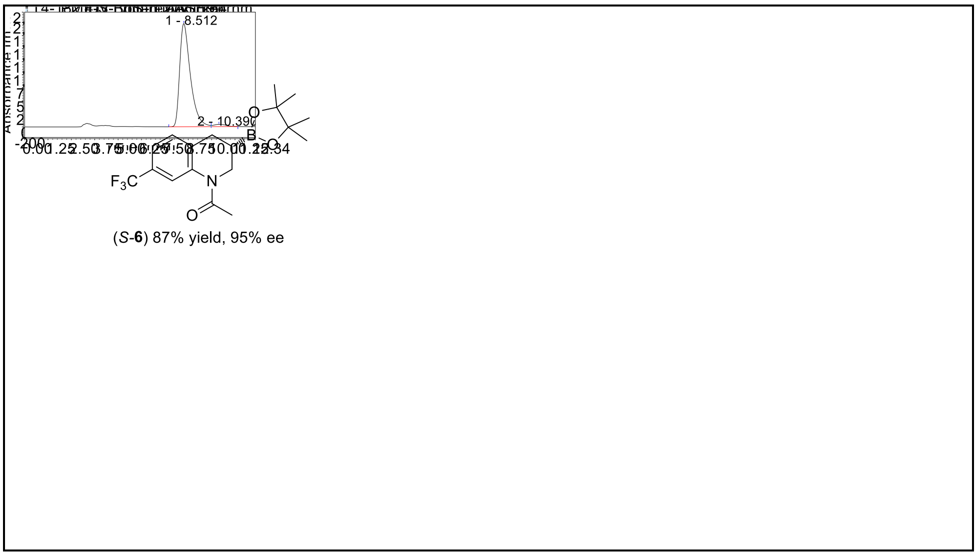


| Integration Results | | | | | | | |
|---|---|---|---|---|---|---|---|
| No. | Peak Name | Retention Time min | Area mAU*min | Height mAU | Relative Area % | Relative Height % | Amount n.a. |
| 1 | | 8.512 | 1310.970 | 1977.178 | 97.58 | 97.87 | n.a. |
| 2 | | 10.390 | 32.450 | 43.087 | 2.42 | 2.13 | n.a. |
| **Total:** | | | **1343.420** | **2020.265** | **100.00** | **100.00** | |

**Supplementary Figure 92. HPLC spectrum of *S*-6.**

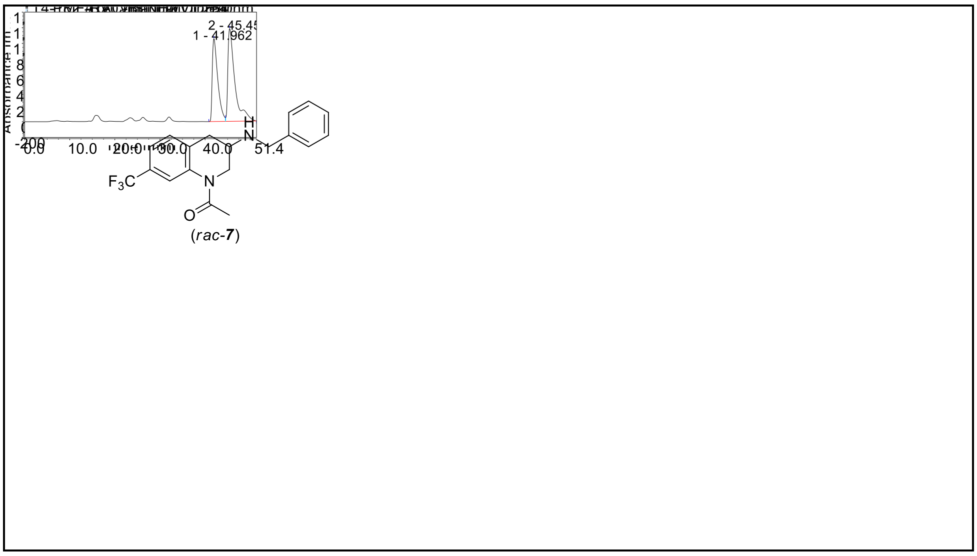


| Integration Results | | | | | | | |
|---|---|---|---|---|---|---|---|
| No. | Peak Name | Retention Time<br>min | Area<br>mAU*min | Height<br>mAU | Relative Area<br>% | Relative Height<br>% | Amount<br>n.a. |
| 1 | | 41.962 | 1508.118 | 1059.190 | 41.47 | 47.18 | n.a. |
| 2 | | 45.453 | 2128.281 | 1186.030 | 58.53 | 52.82 | n.a. |
| **Total:** | | | **3636.399** | **2245.220** | **100.00** | **100.00** | |

**Supplementary Figure 93. HPLC spectrum of *rac*-7.**

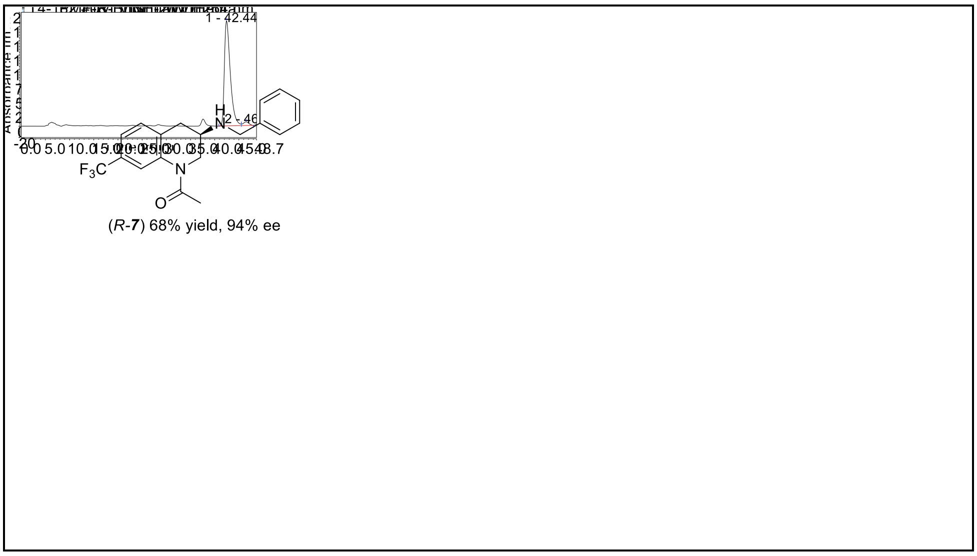


| Integration Results | | | | | | | |
|---|---|---|---|---|---|---|---|
| No. | Peak Name | Retention Time<br>min | Area<br>mAU*min | Height<br>mAU | Relative Area<br>% | Relative Height<br>% | Amount<br>n.a. |
| 1 | | 42.448 | 262.273 | 184.754 | 96.81 | 96.67 | n.a. |
| 2 | | 46.502 | 8.636 | 6.363 | 3.19 | 3.33 | n.a. |
| **Total:** | | | **270.910** | **191.117** | **100.00** | **100.00** | |

**Supplementary Figure 94. HPLC spectrum of *R*-7.**

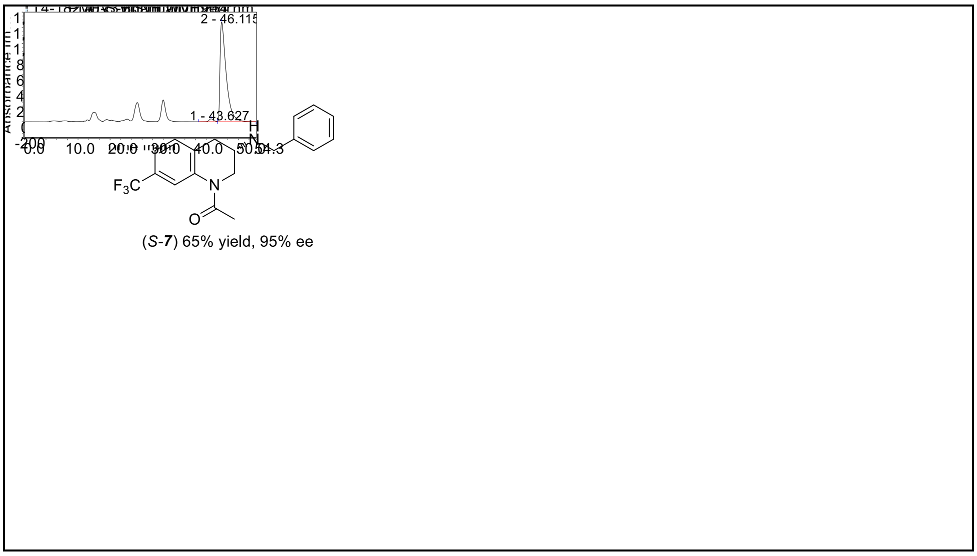


| Integration Results | | | | | | | |
|---|---|---|---|---|---|---|---|
| No. | Peak Name | Retention Time min | Area mAU*min | Height mAU | Relative Area % | Relative Height % | Amount n.a. |
| 1 | | 43.627 | 44.466 | 35.750 | 2.05 | 2.73 | n.a. |
| 2 | | 46.115 | 2127.221 | 1273.781 | 97.95 | 97.27 | n.a. |
| Total: | | | 2171.687 | 1309.531 | 100.00 | 100.00 | |

**Supplementary Figure 95. HPLC spectrum of *S*-7.**

## X-ray crystal structure analysis

### Supplementary Table 2. Crystal data and structure refinement parameters for 1

| | **1** |
|---|---|
| Empirical formula | $C_{58}H_{66}Co_2N_2P_2$ |
| Formula weight | 970.92 |
| Temperature (K) | 172.98(14) |
| Crystal system | triclinic |
| Space group | *P*-1 |
| *a* (Å) | 10.7586(4) |
| *b* (Å) | 16.0325(6) |
| *c* (Å) | 16.1740(6) |
| *α* (°) | 110.623(4) |
| *β* (°) | 101.599(3) |
| *γ* (°) | 98.368(3) |
| Volume (Å$^3$) | 2485.48(17) |
| Z | 2 |
| Density (calculated) (g/cm$^3$) | 1.297 |
| Absorption coefficient (mm$^{-1}$) | 0.613 |
| F(000) | 1024.0 |
| Radiation | Cu*K*α (λ = 1.54184) |
| Crystal color, morphology | orange, block |
| *θ* range (°) | 3.034 to 67.076 |
| Absorption correction | Multi-scan |
| $T_{min}$, $T_{max}$ | 0.632, 1.000 |
| Index ranges | $-12 \leq h \leq 2$, $-19 \leq k \leq 19$, $-19 \leq l \leq 19$ |
| GOF | 1.047 |
| Reflections collected | 23474 |
| Independent reflections | 8746 [$R_{int}$ = 0.0332, $R_{sigma}$ = 0.0386] |
| Final R indexes [I>=2σ (I)] | $R_1$ = 0.0337, $wR_2$ = 0.0840 |
| Final R indexes (all data) | $R_1$ = 0.0515, $wR_2$ = 0.0958 |
| Largest diff. peak / hole (e.Å$^{-3}$) | 0.25/-0.24 |

**Supplementary Table 3. Crystal data and structure refinement parameters for 3h**

| | **3h** |
|---|---|
| Empirical formula | $C_{15}H_{20}BNO_2$ |
| Formula weight | 257.13 |
| Temperature (K) | 173.00(10) |
| Crystal system | trigonal |
| Space group | *R*-3 |
| *a* (Å) | 25.3498(8) |
| *b* (Å) | 25.3498(8) |
| *c* (Å) | 12.7670(4) |
| *α* (°) | 90 |
| *β* (°) | 90 |
| *γ* (°) | 120 |
| Volume (Å$^3$) | 7105.1(5) |
| Z | 18 |
| Density (calculated) (g / cm$^3$) | 1.082 |
| Absorption coefficient (mm$^{-1}$) | 0.553 |
| F(000) | 2484.0 |
| Radiation | Cu*Kα* (*λ* = 1.54184) |
| Crystal color, morphology | colorless, prism |
| *θ* range (°) | 3.478 to 67.068 |
| Absorption correction | Multi-scan |
| $T_{min}$, $T_{max}$ | 0.405, 1.000 |
| Index ranges | -30 ≤ h ≤ 18, -20 ≤ k ≤ 30, -13 ≤ l ≤ 15 |
| GOF | 1.118 |
| Reflections collected | 6203 |
| Independent reflections | 2779 [$R_{int}$ = 0.0315, $R_{sigma}$ = 0.0338] |
| Final *R* indexes [*I*>=2*σ* (*I*)] | $R_1$ = 0.0872, $wR_2$ = 0.2481 |
| Final *R* indexes (all data) | $R_1$ = 0.0998, $wR_2$ = 0.2649 |
| Largest diff. peak / hole (e.Å$^{-3}$) | 0.55/-0.66 |

**Supplementary Table 4. Crystal data and structure refinement parameters for 3u**

| | **3u** |
|---|---|
| Empirical formula | $C_{10}H_{11}N$ |
| Formula weight | 145.20 |
| Temperature (K) | 172.99(10) |
| Crystal system | monoclinic |
| Space group | $P2_1/c$ |
| *a* (Å) | 15.2772(5) |
| *b* (Å) | 5.9053(2) |
| *c* (Å) | 8.7419(2) |
| *α* (°) | 90 |
| *β* (°) | 98.119(3) |
| *γ* (°) | 90 |
| Volume (Å$^3$) | 780.76(4) |
| Z | 4 |
| Density (calculated) (g / cm$^3$) | 1.235 |
| Absorption coefficient (mm$^{-1}$) | 0.553 |
| F(000) | 312.0 |
| Radiation | Cu*Kα* (*λ* = 1.54184) |
| Crystal color, morphology | colorless, block |
| *θ* range (°) | 2.922 to 67.061 |
| Absorption correction | Multi-scan |
| $T_{min}$, $T_{max}$ | 0.304, 1.000 |
| Index ranges | -18 ≤ h ≤ 18, -6 ≤ k ≤ 6, -9 ≤ l ≤ 10 |
| GOF | 1.116 |
| Reflections collected | 4084 |
| Independent reflections | 1384 [$R_{int}$ = 0.0315, $R_{sigma}$ = 0.0338] |
| Final *R* indexes [$I$>=2$\sigma$ ($I$)] | $R_1$ = 0.0579, $wR_2$ = 0.1622 |
| Final *R* indexes (all data) | $R_1$ = 0.0631, $wR_2$ = 0.1674 |
| Largest diff. peak / hole (e.Å$^{-3}$) | 0.55/-0.50 |

**Supplementary Table 5. Crystal data and structure refinement parameters for 3ag**

| | **3ag** |
|---|---|
| Empirical formula | $C_{13}H_{11}N$ |
| Formula weight | 181.23 |
| Temperature (K) | 172.99(10) |
| Crystal system | monoclinic |
| Space group | $P2_1$ |
| $a$ (Å) | 8.7969(5) |
| $b$ (Å) | 5.9315(3) |
| $c$ (Å) | 9.3645(5) |
| $\alpha$ (°) | 90 |
| $\beta$ (°) | 99.195(5) |
| $\gamma$ (°) | 90 |
| Volume (Å$^3$) | 482.35(5) |
| Z | 2 |
| Density (calculated) (g / cm$^3$) | 1.248 |
| Absorption coefficient (mm$^{-1}$) | 0.559 |
| F(000) | 192.0 |
| Radiation | Cu$K\alpha$ ($\lambda$ = 1.54184) |
| Crystal color, morphology | colorless, block |
| $\theta$ range (°) | 4.784 to 67.014 |
| Absorption correction | Multi-scan |
| $T_{min}$, $T_{max}$ | 0.808, 1.000 |
| Index ranges | $-10 \leq h \leq 6$, $-7 \leq k \leq 6$, $-10 \leq l \leq 11$ |
| GOF | 1.093 |
| Reflections collected | 2623 |
| Independent reflections | 1442 [$R_{int}$ = 0.0315, $R_{sigma}$ = 0.0338] |
| Final $R$ indexes [$I$>=2$\sigma$ ($I$)] | $R_1$ = 0.0604, $wR_2$ = 0.1570 |
| Final $R$ indexes (all data) | $R_1$ = 0.0691, $wR_2$ = 0.1730 |
| Largest diff. peak / hole (e.Å$^{-3}$) | 0.24/-0.25 |

**Supplementary Table 6. Crystal data and structure refinement parameters for 4u**

| | **4u** |
|---|---|
| Empirical formula | $C_{10}H_{13}N$ |
| Formula weight | 147.21 |
| Temperature (K) | 172.98(10) |
| Crystal system | monoclinic |
| Space group | $P2_1/c$ |
| *a* (Å) | 15.2608(6) |
| *b* (Å) | 6.1671(3) |
| *c* (Å) | 8.6714(4) |
| *α* (°) | 90 |
| *β* (°) | 96.856(4) |
| *γ* (°) | 90 |
| Volume (Å$^3$) | 810.27(6) |
| Z | 4 |
| Density (calculated) (g / cm$^3$) | 1.207 |
| Absorption coefficient (mm$^{-1}$) | 0.533 |
| F(000) | 320.0 |
| Radiation | Cu*Kα* (*λ* = 1.54184) |
| Crystal color, morphology | colorless, block |
| *θ* range (°) | 2.916 to 67.065 |
| Absorption correction | Multi-scan |
| $T_{min}$, $T_{max}$ | 0.595, 1.000 |
| Index ranges | -18 ≤ h ≤ 12, -6 ≤ k ≤ 7, -10 ≤ l ≤ 10 |
| GOF | 1.104 |
| Reflections collected | 3898 |
| Independent reflections | 1425 [$R_{int}$ = 0.0445, $R_{sigma}$ = 0.0382] |
| Final *R* indexes [$I$>=2$\sigma$ ($I$)] | $R_1$ = 0.0729, $wR_2$ = 0.2081 |
| Final *R* indexes (all data) | $R_1$ = 0.0824, $wR_2$ = 0.2203 |
| Largest diff. peak / hole (e.Å$^{-3}$) | 0.40/-0.49 |

**Supplementary Table 7. Crystal data and structure refinement parameters for *R*-6**

| | ***R*-6** |
|---|---|
| Empirical formula | $C_{18}H_{23}BF_3NO_3$ |
| Formula weight | 369.18 |
| Temperature (K) | 172.98(10) |
| Crystal system | orthorhombic |
| Space group | $P2_12_12_1$ |
| $a$ (Å) | 10.6508(7) |
| $b$ (Å) | 12.4481(6) |
| $c$ (Å) | 27.8553(13) |
| $\alpha$ (°) | 90 |
| $\beta$ (°) | 90 |
| $\gamma$ (°) | 90 |
| Volume ($Å^3$) | 3693.1(3) |
| Z | 8 |
| Density (calculated) (g / $cm^3$) | 1.328 |
| Absorption coefficient ($mm^{-1}$) | 0.920 |
| F(000) | 1552.0 |
| Radiation | Cu$K\alpha$ ($\lambda$ = 1.54184) |
| Crystal color, morphology | colorless, needle |
| $\theta$ range (°) | 3.173 to 67.063 |
| Absorption correction | Multi-scan |
| $T_{min}$, $T_{max}$ | 0.595, 1.000 |
| Index ranges | $-12 \leq h \leq 12$, $-14 \leq k \leq 14$, $-33 \leq l \leq 15$ |
| GOF | 1.043 |
| Reflections collected | 11568 |
| Independent reflections | 6027 [$R_{int}$ = 0.0771, $R_{sigma}$ = 0.0946] |
| Final $R$ indexes [$I$>=2$\sigma$ ($I$)] | $R_1$ = 0.0837, $wR_2$ = 0.2261 |
| Final $R$ indexes (all data) | $R_1$ = 0.1086, $wR_2$ = 0.2568 |
| Largest diff. peak / hole (e.$Å^{-3}$) | 0.58/-0.31 |

**Supplementary Table 8. Crystal data and structure refinement parameters for *S*-6**

| | ***S*-6** |
|---|---|
| Empirical formula | $C_{18}H_{23}BF_3NO_3$ |
| Formula weight | 369.18 |
| Temperature (K) | 172.98(10) |
| Crystal system | orthorhombic |
| Space group | $P2_12_12_1$ |
| $a$ (Å) | 10.6542(3) |
| $b$ (Å) | 12.4646(3) |
| $c$ (Å) | 27.8600(7) |
| $\alpha$ (°) | 90 |
| $\beta$ (°) | 90 |
| $\gamma$ (°) | 90 |
| Volume (Å$^3$) | 3699.82(17) |
| Z | 8 |
| Density (calculated) (g / cm$^3$) | 1.326 |
| Absorption coefficient (mm$^{-1}$) | 0.920 |
| F(000) | 1552.0 |
| Radiation | Cu$K\alpha$ ($\lambda$ = 1.54184) |
| Crystal color, morphology | colorless, needle |
| $\theta$ range (°) | 3.173 to 67.064 |
| Absorption correction | Multi-scan |
| $T_{min}$, $T_{max}$ | 0.595, 1.000 |
| Index ranges | $-9 \leq h \leq 12$, $-8 \leq k \leq 14$, $-33 \leq l \leq 32$ |
| GOF | 1.073 |
| Reflections collected | 11665 |
| Independent reflections | 6147 [$R_{int}$ = 0.0805, $R_{sigma}$ = 0.0875] |
| Final $R$ indexes [$I$>=2$\sigma$ ($I$)] | $R_1$ = 0.0951, $wR_2$ = 0.2576 |
| Final $R$ indexes (all data) | $R_1$ = 0.1106, $wR_2$ = 0.2870 |
| Largest diff. peak / hole (e.Å$^{-3}$) | 0.78/-0.32 |

## Computational details

All calculations presented here in were performed with the Gaussian16 program package[11]. The B3LYP-D3 functional (with Grimme's empirical D3 dispersion correction)[12,13] was used for geometry optimizations with the standard 6-31G(d,p) basis set (SDD[14] pseudupotential for Co). On the basis of the optimized geometries, harmonic vibrational frequency calculations were carried out to verify the nature of all stationary points being local minima or transition state and also to obtain the thermochemical corrections for the Gibbs free energies at 298.15 K. The final and solvation energies in tetrahydrofuran (ε= 7.4257) were calculated using the SMD[15] solvation model with the B3LYP*-D3[16] functional (15% Hartree-Fock exchange including D3 dispersion correction from B3LYP) employing a larger basis set, which is 6-311+G(2df,2pd) for all elements expect Co. It has been revealed that the use of 15% HF exchange gave better relative energies of different spin states for first-row transition metal complexes[17-19]. A concentration correction of 1.9 kcal/mol at room temperature [derived from the free energy change of 1 mol of an ideal gas from 1 atm (24.5 L/mol) to 1M (1 mol/L) at 298.15 K] was added for all species. The final energy reported is B3LYP-D3* free energy including solvation, Gibbs free energy, and D3 dispersion corrections.

Kinetic isotope effects (KIEs) were calculated using conventional transition state theory.

$$k = \frac{k_B T}{h} \exp(-\Delta G^{\neq} / RT) \qquad (1)$$

In equation (1), k, $k_B$, T, h, and R are rate constant, Boltzmann's constant, temperature, Planck's constant, and gas constant, respectively. $\Delta G^{\neq}$ is the total Gibbs energy barrier. Then, the KIEs for $D_3N$ ‑$BH_3$, $H_3N$ ‑$BD_3$, and $D_3N$ ‑$BD_3$ can be expressed as the ration of $\frac{k(H_3NBH_3)}{k(D_3NBH_3)}$, $\frac{k(H_3NBH_3)}{k(H_3NBD_3)}$, and $\frac{k(H_3NBH_3)}{k(D_3NBD_3)}$, respectively.

$$KIE(D_3NBH_3) = \frac{k(H_3NBH_3)}{k(D_3NBH_3)} = \exp((\Delta G^{\neq}_{D_3NBH_3} - \Delta G^{\neq}_{H_3NBH_3})/RT) \qquad (2)$$

$$KIE(H_3NBD_3) = \frac{k(H_3NBH_3)}{k(H_3NBD_3)} = \exp((\Delta G^{\neq}_{H_3NBD_3} - \Delta G^{\neq}_{H_3NBH_3})/RT) \qquad (3)$$

$$KIE(D_3NBD_3) = \frac{k(H_3NBH_3)}{k(D_3NBD_3)} = \exp((\Delta G^{\neq}_{D_3NBD_3} - \Delta G^{\neq}_{H_3NBH_3})/RT) \qquad (4)$$

It should be pointed out that tunneling and re-crossing effects were not taken into account since it is beyond the scope of the present study.

## Computational results

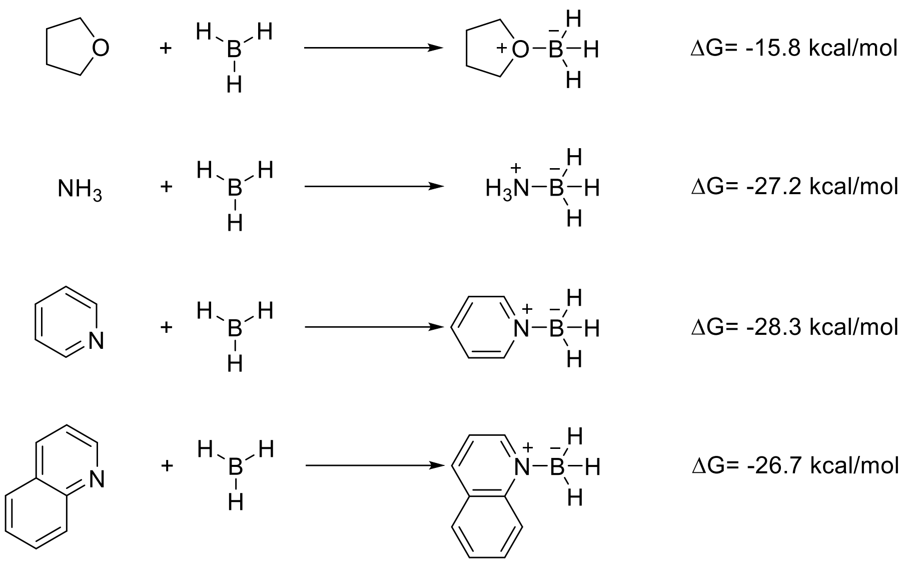


**Supplementary Figure 96. Calculated binding Gibbs free energies of $BH_3$ with different species.**

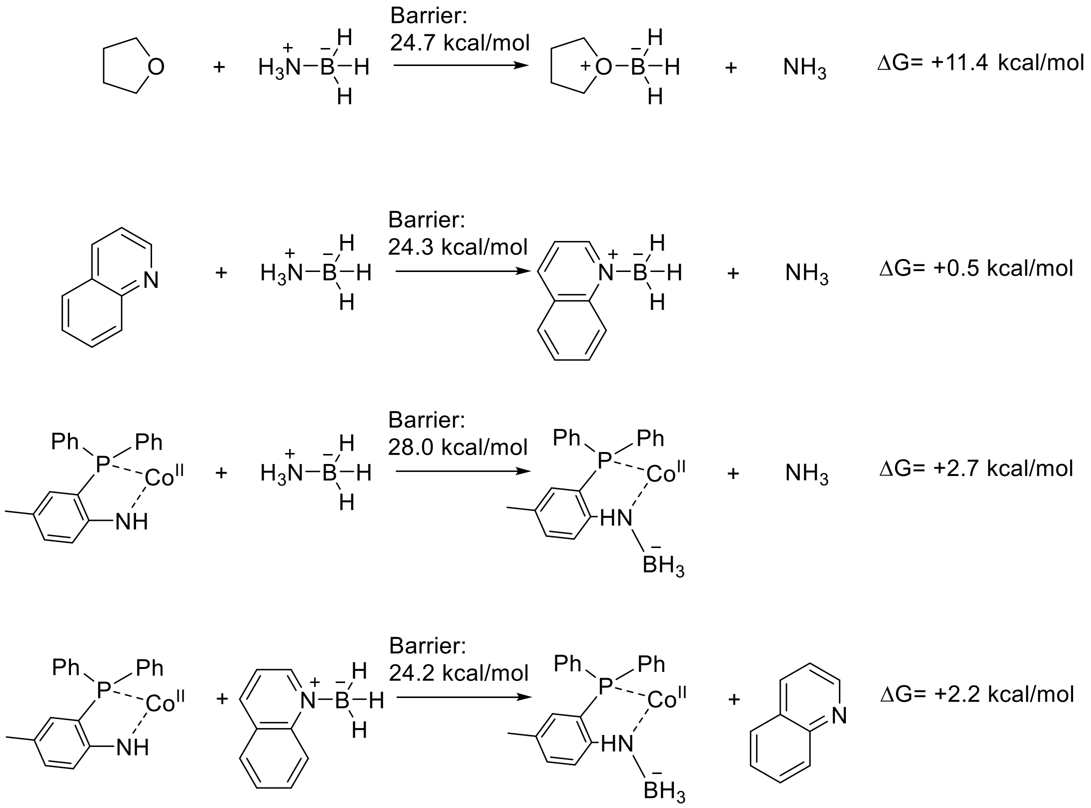


**Supplementary Figure 97. Barriers and reaction energies for the $BH_3$ transfer reactions.**

Five different modes of activation of $H_3N{\cdot}BH_3$ (Supplementary Fig. 98) have been considered. **Mode I** involves transfer hydrogenation from $H_3N{\cdot}BH_3$ to the catalyst (amido-cobalt), which was found to be the most favorable mode. Four different pathways (A, B, C, and D, Supplementary Figures 99-101) have been located on the basis of this mode. **Mode II** and **III** (pathways E, F, and G, Supplementary Figures 102-105) involve activation of $BH_3$ by the catalyst. **Mode IV** (pathway H, Supplementary Figures 106 and 107) involves the activation of a B-H bond by the catalyst, while **Mode V** (pathway I, Supplementary Fig. 108) involves activation of $NH_3$ by Co and activation of $BH_3$ by the amido moiety.

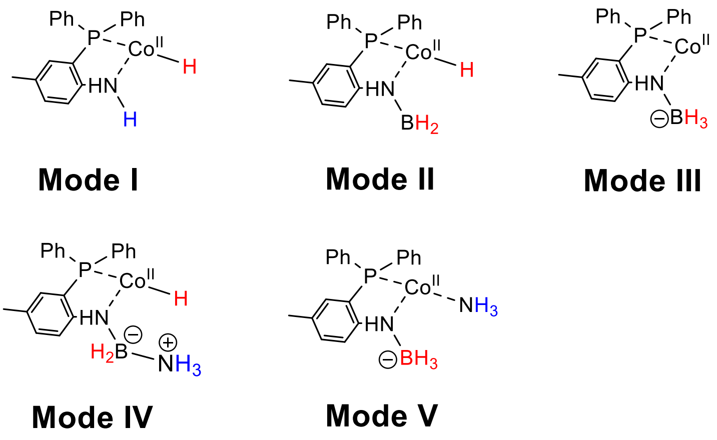


**Supplementary Figure 98. Five possible modes of activation of $H_3N{\cdot}BH_3$ by the cobalt catalyst.**

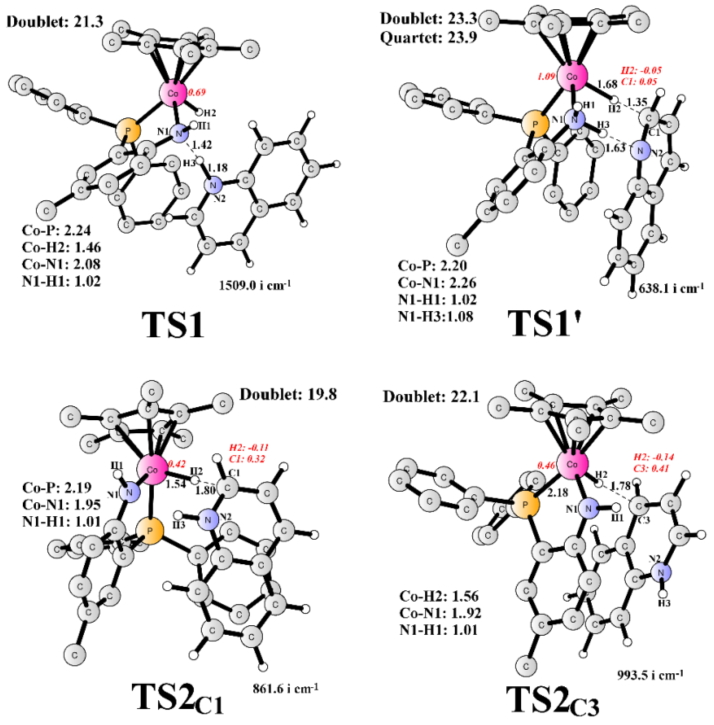


**Supplementary Figure 99. Structures of transition states for the cobalt-catalyzed semi-hydrogenation of quinoline.** For clarity, unimportant hydrogen atoms are not shown. Relative energies are given in kcal/mol relative to **Int1** plus quinoline. Distances are given in Ångstrom. Spin densities on selected atoms are shown in red italics for the spin state with the lowest energy. The imaginary frequencies are also indicated.

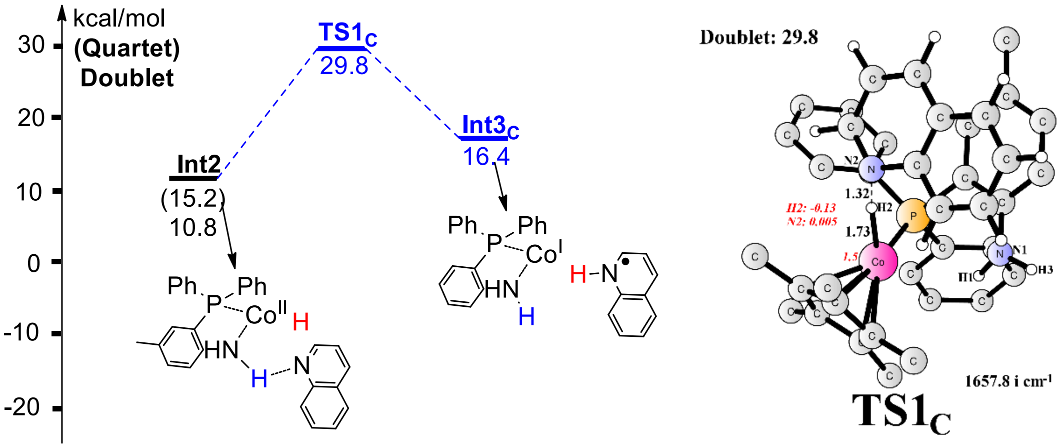


**Supplementary Figure 100. Gibbs energy diagram and structure of TS1C for pathway C.** The hydrogen atom is transferred from $Co^{II}$-H to the quinoline nitrogen atom. Structures of transition states for pathway D.

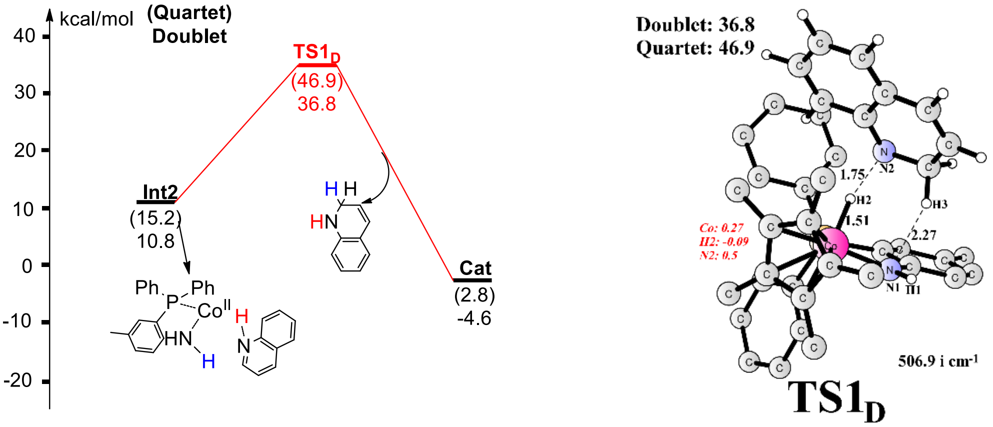


**Supplementary Figure 101. Gibbs energy diagram and structure of TS1D for pathway D.** The hydride is transferred to the quinoline nitrogen. Structures of transition states for pathway D.

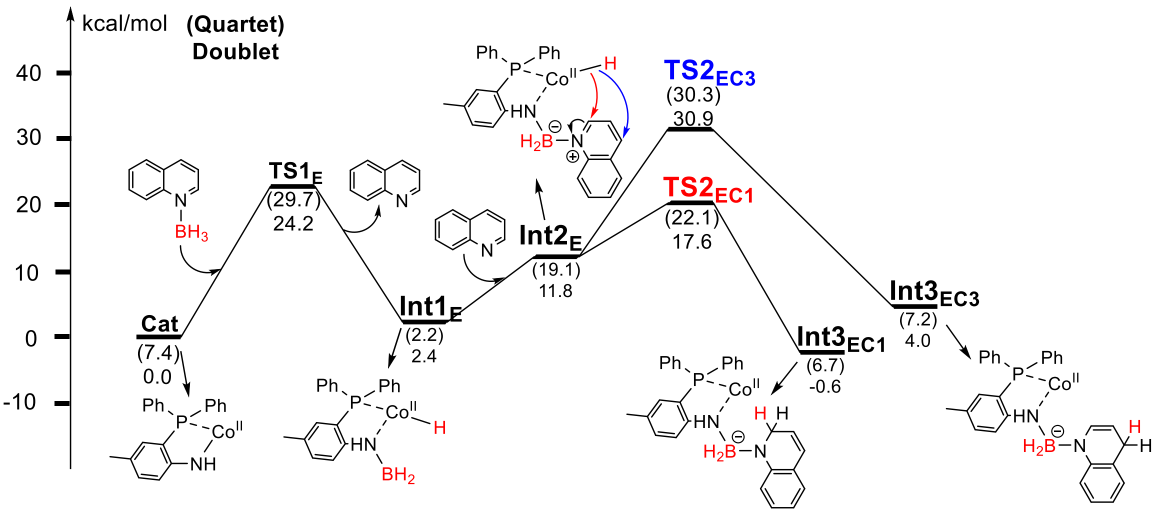


**Supplementary Figure 102. Gibbs energy diagram for pathway E.**

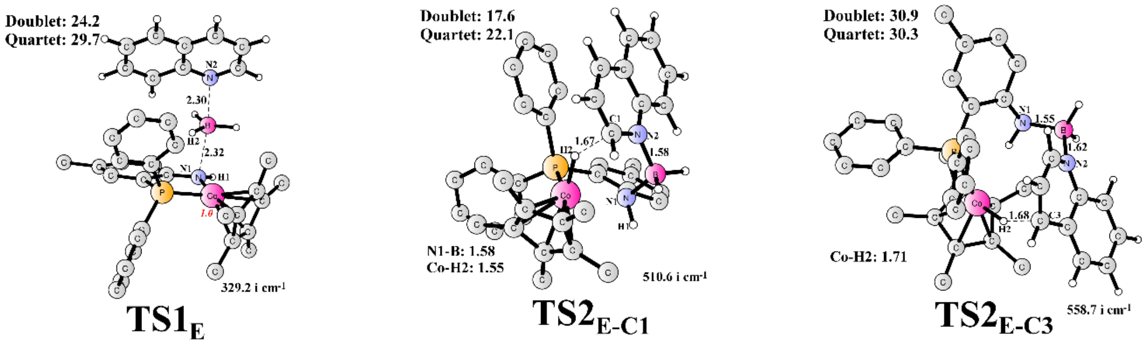


**Supplementary Figure 103. Structures of transition states for pathway E.**

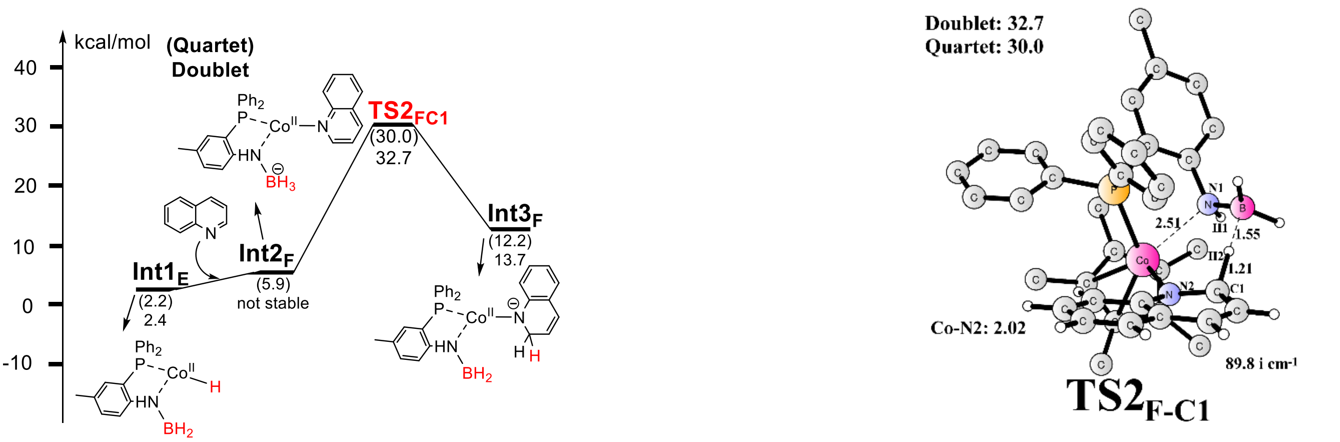


**Supplementary Figure 104. Gibbs energy diagram and structure of TS2FC1 for pathway F.** The hydride is transferred from $BH_3$ to the quinoline C1.

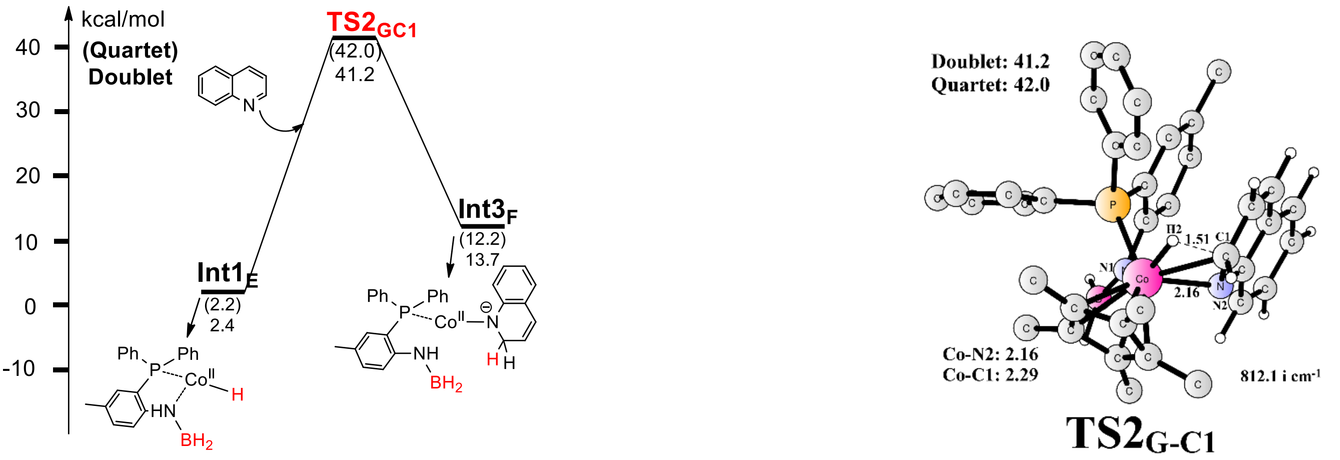


**Supplementary Figure 105. Gibbs energy diagram and structure of TS2GC1 for pathway G.** The hydride is transferred from Co-H to the quinoline C1.

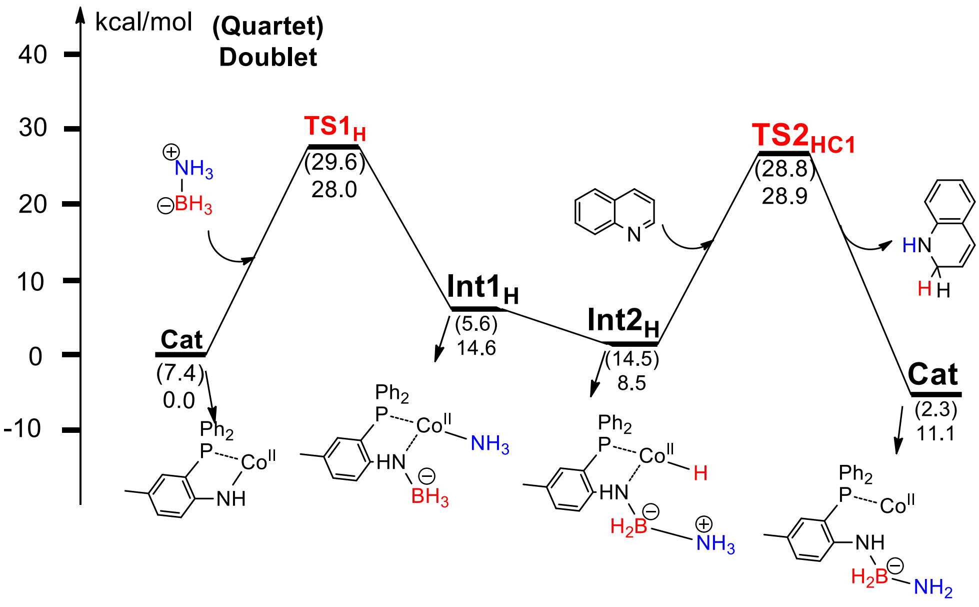


**Supplementary Figure 106. Gibbs energy diagram for pathway H.**

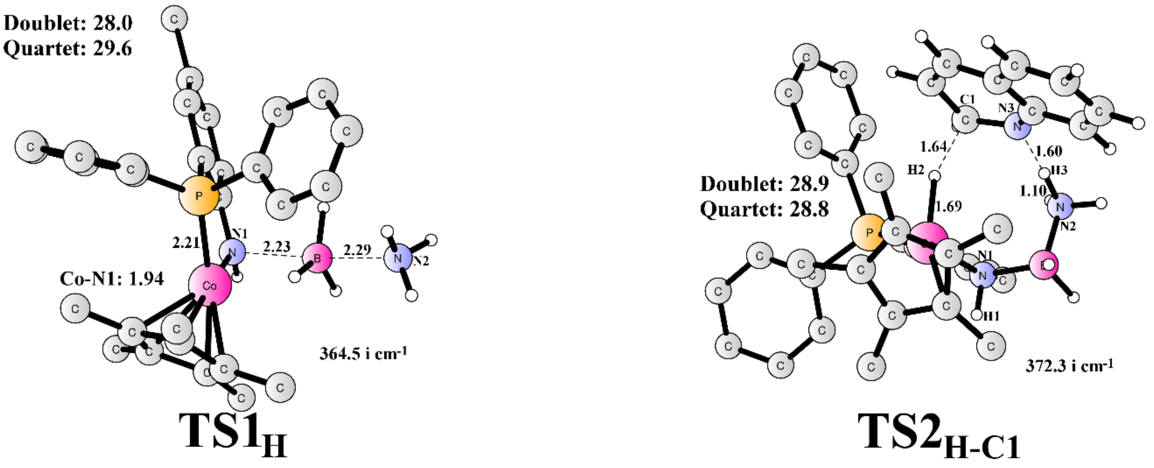


**Supplementary Figure 107. Structures of transition states for pathway H.**

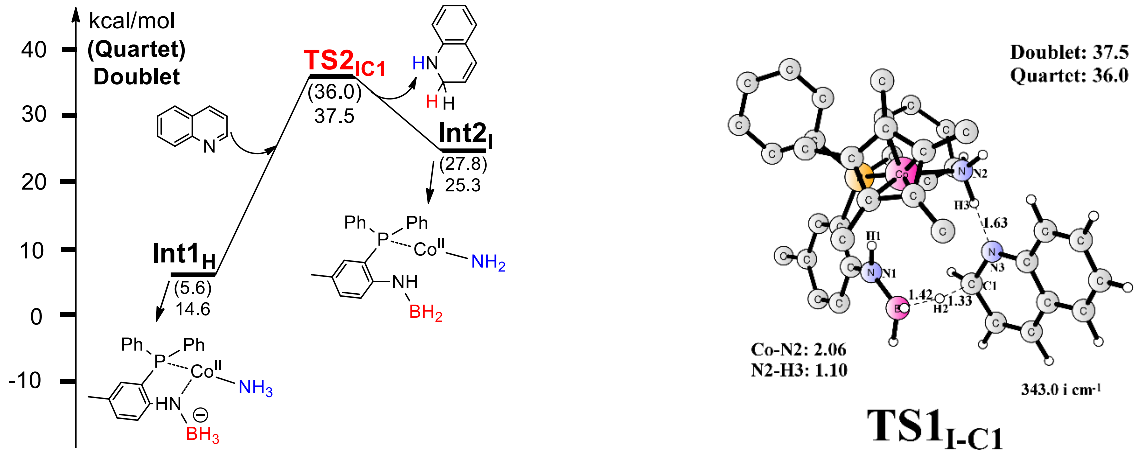


**Supplementary Figure 108. Gibbs energy diagram and structure of $TS2_{IC1}$ for pathway I.** The hydride is transferred from $BH_3$ to the quinoline C1, coupled with a proton transfer from $NH_3$ to quinoline nitrogen.

## Supplementary References


(1) Gelman, D., Jiang, L. & Buchwald, S. L. Copper-Catalyzed C−P Bond Construction *via* Direct Coupling of Secondary Phosphines and Phosphites with Aryl and Vinyl Halides. *Org Lett*. **5**, 2315−2318 (2003).

(2) Zimmermann, C., Anson, C. E. & Dehnen, S. Chalcogenido-Bridged Clusters by Reactions of Chalcogenostannate Salts. *J. Cluster Sci.* **18**, 618−629 (2007).

(3) Pearson, W. H. & Fang, W. K. Reactions of Azides with Electrophiles: New Methods for the Generation of Cationic 2-Azabutadienes. Synthesis of 1, 2, 3, 4-Tetrahydroquinolines and 1, 2-Dihydroquinolines *via* A Hetero Diels–Alder Reaction. *Isr. J. Chem*. **37**, 39−46 (1997).

(4) Idahosa, K. C., Davies-Coleman, M. T. & Kaye, P. T. Exploratory Applications of 2-Nitrobenzaldehyde-Derived Morita–Baylis–Hillman Adducts as Synthons in the Construction of Drug-Like Scaffolds. *Synth. Commun.* **49**, 417−430 (2019).

(5) Batsyts, S., Hübner, E. G., Namyslo, J. C., Gjikaj, M. & Schmidt, A. Synthesis and Characterization of Propeller-Shaped Mono-to Hexacationic Quinolinium-Substituted Benzenes. *Org. Biomol. Chem.* **17**, 4102−4114 (2019).

(6) Forrest, T. P., Dauphinee, G. A. & Deraniyagala, S. A. On the Mechanism of Disproportionation Reactions of 1, 2-Dihydroquinolines. *Can. J. Chem.* **63**, 412−417 (1985).

(7) Fu, Y., Li, G. Y., Ye, F., Zhang, S. S. & Gao, S. Synthesis and Biological Activity of Some Novel *N*-Dichloroacetyl-2, 3-Dihydrobenzoxazole Derivatives. *Heterocycl. Commun.* **17**, 57−60 (2011).

(8) Ding, F. *et al.* $B(C_6F_5)_3$-Promoted Hydrogenations of *N*-Heterocycles with Ammonia Borane. *Chem. Commun.* **53**, 9262−9264 (2017).

(9) Lu, L. Q., Li, Y., Junge, K. & Beller, M. Relay Iron/Chiral Brønsted Acid Catalysis: Enantioselective Hydrogenation of Benzoxazinones. *J. Am. Chem. Soc.* **137**, 2763−2768 (2015).

(10) Kubota, K., Watanabe, Y. & Ito, H. Synthesis of Enantiomerically Enriched Chiral Tetrahydroquinolines via Sequential Dearomatization/Enantioselective Borylation Reactions. *Adv. Synth. Catal.* **358**, 2379−2384 (2016).

(11) Frisch, M. J. *et al.* Gaussian 16, Revision B.01, Gaussian, Inc., Wallingford CT, 2016.

(12) Becke, A. D. Density-Functional Thermochemistry. III. The Role of Exact Exchange. *J. Chem. Phys*. **98**, 5648−5652 (1993).

(13) Grimme, S. Density Functional Theory with London Dispersion Corrections. *Wiley Interdiscip. Rev.: Comput. Mol. Sci.* **1**, 211−228 (2011).

(14) Andrae, D., HaiuBermann, U., Dolg, M., Stoll, H. & PreuB, H. Energy-Adjusted ab Initio Pseudopotentials for the Second and Third Row Transition Elements. *Theor. Chim. Acta.* **77**, 123−144 (1990).

(15) Marenich, A. V., Cramer, C. J. & Truhlar, D. G. Universal Solvation Model Based on Solute Electron Density and on A Continuum Model of the Solvent Defined by the Bulk Dielectric Constant and Atomic Surface Tensions. *J. Phys. Chem. B.* **113**, 6378–6396 (2009).

(16) Reiher, M., Salomon, O. & Hess, B. A. Reparameterization of Hybrid Functionals Based on Energy Differences of States of Different Multiplicity. *Theor. Chem. Acc.* **107**, 48–55 (2001).
(17) Salomon, O., Reiher, M. & Hess, B. A. Assertion and Validation of the Performance of the B3LYP* Functional for the First Transition Metal Row and the G2 Test Set. *J. Chem. Phys.* **117**, 4729–4737 (2002).
(18) Neese, F. Prediction of Molecular Properties and Molecular Spectroscopy with Density Functional Theory: from Fundamental Theory to Exchange-Coupling. *Coord. Chem. Rev.* **253**, 526–563 (2009).
(19) Blomberg, M. R. A., Borowski, T., Himo, F., Liao, R.-Z. & Siegbahn, P. E. M. Quantum Chemical Studies of Mechanisms for Metalloenzymes. *Chem. Rev.* **114**, 3601–3658 (2014).
