# Supplementary material for: Controlled partial transfer hydrogenation of quinolines by cobalt-amido cooperative catalysis
Source: Nat Commun. 2020 Mar 6;11:1249. doi: 10.1038/s41467-020-15118-x (PMC7060234; doi:10.1038/s41467-020-15118-x)
Supplement: Supplementary file 2 — Description of Additional Supplementary Files [file 41467_2020_15118_MOESM2_ESM.pdf]

Description of Additional Supplementary Files

File Name: Supplementary Data 1

Description: Cartesian coordinates for all computed structures.
