# Supplementary material for: Controlled partial transfer hydrogenation of quinolines by cobalt-amido cooperative catalysis
Source: Nat Commun. 2020 Mar 6;11:1249. doi: 10.1038/s41467-020-15118-x (PMC7060234; doi:10.1038/s41467-020-15118-x)
Supplement: Supplementary file 3 — Supplementary Data 1 [file 41467_2020_15118_MOESM3_ESM.pdf]

Cartesian coordinates for all computed structures

Quinoline

C	-2.415608000	-0.964661000	-0.013640000
C	-1.038771000	-0.940771000	-0.013830000
C	-0.340651000	0.296141000	-0.013739000
C	-1.091026000	1.516193000	-0.013435000
C	-2.509710000	1.458361000	-0.013239000
C	-3.157898000	0.243680000	-0.013343000
H	-2.942483000	-1.914496000	-0.013719000
H	-0.449027000	-1.851634000	-0.014058000
C	-0.361937000	2.733204000	-0.013338000
H	-3.073128000	2.388191000	-0.013005000
H	-4.243275000	0.205585000	-0.013195000
C	1.012235000	2.693674000	-0.013536000
C	1.657189000	1.429524000	-0.013838000
H	-0.899337000	3.678377000	-0.013110000
H	1.605657000	3.602431000	-0.013466000
H	2.746216000	1.385272000	-0.013984000
N	1.025904000	0.272681000	-0.013926000

Quinoline·BH3

C	2.419572000	-0.693977000	-0.000004000
C	1.240582000	-1.408361000	-0.000008000
C	0.002170000	-0.721810000	-0.000007000
C	-0.007580000	0.707933000	-0.000001000
C	1.227531000	1.408197000	0.000003000
C	2.419314000	0.721303000	0.000002000
H	3.365204000	-1.227589000	-0.000005000
H	1.236887000	-2.489396000	-0.000012000
C	-1.254822000	1.379314000	-0.000001000
H	1.209907000	2.494457000	0.000007000
H	3.361399000	1.260792000	0.000005000
C	-2.416020000	0.644999000	-0.000005000
C	-2.336152000	-0.760439000	-0.000010000
H	-1.275699000	2.465589000	0.000004000
H	-3.391923000	1.116940000	-0.000004000
H	-3.226211000	-1.377615000	-0.000014000
N	-1.187818000	-1.429441000	-0.000011000
B	-1.212736000	-3.050178000	-0.000017000
H	-0.630814000	-3.401358000	-1.005546000
H	-0.630817000	-3.401366000	1.005511000
H	-2.381256000	-3.362527000	-0.000021000

$H_3N{\cdot}BH_3$

N	0.000000000	0.000000000	0.732404000
H	-0.558890000	0.769570000	1.096798000
H	0.945912000	0.099228000	1.096798000
H	-0.387022000	-0.868798000	1.096798000
B	0.000000000	0.000000000	-0.938223000
H	0.476463000	1.070595000	-1.242035000
H	0.688931000	-0.947927000	-1.242035000
H	-1.165394000	-0.122668000	-1.242035000

Catalyst1

Co	-1.378975000	0.603532000	-0.163274000
P	0.651070000	-0.230784000	-0.032970000
N	-0.541899000	2.253074000	-0.425400000
C	-3.410804000	1.046635000	-0.435006000
C	-3.188869000	0.851065000	0.981041000
C	0.814910000	2.428378000	-0.334969000
C	-3.148750000	-0.188425000	-1.110116000
C	3.025818000	1.393557000	-0.013136000
C	1.636163000	1.287510000	-0.115499000
C	-2.657717000	-1.102278000	-0.134147000
C	1.229772000	-1.314765000	-1.398199000
C	1.130587000	-1.107033000	1.505135000
C	1.477088000	3.676802000	-0.440456000
H	0.889200000	4.576430000	-0.609151000
C	-2.719185000	-0.467065000	1.171867000
C	3.669353000	2.627914000	-0.112596000
C	-3.924970000	2.305360000	-1.069641000
H	-3.589045000	3.192005000	-0.522820000
H	-5.022136000	2.331118000	-1.084857000
H	-3.577476000	2.399601000	-2.102593000
C	-2.277090000	-2.535818000	-0.374963000
H	-1.803963000	-2.672386000	-1.350975000
H	-3.157550000	-3.189841000	-0.334706000
H	-1.573545000	-2.890083000	0.382572000
C	0.454207000	-1.362035000	-2.565867000
H	-0.473161000	-0.797971000	-2.602673000
C	-3.346264000	-0.452804000	-2.574903000
H	-3.034383000	0.401387000	-3.184667000
H	-4.400761000	-0.652074000	-2.807752000
H	-2.773134000	-1.323404000	-2.905687000
C	-3.376170000	1.906761000	2.031053000
H	-2.872244000	1.637046000	2.962392000
H	-4.438338000	2.062963000	2.258684000
H	-2.965014000	2.866451000	1.702245000
C	2.857843000	3.759162000	-0.329520000

H	3.331683000	4.735731000	-0.413280000
C	1.436900000	-0.354466000	2.649793000
H	1.506138000	0.726719000	2.575158000
C	0.871495000	-2.118397000	-3.662076000
H	0.262608000	-2.147996000	-4.561270000
C	2.432497000	-2.036088000	-1.345228000
H	3.038915000	-2.014760000	-0.444679000
C	-2.368307000	-1.123689000	2.475535000
H	-1.520833000	-1.805047000	2.369108000
H	-3.212064000	-1.704748000	2.870878000
H	-2.090293000	-0.385956000	3.232813000
C	1.052423000	-2.504897000	1.609533000
H	0.824338000	-3.102021000	0.732103000
C	1.659100000	-0.988244000	3.871959000
H	1.898375000	-0.394432000	4.749589000
C	2.066356000	-2.837893000	-3.599446000
H	2.390351000	-3.431033000	-4.449885000
C	2.845796000	-2.794994000	-2.440115000
H	3.776782000	-3.352583000	-2.389225000
C	1.277730000	-3.136140000	2.834194000
H	1.218190000	-4.218861000	2.900097000
C	1.578247000	-2.379786000	3.968572000
H	1.751798000	-2.871514000	4.921357000
H	3.617202000	0.495203000	0.153833000
H	-1.059047000	3.115788000	-0.556315000
C	5.169533000	2.758967000	0.004571000
H	5.611894000	3.186443000	-0.903970000
H	5.457302000	3.412945000	0.837360000
H	5.641638000	1.785989000	0.172859000

Int1

Co	-1.356607000	-0.094820000	-0.374336000
P	0.729902000	0.299991000	0.205744000
N	-0.652407000	-1.334786000	-1.605328000
C	-2.948640000	0.117239000	1.091300000
C	-3.429099000	-0.522561000	-0.078953000
C	0.709525000	-1.537957000	-1.763890000
C	-2.428891000	1.401563000	0.691113000
C	2.974198000	-1.101638000	-0.928260000
C	1.598012000	-0.872640000	-0.881736000
C	-2.657292000	1.586903000	-0.711711000
C	1.347713000	0.039266000	1.904954000
C	1.318774000	1.965294000	-0.305740000
C	1.279365000	-2.406395000	-2.720334000
H	0.632095000	-2.917866000	-3.430222000
C	-3.237188000	0.377426000	-1.197377000

C	3.533440000	-1.978710000	-1.861871000
C	-3.034030000	-0.393381000	2.500231000
H	-2.924977000	-1.479825000	2.543932000
H	-3.998522000	-0.131479000	2.955824000
H	-2.250887000	0.039227000	3.128433000
C	-2.297386000	2.807080000	-1.510304000
H	-1.354642000	3.243178000	-1.167003000
H	-3.070268000	3.583868000	-1.440121000
H	-2.172120000	2.565030000	-2.569499000
C	0.588339000	-0.794965000	2.738321000
H	-0.332849000	-1.231098000	2.368610000
C	-1.886541000	2.433782000	1.636948000
H	-1.247289000	1.981584000	2.401269000
H	-2.705470000	2.947970000	2.155579000
H	-1.299833000	3.189372000	1.111538000
C	-4.033681000	-1.895422000	-0.147260000
H	-3.825824000	-2.374286000	-1.110325000
H	-5.124880000	-1.861022000	-0.036244000
H	-3.636574000	-2.537009000	0.644361000
C	2.655034000	-2.611226000	-2.758985000
H	3.063656000	-3.287985000	-3.506874000
C	1.591382000	2.198960000	-1.663231000
H	1.566239000	1.370598000	-2.364871000
C	1.036306000	-1.095401000	4.025166000
H	0.447213000	-1.755308000	4.654502000
C	2.562774000	0.566193000	2.372642000
H	3.157737000	1.213818000	1.735769000
C	-3.616941000	0.087811000	-2.620151000
H	-2.969236000	0.620584000	-3.322024000
H	-4.651613000	0.387479000	-2.828367000
H	-3.541901000	-0.980950000	-2.845168000
C	1.361240000	3.043463000	0.591562000
H	1.157245000	2.880783000	1.644557000
C	1.896756000	3.483442000	-2.111636000
H	2.107390000	3.649158000	-3.164345000
C	2.238209000	-0.559303000	4.490303000
H	2.584618000	-0.789890000	5.493806000
C	3.001426000	0.269876000	3.662906000
H	3.939915000	0.683225000	4.021516000
C	1.670396000	4.327936000	0.139756000
H	1.704166000	5.151999000	0.846779000
C	1.935309000	4.552204000	-1.212374000
H	2.174133000	5.552099000	-1.562842000
H	3.623002000	-0.583008000	-0.225534000
H	-1.195634000	-1.651111000	-2.403724000
B	-0.959004000	-3.987275000	1.548203000

H	0.144949000	-3.583060000	1.841478000
H	-1.120443000	-5.162150000	1.803689000
H	-1.849580000	-3.290463000	1.996984000
N	-1.064286000	-3.858625000	-0.083864000
H	-0.308792000	-4.374827000	-0.531316000
H	-1.001279000	-2.883355000	-0.431641000
H	-1.944095000	-4.244516000	-0.421029000
C	5.018015000	-2.255035000	-1.898420000
H	5.261421000	-3.215228000	-1.425724000
H	5.394576000	-2.297549000	-2.926442000
H	5.580961000	-1.480263000	-1.368875000

BCDB

N	0.612463000	-0.000002000	-0.000022000
H	1.165998000	-0.844151000	0.000191000
H	1.165890000	0.844224000	-0.000044000
B	-0.779518000	-0.000004000	-0.000022000
H	-1.360745000	-1.045687000	-0.000035000
H	-1.360798000	1.045648000	0.000155000

Int2

Co	1.486378000	-0.452298000	-0.640327000
P	-0.552854000	0.118642000	-0.083098000
N	-0.424160000	-2.633201000	-1.666831000
C	3.334529000	0.670996000	-0.664114000
C	3.519819000	-0.722667000	-1.027782000
C	-1.698982000	-2.094814000	-1.498245000
C	2.839613000	0.711266000	0.655430000
C	-3.250934000	-0.413665000	-0.679982000
C	-1.927716000	-0.859271000	-0.841556000
C	2.704838000	-0.653870000	1.130787000
C	-0.909789000	1.880775000	-0.515269000
C	-0.963256000	-0.003314000	1.714509000
C	-2.821800000	-2.830099000	-1.934010000
H	-2.658970000	-3.782891000	-2.433162000
C	3.184666000	-1.524019000	0.108332000
C	-4.358776000	-1.135014000	-1.118329000
C	3.635803000	1.854748000	-1.536153000
H	3.445242000	1.630156000	-2.589535000
H	4.686280000	2.164346000	-1.450290000
H	3.016744000	2.714874000	-1.264058000
C	2.294392000	-1.072885000	2.513324000
H	1.590959000	-0.368512000	2.961026000
H	3.163761000	-1.145010000	3.180628000
H	1.804490000	-2.050502000	2.503750000
C	-0.120362000	2.476373000	-1.512215000

H	0.661460000	1.886799000	-1.979969000
C	2.537442000	1.959815000	1.435532000
H	1.935573000	2.665660000	0.852655000
H	3.460611000	2.477172000	1.725951000
H	1.989766000	1.733087000	2.352043000
C	4.123890000	-1.207933000	-2.314073000
H	3.802375000	-2.226714000	-2.547513000
H	5.221029000	-1.210559000	-2.263051000
H	3.830224000	-0.571215000	-3.152464000
C	-4.113973000	-2.366603000	-1.744562000
H	-4.950262000	-2.966493000	-2.096733000
C	-1.398060000	-1.237168000	2.223984000
H	-1.604704000	-2.057103000	1.542506000
C	-0.333139000	3.803658000	-1.887486000
H	0.287299000	4.248962000	-2.660158000
C	-1.908935000	2.649788000	0.104385000
H	-2.510339000	2.224183000	0.901059000
C	3.275949000	-3.020903000	0.196936000
H	2.485767000	-3.431787000	0.831904000
H	4.238129000	-3.340491000	0.618868000
H	3.180341000	-3.486284000	-0.788286000
C	-0.705474000	1.043498000	2.612955000
H	-0.363904000	2.003569000	2.242517000
C	-1.570713000	-1.417996000	3.595961000
H	-1.907950000	-2.379536000	3.972675000
C	-1.332528000	4.557081000	-1.269211000
H	-1.494776000	5.591786000	-1.557756000
C	-2.118439000	3.977250000	-0.271046000
H	-2.893089000	4.558974000	0.220682000
C	-0.885881000	0.863081000	3.985795000
H	-0.687446000	1.686761000	4.665945000
C	-1.315602000	-0.368768000	4.482101000
H	-1.452423000	-0.509856000	5.550318000
H	-3.424666000	0.535669000	-0.184764000
H	-0.398394000	-3.432970000	-2.286195000
H	1.096663000	-0.335496000	-2.099413000
H	0.292550000	-1.947756000	-1.917543000
C	-5.765535000	-0.615521000	-0.939814000
H	-6.215183000	-0.335882000	-1.900659000
H	-6.418024000	-1.371006000	-0.487317000
H	-5.785705000	0.269969000	-0.297631000

Int3

Co	-0.837689000	-1.343492000	-0.215739000
P	-0.627562000	0.760816000	0.249265000
N	0.014796000	-0.628069000	-2.296766000

C	-1.417432000	-3.108383000	0.909022000
C	-1.293020000	-3.410951000	-0.508573000
C	0.329615000	0.772202000	-2.360958000
C	-2.471739000	-2.185954000	1.074153000
C	0.601425000	2.898130000	-1.237638000
C	0.195645000	1.557589000	-1.203183000
C	-2.973838000	-1.855773000	-0.240447000
C	0.346889000	1.249482000	1.742210000
C	-2.140775000	1.811353000	0.419456000
C	0.822535000	1.360466000	-3.533723000
H	0.932648000	0.748739000	-4.425572000
C	-2.296929000	-2.674965000	-1.195436000
C	1.118514000	3.486750000	-2.393802000
C	-0.584927000	-3.722535000	1.997388000
H	0.449806000	-3.857523000	1.669596000
H	-0.971788000	-4.704575000	2.302735000
H	-0.565916000	-3.086085000	2.887187000
C	-4.140059000	-0.953714000	-0.529195000
H	-4.200847000	-0.128562000	0.184263000
H	-5.091866000	-1.500953000	-0.484810000
H	-4.064888000	-0.508507000	-1.525846000
C	1.003899000	0.236526000	2.453493000
H	0.941837000	-0.780053000	2.077341000
C	-2.968339000	-1.620617000	2.373939000
H	-2.153278000	-1.490802000	3.094012000
H	-3.718328000	-2.271934000	2.843666000
H	-3.435493000	-0.643153000	2.229500000
C	-0.391578000	-4.458593000	-1.094984000
H	-0.104113000	-4.214540000	-2.123345000
H	-0.872290000	-5.446845000	-1.117401000
H	0.528715000	-4.553867000	-0.513348000
C	1.208290000	2.697526000	-3.550645000
H	1.599322000	3.132125000	-4.467584000
C	-2.783775000	2.321969000	-0.719028000
H	-2.319834000	2.211569000	-1.694411000
C	1.727288000	0.536918000	3.609362000
H	2.229823000	-0.260110000	4.150487000
C	0.416031000	2.568651000	2.222120000
H	-0.135525000	3.358868000	1.722270000
C	-2.570366000	-2.706326000	-2.671052000
H	-2.846025000	-1.715570000	-3.049100000
H	-3.396131000	-3.385906000	-2.921347000
H	-1.695467000	-3.048931000	-3.234253000
C	-2.766241000	1.963914000	1.667767000
H	-2.286400000	1.577987000	2.561719000
C	-4.014786000	2.969686000	-0.612212000

H	-4.495742000	3.360264000	-1.504855000
C	1.809756000	1.852540000	4.065091000
H	2.374127000	2.086246000	4.963790000
C	1.150928000	2.869697000	3.368167000
H	1.197537000	3.894696000	3.725828000
C	-3.998563000	2.610158000	1.773775000
H	-4.465858000	2.719215000	2.748615000
C	-4.628382000	3.114412000	0.633753000
H	-5.588233000	3.616138000	0.716101000
H	0.514539000	3.503277000	-0.341396000
H	-0.448645000	-0.945248000	-3.142982000
H	0.600068000	-1.476118000	0.157647000
H	0.892479000	-1.148954000	-2.196844000
C	1.536796000	4.938232000	-2.412902000
H	2.465234000	5.079077000	-2.976282000
H	0.772136000	5.564994000	-2.888143000
H	1.693633000	5.322629000	-1.400893000
C	3.893754000	1.560188000	-0.179319000
C	3.428956000	0.625348000	-1.077567000
C	3.356042000	-0.744092000	-0.714759000
C	3.794473000	-1.138828000	0.590339000
C	4.281756000	-0.152835000	1.486038000
C	4.322930000	1.171259000	1.112486000
H	3.921742000	2.608092000	-0.462056000
H	3.093802000	0.913334000	-2.066862000
C	3.703645000	-2.512966000	0.926349000
H	4.601140000	-0.458133000	2.478837000
H	4.670771000	1.923603000	1.813538000
C	3.212741000	-3.403078000	0.001611000
C	2.801491000	-2.910801000	-1.261322000
H	4.020597000	-2.843410000	1.912600000
H	3.125063000	-4.461888000	0.222286000
H	2.388913000	-3.601243000	-1.994098000
N	2.862040000	-1.643562000	-1.620841000

TS1

Co	-0.215418000	1.522705000	0.095827000
P	-0.961463000	-0.525345000	-0.426828000
N	0.336817000	0.549886000	1.855024000
C	0.168119000	3.615671000	0.253424000
C	-0.859097000	3.323188000	1.195897000
C	-0.523572000	-0.470245000	2.256689000
C	-0.349281000	3.305719000	-1.063275000
C	-1.959389000	-2.337276000	1.595727000
C	-1.183542000	-1.222393000	1.250220000
C	-1.681022000	2.834447000	-0.915501000

C	0.197674000	-1.671343000	-1.302992000
C	-2.550584000	-0.872823000	-1.286446000
C	-0.724719000	-0.852574000	3.600806000
H	-0.238785000	-0.283329000	4.390325000
C	-1.979028000	2.783267000	0.491182000
C	-2.136592000	-2.727731000	2.924668000
C	1.488281000	4.270615000	0.538535000
H	1.864272000	4.008490000	1.532778000
H	1.413775000	5.365875000	0.498439000
H	2.244584000	3.966583000	-0.190338000
C	-2.607074000	2.473293000	-2.039164000
H	-2.074698000	1.967936000	-2.850222000
H	-3.078327000	3.369961000	-2.463205000
H	-3.403853000	1.802525000	-1.713006000
C	1.326433000	-1.158345000	-1.954434000
H	1.511327000	-0.089670000	-1.911644000
C	0.364768000	3.548562000	-2.361068000
H	1.442174000	3.401126000	-2.251122000
H	0.200487000	4.571634000	-2.726025000
H	0.017217000	2.861345000	-3.137456000
C	-0.787786000	3.499168000	2.683078000
H	-1.264998000	2.663346000	3.205853000
H	-1.301190000	4.415570000	3.002387000
H	0.246523000	3.571915000	3.033922000
C	-1.510568000	-1.953196000	3.918360000
H	-1.643147000	-2.224946000	4.963764000
C	-3.774802000	-0.750605000	-0.606940000
H	-3.779049000	-0.576391000	0.463720000
C	2.217598000	-2.012362000	-2.607535000
H	3.097819000	-1.599728000	-3.091943000
C	-0.026772000	-3.057727000	-1.323224000
H	-0.897121000	-3.473146000	-0.825070000
C	-3.285191000	2.393957000	1.123731000
H	-3.908091000	1.823417000	0.432023000
H	-3.861753000	3.275446000	1.434961000
H	-3.128786000	1.774838000	2.013754000
C	-2.570948000	-1.102302000	-2.672044000
H	-1.636045000	-1.203876000	-3.215196000
C	-4.983742000	-0.856932000	-1.294737000
H	-5.920347000	-0.765169000	-0.751540000
C	1.988335000	-3.388275000	-2.619589000
H	2.684890000	-4.053593000	-3.122329000
C	0.862678000	-3.910190000	-1.975633000
H	0.678837000	-4.981197000	-1.980684000
C	-3.780883000	-1.205306000	-3.358366000
H	-3.776949000	-1.384840000	-4.429949000

C	-4.991981000	-1.080389000	-2.673803000
H	-5.933646000	-1.159748000	-3.209273000
H	-2.474367000	-2.888735000	0.812846000
H	0.538330000	1.177030000	2.630885000
H	1.070053000	1.207565000	-0.528329000
H	1.542524000	-0.086590000	1.471613000
C	-2.989310000	-3.918847000	3.294091000
H	-2.419065000	-4.661182000	3.865749000
H	-3.844688000	-3.626139000	3.915321000
H	-3.383671000	-4.418410000	2.404102000
C	4.712783000	1.873288000	-0.144087000
C	3.622612000	1.271314000	0.465157000
C	3.555934000	-0.134089000	0.549655000
C	4.609431000	-0.941610000	0.015116000
C	5.706053000	-0.289321000	-0.602893000
C	5.755385000	1.091980000	-0.681225000
H	4.758370000	2.956327000	-0.210679000
H	2.796990000	1.854236000	0.852240000
C	4.491814000	-2.352388000	0.123643000
H	6.509012000	-0.895533000	-1.013686000
H	6.601859000	1.577222000	-1.158248000
C	3.370407000	-2.909249000	0.728706000
C	2.374382000	-2.071415000	1.223266000
H	5.277618000	-2.983642000	-0.280272000
H	3.247805000	-3.983378000	0.806234000
H	1.468865000	-2.451264000	1.681741000
N	2.470700000	-0.732738000	1.140501000

Int4

Co	0.533010000	-1.444263000	0.396490000
P	0.960914000	0.571197000	-0.346204000
N	0.210178000	-0.595538000	2.103291000
C	0.145655000	-3.306107000	-0.502144000
C	0.360872000	-3.458569000	0.904356000
C	0.449568000	0.727520000	2.300073000
C	1.330610000	-2.738098000	-1.062948000
C	0.938673000	2.920372000	1.291040000
C	0.787102000	1.532628000	1.174939000
C	2.326539000	-2.633646000	-0.014569000
C	-0.138769000	1.305226000	-1.624009000
C	2.653699000	0.943808000	-0.964807000
C	0.310512000	1.399465000	3.546742000
H	0.049490000	0.822550000	4.431204000
C	1.723534000	-3.062581000	1.188058000
C	0.781740000	3.572327000	2.511972000
C	-1.084385000	-3.689721000	-1.270244000

H	-1.989016000	-3.572973000	-0.672895000
H	-1.020748000	-4.734145000	-1.601224000
H	-1.204710000	-3.065537000	-2.158754000
C	3.754391000	-2.212344000	-0.196221000
H	3.874105000	-1.535863000	-1.043749000
H	4.394395000	-3.086050000	-0.374189000
H	4.137552000	-1.694078000	0.686200000
C	-1.344316000	0.671569000	-1.953154000
H	-1.594632000	-0.275741000	-1.488457000
C	1.551822000	-2.459506000	-2.521885000
H	0.661764000	-2.021290000	-2.982073000
H	1.789645000	-3.381405000	-3.067531000
H	2.379685000	-1.764544000	-2.672037000
C	-0.596849000	-4.035371000	1.904044000
H	-0.611173000	-3.451349000	2.830150000
H	-0.322673000	-5.063150000	2.173376000
H	-1.615563000	-4.056549000	1.511108000
C	0.471203000	2.771695000	3.633853000
H	0.345033000	3.252659000	4.602121000
C	3.679358000	1.216241000	-0.046663000
H	3.440390000	1.308894000	1.008317000
C	-2.238695000	1.267125000	-2.843403000
H	-3.180123000	0.772001000	-3.059181000
C	0.165670000	2.542352000	-2.218489000
H	1.104288000	3.039270000	-1.992868000
C	2.359634000	-3.104954000	2.544159000
H	3.242264000	-2.463226000	2.592768000
H	2.669441000	-4.125622000	2.800669000
H	1.664408000	-2.769925000	3.319120000
C	2.972526000	0.841199000	-2.328409000
H	2.187349000	0.651164000	-3.053429000
C	4.995164000	1.374811000	-0.482312000
H	5.778361000	1.588806000	0.239452000
C	-1.931664000	2.497374000	-3.424162000
H	-2.629347000	2.964259000	-4.113643000
C	-0.726168000	3.132405000	-3.112592000
H	-0.481242000	4.089843000	-3.563967000
C	4.289979000	0.997685000	-2.761699000
H	4.521363000	0.918530000	-3.820160000
C	5.305466000	1.260845000	-1.839198000
H	6.330493000	1.382904000	-2.177043000
H	1.179579000	3.503443000	0.405204000
H	0.074886000	-1.131575000	2.952362000
H	-0.819338000	-1.137665000	0.020598000
H	-1.772156000	0.112224000	1.656961000
C	0.949948000	5.066855000	2.649552000

H	0.075561000	5.530540000	3.121975000
H	1.820132000	5.324473000	3.267007000
H	1.088184000	5.542279000	1.673625000
C	-4.421900000	-2.330578000	-0.053357000
C	-3.487807000	-1.564538000	0.657046000
C	-3.507750000	-0.169811000	0.569386000
C	-4.489702000	0.498063000	-0.230066000
C	-5.407456000	-0.307768000	-0.940449000
C	-5.374791000	-1.700188000	-0.855963000
H	-4.403007000	-3.413702000	0.028396000
H	-2.734353000	-2.038202000	1.277680000
C	-4.479835000	1.925319000	-0.278166000
H	-6.155222000	0.184337000	-1.557385000
H	-6.098036000	-2.291625000	-1.410372000
C	-3.506455000	2.640076000	0.445975000
C	-2.561115000	1.979627000	1.190529000
H	-5.217992000	2.442237000	-0.882203000
H	-3.482363000	3.724340000	0.417055000
H	-1.779994000	2.471656000	1.752182000
N	-2.572709000	0.596977000	1.252507000

$TS2_{C1}$

Co	-1.446163000	-0.886026000	-0.505657000
P	-0.271661000	0.741849000	0.377265000
N	-0.577458000	-0.378917000	-2.175542000
C	-2.599933000	-2.565999000	0.111570000
C	-2.851327000	-2.250757000	-1.257154000
C	0.137149000	0.778643000	-2.283278000
C	-2.962526000	-1.421632000	0.887995000
C	1.245955000	2.655706000	-1.144120000
C	0.474467000	1.486797000	-1.093964000
C	-3.574029000	-0.448098000	0.004644000
C	1.017700000	0.276550000	1.601699000
C	-1.135227000	2.126325000	1.233146000
C	0.635742000	1.306323000	-3.505920000
H	0.402087000	0.792671000	-4.436158000
C	-3.489796000	-0.947923000	-1.307159000
C	1.744827000	3.156690000	-2.345048000
C	-2.040958000	-3.849553000	0.650450000
H	-1.500124000	-4.405274000	-0.119445000
H	-2.839700000	-4.500627000	1.027352000
H	-1.341916000	-3.666202000	1.471031000
C	-4.220468000	0.830067000	0.448707000
H	-3.747747000	1.230240000	1.347606000
H	-5.281984000	0.667876000	0.677710000
H	-4.160236000	1.605348000	-0.318756000

C	0.776623000	-0.847880000	2.401977000
H	-0.096719000	-1.457810000	2.201067000
C	-2.947417000	-1.324652000	2.387002000
H	-2.232395000	-2.023442000	2.828691000
H	-3.934560000	-1.559452000	2.806300000
H	-2.681692000	-0.318345000	2.721675000
C	-2.633215000	-3.141225000	-2.444731000
H	-2.327316000	-2.564841000	-3.323837000
H	-3.548364000	-3.681862000	-2.718436000
H	-1.856529000	-3.886636000	-2.253482000
C	1.416579000	2.451266000	-3.521480000
H	1.782695000	2.822907000	-4.476898000
C	-1.727603000	3.149308000	0.476326000
H	-1.585267000	3.169113000	-0.599744000
C	1.667282000	-1.198745000	3.417618000
H	1.470817000	-2.074921000	4.029023000
C	2.180336000	1.031092000	1.814171000
H	2.406634000	1.878425000	1.178571000
C	-3.949273000	-0.274514000	-2.565153000
H	-4.253274000	0.757825000	-2.377114000
H	-4.802239000	-0.800479000	-3.011427000
H	-3.150089000	-0.249107000	-3.313244000
C	-1.318021000	2.117966000	2.624766000
H	-0.851646000	1.343967000	3.226772000
C	-2.489767000	4.138257000	1.097842000
H	-2.938803000	4.926330000	0.499771000
C	2.816335000	-0.437029000	3.629944000
H	3.517626000	-0.713978000	4.412089000
C	3.075523000	0.670758000	2.818943000
H	3.985108000	1.246867000	2.958429000
C	-2.087352000	3.105036000	3.243377000
H	-2.219734000	3.086502000	4.321557000
C	-2.677316000	4.116205000	2.481903000
H	-3.273933000	4.884992000	2.964442000
H	1.439010000	3.204819000	-0.225700000
H	-0.925626000	-0.722926000	-3.064307000
H	-0.258587000	-1.792061000	-0.142688000
H	1.216554000	-1.300174000	-2.025240000
C	2.596723000	4.402546000	-2.398576000
H	3.616674000	4.181824000	-2.738779000
H	2.185025000	5.148677000	-3.089137000
H	2.675638000	4.872506000	-1.413363000
C	4.685754000	0.370327000	-0.917269000
C	3.512722000	-0.160084000	-1.451909000
C	2.950786000	-1.315196000	-0.891683000
C	3.561521000	-1.937940000	0.234392000

C	4.753955000	-1.388171000	0.735038000
C	5.316974000	-0.247274000	0.168364000
H	5.106483000	1.271827000	-1.353551000
H	3.017100000	0.323438000	-2.288006000
C	2.905271000	-3.078172000	0.824279000
H	5.220339000	-1.859739000	1.596100000
H	6.234524000	0.166828000	0.575867000
C	1.691420000	-3.507430000	0.337924000
C	1.073292000	-2.845469000	-0.743576000
H	3.367550000	-3.563300000	1.678226000
H	1.173273000	-4.346502000	0.791194000
H	0.294013000	-3.323892000	-1.320492000
N	1.809231000	-1.888103000	-1.435347000

TS1'

Co	-1.353907000	-0.991765000	-0.429101000
P	-0.267328000	0.743044000	0.381566000
N	-0.179436000	-0.446121000	-2.280833000
C	-2.714746000	-2.519676000	0.369625000
C	-2.688722000	-2.524305000	-1.084296000
C	0.359116000	0.871885000	-2.293313000
C	-3.195440000	-1.266707000	0.788588000
C	1.091476000	2.826537000	-1.047506000
C	0.503185000	1.553478000	-1.069736000
C	-3.485664000	-0.479733000	-0.404847000
C	1.044865000	0.366193000	1.609496000
C	-1.161248000	2.143166000	1.194615000
C	0.784852000	1.502746000	-3.471249000
H	0.673822000	0.985203000	-4.420890000
C	-3.238873000	-1.289559000	-1.541964000
C	1.543162000	3.447481000	-2.213802000
C	-2.286821000	-3.660786000	1.245830000
H	-1.441804000	-4.201311000	0.810295000
H	-3.097558000	-4.386491000	1.391940000
H	-1.974638000	-3.311523000	2.234236000
C	-4.156291000	0.861719000	-0.413871000
H	-3.913505000	1.444638000	0.475934000
H	-5.248773000	0.750677000	-0.448428000
H	-3.860733000	1.453946000	-1.285252000
C	0.749363000	-0.624152000	2.558064000
H	-0.172686000	-1.190707000	2.459000000
C	-3.424154000	-0.804646000	2.197681000
H	-2.823683000	-1.377743000	2.910468000
H	-4.476375000	-0.917419000	2.491500000
H	-3.163734000	0.250093000	2.321386000
C	-2.326643000	-3.694827000	-1.952920000

H	-1.758913000	-3.384473000	-2.836603000
H	-3.221140000	-4.223400000	-2.306896000
H	-1.716519000	-4.423767000	-1.413523000
C	1.364470000	2.766907000	-3.428465000
H	1.690182000	3.234824000	-4.354539000
C	-1.810649000	3.110448000	0.410187000
H	-1.725283000	3.073603000	-0.670960000
C	1.640840000	-0.894949000	3.596407000
H	1.403280000	-1.664003000	4.325908000
C	2.263893000	1.052766000	1.689245000
H	2.530980000	1.783191000	0.934496000
C	-3.452360000	-0.885723000	-2.971476000
H	-3.168532000	0.158813000	-3.139257000
H	-4.503498000	-0.989867000	-3.270436000
H	-2.864185000	-1.506679000	-3.654416000
C	-1.288866000	2.212290000	2.590513000
H	-0.788025000	1.481969000	3.217580000
C	-2.564396000	4.121080000	1.006273000
H	-3.056619000	4.862372000	0.383044000
C	2.846095000	-0.196095000	3.678975000
H	3.547782000	-0.413775000	4.479248000
C	3.160052000	0.767416000	2.717429000
H	4.112607000	1.286431000	2.757337000
C	-2.050076000	3.220367000	3.185044000
H	-2.136153000	3.259173000	4.267276000
C	-2.689844000	4.177796000	2.396105000
H	-3.278729000	4.963701000	2.860096000
H	1.161326000	3.362408000	-0.104681000
H	-0.620227000	-0.671907000	-3.167961000
H	-0.035092000	-1.982840000	-0.113864000
H	0.637457000	-1.133304000	-2.129617000
C	2.205477000	4.804215000	-2.173642000
H	3.296194000	4.713791000	-2.249008000
H	1.875467000	5.436729000	-3.004390000
H	1.983978000	5.330183000	-1.240272000
C	4.776309000	-0.053640000	-1.216653000
C	3.591756000	-0.594600000	-1.682454000
C	2.889752000	-1.574754000	-0.928819000
C	3.457528000	-1.988078000	0.322594000
C	4.679247000	-1.441704000	0.753679000
C	5.337559000	-0.477976000	0.005501000
H	5.283810000	0.706839000	-1.805215000
H	3.161628000	-0.268691000	-2.624851000
C	2.732692000	-2.974272000	1.075632000
H	5.090044000	-1.773969000	1.704678000
H	6.274234000	-0.053602000	0.354559000

C	1.517588000	-3.398407000	0.656057000
C	0.927915000	-2.823050000	-0.562200000
H	3.178994000	-3.370374000	1.985129000
H	0.944389000	-4.128247000	1.220395000
H	0.240019000	-3.487695000	-1.086087000
N	1.741040000	-2.107867000	-1.424463000

1,2-DHQ

C	2.469637000	-0.723996000	0.041383000
C	1.248391000	-1.394832000	-0.031616000
C	0.045890000	-0.675163000	-0.071155000
C	0.084426000	0.742780000	-0.038362000
C	1.321254000	1.391118000	0.031838000
C	2.516128000	0.671613000	0.076897000
H	3.390129000	-1.300355000	0.074250000
H	1.222662000	-2.481870000	-0.061533000
C	-1.186379000	1.458458000	-0.108151000
H	1.338125000	2.478428000	0.052439000
H	3.467709000	1.190298000	0.134907000
C	-2.358640000	0.815911000	-0.008583000
C	-2.417453000	-0.672537000	0.230359000
H	-1.156915000	2.537450000	-0.241685000
H	-3.301640000	1.353560000	-0.044304000
H	-3.254027000	-1.115208000	-0.325331000
N	-1.180816000	-1.309269000	-0.218274000
H	-1.156562000	-2.312380000	-0.099998000
H	-2.623297000	-0.865151000	1.303511000

$TS1_C$

Co	0.901847000	1.276487000	0.017415000
P	0.707092000	-0.867086000	-0.138101000
N	-0.645972000	0.247272000	-2.649417000
C	1.939320000	2.642848000	1.403073000
C	1.142298000	3.349926000	0.428573000
C	-1.391452000	-0.726548000	-1.997751000
C	2.968004000	1.949430000	0.717976000
C	-1.774221000	-2.272448000	-0.161694000
C	-0.933745000	-1.360032000	-0.809964000
C	2.813622000	2.200135000	-0.693302000
C	0.905360000	-1.778937000	1.453984000
C	1.902194000	-1.800286000	-1.209682000
C	-2.658080000	-1.074956000	-2.493873000
H	-3.019817000	-0.593678000	-3.398853000
C	1.705326000	3.086463000	-0.864784000
C	-3.044569000	-2.601858000	-0.642445000
C	1.740904000	2.720688000	2.890028000

H	0.676882000	2.700984000	3.150230000
H	2.156777000	3.648383000	3.307376000
H	2.231035000	1.888532000	3.405386000
C	3.729525000	1.704517000	-1.776993000
H	4.031430000	0.665958000	-1.612819000
H	4.645604000	2.307358000	-1.845741000
H	3.244246000	1.744451000	-2.757104000
C	1.027918000	-1.029372000	2.630628000
H	0.995865000	0.053663000	2.556260000
C	4.052772000	1.133919000	1.365988000
H	3.649279000	0.409078000	2.083019000
H	4.763591000	1.767528000	1.912643000
H	4.629847000	0.578606000	0.621906000
C	0.046718000	4.319307000	0.772527000
H	-0.480636000	4.666124000	-0.120787000
H	0.444562000	5.209593000	1.277592000
H	-0.695903000	3.863808000	1.437013000
C	-3.464016000	-1.990834000	-1.828256000
H	-4.445625000	-2.222496000	-2.234564000
C	1.536000000	-2.439315000	-2.401770000
H	0.494248000	-2.468328000	-2.704470000
C	1.210600000	-1.664261000	3.860849000
H	1.309504000	-1.071303000	4.766010000
C	0.991334000	-3.178376000	1.522019000
H	0.927191000	-3.767613000	0.611722000
C	1.226784000	3.668134000	-2.166574000
H	1.437646000	2.998662000	-3.006527000
H	1.709114000	4.629628000	-2.388046000
H	0.146022000	3.841139000	-2.155421000
C	3.253674000	-1.806982000	-0.825602000
H	3.547404000	-1.324765000	0.101184000
C	2.503434000	-3.058666000	-3.198623000
H	2.204110000	-3.551411000	-4.119763000
C	1.274735000	-3.057268000	3.922864000
H	1.418110000	-3.554380000	4.878196000
C	1.168091000	-3.813226000	2.751221000
H	1.230061000	-4.896970000	2.795520000
C	4.215509000	-2.433192000	-1.615458000
H	5.255391000	-2.433410000	-1.300438000
C	3.843384000	-3.056318000	-2.810630000
H	4.592838000	-3.540204000	-3.430386000
H	-1.435848000	-2.734074000	0.759894000
H	0.345236000	0.313412000	-2.465301000
H	-0.591970000	1.301236000	0.886786000
H	-0.911858000	0.470651000	-3.595716000
C	-3.935804000	-3.549045000	0.124157000

H	-4.387704000	-3.047930000	0.989454000
H	-4.750390000	-3.929190000	-0.500135000
H	-3.376276000	-4.409538000	0.506750000
C	-4.748821000	1.661050000	-1.569332000
C	-4.973239000	0.834697000	-0.487251000
C	-3.174865000	-0.217129000	2.614278000
C	-4.176334000	-0.155485000	1.661114000
C	-2.560343000	2.246556000	-0.707474000
C	-3.528347000	2.360890000	-1.686565000
H	-5.505069000	1.761382000	-2.342619000
H	-5.898654000	0.270708000	-0.404877000
C	-3.996420000	0.685893000	0.528635000
H	-1.604148000	2.747825000	-0.795033000
H	-3.345403000	2.987084000	-2.554949000
C	-2.782872000	1.427282000	0.417310000
N	-1.807147000	1.371833000	1.407873000
H	-5.092451000	-0.728569000	1.764392000
C	-2.022251000	0.561926000	2.465483000
H	-1.239436000	0.553361000	3.216125000
H	-3.281970000	-0.832671000	3.502095000

Int3$_C$

Co	0.856836000	1.274335000	0.086848000
P	0.842461000	-0.936206000	-0.133252000
N	-0.228728000	1.039101000	-1.946988000
C	1.575736000	2.451435000	1.858784000
C	0.994859000	3.323663000	0.865428000
C	-1.070839000	-0.127811000	-1.927098000
C	2.729899000	1.847002000	1.288354000
C	-1.550842000	-2.307691000	-0.990411000
C	-0.692631000	-1.210970000	-1.106017000
C	2.866487000	2.310922000	-0.061709000
C	0.808167000	-2.143382000	1.251840000
C	2.182881000	-1.601751000	-1.212431000
C	-2.280009000	-0.172415000	-2.619820000
H	-2.579183000	0.671124000	-3.234446000
C	1.805663000	3.237011000	-0.312777000
C	-2.782462000	-2.347415000	-1.653355000
C	1.118819000	2.328104000	3.285830000
H	0.025753000	2.335120000	3.368031000
H	1.490438000	3.152237000	3.911587000
H	1.476578000	1.398340000	3.740469000
C	3.964201000	1.938099000	-1.020161000
H	4.240226000	0.882587000	-0.931373000
H	4.875908000	2.531096000	-0.861810000
H	3.656398000	2.098705000	-2.058833000

C	0.666340000	-1.630425000	2.548555000
H	0.602383000	-0.554574000	2.681472000
C	3.643731000	0.877888000	1.985971000
H	3.083895000	0.108194000	2.530184000
H	4.294806000	1.379497000	2.714357000
H	4.295080000	0.365800000	1.272524000
C	-0.061880000	4.361235000	1.141215000
H	-0.591429000	4.665853000	0.231620000
H	0.376970000	5.273102000	1.569955000
H	-0.814262000	4.008362000	1.854935000
C	-3.127099000	-1.268837000	-2.475944000
H	-4.083375000	-1.271810000	-2.991792000
C	2.021968000	-1.744789000	-2.598539000
H	1.045443000	-1.591144000	-3.049795000
C	0.622869000	-2.487934000	3.649537000
H	0.514828000	-2.078883000	4.650084000
C	0.934919000	-3.530072000	1.074365000
H	1.081937000	-3.935074000	0.077064000
C	1.590632000	4.012463000	-1.582490000
H	1.894655000	3.436567000	-2.464301000
H	2.163768000	4.949435000	-1.602491000
H	0.536268000	4.281413000	-1.713632000
C	3.448296000	-1.842767000	-0.650337000
H	3.588032000	-1.741887000	0.421739000
C	3.103383000	-2.107761000	-3.404780000
H	2.961025000	-2.218626000	-4.476337000
C	0.726485000	-3.867677000	3.461677000
H	0.693102000	-4.537226000	4.316470000
C	0.887569000	-4.387375000	2.173745000
H	0.981554000	-5.459810000	2.027815000
C	4.524042000	-2.212645000	-1.455733000
H	5.494847000	-2.398192000	-1.004595000
C	4.357250000	-2.340404000	-2.837754000
H	5.197080000	-2.623808000	-3.465350000
H	-1.282155000	-3.126332000	-0.330105000
H	0.591966000	0.926832000	-2.538710000
H	-1.379564000	2.334560000	0.374695000
H	-0.741086000	1.859158000	-2.257971000
C	-3.744607000	-3.484917000	-1.417090000
H	-4.362444000	-3.270638000	-0.536531000
H	-4.416668000	-3.630993000	-2.268279000
H	-3.219662000	-4.426846000	-1.230407000
C	-4.078733000	-1.308407000	1.785429000
C	-4.767979000	-0.571779000	0.821649000
C	-4.204885000	2.482332000	-1.316298000
C	-4.858396000	1.364598000	-0.772589000

C	-2.239095000	0.258252000	1.672322000
C	-2.811700000	-0.898484000	2.208433000
H	-4.529292000	-2.203606000	2.204539000
H	-5.751200000	-0.893936000	0.488192000
C	-4.213185000	0.589319000	0.246285000
H	-1.248093000	0.585731000	1.975339000
H	-2.259442000	-1.476330000	2.942526000
C	-2.920509000	0.992106000	0.699515000
N	-2.347882000	2.131254000	0.143477000
H	-5.840375000	1.067775000	-1.124716000
C	-2.954040000	2.850912000	-0.872789000
H	-2.412798000	3.719397000	-1.226007000
H	-4.678958000	3.074598000	-2.092526000

TS1$_D$

Co	-0.020537000	-1.218867000	-0.480180000
P	-1.248413000	0.370761000	0.371322000
N	-0.546411000	-0.489892000	-2.171421000
C	0.767758000	-2.520931000	0.988228000
C	1.573485000	-2.591921000	-0.193058000
C	-1.133537000	0.732768000	-2.282588000
C	-0.554139000	-3.000444000	0.656617000
C	-2.214952000	2.619385000	-1.130354000
C	-1.552893000	1.387517000	-1.091867000
C	-0.585731000	-3.292866000	-0.728691000
C	-0.568697000	1.422502000	1.705614000
C	-2.944652000	-0.104599000	0.924422000
C	-1.364855000	1.419208000	-3.504145000
H	-1.042036000	0.962576000	-4.437552000
C	0.729133000	-3.008303000	-1.262202000
C	-2.446184000	3.282948000	-2.335102000
C	1.267589000	-2.205053000	2.367731000
H	2.004808000	-1.399585000	2.348635000
H	1.740178000	-3.083614000	2.826590000
H	0.453944000	-1.892796000	3.025857000
C	-1.750720000	-3.773512000	-1.542379000
H	-2.671388000	-3.785163000	-0.954436000
H	-1.585679000	-4.788595000	-1.923560000
H	-1.915857000	-3.115882000	-2.403175000
C	-0.479028000	0.914911000	3.012249000
H	-0.892905000	-0.061893000	3.242840000
C	-1.659839000	-3.198900000	1.651226000
H	-1.725645000	-2.365106000	2.355547000
H	-1.486992000	-4.111599000	2.235029000
H	-2.634907000	-3.283665000	1.169725000
C	3.045763000	-2.330227000	-0.286709000

H	3.305371000	-1.813976000	-1.213961000
H	3.603882000	-3.275504000	-0.262099000
H	3.399005000	-1.713017000	0.540028000
C	-2.000044000	2.650764000	-3.514607000
H	-2.163179000	3.147573000	-4.469481000
C	-3.632894000	-0.974502000	0.060594000
H	-3.134800000	-1.326545000	-0.838459000
C	0.135891000	1.655375000	4.021715000
H	0.197918000	1.249311000	5.027309000
C	-0.015617000	2.680147000	1.424910000
H	-0.037548000	3.060875000	0.411582000
C	1.153090000	-3.201929000	-2.687692000
H	0.301067000	-3.125427000	-3.369868000
H	1.601495000	-4.191655000	-2.839599000
H	1.895304000	-2.454234000	-2.983083000
C	-3.586646000	0.356707000	2.079273000
H	-3.082036000	1.046315000	2.747460000
C	-4.931013000	-1.383246000	0.353863000
H	-5.452072000	-2.054468000	-0.323276000
C	0.673013000	2.913244000	3.735782000
H	1.156451000	3.489625000	4.519239000
C	0.595187000	3.420970000	2.437912000
H	1.025894000	4.390560000	2.206122000
C	-4.887188000	-0.063106000	2.376724000
H	-5.374102000	0.298187000	3.278341000
C	-5.559769000	-0.933854000	1.519963000
H	-6.569828000	-1.257625000	1.754315000
H	-2.547824000	3.072613000	-0.198602000
H	-0.227761000	-0.883464000	-3.049340000
H	0.990758000	-0.107575000	-0.378396000
H	1.408272000	0.622681000	-2.490417000
C	-3.137603000	4.624590000	-2.388359000
H	-2.482386000	5.404976000	-2.796269000
H	-4.031804000	4.595609000	-3.023348000
H	-3.452715000	4.948966000	-1.391736000
C	4.212104000	0.694159000	2.286077000
C	3.102067000	0.943152000	1.496916000
C	3.206619000	0.990820000	0.077601000
C	4.499279000	0.765017000	-0.511686000
C	5.604301000	0.528120000	0.313483000
C	5.474874000	0.488627000	1.700964000
H	4.103890000	0.661518000	3.366881000
H	2.126799000	1.097314000	1.939236000
C	4.593874000	0.753235000	-1.959273000
H	6.575379000	0.359489000	-0.147105000
H	6.342917000	0.297902000	2.324781000

C	3.505398000	0.993575000	-2.710107000
C	2.177899000	1.300995000	-2.086423000
H	5.558102000	0.534789000	-2.413066000
H	3.557267000	0.976922000	-3.796640000
H	1.830633000	2.301931000	-2.403073000
N	2.081393000	1.230976000	-0.639677000

TS1$_E$

Co	1.332647000	-1.345197000	0.398381000
P	1.020036000	0.734113000	-0.269065000
N	0.885255000	-0.720279000	2.143218000
C	0.973605000	-3.234304000	-0.570677000
C	1.635615000	-3.405823000	0.684130000
C	0.811841000	0.630920000	2.417579000
C	1.812130000	-2.412009000	-1.379679000
C	0.623931000	2.923027000	1.555680000
C	0.779759000	1.549261000	1.335882000
C	3.057378000	-2.172879000	-0.664961000
C	-0.410260000	1.124432000	-1.357091000
C	2.432731000	1.596270000	-1.064983000
C	0.699113000	1.168506000	3.720832000
H	0.710159000	0.493142000	4.572791000
C	2.940469000	-2.778562000	0.601368000
C	0.506068000	3.445503000	2.843041000
C	-0.387255000	-3.764554000	-0.922888000
H	-1.111908000	-3.548572000	-0.131828000
H	-0.367045000	-4.851512000	-1.073444000
H	-0.761911000	-3.315857000	-1.847042000
C	4.246246000	-1.438040000	-1.212096000
H	3.948281000	-0.621205000	-1.873245000
H	4.897037000	-2.108687000	-1.788922000
H	4.848678000	-0.997745000	-0.413200000
C	-1.282505000	0.085970000	-1.713401000
H	-1.084168000	-0.916755000	-1.351553000
C	1.554063000	-1.994173000	-2.799792000
H	0.487095000	-1.873094000	-3.001937000
H	1.946088000	-2.737297000	-3.506185000
H	2.040108000	-1.041580000	-3.026284000
C	1.131476000	-4.203423000	1.850318000
H	1.452732000	-3.766639000	2.801378000
H	1.509781000	-5.233699000	1.822633000
H	0.040271000	-4.242189000	1.860397000
C	0.552924000	2.533810000	3.916090000
H	0.467483000	2.912985000	4.932772000
C	3.517367000	2.000304000	-0.270345000
H	3.453110000	1.905221000	0.809498000

C	-2.411504000	0.344270000	-2.490805000
H	-3.091249000	-0.465107000	-2.739025000
C	-0.679268000	2.426058000	-1.807644000
H	0.000423000	3.236897000	-1.563505000
C	3.949790000	-2.762730000	1.711156000
H	4.714700000	-2.000140000	1.544843000
H	4.457140000	-3.731220000	1.806622000
H	3.473166000	-2.547390000	2.672943000
C	2.522835000	1.731473000	-2.458888000
H	1.689098000	1.433401000	-3.086879000
C	4.666877000	2.525466000	-0.858829000
H	5.497565000	2.837213000	-0.231950000
C	-2.678274000	1.643491000	-2.923689000
H	-3.565506000	1.847547000	-3.515434000
C	-1.808583000	2.683351000	-2.584776000
H	-2.011089000	3.695534000	-2.923670000
C	3.676202000	2.256920000	-3.045393000
H	3.731658000	2.358044000	-4.125653000
C	4.751533000	2.652219000	-2.248013000
H	5.648450000	3.060235000	-2.705008000
H	0.612815000	3.601467000	0.705662000
H	1.065221000	-1.282800000	2.968653000
B	-1.373011000	-1.168196000	1.857001000
H	-1.256592000	-2.359966000	1.851969000
H	-1.640289000	-0.598647000	2.873503000
H	-1.265419000	-0.547430000	0.851275000
C	0.343735000	4.926612000	3.091101000
H	-0.590919000	5.147547000	3.621471000
H	1.160365000	5.327721000	3.704142000
H	0.330517000	5.488806000	2.152163000
C	-4.188765000	2.171330000	0.303869000
C	-3.627370000	1.052628000	0.876050000
C	-4.130337000	-0.236926000	0.555895000
C	-5.224965000	-0.354052000	-0.360822000
C	-5.784257000	0.822695000	-0.925454000
C	-5.274565000	2.059072000	-0.600475000
H	-3.789189000	3.152860000	0.539879000
H	-2.790363000	1.123483000	1.559103000
C	-5.690903000	-1.657662000	-0.668637000
H	-6.616858000	0.728294000	-1.618204000
H	-5.701079000	2.956028000	-1.040110000
C	-5.085155000	-2.748340000	-0.089316000
C	-4.005587000	-2.531483000	0.801883000
H	-6.520202000	-1.777668000	-1.361595000
H	-5.413036000	-3.760421000	-0.303428000
H	-3.494605000	-3.374473000	1.262639000

N	-3.545868000	-1.338079000	1.118371000

Int1$_E$

Co	-1.520189000	0.092982000	-0.490465000
P	0.801355000	-0.353577000	-0.108813000
N	-0.671469000	1.844427000	-1.423314000
C	-3.846029000	0.153348000	-0.372312000
C	-3.361376000	1.237739000	0.417144000
C	0.647835000	2.210920000	-1.082432000
C	-3.351228000	-1.062689000	0.200089000
C	2.841779000	1.633448000	-0.178950000
C	1.529614000	1.264782000	-0.512938000
C	-2.573937000	-0.725871000	1.357986000
C	1.468641000	-1.615063000	-1.255239000
C	1.500030000	-0.795114000	1.529587000
C	1.120125000	3.520709000	-1.271202000
H	0.455122000	4.263980000	-1.703821000
C	-2.575608000	0.700173000	1.485641000
C	3.315339000	2.929471000	-0.386084000
C	-4.709517000	0.271439000	-1.595152000
H	-4.437859000	1.146394000	-2.193798000
H	-5.772166000	0.364582000	-1.334946000
H	-4.605102000	-0.603541000	-2.242471000
C	-1.929457000	-1.704782000	2.299668000
H	-1.556651000	-2.587292000	1.769193000
H	-2.636404000	-2.060156000	3.060902000
H	-1.076135000	-1.263972000	2.820997000
C	0.655835000	-2.723987000	-1.535739000
H	-0.339270000	-2.784637000	-1.101236000
C	-3.647146000	-2.453571000	-0.288574000
H	-3.731897000	-2.486994000	-1.379276000
H	-4.588901000	-2.839628000	0.123704000
H	-2.859220000	-3.156201000	0.002012000
C	-3.626476000	2.695394000	0.173091000
H	-2.791567000	3.316651000	0.514895000
H	-4.522620000	3.044182000	0.703223000
H	-3.784620000	2.901383000	-0.890855000
C	2.421988000	3.867505000	-0.929123000
H	2.757094000	4.889833000	-1.089094000
C	1.521463000	0.176722000	2.544568000
H	1.231793000	1.198270000	2.318723000
C	1.110746000	-3.735509000	-2.382405000
H	0.474364000	-4.589334000	-2.595828000
C	2.734645000	-1.520873000	-1.848309000
H	3.359236000	-0.654012000	-1.658230000
C	-1.922975000	1.503797000	2.574986000

H	-1.156100000	0.924999000	3.095421000
H	-2.651305000	1.833904000	3.327619000
H	-1.441315000	2.404949000	2.178050000
C	1.884453000	-2.108308000	1.835997000
H	1.877269000	-2.870344000	1.062984000
C	1.917284000	-0.161134000	3.837710000
H	1.931523000	0.600802000	4.611828000
C	2.375424000	-3.637916000	-2.965812000
H	2.727654000	-4.419451000	-3.632822000
C	3.183204000	-2.528660000	-2.701735000
H	4.162361000	-2.446411000	-3.164693000
C	2.277720000	-2.443094000	3.133130000
H	2.575061000	-3.463701000	3.356663000
C	2.292960000	-1.473678000	4.137079000
H	2.598082000	-1.736955000	5.145583000
H	3.492420000	0.900513000	0.291906000
H	-1.272447000	2.660641000	-1.439114000
B	-0.951001000	0.911250000	-2.685825000
H	-1.732419000	1.443108000	-3.439432000
H	0.080997000	0.533174000	-3.181166000
H	-1.554030000	-0.149726000	-2.276579000
C	4.734166000	3.316295000	-0.042049000
H	5.342425000	3.443897000	-0.946058000
H	4.769423000	4.264274000	0.505767000
H	5.218253000	2.553785000	0.575298000

Int2$_E$

Co	-0.426599000	1.214344000	-0.142239000
P	1.312719000	-0.077760000	0.203196000
N	-0.437001000	-1.219127000	-2.219290000
C	-1.939197000	2.629538000	-0.305907000
C	-1.350165000	2.468015000	-1.602455000
C	0.776001000	-1.836173000	-1.981733000
C	-0.936927000	3.188747000	0.577367000
C	2.989946000	-2.009307000	-0.947788000
C	1.730726000	-1.375371000	-1.030699000
C	0.261961000	3.319391000	-0.158385000
C	1.152107000	-1.008969000	1.803888000
C	2.913650000	0.821463000	0.405340000
C	1.119544000	-2.991550000	-2.730945000
H	0.397380000	-3.362413000	-3.449166000
C	0.023230000	2.839800000	-1.504168000
C	3.326740000	-3.122937000	-1.708528000
C	-3.383007000	2.425185000	0.048280000
H	-3.852364000	1.685333000	-0.601376000
H	-3.950372000	3.361334000	-0.045593000

H	-3.492169000	2.069388000	1.075701000
C	1.538868000	3.929146000	0.340963000
H	1.690912000	3.735116000	1.405988000
H	1.527911000	5.018456000	0.202016000
H	2.412325000	3.539878000	-0.184362000
C	0.482697000	-0.359538000	2.854387000
H	0.075115000	0.632161000	2.680518000
C	-1.160902000	3.578971000	2.009406000
H	-1.864393000	2.897934000	2.497692000
H	-1.572849000	4.593857000	2.096619000
H	-0.227278000	3.556205000	2.579775000
C	-2.048906000	1.973424000	-2.837731000
H	-1.378090000	1.392002000	-3.476806000
H	-2.438596000	2.806888000	-3.437341000
H	-2.890930000	1.324167000	-2.585086000
C	2.346224000	-3.613640000	-2.591590000
H	2.566953000	-4.488373000	-3.200208000
C	3.659035000	1.164533000	-0.734733000
H	3.344629000	0.804763000	-1.709233000
C	0.295193000	-0.995228000	4.081275000
H	-0.222431000	-0.476832000	4.883833000
C	1.612063000	-2.317426000	2.003365000
H	2.114215000	-2.846116000	1.200816000
C	1.005506000	2.879598000	-2.640356000
H	2.034307000	2.797238000	-2.281947000
H	0.929905000	3.819799000	-3.203397000
H	0.833332000	2.061516000	-3.346555000
C	3.348634000	1.274559000	1.657906000
H	2.792442000	1.014207000	2.552631000
C	4.806840000	1.948723000	-0.623278000
H	5.372594000	2.202833000	-1.515226000
C	0.752465000	-2.302728000	4.269997000
H	0.594881000	-2.805462000	5.220200000
C	1.407073000	-2.961688000	3.227387000
H	1.760178000	-3.980314000	3.363016000
C	4.497766000	2.060243000	1.767621000
H	4.821954000	2.402398000	2.746491000
C	5.228062000	2.403294000	0.629111000
H	6.121246000	3.015111000	0.716139000
H	3.723213000	-1.621236000	-0.245576000
H	-0.471756000	-0.263177000	-1.873885000
B	-1.786432000	-1.886193000	-2.364889000
H	-1.721677000	-3.040052000	-2.710925000
H	-2.538549000	-1.224456000	-3.026796000
H	-1.271921000	0.313594000	0.695364000
C	-3.645540000	-1.403872000	-0.411736000

C	-1.652349000	-2.497579000	0.120777000
C	-4.569275000	-0.902906000	-1.365055000
C	-3.987565000	-1.340678000	0.978094000
C	-1.938602000	-2.498316000	1.494762000
H	-0.724109000	-2.920978000	-0.242681000
C	-5.759435000	-0.345150000	-0.950792000
H	-4.323282000	-0.968518000	-2.414673000
C	-5.214427000	-0.740585000	1.366129000
C	-3.096297000	-1.897740000	1.924521000
H	-1.221346000	-2.926990000	2.183928000
C	-6.085714000	-0.249894000	0.422565000
H	-6.455960000	0.032224000	-1.693392000
H	-5.452757000	-0.687691000	2.424838000
H	-3.339696000	-1.844340000	2.982005000
H	-7.023833000	0.204506000	0.725558000
N	-2.445648000	-1.962461000	-0.802246000
C	4.682475000	-3.779493000	-1.604176000
H	4.594820000	-4.855392000	-1.411279000
H	5.256357000	-3.667687000	-2.532654000
H	5.276823000	-3.342857000	-0.795795000

$TS2_{EC1}$

Co	-1.412059000	-0.969564000	-0.281509000
P	-0.269497000	0.784487000	0.373998000
N	-0.102920000	-0.665521000	-2.198175000
C	-2.775229000	-2.597382000	-0.526836000
C	-3.133731000	-1.625180000	-1.505478000
C	0.504927000	0.630801000	-2.260168000
C	-3.020630000	-2.032260000	0.788655000
C	1.244380000	2.672009000	-1.156232000
C	0.611635000	1.418475000	-1.100426000
C	-3.443796000	-0.703741000	0.608888000
C	0.979736000	0.477069000	1.692620000
C	-1.113570000	2.301620000	1.008196000
C	1.024511000	1.143284000	-3.461944000
H	0.949449000	0.538756000	-4.360961000
C	-3.465783000	-0.425236000	-0.821219000
C	1.788259000	3.166286000	-2.341299000
C	-2.414820000	-4.026293000	-0.809408000
H	-1.806257000	-4.114016000	-1.715230000
H	-3.311367000	-4.643012000	-0.956705000
H	-1.854083000	-4.470531000	0.018899000
C	-3.855391000	0.260658000	1.682557000
H	-3.334170000	0.064764000	2.624394000
H	-4.932964000	0.191338000	1.882622000
H	-3.640178000	1.294187000	1.404316000

C	0.616817000	-0.417828000	2.710845000
H	-0.325301000	-0.952579000	2.627605000
C	-2.834879000	-2.758066000	2.089428000
H	-1.929509000	-3.374011000	2.072892000
H	-3.679181000	-3.423526000	2.314182000
H	-2.739223000	-2.058675000	2.925478000
C	-3.114755000	-1.839290000	-2.990784000
H	-3.057107000	-0.890429000	-3.534074000
H	-4.025275000	-2.347393000	-3.334694000
H	-2.264044000	-2.457780000	-3.296218000
C	1.653607000	2.381961000	-3.499350000
H	2.048579000	2.752615000	-4.442769000
C	-1.768801000	3.150770000	0.100375000
H	-1.685303000	2.964437000	-0.965609000
C	1.466332000	-0.639229000	3.795375000
H	1.172983000	-1.332573000	4.578817000
C	2.221397000	1.122353000	1.761678000
H	2.538029000	1.783706000	0.963545000
C	-3.970822000	0.839782000	-1.454047000
H	-3.815562000	1.702535000	-0.802911000
H	-5.046821000	0.780710000	-1.667889000
H	-3.461301000	1.046454000	-2.400813000
C	-1.225181000	2.569092000	2.380323000
H	-0.723194000	1.931065000	3.100251000
C	-2.520987000	4.233617000	0.554147000
H	-3.019422000	4.880033000	-0.162851000
C	2.698096000	0.015533000	3.861648000
H	3.365611000	-0.161967000	4.700343000
C	3.076342000	0.887281000	2.838265000
H	4.044635000	1.377716000	2.870669000
C	-1.980107000	3.652872000	2.833239000
H	-2.055085000	3.845451000	3.899854000
C	-2.632921000	4.486242000	1.923953000
H	-3.220635000	5.328285000	2.277947000
H	1.277440000	3.290453000	-0.263089000
H	-0.793726000	-0.721418000	-2.941577000
B	0.890491000	-1.867645000	-2.472659000
H	1.693944000	-1.626777000	-3.346521000
H	0.216904000	-2.839261000	-2.749173000
H	-0.277289000	-1.845334000	0.314908000
C	2.825847000	-1.605618000	-0.696810000
C	0.896541000	-2.900008000	-0.227658000
C	3.518180000	-0.674695000	-1.504520000
C	3.389650000	-1.960211000	0.566913000
C	1.452362000	-3.329356000	1.023196000
H	0.110467000	-3.486567000	-0.677078000

C	4.716256000	-0.129875000	-1.076287000
H	3.096723000	-0.383358000	-2.455275000
C	4.623587000	-1.403424000	0.962502000
C	2.671442000	-2.869935000	1.405964000
H	0.864675000	-3.995936000	1.644804000
C	5.285491000	-0.495781000	0.157859000
H	5.222195000	0.592564000	-1.710530000
H	5.031415000	-1.687425000	1.928744000
H	3.113793000	-3.172461000	2.350854000
H	6.229540000	-0.063793000	0.475509000
N	1.638870000	-2.189027000	-1.114829000
C	2.494289000	4.500645000	-2.387513000
H	3.578917000	4.371834000	-2.489945000
H	2.160112000	5.101206000	-3.240513000
H	2.315843000	5.080054000	-1.476988000

$TS2_{EC3}$

Co	-0.792257000	1.062363000	0.156450000
P	1.382799000	-0.004563000	0.230182000
N	-0.255527000	-1.578434000	-2.058487000
C	-2.543159000	2.296133000	-0.554667000
C	-1.893673000	1.816494000	-1.732685000
C	1.035549000	-1.968835000	-1.808896000
C	-1.611104000	3.121805000	0.159947000
C	3.287090000	-1.707978000	-0.848397000
C	1.933936000	-1.295523000	-0.928083000
C	-0.398842000	3.180755000	-0.600578000
C	1.513638000	-0.715155000	1.928006000
C	2.737999000	1.249922000	0.173382000
C	1.550464000	-3.112351000	-2.483539000
H	0.879413000	-3.649203000	-3.143379000
C	-0.575717000	2.370642000	-1.767821000
C	3.778456000	-2.805617000	-1.537959000
C	-3.974509000	2.069615000	-0.175340000
H	-4.376304000	1.166228000	-0.635284000
H	-4.600197000	2.914357000	-0.494677000
H	-4.093773000	1.963555000	0.905950000
C	0.780095000	4.055007000	-0.278442000
H	0.994370000	4.069880000	0.794036000
H	0.591200000	5.091994000	-0.585991000
H	1.689319000	3.721424000	-0.780220000
C	0.895246000	-0.011072000	2.975216000
H	0.375115000	0.920667000	2.767391000
C	-1.908653000	3.889650000	1.416921000
H	-2.594536000	3.337514000	2.066615000
H	-2.375410000	4.859013000	1.196363000

H	-0.997838000	4.092026000	1.988801000
C	-2.520111000	0.986451000	-2.817560000
H	-1.786897000	0.353223000	-3.322969000
H	-2.982851000	1.628263000	-3.579358000
H	-3.296949000	0.327081000	-2.424807000
C	2.862920000	-3.513158000	-2.346815000
H	3.207550000	-4.385284000	-2.899001000
C	3.365137000	1.532396000	-1.050996000
H	3.114617000	0.949570000	-1.930659000
C	0.896438000	-0.520335000	4.273645000
H	0.409828000	0.034037000	5.071027000
C	2.123808000	-1.946004000	2.205000000
H	2.583604000	-2.515632000	1.405251000
C	0.405028000	2.161628000	-2.887258000
H	1.388343000	2.564863000	-2.639741000
H	0.064394000	2.659235000	-3.804072000
H	0.527484000	1.099484000	-3.129338000
C	3.098407000	1.995562000	1.304878000
H	2.643287000	1.784386000	2.266522000
C	4.318597000	2.546620000	-1.141834000
H	4.794252000	2.750444000	-2.096856000
C	1.504165000	-1.749253000	4.540171000
H	1.495268000	-2.154841000	5.547657000
C	2.115488000	-2.458633000	3.503528000
H	2.583522000	-3.418260000	3.703440000
C	4.054021000	3.008274000	1.211301000
H	4.323375000	3.573893000	2.098712000
C	4.662693000	3.292206000	-0.012162000
H	5.404472000	4.082145000	-0.083600000
H	3.964486000	-1.151632000	-0.205190000
H	-0.457268000	-0.647213000	-1.711597000
B	-1.497559000	-2.507052000	-2.062066000
H	-1.179870000	-3.659667000	-2.241781000
H	-2.335111000	-2.118948000	-2.835851000
H	-1.619089000	0.194730000	1.372847000
C	-3.324186000	-1.835169000	-0.213556000
C	-1.242584000	-2.699657000	0.448274000
C	-4.417875000	-1.855298000	-1.107459000
C	-3.507397000	-1.282398000	1.084132000
C	-1.363786000	-2.207628000	1.727883000
H	-0.401036000	-3.319892000	0.158111000
C	-5.655135000	-1.379121000	-0.703530000
H	-4.279176000	-2.273738000	-2.095539000
C	-4.773261000	-0.800975000	1.464812000
C	-2.347342000	-1.210312000	1.946651000
H	-0.626470000	-2.451859000	2.480339000

C	-5.845303000	-0.859527000	0.590676000
H	-6.491713000	-1.421863000	-1.395076000
H	-4.895067000	-0.383391000	2.461085000
H	-2.481830000	-0.804129000	2.944969000
H	-6.822246000	-0.497185000	0.895361000
N	-2.095984000	-2.386856000	-0.557555000
C	5.222901000	-3.232862000	-1.439375000
H	5.312512000	-4.277393000	-1.117226000
H	5.733245000	-3.153313000	-2.407302000
H	5.774693000	-2.615400000	-0.724053000

Int3$_{EC1}$

Co	-1.478438000	-0.893657000	-0.508329000
P	-0.326943000	0.723434000	0.482834000
N	-0.319138000	-0.573645000	-2.071983000
C	-2.585880000	-2.760391000	-0.422782000
C	-3.023534000	-1.932754000	-1.508976000
C	0.273645000	0.729067000	-2.154007000
C	-2.788883000	-2.034663000	0.777996000
C	1.174706000	2.671068000	-1.001061000
C	0.496158000	1.446483000	-0.970632000
C	-3.437746000	-0.780427000	0.436499000
C	0.905104000	0.384057000	1.788526000
C	-1.303757000	2.147771000	1.128621000
C	0.702255000	1.278125000	-3.369490000
H	0.539654000	0.726891000	-4.291025000
C	-3.602353000	-0.725216000	-0.969278000
C	1.644556000	3.204683000	-2.202448000
C	-2.021462000	-4.144328000	-0.547526000
H	-1.355864000	-4.227140000	-1.410075000
H	-2.824606000	-4.882834000	-0.667668000
H	-1.447942000	-4.421547000	0.340356000
C	-3.961653000	0.201851000	1.441799000
H	-3.269141000	0.343259000	2.275113000
H	-4.910776000	-0.157981000	1.858839000
H	-4.137700000	1.184893000	1.003196000
C	0.474499000	-0.349796000	2.907715000
H	-0.540722000	-0.731545000	2.943435000
C	-2.529059000	-2.543676000	2.167590000
H	-1.545109000	-3.016420000	2.248238000
H	-3.277757000	-3.289273000	2.464385000
H	-2.572077000	-1.735996000	2.902908000
C	-2.984353000	-2.321898000	-2.957609000
H	-3.052755000	-1.446749000	-3.611456000
H	-3.825185000	-2.979057000	-3.211611000
H	-2.060723000	-2.857434000	-3.196755000

C	1.376043000	2.495608000	-3.385326000
H	1.709580000	2.904605000	-4.335966000
C	-2.097328000	2.856915000	0.210712000
H	-2.069905000	2.588871000	-0.841355000
C	1.344885000	-0.593305000	3.967229000
H	1.003315000	-1.160936000	4.828068000
C	2.228806000	0.834665000	1.728013000
H	2.599731000	1.338868000	0.843229000
C	-4.245799000	0.374392000	-1.764237000
H	-4.266136000	1.310535000	-1.200439000
H	-5.280128000	0.129059000	-2.038086000
H	-3.701320000	0.566190000	-2.694995000
C	-1.342973000	2.505858000	2.481219000
H	-0.728074000	1.978080000	3.202627000
C	-2.917258000	3.897731000	0.640172000
H	-3.522857000	4.439577000	-0.080821000
C	2.659882000	-0.123209000	3.909947000
H	3.343945000	-0.322201000	4.729908000
C	3.099222000	0.580093000	2.789127000
H	4.130743000	0.908379000	2.715772000
C	-2.171148000	3.547159000	2.910356000
H	-2.192748000	3.815144000	3.962838000
C	-2.960843000	4.242332000	1.994964000
H	-3.602709000	5.051334000	2.331290000
H	1.316422000	3.226669000	-0.077655000
H	-0.858018000	-0.734665000	-2.921332000
B	0.839594000	-1.783320000	-2.158285000
H	1.476416000	-1.546321000	-3.159945000
H	0.170954000	-2.790975000	-2.281788000
H	0.919247000	-3.756782000	-0.396739000
C	2.941355000	-1.276735000	-0.753438000
C	1.190338000	-2.815851000	0.108071000
C	3.411928000	-0.299481000	-1.658777000
C	3.805605000	-1.613517000	0.331751000
C	2.123630000	-3.116055000	1.239710000
H	0.232203000	-2.444666000	0.527477000
C	4.652352000	0.313334000	-1.494998000
H	2.795490000	-0.011986000	-2.496527000
C	5.051416000	-0.992554000	0.467323000
C	3.340516000	-2.571159000	1.324054000
H	1.765548000	-3.817884000	1.989526000
C	5.492080000	-0.021410000	-0.430066000
H	4.960219000	1.065769000	-2.217495000
H	5.676382000	-1.279083000	1.311292000
H	4.003617000	-2.812726000	2.151829000
H	6.459905000	0.454690000	-0.306025000

N	1.689741000	-1.862595000	-0.895630000
C	2.426839000	4.495730000	-2.231785000
H	3.505574000	4.296932000	-2.246100000
H	2.193021000	5.088251000	-3.122068000
H	2.219308000	5.110734000	-1.351049000

Int3$_{EC3}$

Co	0.372656000	1.046100000	0.202305000
P	-1.308568000	-0.312425000	-0.277588000
N	0.236711000	0.381669000	2.097011000
C	1.903449000	2.184811000	-0.932541000
C	1.775203000	2.733721000	0.376399000
C	-0.969767000	-0.342426000	2.419135000
C	0.633717000	2.316929000	-1.571944000
C	-2.990671000	-1.506644000	1.717473000
C	-1.807124000	-0.818230000	1.400137000
C	-0.245548000	3.092824000	-0.702932000
C	-1.090390000	-1.807389000	-1.321650000
C	-2.841324000	0.449336000	-0.965412000
C	-1.330311000	-0.591628000	3.753052000
H	-0.678154000	-0.244888000	4.547883000
C	0.444517000	3.319895000	0.494307000
C	-3.350575000	-1.758446000	3.040240000
C	3.136134000	1.589631000	-1.550362000
H	3.878248000	1.317443000	-0.800066000
H	3.601080000	2.300520000	-2.245912000
H	2.907076000	0.680368000	-2.113010000
C	-1.591069000	3.626236000	-1.092481000
H	-2.104480000	2.968586000	-1.796161000
H	-1.487716000	4.606653000	-1.576842000
H	-2.247964000	3.754642000	-0.228730000
C	-0.363646000	-1.659778000	-2.511766000
H	0.084275000	-0.701855000	-2.751128000
C	0.333999000	1.993745000	-3.007681000
H	1.018619000	1.235269000	-3.396595000
H	0.448678000	2.885914000	-3.637687000
H	-0.691243000	1.635941000	-3.141170000
C	2.868762000	2.925692000	1.384299000
H	2.510802000	2.766929000	2.404991000
H	3.265885000	3.948081000	1.326733000
H	3.691297000	2.230092000	1.218928000
C	-2.496614000	-1.284643000	4.051812000
H	-2.758055000	-1.460044000	5.092712000
C	-3.672884000	1.201967000	-0.120648000
H	-3.450265000	1.264114000	0.939838000
C	-0.183717000	-2.744186000	-3.370665000

H	0.391043000	-2.618726000	-4.283576000
C	-1.631270000	-3.060140000	-1.001189000
H	-2.169798000	-3.202352000	-0.071752000
C	-0.055729000	4.052483000	1.703830000
H	-1.147314000	4.113804000	1.715817000
H	0.335363000	5.077446000	1.740395000
H	0.264351000	3.563876000	2.630787000
C	-3.146843000	0.380743000	-2.332645000
H	-2.528301000	-0.210392000	-2.999875000
C	-4.783620000	1.870508000	-0.633169000
H	-5.419546000	2.445424000	0.033951000
C	-0.719257000	-3.990230000	-3.042608000
H	-0.569666000	-4.838864000	-3.703594000
C	-1.441446000	-4.144877000	-1.856253000
H	-1.851636000	-5.115212000	-1.591601000
C	-4.254826000	1.059380000	-2.844437000
H	-4.478663000	0.996599000	-3.905487000
C	-5.075264000	1.806397000	-1.997680000
H	-5.938172000	2.331194000	-2.396922000
H	-3.659650000	-1.813123000	0.917800000
H	0.250027000	1.207113000	2.694081000
B	1.640340000	-0.394446000	2.538673000
H	1.344310000	-1.151515000	3.442608000
H	2.361669000	0.494244000	2.908052000
H	2.263370000	-1.711639000	-1.928847000
C	3.487812000	-0.978418000	0.779341000
C	1.509285000	-2.289112000	0.901981000
C	4.487006000	-0.238344000	1.443751000
C	3.825357000	-1.601402000	-0.448230000
C	1.762376000	-2.943959000	-0.241871000
H	0.734276000	-2.618610000	1.588696000
C	5.752540000	-0.071915000	0.885863000
H	4.263066000	0.198309000	2.407486000
C	5.109458000	-1.445827000	-0.972851000
C	2.769305000	-2.379421000	-1.203796000
H	1.194433000	-3.828884000	-0.503098000
C	6.077439000	-0.672083000	-0.332443000
H	6.494413000	0.514390000	1.422153000
H	5.344762000	-1.932840000	-1.917337000
H	3.235822000	-3.164701000	-1.812182000
H	7.065255000	-0.551525000	-0.767137000
N	2.207940000	-1.156581000	1.332433000
C	-4.614312000	-2.509306000	3.385827000
H	-4.385175000	-3.492055000	3.814957000
H	-5.211196000	-1.966234000	4.126805000
H	-5.239481000	-2.670566000	2.502961000

Int2$_F$

Co	0.439464000	-1.400394000	-0.392417000
P	-0.935706000	0.643927000	0.093294000
N	-1.165446000	-1.665874000	-1.750269000
C	1.789682000	-3.289026000	0.007608000
C	0.418959000	-3.673502000	-0.045388000
C	-2.363858000	-0.894974000	-1.682590000
C	1.983752000	-2.505827000	1.186797000
C	-3.581341000	1.053373000	-0.875390000
C	-2.420066000	0.263831000	-0.895661000
C	0.723944000	-2.360041000	1.838602000
C	-0.480249000	2.337166000	-0.475075000
C	-1.572245000	0.855060000	1.806170000
C	-3.507324000	-1.261713000	-2.412850000
H	-3.465652000	-2.147209000	-3.040315000
C	-0.251180000	-3.077197000	1.071552000
C	-4.716300000	0.693496000	-1.598765000
C	2.859724000	-3.708072000	-0.959421000
H	2.442512000	-3.887015000	-1.955274000
H	3.364513000	-4.630691000	-0.641684000
H	3.631774000	-2.937326000	-1.058437000
C	0.487376000	-1.716710000	3.176967000
H	1.179040000	-0.889678000	3.366379000
H	0.627432000	-2.437730000	3.993923000
H	-0.523801000	-1.314652000	3.267496000
C	0.276609000	2.423158000	-1.655520000
H	0.550770000	1.516075000	-2.183806000
C	3.318320000	-2.024673000	1.675859000
H	3.908817000	-1.573202000	0.870945000
H	3.911825000	-2.855732000	2.079869000
H	3.223181000	-1.279267000	2.469016000
C	-0.176819000	-4.627732000	-1.042830000
H	-1.266577000	-4.679823000	-0.948382000
H	0.199894000	-5.646704000	-0.887604000
H	0.064269000	-4.346485000	-2.074270000
C	-4.659191000	-0.487969000	-2.360365000
H	-5.532406000	-0.793326000	-2.932703000
C	-2.716839000	0.159636000	2.228963000
H	-3.305073000	-0.398049000	1.508389000
C	0.656050000	3.664587000	-2.162942000
H	1.242618000	3.712317000	-3.076024000
C	-0.868118000	3.520201000	0.170625000
H	-1.470235000	3.479323000	1.072026000
C	-1.702557000	-3.264035000	1.417238000
H	-2.020923000	-2.559453000	2.189080000

H	-1.906569000	-4.276003000	1.791397000
H	-2.351423000	-3.104436000	0.547662000
C	-0.830056000	1.568736000	2.763756000
H	0.053975000	2.118537000	2.460666000
C	-3.103212000	0.173822000	3.570164000
H	-3.990064000	-0.373225000	3.877290000
C	0.286764000	4.837750000	-1.500945000
H	0.588884000	5.804922000	-1.892728000
C	-0.481217000	4.761739000	-0.338061000
H	-0.785901000	5.669961000	0.174759000
C	-1.223503000	1.588904000	4.101400000
H	-0.639493000	2.151576000	4.824451000
C	-2.358124000	0.885913000	4.511824000
H	-2.659991000	0.895295000	5.554985000
H	-3.597749000	1.955116000	-0.267858000
H	-1.433508000	-2.646006000	-1.740934000
B	-0.335163000	-1.464113000	-3.118462000
H	-1.019911000	-1.770614000	-4.078096000
H	0.014120000	-0.297445000	-3.174832000
H	0.633307000	-2.217551000	-3.026093000
C	2.747126000	0.847676000	-0.137567000
C	2.703493000	-0.342821000	-2.128881000
C	2.286228000	1.033762000	1.188708000
C	3.774649000	1.708430000	-0.636374000
C	3.732157000	0.448036000	-2.688109000
H	2.242410000	-1.136215000	-2.705005000
C	2.804077000	2.038774000	1.974314000
H	1.535265000	0.353420000	1.566255000
C	4.278927000	2.743637000	0.194354000
C	4.253744000	1.482643000	-1.950410000
H	4.074885000	0.233509000	-3.694373000
C	3.801489000	2.910076000	1.473768000
H	2.447037000	2.160745000	2.992816000
H	5.052776000	3.397097000	-0.199384000
H	5.031080000	2.126989000	-2.353015000
H	4.192781000	3.701716000	2.105252000
N	2.206510000	-0.149995000	-0.914405000
C	-5.968289000	1.538176000	-1.579166000
H	-6.170671000	1.970684000	-2.566289000
H	-6.846991000	0.944463000	-1.302140000
H	-5.883294000	2.363737000	-0.866697000

$TS2_{FC1}$

Co	0.428234000	-1.315764000	0.041091000
P	-0.765672000	0.826757000	0.043360000
N	-0.679663000	-1.199424000	-2.202799000

C	0.814140000	-3.604486000	0.324689000
C	-0.586569000	-3.489879000	0.084522000
C	-1.961522000	-0.573069000	-2.022258000
C	1.113838000	-2.895502000	1.529418000
C	-3.382093000	1.145000000	-1.059839000
C	-2.150775000	0.471724000	-1.098383000
C	-0.102767000	-2.327064000	2.025773000
C	-0.055534000	2.407997000	-0.580379000
C	-1.483433000	1.310472000	1.663420000
C	-3.051838000	-0.983025000	-2.804310000
H	-2.914156000	-1.797778000	-3.510067000
C	-1.157166000	-2.700179000	1.126688000
C	-4.463261000	0.753175000	-1.850336000
C	1.823360000	-4.341392000	-0.507431000
H	1.470814000	-4.497615000	-1.532083000
H	2.054301000	-5.330540000	-0.090079000
H	2.763939000	-3.783125000	-0.560464000
C	-0.263600000	-1.604358000	3.335491000
H	0.608073000	-0.984831000	3.567611000
H	-0.385095000	-2.311569000	4.166653000
H	-1.132538000	-0.943770000	3.334275000
C	1.046593000	2.371303000	-1.443675000
H	1.490728000	1.425695000	-1.713699000
C	2.480443000	-2.802655000	2.146082000
H	3.217190000	-2.457818000	1.412521000
H	2.815376000	-3.776448000	2.525237000
H	2.499474000	-2.099749000	2.982270000
C	-1.372364000	-4.166377000	-1.002557000
H	-2.231882000	-3.563588000	-1.318983000
H	-1.776754000	-5.129639000	-0.663678000
H	-0.758620000	-4.382400000	-1.884835000
C	-4.282891000	-0.343981000	-2.706549000
H	-5.111057000	-0.683688000	-3.323708000
C	-2.764025000	0.942714000	2.099977000
H	-3.444602000	0.435789000	1.426817000
C	1.587619000	3.552698000	-1.950382000
H	2.452833000	3.502930000	-2.604260000
C	-0.618177000	3.649785000	-0.243023000
H	-1.467409000	3.698821000	0.431278000
C	-2.629497000	-2.445286000	1.293655000
H	-2.825766000	-1.787163000	2.141708000
H	-3.175013000	-3.381137000	1.473321000
H	-3.072654000	-1.981415000	0.403992000
C	-0.614626000	1.944274000	2.571876000
H	0.385649000	2.225463000	2.254709000
C	-3.169453000	1.208128000	3.410491000

H	-4.165168000	0.915562000	3.731509000
C	1.028627000	4.783392000	-1.606100000
H	1.453277000	5.704136000	-1.995901000
C	-0.077702000	4.829173000	-0.753675000
H	-0.518653000	5.784278000	-0.482295000
C	-1.023624000	2.208584000	3.876972000
H	-0.340179000	2.699016000	4.564163000
C	-2.302786000	1.839799000	4.302508000
H	-2.619258000	2.042079000	5.321493000
H	-3.505282000	1.982092000	-0.378541000
H	-0.804025000	-2.201411000	-2.287816000
B	0.176881000	-0.673094000	-3.301468000
H	0.539104000	-1.459246000	-4.133502000
H	0.064402000	0.488488000	-3.559545000
H	1.593068000	-0.570309000	-2.687894000
C	2.962220000	0.181772000	-0.013706000
C	2.490179000	-1.052447000	-2.028631000
C	2.614068000	0.564259000	1.306422000
C	4.074694000	0.845947000	-0.618041000
C	3.743590000	-0.506226000	-2.612457000
H	2.327122000	-2.109214000	-2.269548000
C	3.301209000	1.571445000	1.971267000
H	1.793507000	0.055546000	1.803931000
C	4.753348000	1.856582000	0.077642000
C	4.461327000	0.417987000	-1.945406000
H	4.027262000	-0.840351000	-3.606279000
C	4.374216000	2.236988000	1.360890000
H	2.999346000	1.840503000	2.980824000
H	5.592313000	2.347672000	-0.411547000
H	5.361626000	0.843277000	-2.384540000
H	4.905051000	3.026175000	1.883556000
N	2.252708000	-0.797415000	-0.659879000
C	-5.781230000	1.488607000	-1.800671000
H	-5.926330000	2.098606000	-2.700400000
H	-6.624513000	0.792135000	-1.743225000
H	-5.835683000	2.156877000	-0.936597000

Int3$_F$

Co	0.778872000	-0.835825000	0.076325000
P	-1.171908000	0.597769000	0.013609000
N	-1.774298000	-1.102830000	-2.421948000
C	1.426073000	-2.984327000	-0.199162000
C	0.061076000	-2.995037000	0.239456000
C	-2.870689000	-1.000973000	-1.530046000
C	2.226068000	-2.452220000	0.859844000

C	-3.849439000	-0.211484000	0.545407000
C	-2.760030000	-0.254314000	-0.335039000
C	1.358250000	-2.102477000	1.938984000
C	-1.088793000	1.924791000	-1.257695000
C	-1.435483000	1.538368000	1.566332000
C	-4.078625000	-1.641714000	-1.823608000
H	-4.155232000	-2.216059000	-2.740702000
C	0.021638000	-2.452998000	1.564736000
C	-5.054386000	-0.863363000	0.263767000
C	1.947889000	-3.517150000	-1.503851000
H	1.181693000	-3.490745000	-2.285340000
H	2.281596000	-4.559145000	-1.411921000
H	2.801734000	-2.930236000	-1.856679000
C	1.820893000	-1.565081000	3.266508000
H	2.496935000	-0.712234000	3.142308000
H	2.364049000	-2.330024000	3.835670000
H	0.981769000	-1.242633000	3.890181000
C	0.119844000	2.628856000	-1.380621000
H	0.969990000	2.352111000	-0.766985000
C	3.715653000	-2.275087000	0.838077000
H	4.076192000	-2.075980000	-0.174382000
H	4.230127000	-3.171600000	1.209270000
H	4.026862000	-1.430928000	1.458944000
C	-1.084922000	-3.680008000	-0.452197000
H	-2.046612000	-3.230484000	-0.192686000
H	-1.134130000	-4.739466000	-0.166225000
H	-0.991454000	-3.645947000	-1.541228000
C	-5.154859000	-1.565062000	-0.944273000
H	-6.081786000	-2.075603000	-1.192783000
C	-0.707269000	1.165867000	2.703750000
H	-0.017885000	0.331604000	2.641202000
C	0.237595000	3.659222000	-2.313318000
H	1.178493000	4.194123000	-2.402965000
C	-2.172395000	2.265753000	-2.079427000
H	-3.110551000	1.726320000	-1.996829000
C	-1.207089000	-2.387941000	2.428478000
H	-1.105814000	-1.658072000	3.236103000
H	-1.416529000	-3.359581000	2.895684000
H	-2.094172000	-2.110996000	1.850061000
C	-2.305602000	2.638404000	1.634199000
H	-2.853631000	2.949638000	0.749681000
C	-0.856701000	1.869755000	3.900118000
H	-0.283535000	1.574692000	4.774180000
C	-0.842707000	3.991692000	-3.134052000
H	-0.746012000	4.788551000	-3.865876000
C	-2.046072000	3.293545000	-3.015501000

H	-2.888890000	3.545994000	-3.652596000
C	-2.456433000	3.337420000	2.830760000
H	-3.131289000	4.187201000	2.877767000
C	-1.733971000	2.952942000	3.965103000
H	-1.849723000	3.504243000	4.893784000
H	-3.762261000	0.352790000	1.468455000
H	-0.882375000	-1.225277000	-1.949001000
B	-1.779242000	-1.021321000	-3.819459000
H	-0.738082000	-1.142998000	-4.390613000
H	-2.809763000	-0.835188000	-4.392147000
H	1.856262000	0.880027000	-2.836101000
C	3.160096000	0.792454000	-0.184707000
C	2.265357000	-0.001156000	-2.300646000
C	2.951276000	1.317866000	1.112122000
C	4.482523000	0.849337000	-0.724359000
C	3.684857000	-0.247899000	-2.747165000
H	1.622419000	-0.840012000	-2.603922000
C	3.998833000	1.866768000	1.846510000
H	1.945591000	1.292194000	1.524516000
C	5.515206000	1.414941000	0.031004000
C	4.712641000	0.198455000	-2.007179000
H	3.846515000	-0.746928000	-3.699645000
C	5.290555000	1.926079000	1.310123000
H	3.802297000	2.266550000	2.838262000
H	6.517717000	1.434579000	-0.392259000
H	5.741057000	0.061155000	-2.335348000
H	6.104967000	2.364921000	1.878052000
N	2.131553000	0.178364000	-0.854727000
C	-6.202534000	-0.830149000	1.244075000
H	-6.128174000	0.027095000	1.919523000
H	-7.166768000	-0.774196000	0.728982000
H	-6.217041000	-1.735287000	1.863657000

TS2$_{GC1}$

Co	-1.151191000	-1.149985000	0.439098000
P	-0.219566000	0.949919000	0.018493000
N	0.978336000	-0.500969000	-2.407145000
C	-2.398003000	-2.974587000	0.336838000
C	-2.145242000	-2.487272000	-0.985340000
C	1.918641000	0.289741000	-1.698304000
C	-3.080291000	-1.945650000	1.033448000
C	2.538014000	1.699235000	0.167899000
C	1.545262000	1.004248000	-0.537154000
C	-3.380560000	-0.873674000	0.102096000
C	-0.260659000	2.122695000	1.462027000
C	-1.061306000	2.057133000	-1.214864000

C	3.239510000	0.369234000	-2.154932000
H	3.519573000	-0.190997000	-3.039411000
C	-2.815830000	-1.220323000	-1.145559000
C	3.861340000	1.764472000	-0.272773000
C	-1.951354000	-4.303954000	0.868952000
H	-0.862161000	-4.399463000	0.799208000
H	-2.403101000	-5.128946000	0.304810000
H	-2.237984000	-4.433224000	1.916714000
C	-4.302996000	0.266842000	0.423149000
H	-3.963097000	0.845312000	1.290505000
H	-5.308642000	-0.103445000	0.663412000
H	-4.400317000	0.954365000	-0.418268000
C	-0.744413000	1.715108000	2.710070000
H	-1.084117000	0.692758000	2.836174000
C	-3.561312000	-1.990007000	2.455819000
H	-2.903874000	-2.595327000	3.087515000
H	-4.567768000	-2.423338000	2.524755000
H	-3.607331000	-0.985958000	2.887786000
C	-1.497618000	-3.293441000	-2.072457000
H	-1.072945000	-2.663684000	-2.856889000
H	-2.225186000	-3.961768000	-2.553732000
H	-0.699258000	-3.920930000	-1.667039000
C	4.189540000	1.111190000	-1.465521000
H	5.210957000	1.144940000	-1.836003000
C	-0.559596000	2.345313000	-2.491022000
H	0.405899000	1.968210000	-2.799983000
C	-0.818854000	2.613343000	3.778507000
H	-1.200691000	2.276625000	4.738303000
C	0.124757000	3.466547000	1.299307000
H	0.473700000	3.810800000	0.330687000
C	-2.930745000	-0.480593000	-2.446997000
H	-3.312578000	0.532135000	-2.310145000
H	-3.605717000	-1.007005000	-3.134598000
H	-1.964116000	-0.392963000	-2.950998000
C	-2.287573000	2.626484000	-0.834992000
H	-2.670441000	2.456786000	0.164378000
C	-1.288770000	3.142206000	-3.377171000
H	-0.882999000	3.349322000	-4.363358000
C	-0.412490000	3.935969000	3.610338000
H	-0.470199000	4.635556000	4.439416000
C	0.057465000	4.361945000	2.363994000
H	0.363486000	5.394261000	2.219862000
C	-3.013690000	3.423542000	-1.717659000
H	-3.960751000	3.851187000	-1.400424000
C	-2.521871000	3.674138000	-3.001007000
H	-3.087125000	4.290865000	-3.693715000

H	2.278676000	2.204643000	1.090678000
H	0.309943000	-0.970579000	-1.795675000
B	0.876638000	-0.662413000	-3.791822000
H	1.611267000	-0.055238000	-4.511614000
H	0.040724000	-1.393254000	-4.226903000
H	-0.823653000	-0.821446000	1.891097000
C	1.865540000	-2.271374000	0.458631000
C	0.214452000	-1.898896000	2.118582000
C	2.246908000	-2.891764000	-0.753150000
C	2.870812000	-1.627540000	1.238910000
C	1.196383000	-1.188594000	2.938925000
H	-0.521186000	-2.498056000	2.661036000
C	3.567236000	-2.881116000	-1.167216000
H	1.479185000	-3.384514000	-1.339047000
C	4.205685000	-1.623901000	0.791375000
C	2.466208000	-1.042449000	2.488043000
H	0.882712000	-0.785321000	3.896820000
C	4.558407000	-2.246573000	-0.394349000
H	3.841230000	-3.370340000	-2.097987000
H	4.959138000	-1.125564000	1.397245000
H	3.209691000	-0.519664000	3.085497000
H	5.590915000	-2.240792000	-0.729887000
N	0.561008000	-2.394400000	0.882927000
C	4.914803000	2.479332000	0.538620000
H	5.487094000	1.768051000	1.148025000
H	5.630506000	3.003389000	-0.103211000
H	4.470826000	3.211037000	1.220159000

$TS1_H$

Co	-1.352108000	-0.152664000	0.441272000
P	0.720907000	0.055970000	-0.308772000
N	-0.545014000	0.077921000	2.191766000
C	-3.394148000	0.075744000	-0.258081000
C	-3.334148000	-0.627963000	0.980579000
C	0.802924000	-0.116150000	2.372584000
C	-2.631342000	-0.668007000	-1.204072000
C	3.036274000	-0.312941000	1.359488000
C	1.652641000	-0.131479000	1.232061000
C	-2.231848000	-1.925838000	-0.583364000
C	1.123430000	1.699477000	-1.041570000
C	1.430506000	-1.140653000	-1.506419000
C	1.424415000	-0.275936000	3.634983000
H	0.808478000	-0.263541000	4.531281000
C	-2.655971000	-1.895576000	0.753205000
C	3.638608000	-0.474777000	2.606926000
C	-4.044033000	1.411690000	-0.469801000

H	-3.888745000	2.061930000	0.396365000
H	-5.125599000	1.313533000	-0.628141000
H	-3.628342000	1.924366000	-1.342306000
C	-1.530264000	-3.043517000	-1.297311000
H	-0.837213000	-2.667363000	-2.053267000
H	-2.250223000	-3.696711000	-1.808569000
H	-0.949747000	-3.664281000	-0.609990000
C	0.121808000	2.346866000	-1.779613000
H	-0.847366000	1.869863000	-1.884524000
C	-2.449800000	-0.356838000	-2.663160000
H	-2.610900000	0.704026000	-2.874479000
H	-3.161624000	-0.922070000	-3.279153000
H	-1.444747000	-0.617989000	-3.007866000
C	-3.966221000	-0.199137000	2.272412000
H	-3.477256000	-0.675623000	3.127623000
H	-5.029034000	-0.470727000	2.314503000
H	-3.887004000	0.882834000	2.409047000
C	2.796647000	-0.448724000	3.736582000
H	3.237882000	-0.576136000	4.723541000
C	1.877673000	-2.392263000	-1.054139000
H	1.899628000	-2.600055000	0.011072000
C	0.352654000	3.605358000	-2.338919000
H	-0.429297000	4.094110000	-2.914252000
C	2.355473000	2.339374000	-0.856201000
H	3.127190000	1.864891000	-0.259366000
C	-2.416232000	-2.931470000	1.811054000
H	-1.749535000	-3.720046000	1.453647000
H	-3.352395000	-3.404066000	2.133435000
H	-1.950150000	-2.483939000	2.695608000
C	1.410449000	-0.882948000	-2.885890000
H	1.078492000	0.084223000	-3.251411000
C	2.294185000	-3.363956000	-1.963611000
H	2.641206000	-4.326960000	-1.599748000
C	1.585007000	4.236980000	-2.149156000
H	1.765311000	5.219881000	-2.575427000
C	2.582856000	3.602398000	-1.405032000
H	3.538538000	4.094141000	-1.246750000
C	1.821930000	-1.860167000	-3.793816000
H	1.801781000	-1.647217000	-4.858940000
C	2.263142000	-3.103283000	-3.335672000
H	2.584565000	-3.862456000	-4.042856000
H	3.650132000	-0.363253000	0.462135000
H	-1.085142000	-0.085578000	3.034551000
B	-1.018566000	2.206588000	1.712910000
H	-1.791710000	2.325115000	2.619206000
H	0.134806000	2.496840000	1.804327000

H	-1.446328000	1.855329000	0.635979000
N	-1.356647000	4.328357000	0.919098000
H	-1.015185000	5.019500000	1.580042000
H	-0.758032000	4.350371000	0.097680000
H	-2.295399000	4.593727000	0.636125000
C	5.127647000	-0.681601000	2.753383000
H	5.591220000	0.117712000	3.345154000
H	5.358894000	-1.627275000	3.259197000
H	5.625047000	-0.699606000	1.778610000

Int1$_H$

Co	-1.509648000	-0.422320000	0.482211000
P	0.843480000	0.332557000	0.260450000
N	-0.648015000	-2.210300000	-0.215602000
C	-3.820208000	-0.225374000	0.484770000
C	-3.413768000	-0.807135000	-0.754728000
C	0.676700000	-2.230914000	-0.754179000
C	-3.333828000	1.118338000	0.519967000
C	2.854340000	-1.184677000	-1.064642000
C	1.531591000	-1.129645000	-0.593135000
C	-2.623873000	1.364545000	-0.692346000
C	1.981287000	0.489611000	1.710154000
C	1.224173000	1.763367000	-0.824875000
C	1.164463000	-3.359754000	-1.434150000
H	0.515146000	-4.223099000	-1.543908000
C	-2.658075000	0.169917000	-1.476971000
C	3.340046000	-2.302440000	-1.739593000
C	-4.653642000	-0.925305000	1.522006000
H	-4.290718000	-1.943298000	1.701997000
H	-5.704411000	-1.003169000	1.213352000
H	-4.646882000	-0.394784000	2.480183000
C	-2.089941000	2.698134000	-1.125537000
H	-1.695060000	3.272427000	-0.282619000
H	-2.882016000	3.305949000	-1.585007000
H	-1.285212000	2.604942000	-1.857753000
C	1.899990000	-0.535122000	2.671883000
H	1.227520000	-1.374407000	2.509540000
C	-3.555453000	2.129054000	1.609776000
H	-3.860977000	1.657533000	2.549873000
H	-4.344812000	2.846577000	1.348812000
H	-2.649601000	2.712537000	1.812233000
C	-3.785702000	-2.188314000	-1.216896000
H	-3.238304000	-2.472480000	-2.121481000
H	-4.853654000	-2.253307000	-1.462367000
H	-3.583772000	-2.941715000	-0.446857000
C	2.463239000	-3.386679000	-1.924481000

H	2.816606000	-4.272143000	-2.448226000
C	1.469676000	1.600635000	-2.197276000
H	1.551069000	0.604111000	-2.617530000
C	2.709513000	-0.509720000	3.806528000
H	2.637628000	-1.313961000	4.533355000
C	2.909002000	1.523075000	1.903128000
H	3.020331000	2.306466000	1.162102000
C	-2.052141000	-0.008733000	-2.841076000
H	-1.208762000	0.671516000	-2.992017000
H	-2.776089000	0.182408000	-3.644453000
H	-1.673641000	-1.026949000	-2.983256000
C	1.106283000	3.067658000	-0.313016000
H	0.876932000	3.218839000	0.738067000
C	1.606847000	2.713387000	-3.029856000
H	1.792324000	2.568394000	-4.090306000
C	3.613785000	0.537410000	4.002488000
H	4.241776000	0.559699000	4.888402000
C	3.714736000	1.546480000	3.044590000
H	4.428947000	2.354459000	3.177524000
C	1.263502000	4.176843000	-1.142073000
H	1.175532000	5.176267000	-0.725928000
C	1.511094000	4.003341000	-2.505746000
H	1.622122000	4.866945000	-3.154734000
H	3.506042000	-0.327730000	-0.911721000
H	-1.270605000	-2.595942000	-0.921924000
B	-0.848686000	-3.161059000	1.081203000
H	-2.003036000	-2.980522000	1.462396000
H	-0.666877000	-4.325293000	0.779120000
H	-0.049674000	-2.807242000	1.937267000
N	-1.413023000	-0.413186000	2.645852000
H	-1.288398000	-1.404239000	2.859051000
H	-0.645333000	0.100358000	3.070441000
H	-2.285172000	-0.097706000	3.060643000
C	4.758398000	-2.361504000	-2.255178000
H	5.323499000	-3.166163000	-1.770331000
H	4.783416000	-2.556183000	-3.333641000
H	5.290913000	-1.423990000	-2.071711000

Int2$_H$

Co	-1.552005000	-0.437983000	0.179299000
P	0.540924000	0.278199000	0.180260000
N	0.303572000	-2.663397000	-0.966068000
C	-3.556300000	-0.971376000	0.040365000
C	-3.016297000	-1.030852000	-1.281192000
C	1.531144000	-2.030483000	-1.124057000
C	-3.503114000	0.410957000	0.488189000

C	3.018328000	-0.085393000	-1.110339000
C	1.764661000	-0.662760000	-0.815773000
C	-2.881841000	1.170720000	-0.525225000
C	1.268662000	0.253111000	1.887693000
C	0.772726000	2.026513000	-0.360183000
C	2.623094000	-2.777494000	-1.626088000
H	2.457021000	-3.826642000	-1.845402000
C	-2.537730000	0.271018000	-1.611330000
C	4.073950000	-0.820578000	-1.640471000
C	-4.209650000	-2.106797000	0.774158000
H	-3.740927000	-3.061304000	0.519605000
H	-5.276441000	-2.181310000	0.524398000
H	-4.129112000	-1.975706000	1.856232000
C	-2.658461000	2.654964000	-0.519183000
H	-2.529089000	3.038693000	0.496617000
H	-3.512776000	3.182061000	-0.964309000
H	-1.768280000	2.935140000	-1.085556000
C	0.421887000	0.594696000	2.956126000
H	-0.620205000	0.820033000	2.748735000
C	-4.038348000	0.919654000	1.794806000
H	-3.891055000	0.187342000	2.593386000
H	-5.114322000	1.131254000	1.734373000
H	-3.538528000	1.844455000	2.097239000
C	-2.950028000	-2.252290000	-2.154464000
H	-2.132039000	-2.187048000	-2.876875000
H	-3.881798000	-2.383069000	-2.720209000
H	-2.787044000	-3.159228000	-1.565999000
C	3.853076000	-2.191719000	-1.869956000
H	4.660265000	-2.800782000	-2.271874000
C	0.914139000	2.318761000	-1.726552000
H	1.001963000	1.508607000	-2.442937000
C	0.899096000	0.606258000	4.266379000
H	0.233671000	0.879900000	5.080459000
C	2.595146000	-0.108138000	2.163765000
H	3.262136000	-0.388069000	1.355628000
C	-1.962944000	0.677129000	-2.938300000
H	-1.393332000	1.604759000	-2.859999000
H	-2.758400000	0.841887000	-3.677128000
H	-1.296716000	-0.091411000	-3.342334000
C	0.683837000	3.087456000	0.551909000
H	0.583301000	2.885011000	1.613065000
C	0.954432000	3.640995000	-2.169278000
H	1.065534000	3.848820000	-3.229724000
C	2.222946000	0.245767000	4.532237000
H	2.592326000	0.240147000	5.553865000
C	3.064923000	-0.117622000	3.480354000

H	4.092789000	-0.407503000	3.680375000
C	0.724571000	4.409743000	0.106773000
H	0.654898000	5.220073000	0.826905000
C	0.854968000	4.691580000	-1.254004000
H	0.884704000	5.721033000	-1.598792000
H	3.176184000	0.967168000	-0.890642000
H	-0.478652000	-2.015030000	-1.008325000
B	0.083385000	-3.864197000	-0.073105000
H	0.956991000	-4.697822000	-0.144390000
H	-1.040741000	-4.304529000	-0.168650000
H	-1.386276000	-1.098798000	1.529889000
N	0.160974000	-3.291252000	1.508130000
C	5.404289000	-0.181836000	-1.961049000
H	6.236221000	-0.744641000	-1.521974000
H	5.580376000	-0.143572000	-3.043345000
H	5.457419000	0.843568000	-1.582968000
H	-0.516351000	-2.512564000	1.607434000
H	-0.050138000	-4.012039000	2.196357000
H	1.080954000	-2.904708000	1.721124000

$TS2_{HC1}$

Co	-0.373174000	0.899305000	-0.459462000
P	1.641859000	-0.065191000	0.364759000
N	0.250956000	-0.069084000	-2.328480000
C	-2.103021000	2.398800000	-0.507101000
C	-1.087276000	2.857246000	-1.405780000
C	1.342520000	-1.019047000	-2.237037000
C	-1.569179000	2.456204000	0.822180000
C	3.147120000	-2.066022000	-0.979257000
C	2.066385000	-1.170543000	-1.037696000
C	-0.233860000	2.948264000	0.741406000
C	1.387084000	-1.123721000	1.844892000
C	3.282522000	0.735460000	0.666924000
C	1.713576000	-1.812731000	-3.336985000
H	1.174780000	-1.694958000	-4.271127000
C	0.070681000	3.184154000	-0.634261000
C	3.508119000	-2.861823000	-2.066779000
C	-3.509100000	2.035374000	-0.891600000
H	-3.535580000	1.464818000	-1.824265000
H	-4.128360000	2.931366000	-1.035122000
H	-3.993479000	1.420498000	-0.130765000
C	0.681234000	3.227405000	1.900298000
H	0.301076000	2.776365000	2.822112000
H	0.773275000	4.305079000	2.090837000
H	1.695272000	2.847351000	1.733143000
C	1.035431000	-0.462025000	3.035057000

H	0.958725000	0.620678000	3.044078000
C	-2.292289000	2.094722000	2.089087000
H	-3.187193000	1.506324000	1.879224000
H	-2.601891000	2.988135000	2.648145000
H	-1.664971000	1.490424000	2.753308000
C	-1.246969000	3.109195000	-2.878185000
H	-0.329376000	2.886301000	-3.436549000
H	-1.486262000	4.162847000	-3.077981000
H	-2.043853000	2.499603000	-3.307834000
C	2.767167000	-2.717640000	-3.249158000
H	3.033246000	-3.309959000	-4.121817000
C	3.843410000	1.454627000	-0.403588000
H	3.337611000	1.469174000	-1.364998000
C	0.778498000	-1.181243000	4.201020000
H	0.517554000	-0.652446000	5.113185000
C	1.433256000	-2.524148000	1.841306000
H	1.677720000	-3.058340000	0.929658000
C	1.335622000	3.794003000	-1.168444000
H	2.175259000	3.644718000	-0.484832000
H	1.228454000	4.876339000	-1.325895000
H	1.619777000	3.363748000	-2.136635000
C	3.952626000	0.732235000	1.898521000
H	3.547353000	0.179555000	2.739090000
C	5.040306000	2.149043000	-0.247791000
H	5.457740000	2.697304000	-1.087597000
C	0.833502000	-2.578083000	4.188712000
H	0.619697000	-3.141156000	5.092364000
C	1.151149000	-3.245046000	3.004916000
H	1.182044000	-4.330665000	2.983355000
C	5.150940000	1.434156000	2.054323000
H	5.658065000	1.419075000	3.015117000
C	5.698075000	2.144444000	0.985907000
H	6.628899000	2.689889000	1.110679000
H	3.725801000	-2.130310000	-0.061171000
H	0.614026000	0.773125000	-2.777492000
B	-0.979811000	-0.491247000	-3.229964000
H	-0.683686000	-0.569815000	-4.409046000
H	-1.889174000	0.276380000	-3.044573000
H	-1.003078000	-0.428386000	0.378969000
N	-1.481746000	-1.938186000	-2.761033000
H	-0.716328000	-2.610386000	-2.697607000
H	-2.029127000	-1.933482000	-1.811293000
H	-2.125244000	-2.298269000	-3.463435000
C	-6.189537000	-0.756398000	-0.990828000
C	-4.922142000	-1.266368000	-1.211106000
C	-4.004545000	-1.417845000	-0.142454000

C	-4.434026000	-1.064348000	1.177296000
C	-5.732880000	-0.554153000	1.371814000
C	-6.603144000	-0.390173000	0.305064000
H	-6.870377000	-0.629959000	-1.828285000
H	-4.599164000	-1.537538000	-2.212164000
C	-3.504697000	-1.280103000	2.252079000
H	-6.042191000	-0.285076000	2.379497000
H	-7.597953000	0.013840000	0.465774000
C	-2.233087000	-1.670828000	1.981090000
C	-1.829055000	-1.830795000	0.599909000
H	-3.836491000	-1.116526000	3.274870000
H	-1.495756000	-1.816119000	2.761464000
H	-0.907091000	-2.375998000	0.412064000
N	-2.753081000	-1.904739000	-0.387346000
C	4.648392000	-3.847624000	-1.977849000
H	4.276588000	-4.875769000	-1.888605000
H	5.278639000	-3.809282000	-2.872464000
H	5.282138000	-3.649343000	-1.108893000

TS2$_{IC1}$

Co	0.031678000	-1.364094000	0.082300000
P	-1.745356000	0.304885000	0.218874000
N	0.230365000	1.224123000	-2.113703000
C	1.360841000	-2.679179000	-1.184221000
C	0.159117000	-2.423298000	-1.928756000
C	-0.793614000	2.086039000	-1.789949000
C	0.999469000	-3.437755000	-0.027615000
C	-2.849095000	2.706398000	-0.590980000
C	-1.819959000	1.774347000	-0.853601000
C	-0.421098000	-3.626835000	-0.039109000
C	-1.666314000	1.080667000	1.896573000
C	-3.376432000	-0.530700000	0.149043000
C	-0.867354000	3.362886000	-2.408566000
H	-0.098087000	3.626379000	-3.123513000
C	-0.934542000	-3.028096000	-1.229908000
C	-2.920329000	3.940560000	-1.219198000
C	2.751696000	-2.297046000	-1.607223000
H	2.756755000	-1.351144000	-2.152663000
H	3.185625000	-3.063647000	-2.262328000
H	3.423816000	-2.172738000	-0.755513000
C	-1.202710000	-4.415185000	0.974800000
H	-0.835496000	-4.243381000	1.992775000
H	-1.128172000	-5.494193000	0.786824000
H	-2.263708000	-4.153576000	0.954572000
C	-0.533316000	1.873207000	2.156941000
H	0.219521000	2.011335000	1.386084000

C	1.939396000	-3.966375000	1.017741000
H	2.887790000	-3.421379000	1.018722000
H	2.179090000	-5.022786000	0.841607000
H	1.508952000	-3.904848000	2.024333000
C	0.075856000	-1.854921000	-3.318786000
H	-0.844150000	-1.282832000	-3.473433000
H	0.081703000	-2.664488000	-4.060730000
H	0.914278000	-1.193872000	-3.542813000
C	-1.892264000	4.246393000	-2.134750000
H	-1.905610000	5.207516000	-2.644720000
C	-4.343800000	-0.227836000	-0.817996000
H	-4.169536000	0.577662000	-1.523147000
C	-0.368396000	2.491726000	3.394124000
H	0.514150000	3.097401000	3.577212000
C	-2.637788000	0.937649000	2.894595000
H	-3.532344000	0.353200000	2.710609000
C	-2.333079000	-3.122122000	-1.764972000
H	-3.057756000	-3.378327000	-0.990873000
H	-2.391413000	-3.893131000	-2.544768000
H	-2.659598000	-2.181988000	-2.217969000
C	-3.613857000	-1.589361000	1.043256000
H	-2.857367000	-1.859939000	1.774850000
C	-5.529494000	-0.964209000	-0.880758000
H	-6.268849000	-0.723834000	-1.639073000
C	-1.337089000	2.333505000	4.390159000
H	-1.210024000	2.814354000	5.355497000
C	-2.470480000	1.562361000	4.134410000
H	-3.234296000	1.446555000	4.898137000
C	-4.805957000	-2.309103000	0.991100000
H	-4.978109000	-3.119024000	1.694049000
C	-5.766577000	-1.999651000	0.023737000
H	-6.691155000	-2.566960000	-0.026312000
H	-3.603513000	2.452268000	0.150238000
H	0.079114000	0.273752000	-1.804820000
B	1.584304000	1.525709000	-2.643871000
H	2.167921000	0.570027000	-3.085580000
H	1.740980000	2.580625000	-3.197953000
H	2.398174000	1.743109000	-1.503580000
N	1.274178000	-0.699312000	1.580502000
H	0.802215000	-0.205446000	2.336499000
H	1.803164000	-1.456839000	2.007276000
C	-4.030533000	4.922654000	-0.935216000
H	-3.635619000	5.885942000	-0.591070000
H	-4.628372000	5.124679000	-1.832587000
H	-4.708831000	4.547172000	-0.163114000
C	7.136666000	-0.284424000	0.632875000

C	6.820265000	0.957262000	0.097522000
C	5.490455000	1.404108000	0.020706000
C	4.421624000	0.570892000	0.490484000
C	4.779999000	-0.675766000	1.070520000
C	6.100313000	-1.093518000	1.130121000
H	5.934009000	3.422231000	-0.686266000
H	8.167903000	-0.620508000	0.678779000
H	7.609022000	1.611192000	-0.269768000
C	5.136618000	2.721497000	-0.446976000
H	3.995204000	-1.311283000	1.469022000
H	6.334184000	-2.061282000	1.567488000
C	2.786888000	2.086349000	-0.284149000
C	3.839496000	3.082704000	-0.551564000
H	2.003646000	-0.010083000	1.140552000
H	1.815397000	2.509571000	-0.004242000
H	3.544229000	4.065766000	-0.906225000
N	3.114164000	0.956699000	0.445432000

Int2$_I$

Co	0.108468000	-1.407848000	0.254579000
P	-1.492071000	0.157807000	0.251662000
N	0.724425000	1.239470000	-1.873889000
C	1.526156000	-2.473090000	-1.147214000
C	0.321602000	-2.283744000	-1.861917000
C	-0.439412000	2.011477000	-1.726799000
C	1.206277000	-3.224897000	0.037272000
C	-2.586678000	2.527249000	-0.702880000
C	-1.484784000	1.656369000	-0.840191000
C	-0.185730000	-3.607202000	-0.020068000
C	-1.767056000	1.002337000	1.884745000
C	-3.122464000	-0.644349000	-0.064488000
C	-0.580215000	3.206790000	-2.465246000
H	0.205848000	3.488159000	-3.154241000
C	-0.733186000	-3.012278000	-1.173803000
C	-2.721475000	3.705135000	-1.427344000
C	2.910127000	-2.035988000	-1.531613000
H	2.922075000	-1.568847000	-2.517178000
H	3.586379000	-2.898886000	-1.563366000
H	3.333928000	-1.321436000	-0.822950000
C	-0.892602000	-4.480196000	0.975428000
H	-0.570025000	-4.254575000	1.997120000
H	-0.692059000	-5.544842000	0.796889000
H	-1.975646000	-4.336211000	0.931026000
C	-0.714061000	1.779927000	2.395660000
H	0.218210000	1.844773000	1.851420000
C	2.214223000	-3.724985000	1.029238000

H	3.053961000	-3.030983000	1.121351000
H	2.623294000	-4.695211000	0.718627000
H	1.771238000	-3.864360000	2.020512000
C	0.139404000	-1.650830000	-3.212247000
H	-0.707226000	-0.956994000	-3.226109000
H	-0.056751000	-2.417696000	-3.973087000
H	1.022863000	-1.089998000	-3.519492000
C	-1.687619000	4.023863000	-2.323130000
H	-1.750067000	4.935900000	-2.912400000
C	-3.969393000	-0.323395000	-1.132170000
H	-3.699591000	0.468404000	-1.822059000
C	-0.851157000	2.468297000	3.599471000
H	-0.021473000	3.060266000	3.974871000
C	-2.967099000	0.943718000	2.607305000
H	-3.805994000	0.368803000	2.233077000
C	-2.087454000	-3.250789000	-1.768972000
H	-2.807322000	-3.610729000	-1.033412000
H	-2.014969000	-4.006495000	-2.562684000
H	-2.502209000	-2.347369000	-2.221084000
C	-3.488855000	-1.689506000	0.802499000
H	-2.818271000	-1.974072000	1.608425000
C	-5.165283000	-1.023174000	-1.319840000
H	-5.808044000	-0.768456000	-2.157603000
C	-2.047898000	2.396053000	4.315917000
H	-2.156942000	2.931457000	5.254580000
C	-3.103311000	1.634903000	3.814119000
H	-4.042132000	1.578801000	4.358017000
C	-4.692635000	-2.368552000	0.628337000
H	-4.966054000	-3.165010000	1.314569000
C	-5.534693000	-2.038087000	-0.438035000
H	-6.467914000	-2.574448000	-0.582582000
H	-3.365527000	2.266648000	0.008201000
H	0.726699000	0.396592000	-1.302966000
B	1.895850000	1.491192000	-2.617864000
H	2.753135000	0.671047000	-2.592686000
H	1.994342000	2.456845000	-3.309318000
H	1.371303000	2.575233000	0.271089000
N	0.881905000	-0.881871000	1.867393000
H	0.288634000	-0.473000000	2.584589000
H	1.430973000	-1.609623000	2.319949000
C	-3.917508000	4.610487000	-1.260247000
H	-3.615697000	5.613041000	-0.935383000
H	-4.462886000	4.729508000	-2.204000000
H	-4.617080000	4.216336000	-0.517696000
C	6.736827000	-0.195978000	0.438403000
C	6.315137000	0.970370000	-0.200415000

C	5.004429000	1.440838000	-0.066774000
C	4.078215000	0.712802000	0.731852000
C	4.512035000	-0.460808000	1.378139000
C	5.823673000	-0.905741000	1.225848000
H	5.141600000	3.076717000	-1.516637000
H	7.758112000	-0.545997000	0.326416000
H	7.008426000	1.531830000	-0.823208000
C	4.505445000	2.620022000	-0.761542000
H	3.808010000	-1.005085000	1.999093000
H	6.137637000	-1.813008000	1.735804000
C	2.420445000	2.522764000	0.578956000
C	3.289474000	3.128169000	-0.494862000
H	2.095277000	0.501077000	1.254964000
H	2.500829000	3.123384000	1.510518000
H	2.921451000	4.012485000	-1.007039000
N	2.771587000	1.129113000	0.793136000
